# Clonal Complexes Distribution of *Staphylococcus aureus* Isolates from Clinical Samples from the Caribbean Islands

**DOI:** 10.3390/antibiotics12061050

**Published:** 2023-06-14

**Authors:** Stefan Monecke, Patrick Eberechi Akpaka, Margaret R. Smith, Chandrashekhar G. Unakal, Camille-Ann Thoms Rodriguez, Khalil Ashraph, Elke Müller, Sascha D. Braun, Celia Diezel, Martin Reinicke, Ralf Ehricht

**Affiliations:** 1 Leibniz Institute of Photonic Technology (IPHT), 07745 Jena, Germany; 2 InfectoGnostics Research Campus, 07743 Jena, Germany; 3 Institute for Medical Microbiology and Virology, Dresden University Hospital, 01307 Dresden, Germany; 4 Department of Para-Clinical Sciences, Faculty of Medical Sciences, St. Augustine Campus, The University of the West Indies, St. Augustine, Trinidad and Tobago; 5 Department of Microbiology, Faculty of Medical Sciences, Mona Campus, The University of the West Indies, Kgn7, Kingston, Jamaica; 6 Institute of Physical Chemistry, Friedrich-Schiller University, 07743 Jena, Germany

**Keywords:** *Staphylococcus aureus*, MRSA, *mecA*, SCC*mec* element, Panton–Valentine leukocidin (PVL)

## Abstract

The aim of this study was to comprehensively characterise *S. aureus* from the Caribbean Islands of Trinidad and Tobago, and Jamaica. A total of 101 *S. aureus*/*argenteus* isolates were collected in 2020, mainly from patients with skin and soft tissue infections. They were characterised by DNA microarray allowing the detection of ca. 170 target genes and assignment to clonal complexes (CC)s and strains. In addition, the in vitro production of Panton–Valentine leukocidin (PVL) was examined by an experimental lateral flow assay. Two isolates were identified as *S. argenteus*, CC2596. The remaining *S. aureus* isolates were assigned to 21 CCs. The PVL rate among methicillin-susceptible *S. aureus* (MSSA) isolates was high (38/101), and 37 of the 38 genotypically positive isolates also yielded positive lateral flow results. The isolate that did not produce PVL was genome-sequenced, and it was shown to have a frameshift mutation in *agrC*. The high rate of PVL genes can be attributed to the presence of a known local CC8–MSSA clone in Trinidad and Tobago (n = 12) and to CC152–MSSA (n = 15). In contrast to earlier surveys, the USA300 clone was not found, although one MSSA isolate carried the ACME element, probably being a *mecA*-deficient derivative of this strain. Ten isolates, all from Trinidad and Tobago, were identified as MRSA. The pandemic ST239–MRSA–III strain was still common (n = 7), but five isolates showed a composite SCC*mec* element not observed elsewhere. Three isolates were sequenced. That showed a group of genes (among others, *speG*, *crzC,* and *ccrA*/*B*-4) to be linked to its SCC element, as previously found in some CC5– and CC8–MRSA, as well as in *S. epidermidis*. The other three MRSA belonged to CC22, CC72, and CC88, indicating epidemiological connections to Africa and the Middle East.

## 1. Introduction

*Staphylococcus aureus*, a ubiquitous bacterium, is a common cause of hospital- and community-associated infections in humans around the world [1,2,3,4,5]. Bacterial infections caused by methicillin-resistant *S. aureus* (MRSA) have become a serious global healthcare problem [6,7]. Aside from resistance, which limits options for treatment, another cause for concern is its continuous evolution and global spread of emerging and/or virulent clones [1]. Indeed, MRSA strains are associated with a number of widespread or even pandemic lineages. To describe these lineages, clonal complexes (CCs) based on multilocus sequence typing (MLST; [8]) are identified. The classification of CCs is based on the sequencing of seven ubiquitous housekeeping genes and the analysis of their sequence variations [8]. In addition, Staphylococcal cassette chromosome *mec* (SCC*mec*) elements can be assigned to distinct types [9,10]. These are large mobile genetic elements that carry *mecA* or *mecC* genes, responsible for methicillin/beta-lactam resistance—thereby defining MRSA [11]—as well as other resistance and/or virulence-associated markers. Methicillin-resistant *S. aureus* infections are among the most challenging infection prevention and control issues, particularly in hospitals. Hospital and other healthcare-related infections are frequently severe and can be fatal [1]. Apart from hospital-acquired MRSA infections (HA–MRSA), community-acquired MRSA (CA–MRSA) infections are emerging as a human health problem in many countries and have been reported in people who have had little or no previous contact with healthcare systems [12,13,14,15,16,17]. Despite being—in many cases—more susceptible to antibiotics other than beta-lactams, CA–MRSA infections have been detected in hospitals and are responsible for a large percentage of hospital-onset MRSA infections [18,19,20,21,22]. Furthermore, these CA–MRSA strains have been reported to be more virulent than some HA–MRSA strains. This is usually attributable to the presence of Panton–Valentine leukocidin (PVL) [5,17,23,24,25,26]. This is a pore-forming bicomponent toxin with high specificity to human leukocytes. It is associated with severe, chronic, or recurrent skin and soft tissue infections as well as with highly lethal but rare necrotising pneumonia. The PVL genes (*lukF*–PV and *lukS*–PV) are localised on prophages, and thus, they can horizontally be transferred across different strains and lineages of *S. aureus* [27,28,29,30].

The knowledge on *S. aureus* population structure and circulating MRSA clones for Caribbean islands is limited, with few published studies discussing a rather low number of typed isolates. On Francophone islands (Guadeloupe and Martinique), MRSA strains were observed that were also common in France (CC5 “Geraldine and CC8 “Lyon” clones as well as PVL-positive CC80–MRSA–IV; [31,32]). Regarding MSSA, several lineages were identified, including CC5, CC7, CC152, CC188, and CC398 [32]. A study from St. Kitts and Nevis showed high rates of the USA300/ST8–MRSA–IV (PVL+/ACME+) strain [33]. This strain was also observed in Cuba [34], alongside ACME negative, PVL-positive CC8–MRSA–IV and CC72–MRSA–V in hospitals [35] as well as other CC8–MRSA–IV and CC5–MRSA–IV in livestock [36]. In Barbados, high rates of PVL-positive *S. aureus* and rather high MRSA rates were observed [37]. A study from Haiti revealed a presence of CC5–MRSA–II and -IV clones of CC72–MRSA–IV as well as of various MSSA lineages, including CC1, CC5, CC8, CC15, CC45, CC72, and, notably, PVL-positive CC152 [38]. In a study from the Dominican Republic [32], CC5–, CC30–, and CC72–MRSA predominated, while PVL-positive CC30–MSSA was the dominant strain among a variety of MSSA lineages.

In Trinidad and Tobago, most *S. aureus* strains identified during previous studies were methicillin-susceptible *S. aureus* (MSSA) that were assigned to various CCs [39]. Virulent community-acquired MSSA is a cause for concern as PVL genes are common, and thus they pose an interesting issue for investigation. This follows a confirmed case of fatal multi-organ failure involving a young, previously healthy child that has been documented in the literature [40]. Among MRSA, the HA–MRSA strain ST239–MRSA–III was most prevalent, with other clones such as the CC5–MRSA–II being sporadically identified [41]. Further research on MRSA strains by Akpaka et al. [42] revealed the existence of a pulse-field gel electrophoresis (PFGE) banding pattern similar to that of a Canadian strain, CMRSA–6 (corresponding to a variant of ST239). In that study, all 60 isolates were PVL-negative [42]. In Trinidad and Tobago, an increase in the emergence of CA–MRSA infections has been reported, with prevalence rising from 4.1% to 8.1% between 1999 and 2004 [43]. The emerging CA–MRSA clone USA300/ST8–MRSA–IV (PVL+/ACME+) was also identified [41]. Available data suggest that the prevalence of MRSA-related infections appears to be increasing in Trinidad, Tobago, and Jamaica [31,44,45]. In Trinidad and Tobago, a prevalence rate of 4.6% was reported by Swanston in 1995 [46]. In 2006, a 12.8% prevalence rate was reported by Akpaka et al. [47], while Orrett [43] reported a prevalence of 20.8% in the same year.

In Jamaica, the prevalence of MRSA has continued to increase since the identification of the first case at the University Hospital of the West Indies in 1988 [48]. Reports emanating from Jamaica show that MRSA has fairly remained very low in prevalence, with 4% (2004), 5% (2005), and 7% in 2008 [44,45]. This is even lower at the teaching hospital where of 7304 clinical isolates analysed, 689 were identified as *S. aureus,* with only 31 (4.5%) proved to be methicillin-resistant in 2017 (unpublished data). This is comparable to the 3% prevalence found in 2013. In Jamaica, commonly identified CCs include ST8–MRSA–IV, USA300, and ST5/ST225–MRSA–II, New York–Japan clone [31].

In general, knowledge of the population structure of *S. aureus*/MRSA on the Caribbean islands is poor, and changes to it might remain unnoticed. In order to address this, DNA microarray-based analysis was used in this study to classify and assign clinical *S. aureus* isolates from Trinidad and Tobago, and Jamaica into clonal complexes, strains, and SCC*mec* types. In addition, the presence and expression of PVL were studied.

## 2. Results

### 2.1. Analysis of Clinical Isolates

Just over half of the specimens analysed from Trinidad and Tobago (48/85; 57%) were recovered from wound swabs. The remaining isolates were recovered from blood (12/85; 14%), pus (7/85; 8%), urine (8/85; 9%), ear swabs, vulvar swabs, peritoneal swabs, joint fluid, eye swabs, and, in one case, from an undocumented specimen (10/85; 12%). Most of the Jamaican isolates (13/16; 81%) were from wounds, while the remaining isolates were recovered from a throat swab (1/16; 6%) and blood (2/16; 13%).

Table 1 and Table 2 show the specimen source distribution of MSSA, MRSA, and *S. argenteus* isolates from both islands. Table 3 shows the age distribution of patients infected with MSSA, MRSA, and *S. argenteus* strains from Trinidad and Tobago. Patients aged 30 to 39 years had the highest rate of *S. aureus* infections (22%; 19/85). Of the 19 patients, 95% of infections were caused by MSSA strains. MRSA accounted for one infection (5%) in this group. The second group of patients that frequently experienced infections were those in the paediatric group (0 to 9 years) and also those between 10 and 19 years of age. Both groups had 18% of patients being infected. In these groups, MRSA accounted for 13% (2/15) and 7% (1/15) of infections, while MSSA accounted for 80% (12/15) and 93% (14/15) of infections, respectively. MSSA infections were found in 92% of patients over the age of 60, while the remaining 8% were related to MRSA. *S. argenteus* infections were found in two patients, ages 6 and 45 years.

Data from Table 4 showed that males (n = 46) more commonly presented with staphylococcal infections than women. MSSA infections were also most prevalent in both males and females as opposed to MRSA, with 89% (41/46) occurring in males and 81% (30/37) in females. MRSA infections were found in 9% (4/46) of male patients and 16% (6/37) of female patients. *S. argenteus* isolates accounted for 2% (1/46) of infections in males and 3% (1/37) in females.

### 2.2. Distribution of S. aureus Clonal Complexes and Strains

High clonal diversity was reported from Trinidad and Tobago, as *S. aureus* isolates were assigned to twenty different clonal complexes and 83 strains comprising both MRSA and MSSA (Table 5). Two additional isolates were identified as *S. argenteus*, CC2596. The most prevalent CC identified was CC8 (15 isolates), followed by CC152 (14 isolates). Other CCs included CC97, CC6, and CC239, which comprised the majority of MRSA. The remaining CCs were rare, comprising five or fewer isolates each. All 16 isolates (100%) from Jamaica were MSSA and distributed across eight different CCs. CC1 (four isolates) and CC6 (three isolates) were the most preventable among these isolates; CC152 was also found, but CC8 was not identified. However, the numbers are too low to assess the population structure of *S. aureus*/MSSA on this island.

The overall distribution of CCs from both islands, the various strains identified, as well as results for the resistance and virulence markers, SCC*mec* elements, and toxins examined can be found in the Supplemental File S1 attached, and summarized in Table 5.

Most of the strains from Trinidad and Tobago were MSSA (73/85; 86%), and two were methicillin-susceptible *S. argenteus*. All isolates from Jamaica were MSSA (16/16; 100%).

Of the 85 clinical isolates from Trinidad and Tobago, only ten (10/85; 12%) strains were identified as methicillin-resistant *S. aureus* (Table 5). The majority (7/10; 70%) belonged to CC239, while the three remaining strains were categorized as CC22, CC72, and CC88. There were no MRSA strains isolated from Jamaica.

### 2.3. Observations Regarding Individual Clonal Complexes of S. aureus

Four isolates from Jamaica and one from Trinidad and Tobago belonged to CC1. All were MSSA, all lacked the SCC-borne fusidic-acid resistance gene *fusC,* and all were PVL-negative. All carried the enterotoxin genes *seh*, *sek + seq,* and, in addition, all Jamaican isolates were also positive for the toxic shock syndrome toxin gene *tst1*, as well as for the enterotoxin genes *sec* and *sel.*

Three MSSA isolates from Trinidad and Tobago were assigned to CC5. Two of them carried, in addition to PVL, the *edinA* gene encoding an epidermal cell differentiation inhibitor.

PVL-positive as well as PVL-negative CC6–MSSA were found in Trinidad and Tobago as well as in Jamaica. Six isolates from this CC harboured PVL genes; five were positive for the enterotoxin A gene *sea*.

CC7 was represented by five MSSA isolates from Trinidad and Tobago. All were PVL-negative but harboured a *sea* allele as known from N315 (BA000018.3; pos. 2,011,380 to 2,012,153), also known as enterotoxin P or *sep*.

All CC8 isolates originated from Trinidad and Tobago; not a single one was recovered from Jamaica. Twelve isolates belonged to a previously noticed CC8–MSSA that harboured PVL genes, enterotoxin genes *sek* and *seq,* as well as (in 11/12) also *sed*, *sej* and *ser*. Both sequenced isolates showed *sed*, *sej,* and *ser* to be located on the same contig as *blaZ + blaI + blaR* together with plasmid genes and a cadmium resistance operon. A single isolate out of these twelve genotypically PVL-positive isolates was negative for PVL production, as discussed below. Another two CC8–MSSA isolates were negative for PVL and enterotoxin genes.

CC8–MRSA were not noted, neither “North American” (ACME-positive) nor “South American (ACME-negative/*mer*-operon-positive) USA300 strains. However, one additional PVL-positive isolate carried ACME genes, the SCC-associated copper resistance gene *copA2*–SCC, *opp3B*, *opp3C*, *adhC,* and *speG*, but lacked *mecA* as well as all genes associated with SCC*mec* IV. This suggests that it might have been a *mecA-*deficient derivative of the North American USA300 strain.

A Clonal Complex 22 MRSA isolate was retrieved from a patient presenting with cellulitis/trauma to the left foot. It was also the only isolate assigned to this common and widespread lineage and the only PVL- and *tst1*- (toxic shock syndrome toxin 1) positive MRSA identified. It harboured various genes associated with SCC*mec,* which encodes methicillin resistance. These include *ugpQ*, *mecA*, the truncated methicillin resistance operon repressor 1, *ccrA*/*B*-2, and *Q9XB68dcs*. Subtyping by an additional array [49] identified the SCC*mec* element as SCC*mec* IVa.

A total of three CC72 isolates were identified; however, only one was methicillin-resistant. This was a PVL-negative strain with a composite SCC*mec* VT element comprising *ugpQ*, *mecA,* and *ccrC* genes that also included the fusidic acid resistance gene *fus*C (=Q6GD50). The probe for D1GU38, a marker that accompanies the second copy of *ccrC* in SCC*mec* VT, yielded a signal indicating that the composite element derived from SCC*mec* VT rather than V. The other two CC72 isolates were MSSA that lacked PVL but carried enterotoxin genes *sec* and *sel.*

A CC88–MRSA–IV was the only isolate of this lineage collected from a patient with a vulval abscess. As it carried a SCC*mec* IVa (MW2-like) element, this observation suggested an African connection. Except for the beta-lactamase operon *blaZ*/*I*/*R*, no other resistance genes were detected. Neither PVL nor enterotoxin or exfoliative toxin genes were detected.

Sixteen isolates (including two from Jamaica) belonged to CC152. All were MSSA, but 13 harboured the penicillinase operon. A total of 15 out of 16 were positive for PVL genes, and these 15 also yielded phenotypically detectable PVL. In addition, all were positive for the *edinB* gene.

CC239 comprised seven CC239–MRSA–III isolates. The majority (n = 5) of CC239 isolates carried a complex SCC [*mec* III + *spe*G + Cd/*czrC + ccrAB4 + ccrC*] element. Three of these isolates were sequenced (see below). Four isolates from this cluster also harboured a mercury resistance operon that, however, in two sequenced isolates, was plasmid-borne rather than SCC-associated.

The other two CC239 isolates carried SCC [III + SCC*mer* + *ccr*C] elements but lacked *czr*C, *spe*G, and the additional recombinase genes. Four of the seven isolates (representing both strains) were tested for *sasX* = *sesI*, and all were positive in accordance with an affiliation to the “Southeast Asian Clade” of CC239–MRSA–III.

The remaining Clonal Complexes, CC9, CC12, CC15, CC30, CC59, CC101, CC121, and CC188, accounted for sporadic PVL- and *mecA-*negative isolates. Details are provided in Table 5 and Supplemental File S1.

### 2.4. Observations Regarding S. argenteus

Two isolates were assigned to *S. argenteus*. They yielded signals for *agr* III and capsule type 5 alleles, harboured *cna* but lacked the *egc* enterotoxin gene cluster as the genome sequence of H115100079; GenBank CCEP/SAMEA1557135 does. Thus, they were assigned to CC2596. Both isolates lacked PVL genes, and any resistance or enterotoxin genes were covered by the array.

### 2.5. Detection of PVL

**Figure 1:**
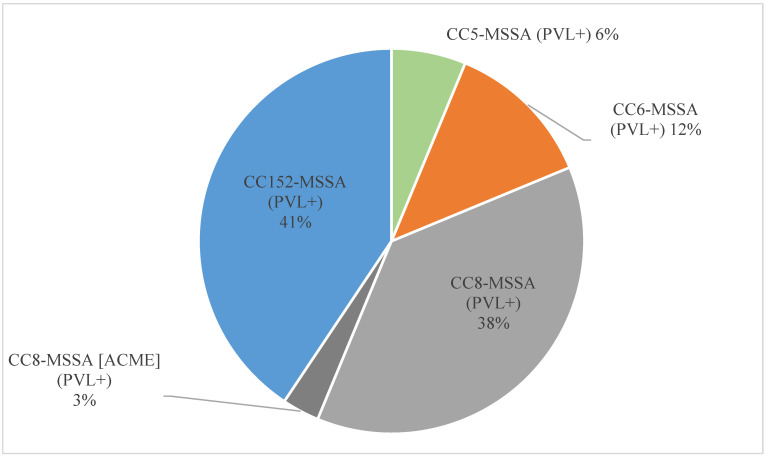
Clonal distribution of PVL-positive MSSA strains from Trinidad and Tobago.

Among isolates from Trinidad and Tobago (Figure 1), PVL genes were common, albeit no MRSA and no *S. argenteus* with these genes were identified. Thirty-two out of 73 (44%) MSSA isolates harboured *lukF*/*S–*PV genes. PVL genes were found in CC5, CC6, CC8, and CC152, with CC8 and CC152 being the dominant lineages among PVL positives (thirteen isolates each). Of these isolates, CC8 and CC152 were the largest contributors of PVL, with thirteen PVL-positive strains each. However, the CC8 strain commonly found in Trinidad and Tobago was not found among the Jamaica isolates. PVL-positive isolates from that island belonged to CC6 and CC152.

While thirty-two isolates carried *lukF*/*S–PV*, only thirty-one were found to be phenotypically positive for LukF–PV production by the lateral flow. Thus, the lateral flow assay—compared to genotyping—yielded a sensitivity of 96.9%, a specificity of 100%, a positive predictive value of 100%, and a negative predictive value of 98.6%. The one genotypically positive but phenotypically negative isolate (a CC8–MSSA; 2020-042_7641M) was re-cultured and re-tested, but the results remained unchanged. It was then subjected to genome sequencing, in parallel to a pheno- and genotypically PVL-positive isolate of the same strain, in order to find a reason for the discrepant results. It showed a frameshift mutation in *agrC* because of a deletion of a single nucleotide (see Supplemental File S2, 2020-042_7641M chromosome, positions 2,055,453 to 2,056,744; whereas *agrC* of a phenotypically PVL positive control can be found at Supplemental File S2, 2020-043_7352M, chromosome, pos. 2,099,143 to 2,100,435).

The other *agr* genes (*agrA*, *agrB,* and *agrD*) and *hld* were inconspicuous (for an alignment to the sequences of reference strain NCTC8325 and PVL-producing 2020-043_7352M, see Supplemental File S3), as were the *lukF–PV* and *lukS–PV* genes.

### 2.6. Sequencing the Composite SCCmec Element in Clonal Complex 239 Isolates

**Figure 2:**
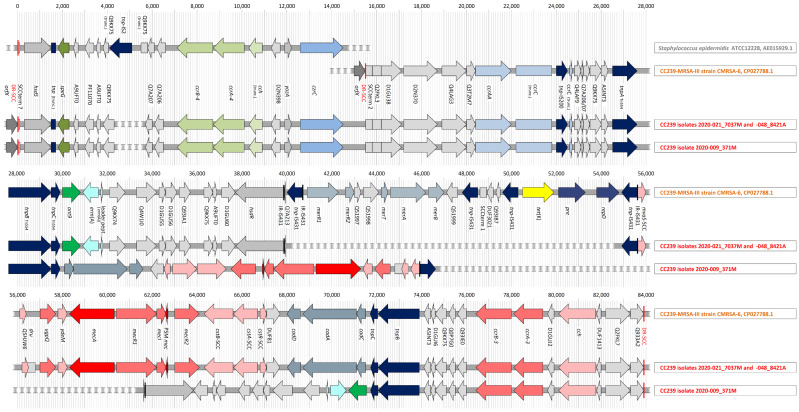
Schematic representation of a part of the SCC element in *S. epidermidis* ATCC12228 (GenBank AE015929.1; positions 51,506 to 66,000) and the SCC*mec* elements in CMRSA-6 (GenBank CP027788.1) as well as the three sequenced CC239 study strains.

Three CC239 isolates (2020-021_7037M, 2020-048_8421A, and 2020-009_371M) were sequenced (Supplemental File S2) to characterise the novel composite SCC*mec* element. It encompassed approximately 67,500 base pairs. Its gene content is summarised in Table 6, and a graphical overview is provided in Figure 2.

It comprised, directly adjacent to *orfX*, a gene encoding a type I restriction–modification system site-specificity determinate, *hsdS*, followed by a truncated transposase gene, the spermidine N-acetyltransferase gene *speG*, five genes encoding “putative proteins” (one of which was present in two copies), and recombinase genes *ccrA*/*B-4.* These are followed by a truncated *ccr-*associated cassette chromosome helicase gene (*cch*), yet another gene encoding a “putative protein,” as well as by *yozA* (HTH-type transcriptional repressor) and *czrC* (cadmium and zinc resistance gene C, formerly known as *cadA* or *copA*; [50]).

The remaining part of the composite SCC*mec* III element of the Trinidad and Tobago strain was essentially identical to the corresponding part of the SCC*mec* element of the Southeast Asian Clade CC239 strain TW20, FN433596.1:(34140 to 48481) and in CMRSA-6 (CP027788.1). Besides the *mec* element (including *mecA*, *mecI*, *mecR2* and *mecR1*, *psm*MEC, *ydeM*, *ugpQ*, and the *dru* region) and recombinase genes *ccrA*/*B-3* as well as *ccrAA*/*C,* it harboured the aminoglycoside resistance gene *ant9* and the macrolide/clindamycin resistance gene *erm*(A) as well as a cadmium-resistance operon (*cadC*, *cadA*, *cadD*).

Contrarily to TW20 and CMRSA-6, where the mercury resistance operon is localised on the SCC*mec* element, it was plasmid-borne in two of the three sequenced strains, and absent in the third one. When present, it was accompanied by yet another set of cadmium resistance genes and by quaternary ammonium compound resistance markers (*qacA*/*R*).

In one of the three isolates sequenced (2020-009_371M), a region comprising roughly 27,000 base pairs flanked on both sides by multiple transposase genes was inverted in order and orientation. It contained the *mec* element, the cadmium, and *cstR*/*A*/*B–SCC* operons, as well as *ant*9 and *erm*(A).

**Table 6:** SCC*mec* III composite elements in three CC239 isolates from Trinidad and Tobago.

Gene ID	Gene Product/Explanation	Position in SCC of 2020-021_7037M	Position in SCC of 2020-048_8421A	Orientation in 2020-021_7037M and 048_8421A	Position in SCC of 2020-009_371M	Orientation in 2020-009_371M	Present in ATCC1228, M1, etc.
DR-SCC	direct repeat of SCC	1 to 19	1 to 19	n/a *	1 to 19	n/a	-
SCCterm07	terminus of SCC towards *orfX*	20 to 122	20 to 122	n/a	20 to 122	n/a	X **
*hsdS*	type I restriction-modification system site-specificity determinate	256 to 1458	256 to 1458	forward	256 to 1458	forward	X
*tnp* trnc.	transposase for IS1272	1513 to 1712	1513 to 1712	trnc.	1513 to 1712	trnc.	X
*speG*	spermidine N-acetyltransferase	1781 to 2278	1781 to 2278	rev. compl.	1781 to 2278	rev. compl.	X
A9UFT0	LPXTG protein homologue	2531 to 2753	2531 to 2753	rev. compl., trnc., frameshift	2531 to 2753	rev. compl., trnc., frameshift	X
PF11070	putative PF11070 family protein	2950 to 3360	2950 to 3360	rev. compl.	2950 to 3360	rev. compl.	X
A9UFT0	LPXTG protein homologue	3523 to 3743	3523 to 3743	rev. compl.	3523 to 3743	rev. compl.	X
Q9KX75	putative protein	3758 to 4261	3758 to 4261	rev. compl.	3758 to 4261	rev. compl.	X
Q7A207	putative protein	4277 to 4588	4277 to 4588	rev. compl.	4277 to 4588	rev. compl.	X
Q7A206	putative protein	4675 to 5025	4675 to 5025	rev. compl.	4675 to 5025	rev. compl.	X
UTR*_ccrB-4*	conserved 3’-untranslated region of *ccrB*	5026 to 5525	5026 to 5525	n/a	5026 to 5525	n/a	X
*ccrB-4*	cassette chromosome recombinase B, type 4	5526 to 7154	5526 to 7154	rev. compl.	5526 to 7154	rev. compl.	X
*ccrA-4*	cassette chromosome recombinase A, type 4	7151 to 8512	7151 to 8512	rev. compl.	7151 to 8512	rev. compl.	X
*cch*	cassette chromosome helicase	8699 to 9334	8699 to 9334	rev. compl., trnc.	8699 to 9335	rev. compl., trnc.	X
D2N398	putative protein	9787 to 10,146	9787 to 10,146	rev. compl.	9787 to 10,146	rev. compl.	X
*yozA*	HTH-type transcriptional repressor	10,353 to 10,679	10,353 to 10,679	forward	10,353 to 10,679	forward	X
*czrC*	cadmium and zinc resistance gene C	11,000 to 12,934	11,000 to 12,934	forward	11,000 to 12,934	forward	X
SCCterm 02	terminus of SCC towards *orfX*	13,941 to 14,257	13,941 to 14,257	n/a	13,941 to 14,257	n/a	-
Q2FKL3	HNH endonuclease family protein	14,258 to 14,624	14,258 to 14,624	trnc., frameshift	14,258 to 14,624	trnc., frameshift	-
D1GU38	putative protein	14,689 to 15,552	14,689 to 15,552	forward	14,689 to 15,552	forward	-
D2N370	putative protein	15,660 to 17,134	15,660 to 17,134	forward	15,660 to 17,134	forward	-
Q4LAG3	putative protein	17,361 to 18,461	17,361 to 18,461	forward	17,361 to 18,461	forward	-
Q3T2M7	putative protein	18,454 to 18,825	18,454 to 18,825	forward	18,454 to 18,825	forward	-
*ccrAA*	cassette chromosome recombinase “AA”	18,822 to 20,465	18,822 to 20,465	forward	18,822 to 20,465	forward	-
*ccrC*	cassette chromosome recombinase C	20,690 to 22,292	20,690 to 22,292	forward, trnc.	20,690 to 22,292	forward, trnc.	-
*tnp*_IS200	transposase of IS200	22,423 to 22,908	22,423 to 22,908	forward	22,422 to 22,907	forward	-
*ccrC*	cassette chromosome recombinase C	23,030 to 23,111	23,030 to 23,111	forward, trnc.	23,029 to 23,110	forward, trnc.	-
Q4LAF9	putative protein	23,200 to 23,538	23,200 to 23,538	forward	23,199 to 23,537	forward	-
Q7A206	putative protein	23,544 to 23,630	23,544 to 23,630	forward, trnc.	23,543 to 23,629	forward, trnc.	-
Q7A207	putative protein	23,632 to 23,943	23,632 to 23,943	forward	23,631 to 23,942	forward	-
Q9KX75	putative protein	23,959 to 24,465	23,959 to 24,465	forward	23,958 to 24,464	forward	-
A5INT3	putative protein	24,486 to 24,803	24,486 to 24,803	forward	24,485 to 24,802	forward	-
*tnpA*_Tn554	transposase A of transposon Tn554	24,922 to 26,007	24,922 to 26,007	forward	24,921 to 26,006	forward	-
*tnpB*_Tn554	transposase B of transposon Tn554	26,004 to 27,896	26,004 to 27,896	forward	26,003 to 27,895	forward	-
*tnpC*_Tn554	transposase C of transposon Tn554	27,903 to 28,280	27,903 to 28,280	forward	27,902 to 28,279	forward	-
*ant*9	adenyltransferase AAd9	28,431 to 29,213	28,431 to 29,212	forward	54,251 to 55,033	rev. compl.	-
*erm*(A)	rRNA adenine N-6-methyltransferase	29,339 to 30,070	29,338 to 30,069	rev. compl.	53,394 to 54,125	forward	-
lp_*erm*(A)	leader peptide of *erm*(A)	30,128 to 30,187	30,127 to 30,186	rev. compl.	53,277 to 53,336	forward	-
Q9KX74	putative methyltransferase	30,579 to 31,241	30,578 to 31,240	forward	52,223 to 52,885	rev. compl.	-
Q4W1I0	putative DNA binding regulator	31,789 to 32,661	31,788 to 32,660	forward	50,803 to 51,675	rev. compl.	-
D1GU55	putative membrane protein	32,709 to 33,008	32,708 to 33,007	forward	50,456 to 50,755	rev. compl.	-
D1GU56	putative protein	33,024 to 33,512	33,023 to 33,511	forward	49,952 to 50,440	rev. compl.	-
Q93IA1	putative membrane protein	33,647 to 34,174	33,646 to 34,173	forward	49,290 to 49,817	rev. compl.	-
Q9KX75	putative protein	34,753 to 35,105	34,752 to 35,104	forward trnc.	48,358 to 48,711	rev. compl., trnc., frameshift	-
A9UFT0	LPXTG protein homologue	35,119 to 35,340	35,118 to 35,339	forward	48,123 to 48,344	rev. compl.	-
D1GU60	putative protein	35,490 to 36,056	35,489 to 36,055	forward	47,407 to 47,973	rev. compl.	-
*hsdR-*SCC	trnc. fragment of *hsdR*	36,136 to 38,349	36,135 to 38,348	rev. compl. trnc.	45,114 to 47,327	forward, trnc.	-
IR_IS431	inverted repeat of IS431	38,352 to 38,367	38,351 to 38,366	n/a	45,096 to 45,111	n/a	-
Q7A213	putative protein	38,352 to 38,379	38,351 to 38,378	forward trnc. frameshift	45,084 to 45,111	rev. compl., trnc., frameshift	-
*tnp*_IS431	transposase for IS431	38,411 to 39,085	38,410 to 39,084	rev. compl.	44,378 to 45,052	forward	-
IR_IS431	inverted repeat of IS431	39,116 to 39,149	39,115 to 39,148	trnc.	44,322 to 44,337	trnc.	-
Teg143	trans-encoded RNA associated with tnpIS431	39,126 to 39,141	39,125 to 39,140	n/a	44,314 to 44,347	n/a	-
*mvaS-*SCC	trnc. 3-hydroxy-3-methylglutaryl CoA synthase	39,158 to 39,510	39,157 to 39,509	forward, frameshift	43,953 to 44,305	rev. compl., frameshift	-
Q5HJW6	putative protein	39,608 to 39,911	39,607 to 39,910	forward trnc.	43,552 to 43,855	rev. compl., trnc.	-
*dru*	SCC direct repeat units	39,748 to 40,305	39,747 to 40,304	n/a	43,158 to 43,715	n/a	-
*ugpQ*	glycerophosphoryl diester phosphodiesterase	40,507 to 41,250	40,506 to 41,249	forward	42,213 to 42,956	rev. compl.	-
*ydeM*	putative dehydratase	41,347 to 41,775	41,346 to 41,774	forward	41,688 to 42,116	rev. compl.	-
*mecA*	penicillin-binding protein 2a	41,821 to 43,827	41,820 to 43,826	rev. compl.	39,637 to 41,642	forward	-
*mecR1*	methicillin resistance operon repressor 1	43,927 to 45,684	43,926 to 45,683	forward	37,779 to 39,537	rev. compl.	-
*mecI*	methicillin resistance regulatory protein	45,684 to 46,055	45,683 to 46,054	forward	37,408 to 37,779	rev. compl.	-
*psm*MEC	phenol-soluble modulin from SCC*mec*	46,140 to 46,208	46,139 to 46,207	rev. compl.	37,255 to 37,323	forward	-
*mecR2*	methicillin resistance operon repressor 2	46,528 to 47,676	46,527 to 47,675	forward	35,786 to 36,935	rev. compl.	-
*cstB-*SCC	CsoR-like sulphur transferase-regulated gene B	47,790 to 49,124	47,789 to 49,123	rev. compl.	34,338 to 35,672	forward	-
*cstA*-SCC	CsoR-like sulphur transferase-regulated gene A	49,156 to 50,220	49,155 to 50,219	rev. compl.	33,242 to 34,306	forward	-
*cstR-*SCC	copper-sensing transcriptional repressor	50,356 to 50,616	50,355 to 50,615	forward	32,846 to 33,106	rev. compl.	-
DUF81-SCC	putative sulfite/sulfonate efflux	50,616 to 51,259	50,615 to 51,258	forward	32,203 to 32,846	rev. compl.	-
*cadD*_R35	cadmium transport protein D	51,527 to 52,144	51,526 to 52,143	rev. compl. frameshift	31,318 to 31,935	forward	-
*cadA*_Tn554	cadmium efflux adenosine triphosphatase	52,225 to 54,639	52,224 to 54,638	rev. compl.	28,823 to 31,237	forward	-
*cadC_*Tn554	putative regulator of cadmium efflux	54,632 to 54,997	54,631 to 54,996	rev. compl.	28,465 to 28,830	forward	-
*tnpC*_Tn554	transposase C of transposon Tn554	55,183 to 55,561	55,182 to 55,559	rev. compl.	55,184 to 55,561	rev. compl.	-
*tnpB_*Tn554	transposase B of transposon Tn554	55,568 to 57,460	55,566 to 57,458	rev. compl.	55,568 to 57,460	rev. compl.	-
A5INT3	putative protein	57,655 to 57,978	57,653 to 57,976	rev. compl.	57,655 to 57,978	rev. compl.	-
D1GUI6	putative protein	57,971 to 58,177	57,969 to 58,175	rev. compl.	57,971 to 58,177	rev. compl.	-
Q9KX75	putative protein	58,179 to 58,700	58,177 to 58,698	rev. compl.	58,179 to 58,700	rev. compl.	-
Q0P7G0	putative protein	58,719 to 59,030	58,717 to 59,028	rev. compl.	58,719 to 59,030	rev. compl.	-
Q93IE0	putative protein	59,115 to 59,465	59,113 to 59,463	rev. compl.	59,115 to 59,465	rev. compl.	-
*ccrB-3*	cassette chromosome recombinase B, type 3	59,935 to 61,562	59,933 to 61,560	rev. compl.	59,936 to 61,563	rev. compl.	-
*ccrA-3*	cassette chromosome recombinase A, type 3	61,583 to 62,929	61,581 to 62,927	rev. compl.	61,584 to 62,930	rev. compl.	-
D1GUJ2	putative protein	63,122 to 63,397	63,120 to 63,395	rev. compl.	63,123 to 63,398	rev. compl.	-
*cch*	cassette chromosome helicase	63,487 to 65,274	63,485 to 65,272	rev. compl.	63,488 to 65,275	rev. compl.	-
DUF1413	putative protein associated with *ccr*	65,274 to 65,561	65,272 to 65,559	rev. compl.	65,275 to 65,562	rev. compl.	-
Q2FKL7	putative protein	65,695 to 66,744	65,693 to 66,742	forward	65,696 to 66,745	forward	-
Q933A2	putative ADP-ribosyltransferase	66,797 to 67,369	66,795 to 67,367	forward	66,798 to 67,370	forward	-
DR-SCC	direct repeat of SCC	67,459 to 67,477	67,457 to 67,475	n/a	67,460 to 67,478	n/a	-

* n/, not applicable. Non-coding regions. ** Genes marked with ”X” are present in sequences of ATCC1228 and M1.

### 2.7. The sasX = sesI Gene in the Clonal Complex 239 Sequences

The *sasX = sesI* gene encoding an LPxTG motif “surface-anchored protein X” [51] was accompanied, likewise in all three sequences, by several copies of transposase genes, genes encoding integrases, holins, exonucleases, and amidases, as well as by the aminoglycoside resistance genes *aacA–aphD*, *aphA3*, and the streptothricin resistance gene *sat*. This region encompassed as many as roughly 127,000 bp and is integrated into *yeeE* = DUF395 (“SAUR2215 (SAR_RS11075)”; see https://pubmlst.org, accessed on 16 May 2023). This is the same constellation as in TW20 ([52]; FN433596, pos. 2,180,899 to 2,309,183) and in CMRSA-6 (CP027788.1).

## 3. Discussion

The DNA microarray analysis approach, along with MLST and SCC*mec* typing, provided a comprehensive characterization and assignment of all isolates into clonal complexes and strains. Some clinically useful data with respect to antimicrobial resistance, virulence, and toxin gene carriage were also revealed. Results confirmed a high diversity of *S. aureus* clonal complexes from Trinidad and Tobago, with CC8 being the most prominent. While most of the infections in Trinidad and Tobago were caused by MSSA (73/85; 86%), all were caused by MSSA in Jamaica. Although the sample size from both islands was relatively small, most of the findings were consistent when compared to previous studies conducted on *S. aureus* isolates from both islands on much larger sample sizes. In the present study, most of the *S. aureus* infections occurred in patients with wound infections, mainly post-surgical in nature. Similar findings have been reported previously by several authors from Trinidad and Tobago [42,43,46,47,53,54] and Jamaica [45]. The exact reason for this occurrence is unknown; some authors have outlined possible explanations in reports from Trinidad and Tobago. Akpaka et al. [47] attributed the high levels of infection to excessive wound swabbing following medical procedures, and, in another report, noted that some surgical procedures can result in nosocomial infections with *S. aureus* as the main aetiological agent [54]. According to Orrett [43], the hands of health care providers may contribute to infection during dressing exercises, whereas Swanston [46] attributed the high usage of antibiotics to the increase in MRSA isolates from the surgical wards at the Port of Spain General Hospital. While any of the reasons outlined may be plausible as a contributory factor, it re-emphasizes the importance of implementing a strict infection control policy and applying guidelines for the prevention of surgical site infections in hospitals. This would include some form of multifaceted approach involving all team members to provide quality service before, during, and after surgical procedures.

Of the studies emanating from Trinidad and Tobago, Akpaka et al. [54] discovered a high prevalence of *S. aureus* infections in children aged 0 to 9. This high infection rate was attributed to an underdeveloped immune system and its inability to recognize staphylococcal components during infection at that age. Usually, older people tend to be more vulnerable to infections because aging is associated with immune dysfunction, multiple comorbidities, increased hospitalizations, and increased antibiotic use. Ramdass et al. [55] reported, at the Port of Spain General Hospital Trinidad, a peak age range of 60 to 69 years, in which 93% (14/15) of the MRSA-positive patients had used antibiotics (mainly beta-lactams) prior to admission into the hospital, had comorbidities (diabetes and hypertension), had long hospital stays (>1 week), were previously hospitalized and had previous surgery. In Trinidad and Tobago, most infections occurred among patients aged 30 to 39. This was followed by 0 to 9, 10 to 19 and finally patients over 60 years of age. Similar to the risk factors associated with infants and elderly, who are most often confined to nurseries and nursing homes, overcrowding may be a contributing factor to the high infection rate among the 10 to 39 age group. Persons in this age group frequently congregate in crowded areas of the community where the risk and exposure to infection are increased, such as correctional facilities, military/army camps, schools, and sports club settings. In Trinidad and Tobago, Orrett [53] outlined other major contributory factors that could be applied; these include the inappropriate use of antimicrobials and patients discontinuing therapy after being discharged due to the cost of prescriptions at local pharmacies. Most *S. aureus* infections in this study occurred in males from Trinidad and Tobago. Though not statistically significant, high *S. aureus* infection rates among males have been reported previously in other studies from the island [54,55]. According to Akpaka et al. [47], no obvious reason for the impact of gender on the prevalence of MRSA or MSSA in community or hospital settings has been reported in the literature. However, similar observations also have been made in other parts of the world.

Reports on the molecular characteristics and prevalence of MRSA in hospitals and within the community are constantly expanding in Trinidad and Tobago. The 12% MRSA prevalence observed herein was consistent with previously reported rates ranging from 9.8% to 15.3% [39,42,47,53,54,56], although, in 2018, Vire et al. [57] recorded the highest reported rate for the island, 44.4%. The most common MRSA strain was CC239–MRSA–III (8.3% of all *S. aureus* isolates, and 70% of MRSA). It has already been identified as the most prevalent clone in Trinidad and Tobago [41,54,56]. Until 2004, one variant of this strain (CMRSA-6) was the only MRSA clone circulating in Trinidad and Tobago [42]. According to previous work [58,59], CC239 with SCC*mec* III is a major dominant hospital-associated MLST type that has been described as the oldest truly pandemic MRSA strain in circulation since the 1970s and as the most successful international epidemic clone of MRSA. Its clade harbouring the virulence factor *sasX*/*sesI* is known to be widespread in Southeast Asia [51], but it was also, surprisingly, present in Trinidad and Tobago [59]. In this study, isolates were also tested positive for this gene. Sequencing revealed the localisation of *sasX*/*sesI* on a large mobile element, together with aminoglycoside genes (*aacA–aphD* and *aphA*3) and genes for phage enzymes. However, genes encoding phage structural proteins, such as those forming the capsid or tail, were not noted. Essentially the same complex can be found in TW20 (FN433596.1) and CMRSA-6 (CP027788.1), confirming the previous observation on the close relationship of the Trinidad and Tobago CC239 strains to the “South East Asian” Clade of CC239–MRSA–III and CMRSA-6 [42].

The Trinidad and Tobago variant of the “Southeast Asian” CC230–MRSA–III lineage, as described herein, harbours a composite SCC*mec* element including *speG*, *czrC,* and *ccrA*/*B-*4 recombinase genes in addition to its SCC*mec* III element. This variant has, to the best of our knowledge, exclusively been described from Trinidad and Tobago. Apparently, it has been extant there for at least 10 years (see [41]; when six out of seventy-six CC239 isolates collected in 2010 yielded array hybridisation signals for *ccrA*/*B-4* genes).

The first 12,000 base-pairs of the SCC element of this strain are essentially identical to a “SCC–M1” element with *speG* and *crzC* that was found nearly years ago in two Irish epidemic CC8 strains, CC8–MRSA–[IIA/B/C/D/E + SCC–M1], “Irish AR13/14” and CC8–MRSA–[IVG/E + ccrAB4] (see [60] and GenBank HE858191.1, LS483301.1) and a Danish CC8–MRSA, (see [61] and GenBank HF937103.1). It was also identified in CC5, which includes MSSA with an SCC element harbouring *speG*, *czrC,* and *ccrA*/*B-*4 (GenBank CP053634.1), an MRSA strain from the US, where this element accompanies SCC*mec* II (see [62] and GenBank CP053636.1) and a Spanish epidemic MRSA strain in which it is linked to SCC*mec* IV (GenBank ASTH/SAMN02146299).

The *speG*, *czrC,* and *ccrA*/*B-4* cluster or “SCC–M1” element might have been transferred into *S. aureus* from *Staphylococcus epidermidis.* To the best of our knowledge, the oldest strain in which that element can be found is ATCC12228 (see [63] and GenBank AE015929.1), which was isolated many decades ago [64]. In ATCC12228, it differs only in an apparently random integration of a transposase gene, and it is not linked to a SCC*mec* element but to an SCC element carrying, among other genes, heavy metal resistance genes and a second copy of *speG*. The *speG*, *czrC,* and *ccrA*/*B-4* cluster is also present in several other sequences of *S. epidermidis,* including the one of strain ATCC14990 (GenBank CP035288.1), which was isolated in 1963 [65].

For two reasons, it might be speculated that the acquisition of this element might confer an evolutionary advantage. First, it recently appeared in several unrelated *S. aureus* strains in distant parts of the world. Second, in Trinidad and Tobago, it was noted in 6 out of 76 isolates collected in 2010, but in 5 out of 7 in 2020, indicating an increasing prevalence (with the caveat of low numbers). The gene *crzC* causes cadmium and zinc resistance [50], and it is frequently observed in livestock-associated MRSA [66], which might have benefited from resistance to formerly widely used zinc supplements to animal fodder. Whether the dermatological use of zinc-containing cremes and ointments might pose a selective pressure favouring this strain remains to be clarified. The *speG* gene, encoding a spermidine N-acetyltransferase, can also be found as part of ACME elements, such as in the USA300 strain and in composite SCC*mec* elements (see above and [67]). It has been associated with resistance to exogenous polyamines [68].

Similar to the CC239–MRSA–III, the rapidly emerging “USA300” CC8 (ST8–MRSA–IV) clone has been isolated previously in Trinidad and Tobago and appeared to be quite common [31,39,41,54,56]. This well-known pandemic CA–MRSA strain is common in various regions of the globe. A direct transfer of this strain from North American visitors was proposed as a reason for its high local prevalence [54], along with its emergence from the Caribbean/Latin American region [69]. However, in this study, no USA300 isolate was found. Only one CC8–MSSA isolates harboured both PVL and ACME in this study and was assumed to be a variant of “USA300” that lost its *mecA*/SCC*mec* IV element. Thus, it is tempting to speculate that the prevalence of this hypervirulent, multi-resistant clone in Trinidad and Tobago may be declining. The related but distinct “Spanish/Latin American USA300” CC8–MRSA–IV ([70], as represented by the genomes with GenBank accession numbers CP007670; CP007672), was also not detected despite geographical proximity.

Frequent domestic and international travel to Trinidad and Tobago has resulted in the discovery of several strains with global and regional connections. This study identified one CC88–MRSA–IVa strain, which has previously been reported in Africa and Australia [71]. The CC22–MRSA–IV (PVL+/*tst1*+) strain with a SCC*mec* IVa (MW2-like) element might indicate a Middle Eastern/Persian Gulf connection. It was observed in the Middle Eastern/Persian Gulf region and in Central Asia (see [72,73,74,75] as well as GenBank CP038850.1, from Pakistan). Another strain, CC72–MRSA–[VT + *fusC*], could also have been brought from there as composite SCC*mec* elements harbouring *fusC* are abundant in the Greater Middle East, and, indeed, similar or identical isolates have previously been observed from the UAE [76]. Findings of this nature should initiate a thorough investigation into a patient’s travel history, which should provide a clear indication of the strains’ possible origin. Moreover, a possible emergence of strains with composite SCC*mec* elements harbouring *fusC* should be closely monitored, as fusidic acid is commonly used in Trinidad and Tobago.

Another interesting observation is the high prevalence of PVL-positive *S. aureus*, reported consistently over the last decade with rates from 48% or 50% [39,77] to up to 62% [57]. In this study, the overall PVL prevalence for Trinidad and Tobago was lower, at 39%. CC8– and CC152–MSSA strains were the most common PVL-positive strains accounting for 15% and 14% of all Trinidad and Tobago isolates tested, with the former one apparently decreasing and the latter increasing compared to earlier studies [39,77]. The authors had already proposed an endemicity of the PVL-positive CC8–MSSA strains based on earlier observations [39], including the case of a severe and fatal infection in a previously healthy child [40]. Another common, endemic strain is PVL-positive CC152–MSSA. Interestingly, CC152 is an abundant lineage in Africa [78,79,80,81,82], and it was also observed in Haiti and Martinique [32,38]. Thus, it might be speculated that it was brought to the Caribbean islands together with people of African descent sometime in the history of colonisation. This might also apply to CC72. Since PVL-positive CC8– and CC152–MSSA has dominated Trinidad and Tobago for at least a decade [41], the rate of infection and possible patterns of evolution of these two clonal complex lineages will need to be closely monitored in the future.

While little information on PVL and MSSA is available for Jamaica, the 25% of PVL-positive MSSA strains identified should be monitored.

In Trinidad and Tobago’s literature, there are currently limited reports of *S. argenteus* strains. A low prevalence of *S. argenteus* strains to belong to CC2596 (2/85; 2.35%) was reported in this study, which is comparable with previous findings by Monecke et al. [39], where seven (7/294; 2.38%) *S. argenteus* strains were identified. When re-assessing hybridisation patterns of *S. argenteus* isolates from all Trinidad and Tobago studies by the authors, three can be assigned to CC2596 (23%, including the two from this study), three to CC1223 (23%) and seven to CC2250 (54%), but none to CC1850 (“ST75”, the longest known *S. argenteus* lineage). No *S. argenteus* isolate was found positive for *mecA* or PVL genes. Despite a low prevalence in both studies, *S. argenteus* should not be overlooked as this non-staphyloxanthin-producing strain [83], previously thought to be less virulent than *S. aureus*, is said to be becoming clinically important, with significant global prevalence and a virulence comparable to the one of *S. aureus* [84,85]. In the future, it is recommended that these strains should closely be monitored and reported [86]. The creation of a database within laboratories for the epidemiology, clinical impact, and implications for infection control of such isolates would be beneficial to combat potential outbreaks or increases in prevalence or virulence, given especially the observations of emerging PVL-positive [87] or methicillin-resistant *S. argenteus* [88,89]

Isolates in this study harboured a slew of resistance genes, virulence factors, and toxins. Despite this, all were vancomycin susceptible. This indicates that vancomycin remains the first-line treatment for (severe and/or bloodstream) infections with MRSA or with any *S. aureus* as long as susceptibility tests are pending, as previously reported from Trinidad and Tobago and Jamaica [44,47].

Unfortunately, with a strong local presence of virulent, PVL-positive strains in the community and the ongoing evolution of multidrug-resistant MRSA in healthcare settings, a need for vaccine development, ongoing surveillance, proper infection control, and reinforcement of preventative measures in both hospital and community settings, remains.

## 4. Materials and Methods

### 4.1. Study Design and Eligibility

This cross-sectional study was conducted using 101 clinical samples recovered from Trinidad and Tobago and Jamaica. Samples were collected from August to September 2020. All eligible subjects, regardless of ethnic group, gender, social status, or educational level, who agreed to participate by means of written consent and assent, were included in this study. This includes patients with varying clinical presentations who were diagnosed with various *S. aurei* infections such as furunculosis or carbuncles, as well as with cutaneous abscesses, mastitis, and other prominent and severe skin and soft tissue infections (SSTI; necrotizing fasciitis, chronically purulent and painful “spider bites”, particularly in cases with travel history) and recurrent or chronic SSTIs and necrotizing community-acquired pneumonia, including cases associated with influenza.

### 4.2. Laboratory Identification of Isolates

Samples were subjected to routine screening tests for the detection and isolation of *S. aureus*/*argenteus*. The clinical samples were cultured on mannitol fermentation salt agar at 37 °C for 24–48 h. Presumptive *S. aureus* was identified as gram-positive cocci isolates that produced yellow colonies with a yellow halo on mannitol salt agar. Colonies that were mannitol-salt-positive were conventionally identified as *S. aureus* based on colony morphology, Gram stain, haemolysis on sheep blood agar, catalase, and coagulase/latex agglutination tests.

Antibiotics susceptibility tests for sixteen antibiotics (including ampicillin, ampicillin + clavulanic acid, cefoxitin, ceftriaxone, cefuroxime, ceftazidime, ciprofloxacin, clindamycin, chloramphenicol, ertapenem, erythromycin, gentamicin, norfloxacin, penicillin, vancomycin, tetracycline) were routinely tested using the Kirby Bauer disc diffusion method on Mueller–Hinton agar in accordance with the Clinical and Laboratory Standards Institute (CLSI) guidelines. Cefoxitin 1 μg and oxacillin 30 μg antibiotic discs were used to screen for methicillin resistance. *S. aureus* strains ATCC 29213 and ATCC 25923 were used as controls.

All suspected *S. aureus*/*argenteus* samples were subjected to molecular testing using DNA microarray analysis as described below. This led to the exclusion of nine coagulase-negative *Staphylococcus spec*. isolates and the identification of two *S. argenteus* isolates, which were included in the analysis. An experimental lateral flow test was applied to all of these isolates to detect PVL expression (see below).

### 4.3. Microarray Analysis

The detection of virulence genes and resistance markers, as well as the assignment to clonal complexes, epidemic strains, and SCC*mec* types, was performed by microarray analysis using the StaphyType DNA microarray (Abbott [Alere Technologies GmbH], Jena, Germany) and the INTER-ARRAY Genotyping Kit *S.aureus* (Inter-Array GmbH, Bad Langensalza, Germany). The probes, primers, and procedures were previously described [2,49,90]. SCC*mec* subtyping and detection of *sasX* = *sesI*, using a second experimental array, was carried out on selected isolates as previously described [49]. In short, isolates were cultured overnight at 37 °C on Columbia blood agar. DNA was purified using Qiagen columns (Qiagen, Hilden, Germany) after enzymatic lysis of staphylococcal cells. The assay was based on a linear primer elongation that used one primer per target but amplified all targets simultaneously. During amplification, biotin-16-dUTP was incorporated into the single-stranded amplicons. After hybridisation to the DNA probes immobilised on the surface of the microarray, washing, and blocking, horseradish-peroxidase-streptavidin was conjugated. In case of a positive reaction, this conjugate bound to the biotin labels incorporated into the amplicons, causing in the next step a localised dye precipitation resulting in a visible and detectable formation. A transmission image of the microarray was recorded and analysed using a designated reader and software. This allowed the detection of individual target genes, as well as an automated comparison to a reference database facilitating assignment to CCs, strains, and SCC*mec* types [2,49,90].

### 4.4. Nanopore Sequencing

The Oxford Nanopore MinION platform was used to sequence the genomes of two MSSA isolates (2020-042_7641M, 2020-043_7352M) in order to investigate the lack of PVL production in the former. In addition, three CC239–MRSA (2020-009_371M, 2020-021_7037M, and 2020-048_8421A) were sequenced with the aim of describing their SCC*mec* elements.

The library preparation was performed using the 1D genomic DNA ligation kit (SQK-LSK109 with barcoding kit EXP-NBD104, and SQK-NBD114.24; Oxford Nanopore Technologies, Oxford, UK) following the manufacturer’s instructions for flow cells (FLO-MIN106 containing an R9.4.1 pore, and FLO-MIN114 containing an R10.4.1 pore). Prior to library preparation, a size selection was performed using AMPure-beads (Beckman Coulter, Krefeld, Germany) in a ratio of 1:1 (*v*/*v*) with the isolated DNA sample. The flow cell was loaded with a total of approximately 600 ng/µL DNA (according to Qubit4 Fluorometer; Thermo Fisher Scientific, Waltham, MA, USA). The sequencing ran for 72 h using MinKNOW software version 22.12.5, and 22.12.7 starting with a total of 1200–1600 active pores.

The guppy basecaller (version 6.4.6 + ae70e8f, Oxford Nanopore Technologies) was utilised to translate MinION raw reads (FAST5) into quality tagged sequence reads (4000 reads per FASTQ-file) using its barcode trimming option (model version: dna_r9.4.1_450bps_sup.cfg, and dna_r10.4.1_e8.2_400bps_sup). Flye (version 2.9.1-b1780) was used to assemble each strain’s quality tagged sequence reads into a complete, circular contig. Assemblies were polished in two steps. First, racon (v1.5.0) was iteratively used four times with the following parameters: match 8; mismatch 6; gap 8, and windows-lengths 500. Then, medaka (version 1.7.3) ran on the last racon-polished assembly using the model r941_min_sup_g507, and r10.4.1_e82_400bps_sup_g615. The resulting corrected assembly was used for further analysis.

### 4.5. PVL Lateral Flow Test

For the detection of PVL production, an experimental lateral flow assay (Senova, Weimar, Germany) was utilised as previously described [91]. In short, the assay relied on monoclonal antibodies targeting the LukF–PV protein. In order to test them, subcultured *S. aureus* isolates were incubated overnight or maximally up to 24 h at 37 ± 2 °C on Columbia Blood Agar. One inoculation loop (~10 µL) full of culture material was inoculated into 300 µL of the kit buffer and vortexed for 15–30 s in order to produce a homogenous suspension of cells. Then, the buffer with the inoculum was centrifuged at 2000× *g* for 30 s. A total of 100 µL of the supernatant was pipetted onto the sample well of the test device, and it was then incubated for 10 min at room temperature. The appearance of test and/or control lines was assessed visually, and the lateral flow device was photographed. The image of the test result was independently reviewed by two authors.

## 5. Conclusions

This study was conducted on 101 clinical *S. aureus*/*argenteus* isolates from Trinidad and Tobago and Jamaica. Samples were collected from August to September 2020. Ten isolates, all from Trinidad and Tobago, were identified as MRSA. The pandemic ST239–MRSA–III strain was still common (n = 7), but five of these isolates showed a novel composite SCC*mec* element in which a group of genes including *speG*, *crzC,* and *ccrA*/*B-4* was linked to SCC*mec* III. The prevalence of PVL genes was high (in 38/101 isolates), although lower than in previous studies from Trinidad and Tobago. The USA300 PVL–MRSA strain was not found anymore, while the predominant PVL-positive strains were CC6–, CC8–, and CC152–MSSA.

## Figures and Tables

**Table 1:** Specimen source distribution of MSSA, MRSA, and *S. argenteus* isolates from Trinidad and Tobago (%).

Strain	N (%)	W/Swab	Blood	Pus	Urine	Other
MSSA	73 (86)	41	9	7	8	8
MRSA	10 (12)	6	2	0	0	2
*S. argenteus*	2 (2)	1	1	0	0	0
Total	85 (100)	48 (57)	12 (14)	7 (8)	8 (9)	10 (12)

MRSA, Methicillin-resistant *Staphylococcus aureus*; MSSA, Methicillin-susceptible *Staphylococcus aureus*; N, number encountered; W/Swab, Wound swab.

**Table 2:** Specimen source distribution of MSSA and MRSA isolates from Jamaica (%).

Strain	N (%)	W/Swab	Throat	Blood
MSSA	16 (100)	13	1	2
MRSA	0 (0)	0	0	0
Total	16 (100)	13 (81)	1 (6)	2 (13)

MRSA, Methicillin-resistant *Staphylococcus aureus*; MSSA, Methicillin-susceptible *Staphylococcus aureus*; N, number encountered; W/Swab, Wound swab.

**Table 3:** Age group distribution of patients infected with MSSA, MRSA, and *S. argenteus* strains from Trinidad and Tobago.

Age Group (Years)	N (%)	MRSA	MSSA	*S. argenteus*
0–9	15 (18)	2	12	1
10–19	15 (18)	1	14	0
20–29	4 (5)	1	3	0
30–39	19 (22)	1	18	0
40–49	9 (11)	1	7	1
50–59	10 (12)	3	7	0
60–69	7 (8)	1	6	0
70–79	2 (2)	0	2	0
80+	3 (3)	0	3	0
Other/unrecorded	1 (1)	0	1	0
Total	85 (100)	10 (12)	73 (86)	2 (2)

MRSA, Methicillin-resistant *Staphylococcus aureus*; MSSA, Methicillin-susceptible *Staphylococcus aureus*; N, number encountered.

**Table 4:** Gender distribution of patients infected with MSSA, MRSA, and *S. argenteus* strains from Trinidad and Tobago.

Age Group (Years)	N (%)	MRSA	MSSA	*S. argenteus*
Male	46 (54)	4	41	1
Female	37 (44)	6	30	1
Not recorded	2 (2)	0	2	0
Total	85 (100)	10 (12)	73 (86)	2 (2)

MRSA, Methicillin-resistant *Staphylococcus aureus*; MSSA, Methicillin-susceptible *Staphylococcus aureus*; N, number encountered.

**Table 5:** Distribution of *S. aureus* clonal complexes, MSSA strains, and SCC*mec* elements as identified by array hybridisation for Trinidad, Tobago, and Jamaica.

CC	Strain	SCC*mec* Type	N (%) for Trinidad and Tobago	N (%) for Jamaica
CC1	CC1–MSSA	-	1 (1.2%)	4 (25%)
CC5	CC5–MSSA	-	1 (1.2%)	-
	CC5–MSSA (PVL+)	-	2 (2.4%)	-
CC6	CC6–MSSA	-	4 (4.7%)	1 (6.3%)
	CC6–MSSA (PVL+)	-	4 (4.7%)	2 (12.5%)
CC7	CC7–MSSA	-	5 (5.9%)	-
CC8	CC8–MSSA	-	2 (2.4%)	-
	CC8–MSSA (PVL+)	-	12 (14.1%)	-
	CC8–MSSA (ACME/PVL+)	ACME-I + *speG + adhC + copA*2	1 (1.2%)	-
CC9	CC9–MSSA	-	1 (1.2%)	-
CC12	CC12–MSSA	-	1 (1.2%)	-
CC15	CC15–MSSA	-	3 (3.5%)	-
CC22	CC22–MRSA–IV (PVL+/tst+)	SCC*mec* IVa	1 (1.2%)	-
CC30	CC30–MSSA	-	1 (1.2%)	-
CC45	CC45–MSSA	-	2 (2.4%)	2 (12.5%)
CC59	CC59–MSSA	-	1 (1.2%)	-
CC72	ST72–MSSA	-	2 (2.4%)	2 (12.5%)
	ST72–MRSA–[*mec*VT + fus]	SCC*mec* VT + fus	1 (1.2%)	-
CC88	CC88–MRSA IV	SCC*mec* IVa	1 (1.2%)	-
CC97	CC97–MSSA	-	9 (10.6%)	1 (6.3%)
CC101	CC101–MSSA	-	1 (1.2%)	1 (6.3%)
CC121	CC121–MSSA	-	1 (1.2%)	-
CC152	CC152–MSSA	-	1 (1.2%)	-
	CC152–MSSA (PVL+)	-	13 (15.3%)	2 (12.5%)
CC188	CC188–MSSA	-	-	1 (6.3%)
CC239	CC239–MRSA–III, atypical “Southeast Asian Clade” *	Composite SCC*mec* III *	5 (5.9%)	-
	CC239–MRSA–[*mec* III + Cd/Hg + *ccrC*], “Southeast Asian Clade”	SCC [*mec* III + Cd/Hg + ccrC] (Bmb9393)	2 (2.4%)	-
CC398	CC398–MSSA	-	5 (5.9%)	-
*S. argenteus* CC2596	CC2596–MSSarg	-	2 (2.4%)	-

* See separate paragraph and Table 6 for further explanation.

## Data Availability

All relevant data are provided as Supplementary Files. The sequences of the genomes discussed, including the SCC*mec* element discussed, can be accessed under BioProject accession number PRJNA978032, and GenBank accession numbers CP127017 to CP127027.

## Supplementary Materials

The following supporting information can be downloaded at: https://www.mdpi.com/article/10.3390/antibiotics12061050/s1, Supplemental File S1: Hybridisation profiles and CC/strain affiliations of tested isolates (pdf). Supplemental File S2: Genome sequences of isolates 2020-042_7641M (CC8–MSSA, PVL-non-producer), 2020-043_7352M (CC8–MSSA, PVL-producer, as control), 2020-009_371M (CC239–MRSA–III var. with partially flipped/inverted SCC*mec*), 2020-021_7037M (CC239–MRSA–III var.) and 2020-048_8421A (CC239–MRSA–III var.). Supplemental File S3: Alignment of *hld* and the *agr* genes of the CC8 reference sequence NCTC8325 CP000253.1, the PVL-non-producer 2020-042_7641M (TT-042), and the PVL-producing isolate 2020-043_7352M (TT-043). The red arrow indicates a deletion in *agrC*-I of 2020-042_7641M (TT-042).

## References

[1] Köck R., Becker K., Cookson B., van Gemert-Pijnen J.E., Harbarth S., Kluytmans J., Mielke M., Peters G., Skov R.L., Struelens M.J. (2010). Methicillin-resistant *Staphylococcus aureus* (MRSA): Burden of disease and control challenges in Europe. Eurosurveillance.

[2] Monecke S., Coombs G., Shore A.C., Coleman D.C., Akpaka P., Borg M., Chow H., Ip M., Jatzwauk L., Jonas D. (2011). A field guide to pandemic, epidemic and sporadic clones of methicillin-resistant *Staphylococcus aureus*. PLoS ONE.

[3] Mediavilla J.R., Chen L., Mathema B., Kreiswirth B.N. (2012). Global epidemiology of community-associated methicillin resistant *Staphylococcus aureus* (CA-MRSA). Curr. Opin. Microbiol..

[4] Holden M.T.G., Hsu L.-Y., Kurt K., Weinert L.A., Mather A.E., Harris S.R., Strommenger B., Layer F., Witte W., de Lencastre H. (2013). A genomic portrait of the emergence, evolution, and global spread of a methicillin-resistant *Staphylococcus aureus* pandemic. Genome Res..

[5] Otto M. (2013). Community-associated MRSA: What makes them special?. Int. J. Med. Microbiol..

[6] Tacconelli E., Carrara E., Savoldi A., Harbarth S., Mendelson M., Monnet D.L., Pulcini C., Kahlmeter G., Kluytmans J., Carmeli Y. (2018). Discovery, research, and development of new antibiotics: The WHO priority list of antibiotic-resistant bacteria and tuberculosis. Lancet Infect. Dis..

[7] De Kraker M.E.A., Stewardson A.J., Harbarth S. (2016). Will 10 Million People Die a Year due to Antimicrobial Resistance by 2050?. PLoS Medicine.

[8] Enright M.C., Day N.P., Davies C.E., Peacock S.J., Spratt B.G. (2000). Multilocus sequence typing for characterization of methicillin-resistant and methicillin-susceptible clones of *Staphylococcus aureus*. J. Clin. Microbiol..

[9] Ito T., Katayama Y., Asada K., Mori N., Tsutsumimoto K., Tiensasitorn C., Hiramatsu K. (2001). Structural comparison of three types of staphylococcal cassette chromosome *mec* integrated in the chromosome in methicillin-resistant *Staphylococcus aureus*. Antimicrob. Agents Chemother..

[10] Ito T., Ma X.X., Takeuchi F., Okuma K., Yuzawa H., Hiramatsu K. (2004). Novel type V staphylococcal cassette chromosome *mec* driven by a novel cassette chromosome recombinase, ccrC. Antimicrob. Agents Chemother..

[11] Ubukata K., Nonoguchi R., Matsuhashi M., Konno M. (1989). Expression and inducibility in *Staphylococcus aureus* of the mecA gene, which encodes a methicillin-resistant *S. aureus*-specific penicillin-binding protein. J. Bacteriol..

[12] Boyle-Vavra S., Daum R.S. (2007). Community-acquired methicillin-resistant *Staphylococcus aureus*: The role of Panton-Valentine leukocidin. Lab. Invest..

[13] Buescher E.S. (2005). Community-acquired methicillin-resistant *Staphylococcus aureus* in pediatrics. Curr. Opin. Pediatr..

[14] Bukharie H.A. (2010). Increasing threat of community-acquired methicillin-resistant *Staphylococcus aureus*. Am. J. Med. Sci..

[15] Coombs G.W., Monecke S., Pearson J.C., Tan H.L., Chew Y.K., Wilson L., Ehricht R., O’Brien F.G., Christiansen K.J. (2011). Evolution and diversity of community-associated methicillin-resistant *Staphylococcus aureus* in a geographical region. BMC Microbiol..

[16] Said-Salim B., Mathema B., Kreiswirth B.N. (2003). Community-acquired methicillin-resistant *Staphylococcus aureus*: An emerging pathogen. Infect. Control Hosp. Epidemiol..

[17] Vandenesch F., Naimi T., Enright M.C., Lina G., Nimmo G.R., Heffernan H., Liassine N., Bes M., Greenland T., Reverdy M.E. (2003). Community-acquired methicillin-resistant *Staphylococcus aureus* carrying Panton-Valentine leukocidin genes: Worldwide emergence. Emerg. Infect. Dis..

[18] Alioua M.A., Labid A., Amoura K., Bertine M., Gacemi-Kirane D., Dekhil M. (2014). Emergence of the European ST80 clone of community-associated methicillin-resistant *Staphylococcus aureus* as a cause of healthcare-associated infections in Eastern Algeria. Med. Mal. Infect..

[19] Naas T., Fortineau N., Spicq C., Robert J., Jarlier V., Nordmann P. (2005). Three-year survey of community-acquired methicillin-resistant *Staphylococcus aureus* producing Panton-Valentine leukocidin in a French university hospital. J. Hosp. Infect..

[20] Takano T., Saito K., Teng L.J., Yamamoto T. (2007). Spread of community-acquired methicillin-resistant *Staphylococcus aureus* (MRSA) in hospitals in Taipei, Taiwan in 2005, and comparison of its drug resistance with previous hospital-acquired MRSA. Microbiol. Immunol..

[21] Kourbatova E.V., Halvosa J.S., King M.D., Ray S.M., White N., Blumberg H.M. (2005). Emergence of community-associated methicillin-resistant *Staphylococcus aureus* USA 300 clone as a cause of health care-associated infections among patients with prosthetic joint infections. Am. J. Infect. Control.

[22] Seybold U., Kourbatova E.V., Johnson J.G., Halvosa S.J., Wang Y.F., King M.D., Ray S.M., Blumberg H.M. (2006). Emergence of community-associated methicillin-resistant *Staphylococcus aureus* USA300 genotype as a major cause of health care-associated blood stream infections. Clin. Infect. Dis..

[23] Berglund C., Molling P., Sjoberg L., Soderquist B. (2005). Predominance of staphylococcal cassette chromosome *mec* (SCC*mec*) type IV among methicillin-resistant *Staphylococcus aureus* (MRSA) in a Swedish county and presence of unknown SCC*mec* types with Panton-Valentine leukocidin genes. Clin. Microbiol. Infect..

[24] Bonnstetter K.K., Wolter D.J., Tenover F.C., McDougal L.K., Goering R.V. (2007). Rapid multiplex PCR assay for identification of USA300 community-associated methicillin-resistant *Staphylococcus aureus* isolates. J. Clin. Microbiol..

[25] Ellington M.J., Perry C., Ganner M., Warner M., McCormick Smith I., Hill R.L., Shallcross L., Sabersheikh S., Holmes A., Cookson B.D. (2009). Clinical and molecular epidemiology of ciprofloxacin-susceptible MRSA encoding PVL in England and Wales. Eur. J. Clin. Microbiol. Infect. Dis..

[26] Tristan A., Bes M., Meugnier H., Lina G., Bozdogan B., Courvalin P., Reverdy M.E., Enright M.C., Vandenesch F., Etienne J. (2007). Global distribution of Panton-Valentine leukocidin--positive methicillin-resistant *Staphylococcus aureus*, 2006. Emerg. Infect. Dis..

[27] Kaneko J., Kamio Y. (2004). Bacterial two-component and hetero-heptameric pore-forming cytolytic toxins: Structures, pore-forming mechanism, and organization of the genes. Biosci. Biotechnol. Biochem..

[28] Kaneko J., Kimura T., Kawakami Y., Tomita T., Kamio Y. (1997). Panton-valentine leukocidin genes in a phage-like particle isolated from mitomycin C-treated *Staphylococcus aureus* V8 (ATCC 49775). Biosci. Biotechnol. Biochem..

[29] Kaneko J., Kimura T., Narita S., Tomita T., Kamio Y. (1998). Complete nucleotide sequence and molecular characterization of the temperate staphylococcal bacteriophage phiPVL carrying Panton-Valentine leukocidin genes. Gene.

[30] Zou D., Kaneko J., Narita S., Kamio Y. (2000). Prophage, phiPV83-pro, carrying panton-valentine leukocidin genes, on the *Staphylococcus aureus* P83 chromosome: Comparative analysis of the genome structures of phiPV83-pro, phiPVL, phi11, and other phages. Biosci. Biotechnol. Biochem..

[31] Chroboczek T., Boisset S., Rasigade J.P., Meugnier H., Akpaka P.E., Nicholson A., Nicolas M., Olive C., Bes M., Vandenesch F. (2013). Major West Indies MRSA clones in human beings: Do they travel with their hosts?. J. Travel. Med..

[32] Uhlemann A.C., Dumortier C., Hafer C., Taylor B.S., Sánchez J., Rodriguez-Taveras C., Leon P., Rojas R., Olive C., Lowy F.D. (2012). Molecular characterization of *Staphylococcus aureus* from outpatients in the Caribbean reveals the presence of pandemic clones. Eur. J. Clin. Microbiol. Infect. Dis..

[33] Guardabassi L., Moodley A., Williams A., Stegger M., Damborg P., Halliday-Simmonds I., Butaye P. (2019). High Prevalence of USA300 Among Clinical Isolates of Methicillin-Resistant *Staphylococcus aureus* on St. Kitts and Nevis, West Indies. Front. Microbiol..

[34] Hopman J., Peraza G.T., Espinosa F., Klaassen C.H., Velázquez D.M., Meis J.F., Voss A. (2012). USA300 Methicillin-resistant *Staphylococcus aureus* in Cuba. Antimicrob. Resist. Infect. Control.

[35] Leiva Peláez O., Stojanov M., Zayas Tamayo A.M., Barreras García G., González Aleman M., Martínez Ceballos L., Muñoz del Campo J.L., Bello Rodríguez O., Gonzalez Mesa L., Blanc D.S. (2015). Molecular epidemiology of methicillin-resistant *Staphylococcus aureus* from 4 Cuban hospitals. Diagn. Microbiol. Infect. Dis..

[36] Baez M., Collaud A., Espinosa I., Perreten V. (2017). MRSA USA300, USA300-LV and ST5-IV in pigs, Cuba. Int. J. Antimicrob. Agents.

[37] Gittens-St Hilaire M.V., Chase E., Alleyne D. (2020). Prevalence, molecular characteristics and antimicrobial susceptibility patterns of MRSA in hospitalized and nonhospitalized patients in Barbados. New Microbes New Infect..

[38] Rosenthal M.E., Mediavilla J., Chen L., Sonnenfeld J., Pierce L., Shannon A., Boucher H., Pearlmutter M., Kreiswirth B., Kuo Y.-H. (2014). Molecular epidemiology of *Staphylococcus aureus* in post-earthquake northern Haiti. Int. J. Infect. Dis..

[39] Monecke S., Stieber B., Roberts R., Akpaka P.E., Slickers P., Ehricht R. (2014). Population structure of *Staphylococcus aureus* from Trinidad & Tobago. PLoS ONE.

[40] Akpaka P.E., Monecke S., Swanston W.H., Rao A.C., Schulz R., Levett P.N. (2011). Methicillin sensitive *Staphylococcus aureus* producing Panton-Valentine leukocidin toxin in Trinidad & Tobago: A case report. J. Med. Case Rep..

[41] Monecke S., Nitschke H., Slickers P., Ehricht R., Swanston W., Manjunath M., Roberts R., Akpaka P.E. (2012). Molecular epidemiology and characterisation of MRSA isolates from Trinidad and Tobago. Eur. J. Clin. Microbiol. Infect. Dis..

[42] Akpaka P.E., Kissoon S., Rutherford C., Swanston W.H., Jayaratne P. (2007). Molecular epidemiology of methicillin-resistant *Staphylococcus aureus* isolates from regional hospitals in Trinidad and Tobago. Int. J. Infect. Dis..

[43] Orrett F.A., Land M. (2006). Methicillin-resistant *Staphylococcus aureus* prevalence: Current susceptibility patterns in Trinidad. BMC Infect. Dis..

[44] Brown P.D., Ngeno C. (2007). Antimicrobial resistance in clinical isolates of *Staphylococcus aureus* from hospital and community sources in southern Jamaica. Int. J. Infect. Dis..

[45] Nicholson A.M., Thorns C., Wint H., Didier M., Willis R., McMorris N., Castle D., Maharaj N., Orrett F.A. (2010). The detection of mupirocin resistance and the distribution of methicillin-resistant *Staphylococcus aureus* at the University Hospital of the West Indies, Jamaica. West Indian Med. J..

[46] Swanston W.H. (1999). Methicillin resistant *Staphylococcus aureus*. West Indian Med. J..

[47] Akpaka P.E., Kissoon S., Swanston W.H., Monteil M. (2006). Prevalence and antimicrobial susceptibility pattern of methicillin resistant *Staphylococcus aureus* isolates from Trinidad & Tobago. Ann. Clin. Microbiol. Antimicrob..

[48] Bodonaik N.C., Nicholson A. (2002). Methicillin resistance in Strains of *Staphylococcus aureus* at the University Hospital of the West Indies, Jamaica, 1980–1997. Int. Sci. Exc..

[49] Monecke S., Jatzwauk L., Müller E., Nitschke H., Pfohl K., Slickers P., Reissig A., Ruppelt-Lorz A., Ehricht R. (2016). Diversity of SCC*mec* elements in *Staphylococcus aureus* as observed in South-Eastern Germany. PLoS ONE.

[50] Cavaco L.M., Hasman H., Stegger M., Andersen P.S., Skov R., Fluit A.C., Ito T., Aarestrup F.M. (2010). Cloning and Occurrence of *czrC*, a Gene Conferring Cadmium and Zinc Resistance in Methicillin-Resistant *Staphylococcus aureus* CC398 Isolates. Antimicrob. Agents Chemother..

[51] Li M., Du X., Villaruz A.E., Diep B.A., Wang D., Song Y., Tian Y., Hu J., Yu F., Lu Y. (2012). MRSA epidemic linked to a quickly spreading colonization and virulence determinant. Nat. Med..

[52] Holden M.T.G., Lindsay J.A., Corton C., Quail M.A., Cockfield J.D., Pathak S., Batra R., Parkhill J., Bentley S.D., Edgeworth J.D. (2010). Genome Sequence of a Recently Emerged, Highly Transmissible, Multi-Antibiotic- and Antiseptic-Resistant Variant of Methicillin-Resistant *Staphylococcus aureus*, Sequence Type 239 (TW). J. Bacteriol..

[53] Orrett F.A. (1999). Methicillin resistance among Trinidadian isolates of community and hospital strains of *Staphylococcus aureus* and their patterns of resistance to non-beta-lactam antibiotics. Jpn. J. Infect. Dis..

[54] Akpaka P., Roberts R., Monecke S. (2015). Molecular Analysis of *Staphylococcus aureus* Infections in Trinidad and Tobago. Br. Microbiol. Res. J..

[55] Ramdass M., Balliram S., Cadan A., Bhaggan N., Mohammed B., Singh R., Maharaj J., Boodram A. (2018). Prevalence of methicillin-resistant *Staphylococcus aureus* in the surgical wards of the Port-of-Spain General Hospital, Trinidad and Tobago. West Indian Med. J..

[56] Akpaka P.E., Roberts R., Monecke S. (2017). Molecular characterization of antimicrobial resistance genes against *Staphylococcus aureus* isolates from Trinidad and Tobago. J. Infect. Public Health.

[57] Vire F.P., Akpaka E.P., Unakal C. (2018). Prevalent virulent genes among methicillin resistant *Staphylococcus aureus* isolates from community settings in Trinidad and Tobago. Int. J. Infect. Dis..

[58] Smyth D.S., McDougal L.K., Gran F.W., Manoharan A., Enright M.C., Song J.H., de Lencastre H., Robinson D.A. (2010). Population structure of a hybrid clonal group of methicillin-resistant *Staphylococcus aureus*, ST239-MRSA-III. PLoS ONE.

[59] Monecke S., Slickers P., Gawlik D., Müller E., Reissig A., Ruppelt-Lorz A., Akpaka P., Bandt D., Bes M., Boswihi S. (2018). Molecular typing of ST239-MRSA-III from diverse geographic locations and the evolution of the SCC*mec* III element during its intercontinental spread. Front. Microbiol..

[60] Shore A., Rossney A.S., Keane C.T., Enright M.C., Coleman D.C. (2005). Seven novel variants of the staphylococcal chromosomal cassette *mec* in methicillin-resistant *Staphylococcus aureus* isolates from Ireland. Antimicrob. Agents Chemother..

[61] Larner-Svensson H., Worning P., Bartels M.D., Hestbjerg Hansen L., Boye K., Westh H. (2013). Complete Genome Sequence of *Staphylococcus aureus* Strain M1, a Unique t024-ST8-IVa Danish Methicillin-Resistant *S. aureus* Clone. Genome Announc..

[62] Hau S.J., Bayles D.O., Alt D.P., Nicholson T.L. (2017). Draft Genome Sequences of 14 *Staphylococcus aureus* Sequence Type 5 Isolates from California, USA. Genome Announc..

[63] Zhang Y.Q., Ren S.X., Li H.L., Wang Y.X., Fu G., Yang J., Qin Z.Q., Miao Y.G., Wang W.Y., Chen R.S. (2003). Genome-based analysis of virulence genes in a non-biofilm-forming *Staphylococcus epidermidis* strain (ATCC 12228). Mol. Microbiol..

[64] Bowman F.W. (1957). Test organisms for antibiotic microbial assays. Antibiot Chemother.

[65] Hugh R., Ellis M.A. (1968). The neotype strain for *Staphylococcus epidermidis* (Winslow and Winslow 1908) Evans 1916. Int. J. Syst. Evol. Microbiol..

[66] Monecke S., Slickers P., Gawlik D., Müller E., Reissig A., Ruppelt-Lorz A., de Jäckel S.C., Feßler A.T., Frank M., Hotzel H. (2018). Variability of SCC*mec* elements in livestock-associated CC398 MRSA. Vet. Microbiol..

[67] Aung M.S., Urushibara N., Kawaguchiya M., Hirose M., Ito M., Habadera S., Kobayashi N. (2021). Clonal diversity of methicillin-resistant *Staphylococcus aureus* (MRSA) from bloodstream infections in northern Japan: Identification of spermidine N-acetyltransferase gene (*speG*) in staphylococcal cassette chromosomes (SCCs) associated with type II and IV SCCmec. J. Glob. Antimicrob. Resist..

[68] Joshi G.S., Spontak J.S., Klapper D.G., Richardson A.R. (2011). Arginine catabolic mobile element encoded *speG* abrogates the unique hypersensitivity of *Staphylococcus aureus* to exogenous polyamines. Mol. Microbiol..

[69] Arias C.A., Rincon S., Chowdhury S., Martinez E., Coronell W., Reyes J., Nallapareddy S.R., Murray B.E. (2008). MRSA USA300 clone and VREF--a U.S.-Colombian connection?. N. Engl. J. Med..

[70] Planet P.J., Diaz L., Rios R., Arias C.A. (2016). Global Spread of the Community-Associated Methicillin-Resistant *Staphylococcus aureus* USA300 Latin American Variant. J. Infect. Dis..

[71] Earls M.R., Coleman D.C., Brennan G.I., Fleming T., Monecke S., Slickers P., Ehricht R., Shore A.C. (2018). Intra-Hospital, Inter-Hospital and Intercontinental Spread of ST78 MRSA From Two Neonatal Intensive Care Unit Outbreaks Established Using Whole-Genome Sequencing. Front. Microbiol..

[72] Roberts M.C., Joshi P.R., Greninger A.L., Melendez D., Paudel S., Acharya M., Bimali N.K., Koju N.P., No D., Chalise M. (2018). The human clone ST22 SCC*mec* IV methicillin-resistant *Staphylococcus aureus* isolated from swine herds and wild primates in Nepal: Is man the common source?. FEMS Microbiol. Ecol..

[73] Roberts M.C., Joshi P.R., Monecke S., Ehricht R., Müller E., Gawlik D., Paudel S., Acharya M., Bhattarai S., Pokharel S. (2019). MRSA Strains in Nepalese Rhesus Macaques (*Macaca mulatta*) and Their Environment. Front. Microbiol..

[74] Monecke S., Syed M.A., Khan M.A., Ahmed S., Tabassum S., Gawlik D., Müller E., Reissig A., Braun S.D., Ehricht R. (2020). Genotyping of methicillin-resistant *Staphylococcus aureus* from sepsis patients in Pakistan and detection of antibodies against staphylococcal virulence factors. Eur. J. Clin. Microbiol. Infect. Dis..

[75] Boswihi S.S., Verghese T., Udo E.E. (2022). Diversity of clonal complex 22 methicillin-resistant *Staphylococcus aureus* isolates in Kuwait hospitals. Front. Microbiol..

[76] Senok A., Nassar R., Celiloglu H., Nabi A., Alfaresi M., Weber S., Rizvi I., Müller E., Reissig A., Gawlik D. (2020). Genotyping of methicillin resistant *Staphylococcus aureus* from the United Arab Emirates. Sci. Rep..

[77] Monecke S., Müller E., Buechler J., Rejman J., Stieber B., Akpaka P.E., Bandt D., Burris R., Coombs G., Hidalgo-Arroyo G.A. (2013). Rapid detection of Panton-Valentine leukocidin in *Staphylococcus aureus* cultures by use of a lateral flow assay based on monoclonal antibodies. J. Clin. Microbiol..

[78] Breurec S., Fall C., Pouillot R., Boisier P., Brisse S., Diene-Sarr F., Djibo S., Etienne J., Fonkoua M.C., Perrier-Gros-Claude J.D. (2011). Epidemiology of methicillin-susceptible *Staphylococcus aureus* lineages in five major African towns: High prevalence of Panton-Valentine leukocidin genes. Clin. Microbiol. Infect..

[79] Egyir B., Guardabassi L., Esson J., Nielsen S.S., Newman M.J., Addo K.K., Larsen A.R. (2014). Insights into nasal carriage of *Staphylococcus aureus* in an urban and a rural community in Ghana. PLoS ONE.

[80] Egyir B., Guardabassi L., Sorum M., Nielsen S.S., Kolekang A., Frimpong E., Addo K.K., Newman M.J., Larsen A.R. (2014). Molecular epidemiology and antimicrobial susceptibility of clinical *Staphylococcus aureus* from healthcare institutions in Ghana. PLoS ONE.

[81] Okuda K.V., Toepfner N., Alabi A.S., Arnold B., Belard S., Falke U., Menschner L., Monecke S., Ruppelt-Lorz A., Berner R. (2016). Molecular epidemiology of *Staphylococcus aureus* from Lambarene, Gabon. Eur. J. Clin. Microbiol. Infect. Dis..

[82] Shittu A.O., Oyedara O., Kenneth O.O., Raji A., Peters G., von Müller L., Schaumburg F., Herrmann M., Ruffing U. (2015). An assessment on DNA microarray and sequence-based methods for the characterization of methicillin-susceptible *Staphylococcus aureus* from Nigeria. Front. Microbiol..

[83] Holt D.C., Holden M.T.G., Tong S.Y.C., Castillo-Ramirez S., Clarke L., Quail M.A., Currie B.J., Parkhill J., Bentley S.D., Feil E.J. (2011). A very early-branching *Staphylococcus aureus* lineage lacking the carotenoid pigment staphyloxanthin. Genome Biol. Evol..

[84] Chen S.Y., Lee H., Wang X.M., Lee T.F., Liao C.H., Teng L.J., Hsueh P.R. (2018). High mortality impact of *Staphylococcus argenteus* on patients with community-onset staphylococcal bacteraemia. Int. J. Antimicrob. Agents.

[85] Johansson C., Rautelin H., Kaden R. (2019). *Staphylococcus argenteus* and *Staphylococcus schweitzeri* are cytotoxic to human cells in vitro due to high expression of alpha-hemolysin Hla. Virulence.

[86] Becker K., Schaumburg F., Kearns A., Larsen A.R., Lindsay J.A., Skov R.L., Westh H. (2019). Implications of identifying the recently defined members of the *Staphylococcus aureus* complex *S. argenteus* and *S. schweitzeri*: A position paper of members of the ESCMID Study Group for Staphylococci and Staphylococcal Diseases (ESGS). Clin. Microbiol. Infect..

[87] Dupieux C., Blondé R., Bouchiat C., Meugnier H., Bes M., Laurent S., Vandenesch F., Laurent F., Tristan A. (2015). Community-acquired infections due to *Staphylococcus argenteus* lineage isolates harbouring the Panton-Valentine leucocidin, France, 2014. Eurosurveillance.

[88] McDonald M., Dougall A., Holt D., Huygens F., Oppedisano F., Giffard P.M., Inman-Bamber J., Stephens A.J., Towers R., Carapetis J.R. (2006). Use of a single-nucleotide polymorphism genotyping system to demonstrate the unique epidemiology of methicillin-resistant *Staphylococcus aureus* in remote aboriginal communities. J. Clin. Microbiol..

[89] Tång Hallbäck E., Karami N., Adlerberth I., Cardew S., Ohlén M., Engström Jakobsson H., Svensson Stadler L. (2018). Methicillin-resistant *Staphylococcus argenteus* misidentified as methicillin-resistant *Staphylococcus aureus* emerging in western Sweden. J. Med. Microbiol..

[90] Monecke S., Slickers P., Ehricht R. (2008). Assignment of *Staphylococcus aureus* isolates to clonal complexes based on microarray analysis and pattern recognition. FEMS Immunol. Med. Microbiol..

[91] Senok A., Monecke S., Nassar R., Celiloglu H., Thyagarajan S., Müller E., Ehricht R. (2021). Lateral Flow Immunoassay for the Detection of Panton-Valentine Leukocidin in *Staphylococcus aureus* from skin and soft tissue infections in the United Arab Emirates. Front. Microbiol..

