# Supplementary material for: Clonal Complexes Distribution of Staphylococcus aureus Isolates from Clinical Samples from the Caribbean Islands
Source: Antibiotics (Basel). 2023 Jun 14;12(6):1050. doi: 10.3390/antibiotics12061050 (PMC10295549; doi:10.3390/antibiotics12061050)
Supplement: Supplementary file 1 [file antibiotics-12-01050-s001.zip › Supplemental File S1_Array Hybridisation Profiles_2023-05-10.pdf]

STRAIN / ISOLATE	BACKGROUND INFO			TYPING DATA		SPECIES MARKER							REGULATORY GENES																						
																agrI				agrII				agrIII				agrIV			
	HOST SPECIES	SAMPLE TYPE/DIAGNOSIS	PATIENT	CC		rrnD1	gapA	katA	CoA	nuc1	spa	sbi	sarA	saeS	vraS	agrI (total)	agrB-I	agrC-I	agrD-I	agrII (total)	agrB-II	agrC-II	agrD-II	agrIII (total)	agrB-III	agrC-III	agrD-III	agrIV (total)	agrB-IV	agrC-IV	hld
						Domain 1 of 23S-rRNA	glyceraldehyde 3-phosphate dehydrogenase, locus 1	katalase A	coagulase	thermostable extracellular nuclease	staphylococcal protein A	IgG-binding protein	staphylo-coccal accessory regulator A	histidine protein kinase, sae locus	sensor protein	accessory gene regulator allele I				accessory gene regulator allele II				accessory gene regulator allele III				accessory gene regulator allele IV			haemolysin delta
>CC1																															
Jamaica_2020-064_JM03	Human			CC1	CC1-MSSA	POS	POS	POS	POS	POS	POS	POS	POS	POS	POS	NEG	NEG	NEG	NEG	NEG	NEG	NEG	NEG	POS	POS	POS	POS	NEG	NEG	NEG	POS
Jamaica_2020-073_JM12	Human			CC1	CC1-MSSA	POS	POS	POS	POS	POS	POS	POS	POS	POS	POS	NEG	NEG	NEG	NEG	NEG	NEG	NEG	NEG	POS	POS	POS	POS	NEG	NEG	NEG	POS
Jamaica_2020-081_JM15	Human			CC1	CC1-MSSA	POS	POS	POS	POS	POS	POS	POS	POS	POS	POS	NEG	NEG	NEG	NEG	NEG	NEG	NEG	NEG	POS	POS	POS	POS	NEG	NEG	NEG	POS
Jamaica_2020-082_JM16	Human			CC1	CC1-MSSA	POS	POS	POS	POS	POS	POS	POS	POS	POS	POS	NEG	NEG	NEG	NEG	NEG	NEG	NEG	NEG	POS	POS	POS	POS	NEG	NEG	NEG	POS
Trinidad&Tobago_2020-099_59032	Human	Wound Swab: Cellulitis	male, born in 1965	CC1	CC1-MSSA	POS	POS	POS	NEG	POS	POS	POS	POS	POS	POS	NEG	NEG	NEG	NEG	NEG	NEG	NEG	NEG	POS	POS	NEG	POS	NEG	NEG	NEG	POS
>CC5																															
Trinidad&Tobago_2020-103_54985	Human			CC5	CC5-MSSA	POS	POS	POS	POS	POS	POS	POS	POS	POS	POS	NEG	NEG	NEG	NEG	POS	POS	POS	POS	NEG	NEG	NEG	NEG	NEG	NEG	NEG	POS
Trinidad&Tobago_2020-016_6988M	Human	Pus: Nasal Vestibular abscess/Nasal abscess	male, born in 1983	CC5	CC5-MSSA (PVL+)	POS	POS	POS	POS	POS	POS	POS	POS	POS	POS	NEG	NEG	NEG	NEG	POS	POS	POS	POS	NEG	NEG	NEG	NEG	NEG	NEG	NEG	POS
Trinidad&Tobago_2020-086_55562	Human	Wound Swab: Recurrent ear infection	male, born in 1986	CC5	CC5-MSSA (PVL+)	POS	POS	POS	POS	POS	POS	POS	POS	POS	POS	NEG	NEG	NEG	NEG	POS	POS	POS	POS	NEG	NEG	NEG	NEG	NEG	NEG	NEG	POS
>CC6																															
Jamaica_2020-062_JM01	Human	Throat swab		CC6	CC6-MSSA	POS	POS	POS	POS	POS	POS	POS	POS	POS	POS	POS	POS	POS	POS	NEG	NEG	NEG	NEG	NEG	NEG	NEG	NEG	AMB	POS	NEG	POS
Trinidad&Tobago_2020-031_7269M	Human	Wound Swab: Facial abscess/swab from face	male, born in 1984	CC6	CC6-MSSA	POS	POS	POS	POS	POS	POS	POS	POS	POS	POS	POS	POS	POS	POS	NEG	NEG	NEG	NEG	NEG	NEG	NEG	NEG	NEG	AMB	NEG	POS
Trinidad&Tobago_2020-052_8515B	Human	BLOOD: Sepsis	male, born in 2004	CC6	CC6-MSSA	POS	POS	POS	POS	POS	POS	POS	POS	POS	POS	POS	POS	POS	POS	NEG	NEG	NEG	NEG	NEG	NEG	NEG	NEG	NEG	POS	NEG	POS
Trinidad&Tobago_2020-105_6958M	Human	Wound Swab: Cellulitis/Left hand wound	female, born in 1951	CC6	CC6-MSSA	POS	POS	POS	POS	POS	POS	POS	POS	POS	POS	POS	POS	POS	POS	NEG	NEG	NEG	NEG	NEG	NEG	NEG	NEG	AMB	POS	NEG	POS
Trinidad&Tobago_2020-106_6959M	Human	Wound Swab: Skin abscesss	male, born in 1985	CC6	CC6-MSSA	POS	POS	POS	POS	POS	POS	POS	POS	POS	POS	POS	POS	POS	POS	NEG	NEG	NEG	NEG	NEG	NEG	NEG	NEG	NEG	NEG	NEG	POS
Jamaica_2020-065_JM04	Human	Blood		CC6	CC6-MSSA (PVL+)	POS	POS	POS	POS	POS	POS	POS	POS	POS	POS	POS	POS	POS	POS	NEG	NEG	NEG	NEG	NEG	NEG	NEG	NEG	NEG	POS	NEG	POS
Jamaica_2020-070_JM09	Human			CC6	CC6-MSSA (PVL+)	POS	POS	POS	POS	POS	POS	POS	POS	POS	POS	POS	POS	POS	POS	NEG	NEG	NEG	NEG	NEG	NEG	NEG	NEG	NEG	POS	NEG	POS
Trinidad&Tobago_2020-015_6960M	Human	Wound Swab: Fournier's gangrene/Ca Prostate	male, born in 1938	CC6	CC6-MSSA (PVL+)	POS	POS	POS	AMB	POS	POS	POS	POS	POS	POS	POS	POS	POS	POS	NEG	NEG	NEG	NEG	NEG	NEG	NEG	NEG	NEG	AMB	NEG	POS
Trinidad&Tobago_2020-028_7195M	Human	Wound Swab: Reccurent axillary abscess/suppurat. hydranitis	male, born in 1984	CC6	CC6-MSSA (PVL+)	POS	POS	POS	POS	POS	POS	POS	POS	POS	POS	POS	POS	POS	POS	NEG	NEG	NEG	NEG	NEG	NEG	NEG	NEG	NEG	POS	NEG	POS
Trinidad&Tobago_2020-034_7255M	Human	Wound Swab: Skin abscess/abscess under right arm	male, born in 2006	CC6	CC6-MSSA (PVL+)	POS	POS	POS	POS	POS	POS	POS	POS	POS	POS	POS	POS	POS	POS	NEG	NEG	NEG	NEG	NEG	NEG	NEG	NEG	NEG	POS	NEG	POS
Trinidad&Tobago_2020-087_56696	Human	Urine, Pregnant	female, born in 1987	CC6	CC6-MSSA (PVL+)	POS	POS	POS	POS	POS	POS	POS	POS	POS	POS	POS	POS	NEG	POS	NEG	NEG	NEG	NEG	NEG	NEG	NEG	NEG	NEG	NEG	NEG	POS
>CC7																															
Trinidad&Tobago_2020-003_4216M	Human	Wound Swab: Infected laceration/Chop wound to left foot	male, born in 1967	CC7	CC7-MSSA	POS	POS	POS	POS	POS	POS	POS	POS	POS	POS	POS	POS	POS	POS	NEG	NEG	NEG	NEG	NEG	NEG	NEG	NEG	NEG	POS	NEG	POS
Trinidad&Tobago_2020-019_7024M	Human	Wound Swab: Surgical site/post surgical Infection	female, born in 1966	CC7	CC7-MSSA	POS	POS	POS	POS	POS	POS	POS	POS	POS	POS	POS	POS	POS	POS	NEG	NEG	NEG	NEG	NEG	NEG	NEG	NEG	AMB	POS	NEG	POS
Trinidad&Tobago_2020-027_7194M	Human	Wound Swab: Surgical site Infections/	male, born in 1987	CC7	CC7-MSSA	POS	POS	POS	POS	POS	POS	POS	POS	POS	POS	POS	POS	POS	POS	NEG	NEG	NEG	NEG	NEG	NEG	NEG	NEG	NEG	POS	NEG	POS
Trinidad&Tobago_2020-030_7403M	Human	Wound Swab: Surgical site Infections/	male, born in 1938	CC7	CC7-MSSA	POS	POS	POS	POS	POS	POS	POS	POS	POS	POS	POS	POS	POS	POS	NEG	NEG	NEG	NEG	NEG	NEG	NEG	NEG	NEG	POS	NEG	POS
Trinidad&Tobago_2020-055_8942U	Human	Urine: UTI	female, born in 1987	CC7	CC7-MSSA	POS	POS	POS	POS	POS	POS	POS	POS	POS	POS	POS	POS	POS	POS	NEG	NEG	NEG	NEG	NEG	NEG	NEG	NEG	NEG	POS	NEG	POS
>CC8																															
Trinidad&Tobago_2020-049_8398A	Human	BLOOD: Sepsis/Generalized weakness, fever	male, born in 1946	CC8	CC8-MSSA	POS	POS	POS	POS	POS	POS	POS	POS	POS	POS	POS	POS	POS	POS	NEG	NEG	NEG	NEG	NEG	NEG	NEG	NEG	NEG	POS	NEG	POS
Trinidad&Tobago_2020-077_8398B	Human	Peritoneal swab: surgical site Infections, abdominal pain, fever, vomiting	female, born in 1951	CC8	CC8-MSSA	POS	POS	POS	POS	POS	POS	POS	POS	POS	POS	POS	POS	NEG	POS	NEG	NEG	NEG	NEG	NEG	NEG	NEG	NEG	NEG	NEG	NEG	POS
Trinidad&Tobago_2020-002_4209M	Human	Wound Swab: Labial abscess/Skin abscess	Four months old baby	CC8	CC8-MSSA (PVL+)	POS	POS	POS	POS	POS	POS	POS	POS	POS	POS	POS	POS	POS	POS	NEG	NEG	NEG	NEG	NEG	NEG	NEG	NEG	NEG	AMB	NEG	POS
Trinidad&Tobago_2020-011_4173M	Human	Wound Swab: Gluteal abscess/Right Gluteal abscess	female, born in 2018	CC8	CC8-MSSA (PVL+)	POS	POS	POS	POS	POS	POS	POS	POS	POS	POS	POS	POS	POS	POS	NEG	NEG	NEG	NEG	NEG	NEG	NEG	NEG	NEG	NEG	NEG	POS
Trinidad&Tobago_2020-013_6950M	Human	Wound Swab: Skin abscess/Abscess to right leg	male, born in 1975	CC8	CC8-MSSA (PVL+)	POS	POS	POS	POS	POS	POS	POS	POS	POS	POS	POS	POS	POS	POS	NEG	NEG	NEG	NEG	NEG	NEG	NEG	NEG	NEG	AMB	NEG	POS
Trinidad&Tobago_2020-014_7203M(1)	Human	Wound Swab: Appendicitis/inflammed appendix	male, born in 2009	CC8	CC8-MSSA (PVL+)	POS	POS	POS	POS	POS	POS	POS	POS	POS	POS	POS	POS	POS	POS	NEG	NEG	NEG	NEG	NEG	NEG	NEG	NEG	NEG	POS	NEG	POS
Trinidad&Tobago_2020-017_7015M	Human	Wound Swab: Skin abscess/Left thigh abscess	female, born in 2012	CC8	CC8-MSSA (PVL+)	POS	POS	POS	POS	POS	POS	POS	POS	POS	POS	POS	POS	POS	POS	NEG	NEG	NEG	NEG	NEG	NEG	NEG	NEG	NEG	POS	NEG	POS
Trinidad&Tobago_2020-035_7297M	Human	Eye swab: Cellulitis/? Sepsis	female, born in 2006	CC8	CC8-MSSA (PVL+)	POS	POS	POS	POS	POS	POS	POS	POS	POS	POS	POS	POS	POS	POS	NEG	NEG	NEG	NEG	NEG	NEG	NEG	NEG	NEG	AMB	NEG	POS
Trinidad&Tobago_2020-038_7309M	Human	Pus: Surgical site Infections/Laparatomy	male, born in 1989	CC8	CC8-MSSA (PVL+)	POS	POS	POS	POS	POS	POS	POS	POS	POS	POS	POS	POS	POS	POS	NEG	NEG	NEG	NEG	NEG	NEG	NEG	NEG	NEG	POS	NEG	POS
Trinidad&Tobago_2020-042_7641M	Human	Wound Swab: Surgical site Infections	male, born in 2006	CC8	CC8-MSSA (PVL+)	POS	POS	POS	POS	POS	POS	POS	POS	POS	POS	POS	POS	POS	POS	NEG	NEG	NEG	NEG	NEG	NEG	NEG	NEG	NEG	AMB	NEG	POS
Trinidad&Tobago_2020-043_7352M	Human	Wound Swab: Skin abscess/Right shoulder abscess	female, born in 2018	CC8	CC8-MSSA (PVL+)	POS	POS	POS	POS	POS	POS	POS	POS	POS	POS	POS	POS	POS	POS	NEG	NEG	NEG	NEG	NEG	NEG	NEG	NEG	NEG	POS	NEG	POS
Trinidad&Tobago_2020-057_8928P	Human	BLOOD: LRTI/Broncholitis	female, born in 2019	CC8	CC8-MSSA (PVL+)	POS	POS	POS	POS	POS	POS	POS	POS	POS	POS	POS	POS	POS	POS	NEG	NEG	NEG	NEG	NEG	NEG	NEG	NEG	NEG	POS	NEG	POS
Trinidad&Tobago_2020-090_56227	Human	Urine: UTI /Hydronephrosis	male, born in 2019	CC8	CC8-MSSA (PVL+)	POS	POS	POS	POS	POS	POS	POS	POS	POS	POS	POS	POS	POS	POS	NEG	NEG	NEG	NEG	NEG	NEG	NEG	NEG	NEG	POS	NEG	POS
Trinidad&Tobago_2020-108_7203M_1	Human	Wound Swab: Appendicitis/Surgical site wound Infections	male, born in 2009	CC8	CC8-MSSA (PVL+)	POS	POS	POS	POS	POS	POS	POS	POS	POS	POS	POS	POS	NEG	POS	NEG	NEG	NEG	NEG	NEG	NEG	NEG	NEG	NEG	NEG	NEG	POS
Trinidad&Tobago_2020-033_7223M	Human	Wound Swab: Surgical site Infections	male, born in 1974	CC8	CC8-MSSA-[ACME] (PVL+)	POS	POS	POS	POS	POS	POS	POS	POS	POS	POS	POS	POS	POS	POS	NEG	NEG	NEG	NEG	NEG	NEG	NEG	NEG	NEG	POS	NEG	POS
>CC9																															
Trinidad&Tobago_2020-061_7673M1	Human	Wound Swab: Post partum Hemmorrhage/Post surgical Infection	female, born in 1972	CC9	CC9-MSSA	POS	POS	POS	POS	POS	POS	POS	POS	POS	POS	NEG	NEG	NEG	NEG	POS	POS	POS	POS	NEG	NEG	NEG	NEG	NEG	NEG	NEG	POS
>CC12																															
Trinidad&Tobago_2020-004_4242M	Human	Wound Swab: Skin sepsis/Umblical stump wound	female, born in 2020	CC12	CC12-MSSA	POS	POS	POS	POS	POS	POS	POS	POS	POS	POS	NEG	NEG	NEG	NEG	POS	POS	POS	POS	NEG	NEG	NEG	NEG	NEG	NEG	NEG	POS
>CC15																															
Trinidad&Tobago_2020-046_7674M	Human	Eye swab: Opthalmia neonatorum	female, born in 2019	CC15	CC15-MSSA	POS	POS	POS	POS	POS	POS	POS	POS	POS	POS	NEG	NEG	NEG	NEG	POS	POS	POS	POS	NEG	NEG	NEG	NEG	NEG	NEG	NEG	POS
Trinidad&Tobago_2020-056_8936U	Human	Urine: UTI	female, born in 1982	CC15	CC15-MSSA	POS	POS	POS	POS	POS	POS	POS	POS	POS	POS	NEG	NEG	NEG	NEG	POS	POS	POS	POS	NEG	NEG	NEG	NEG	NEG	NEG	NEG	POS
Trinidad&Tobago_2020-058_8936A	Human	BLOOD: Sepsis	male, born in 1970	CC15	CC15-MSSA	POS	POS	POS	POS	POS	POS	POS	POS	POS	POS	NEG	NEG	NEG	NEG	POS	POS	POS	POS	NEG	NEG	NEG	NEG	NEG	NEG	NEG	POS
>CC22																															
Trinidad&Tobago_2020-036_7303M	Human	Wound Swab: Cellulitis/Trauma to left foot	male, born in 1972	CC22	CC22-MRSA-IVa (PVL+/tst+)	POS	POS	POS	POS	POS	POS	POS	POS	POS	POS	POS	POS	POS	POS	NEG	NEG	NEG	NEG	NEG	NEG	NEG	NEG	NEG	POS	NEG	POS
>CC30																															
Trinidad&Tobago_2020-095_59556	Human	Wound Swab: Cellulitis	male, born in 1951	CC30	CC30-MSSA	POS	POS	POS	POS	POS	POS	POS	POS	POS	POS	NEG	NEG	NEG	NEG	NEG	NEG	NEG	NEG	POS	POS	POS	POS	NEG	NEG	NEG	POS
>CC45 [agr I]																															
Jamaica_2020-071_JM10	Human	Blood		CC45 [agr I]	CC45-MSSA	POS	POS	POS	POS	POS	POS	POS	POS	POS	POS	POS	POS	POS	POS	NEG	NEG	NEG	NEG	NEG	NEG	NEG	NEG	NEG	NEG	NEG	POS
Jamaica_2020-072_JM11	Human			CC45 [agr I]	CC45-MSSA	POS	POS	POS	POS	POS	POS	POS	POS	POS	POS	POS	POS	POS	POS	NEG	NEG	NEG	NEG	NEG	NEG	NEG	NEG	NEG	NEG	NEG	POS
Trinidad&Tobago_2020-001_5083A/B	Human	BLOOD: Febrile illness/Sepsis	female, born in 1999	CC45 [agr I]	CC45-MSSA	POS	POS	POS	POS	POS	POS	POS	POS	POS	POS	POS	POS	POS	POS	NEG	NEG	NEG	NEG	NEG	NEG	NEG	NEG	NEG	NEG	NEG	POS
Trinidad&Tobago_2020-096_54088	Human	Wound Swab: Cellulitis	male, born in 1983	CC45 [agr I]	CC45-MSSA	POS	POS	POS	POS	POS	POS	POS	POS	POS	POS	POS	POS	POS	POS	NEG	NEG	NEG	NEG	NEG	NEG	NEG	NEG	NEG	NEG	NEG	POS
>CC59																															
Trinidad&Tobago_2020-083_4260B	Human	BLOOD: Neonatal sepsis/Fever, irritability	male, born in 2019	CC59	CC59-MSSA	POS	POS	POS	POS	POS	POS	POS	POS	POS	POS	POS	POS	POS	POS	NEG	NEG	NEG	NEG	NEG	NEG	NEG	NEG	NEG	NEG	NEG	POS
>CC72																															
Jamaica_2020-067_JM06	Human			CC72	ST72-MSSA	POS	POS	POS	POS	POS	POS	POS	POS	POS	POS	POS	POS	POS	POS	NEG	NEG	NEG	NEG	NEG	NEG	NEG	NEG	NEG	NEG	NEG	POS
Jamaica_2020-074_JM13	Human			CC72	ST72-MSSA	POS	POS	POS	POS	POS	POS	POS	POS	POS	POS	POS	POS	POS	POS	NEG	NEG	NEG	NEG	NEG	NEG	NEG	NEG	NEG	POS	NEG	POS
Trinidad&Tobago_2020-100_55943	Human	Wound Swab: R hand abscess	male, born in 1980	CC72	ST72-MSSA	POS	POS	POS	POS	POS	POS	POS	POS	POS	POS	POS	POS	NEG	POS	NEG	NEG	NEG	NEG	NEG	NEG	NEG	NEG	NEG	NEG	NEG	POS
Trinidad&Tobago_2020-101_53748	Human	Wound Swab: Cellulitis L foot	female, born in 1950	CC72	ST72-MSSA	POS	POS	POS	POS	POS	POS	POS	POS	POS	POS	POS	POS	POS	POS	NEG	NEG	NEG	NEG	NEG	NEG	NEG	NEG	AMB	POS	NEG	POS
Trinidad&Tobago_2020-039_7325M	Human	Eye swab: Opthalmia neonatorum	male, born in 2019	CC72	ST72-MRSA-[VT+fus]	POS	POS	POS	POS	POS	POS	POS	POS	POS	POS	POS	POS	POS	POS	NEG	NEG	NEG	NEG	NEG	NEG	NEG	NEG	NEG	POS	NEG	POS
>CC88																															
Trinidad&Tobago_2020-022_7052M	Human	Vulval swab: Vulval abscess/swab from vulva	female, born in 1992	CC88	CC88-MRSA-IVa	POS	POS	POS	POS	POS	POS	POS	POS	POS	POS	NEG	NEG	NEG	NEG	NEG	NEG	NEG	NEG	POS	POS	POS	POS	NEG	NEG	NEG	POS
>CC97																															
Jamaica_2020-068_JM07	Human			CC97	CC97-MSSA	POS	POS	POS	POS	POS	POS	POS	POS	POS	POS	POS	POS	POS	POS	NEG	NEG	NEG	NEG	NEG	NEG	NEG	NEG	NEG	POS	NEG	POS
Trinidad&Tobago_2020-044_7379M	Human	Pus: Cellulitis/Right hand swelling	female, born in 1956	CC97	CC97-MSSA	POS	POS	POS	POS	POS	POS	POS	POS	POS	POS	POS	POS	POS	POS	NEG	NEG	NEG	NEG	NEG	NEG	NEG	NEG	NEG	POS	NEG	POS
Trinidad&Tobago_2020-045_7405M	Human	Wound Swab: Skin abscess/Right knee swelling	male, born in 2006	CC97	CC97-MSSA	POS	POS	POS	POS	POS	POS	POS	POS	POS	POS	POS	POS	POS	POS	NEG	NEG	NEG	NEG	NEG	NEG	NEG	NEG	NEG	POS	NEG	POS
Trinidad&Tobago_2020-047_7673M	Human	Tracheal aspirate: Intraparenchymal haemorrhage	female, born in 1982	CC97	CC97-MSSA	POS	POS	POS	POS	POS	POS	POS	POS	POS	POS	POS	POS	POS	POS	NEG	NEG	NEG	NEG	NEG	NEG	NEG	NEG	NEG	POS	NEG	POS
Trinidad&Tobago_2020-051_8514A	Human	BLOOD: Line Sepsis	male, born in 1983	CC97	CC97-MSSA	POS	POS	POS	POS	POS	POS	POS	POS	POS	POS	POS	POS	POS	POS	NEG	NEG	NEG	NEG	NEG	NEG	NEG	NEG	NEG	POS	NEG	POS
Trinidad&Tobago_2020-053_8755A	Human	BLOOD: Sepsis/ESRD on haemodialysis	male, born in 1955	CC97	CC97-MSSA	POS	POS	POS	POS	POS	POS	POS	POS	POS	POS	POS	POS	POS	POS	NEG	NEG	NEG	NEG	NEG	NEG	NEG	NEG	NEG	POS	NEG	POS
Trinidad&Tobago_2020-054_8803A	Human	BLOOD: Line Sepsis/Cardiac Failure	male, born in 2005	CC97	CC97-MSSA	POS	POS	POS	POS	POS	POS	POS	POS	POS	POS	POS	POS	POS	POS	NEG	NEG	NEG	NEG	NEG	NEG	NEG	NEG	NEG	POS	NEG	POS
Trinidad&Tobago_2020-078_8803U	Human	Urine: UTI	female, born in 1985	CC97	CC97-MSSA	POS	POS	POS	POS	POS	POS	POS	POS	POS	POS	POS	POS	NEG	POS	NEG	NEG	NEG	NEG	NEG	NEG	NEG	NEG	NEG	NEG	NEG	POS
Trinidad&Tobago_2020-079_5639U	Human	Urine: UTI	female, born in 2001	CC97	CC97-MSSA	POS	POS	POS	POS	POS	POS	POS	POS	POS	POS	POS	POS	NEG	POS	NEG	NEG	NEG	NEG	NEG	NEG	NEG	NEG	NEG	NEG	NEG	POS
Trinidad&Tobago_2020-080_8514U	Human	Urine: UTI	female, born in 1966	CC97	CC97-MSSA	POS	POS	POS	POS	POS	POS	POS	POS	POS	POS	POS	POS	POS	POS	NEG	NEG	NEG	NEG	NEG	NEG	NEG	NEG	NEG	NEG	NEG	POS
>CC101																															
Jamaica_2020-075_JM14	Human			CC101	CC101-MSSA	POS	POS	POS	POS	POS	POS	POS	POS	POS	POS	POS	POS	POS	POS	NEG	NEG	NEG	NEG	NEG	NEG	NEG	NEG	NEG	POS	NEG	POS
Trinidad&Tobago_2020-008_362M	Human	Wound Swab: Septic arthritis/Left knee effusion	male, born in 1980	CC101	CC101-MSSA	POS	POS	POS	POS	POS	POS	POS	POS	POS	POS	POS	POS	POS	POS	NEG	NEG	NEG	NEG	NEG	NEG	NEG	NEG	NEG	AMB	NEG	POS
>CC121																															
Trinidad&Tobago_2020-005_4257M	Human	Joint fluid: Septic arthritis/Right knee swelling	male, born in 1940	CC121	CC121-MSSA	POS	POS	POS	POS	POS	POS	POS	POS	POS	POS	NEG	NEG	POS	NEG	NEG	NEG	NEG	NEG	NEG	NEG	NEG	NEG	POS	POS	POS	POS
>CC152																															
Trinidad&Tobago_2020-020_7031M	Human	Wound Swab: Surgical site/post surgical Infection	male, born in 1986	CC152	CC152-MSSA	POS	POS	POS	POS	POS	POS	AMB	POS	POS	POS	POS	POS	POS	POS	NEG	NEG	NEG	NEG	NEG	NEG	NEG	NEG	POS	POS	NEG	POS
Jamaica_2020-063_JM02	Human			CC152	CC152-MSSA (PVL+)	POS	POS	POS	POS	POS	POS	AMB	POS	POS	POS	POS	POS	POS	POS	NEG	NEG	NEG	NEG	NEG	NEG	NEG	NEG	POS	POS	NEG	POS
Jamaica_2020-066_JM05	Human	Wound swab		CC152	CC152-MSSA (PVL+)	POS	POS	POS	POS	POS	POS	AMB	POS	POS	POS	POS	POS	POS	POS	NEG	NEG	NEG	NEG	NEG	NEG	NEG	NEG	AMB	POS	NEG	POS
Trinidad&Tobago_2020-006_391M	Human	Wound Swab: Tenosynovitis/4th right digit Flexo swelling	male, born in 1980	CC152	CC152-MSSA (PVL+)	POS	POS	POS	POS	POS	POS	AMB	POS	POS	POS	POS	POS	POS	POS	NEG	NEG	NEG	NEG	NEG	NEG	NEG	NEG	POS	POS	NEG	POS
Trinidad&Tobago_2020-012_4176M	Human	Pus: Digit abscess/Right situ digit abscess	female, born in 2004	CC152	CC152-MSSA (PVL+)	POS	POS	POS	POS	POS	POS	AMB	POS	POS	POS	POS	POS	POS	POS	NEG	NEG	NEG	NEG	NEG	NEG	NEG	NEG	POS	POS	NEG	POS
Trinidad&Tobago_2020-024_7084M	Human	Pus swab: Skin abscess/Abscess on the hand	male, born in 1983	CC152	CC152-MSSA (PVL+)	POS	POS	POS	POS	POS	POS	AMB	POS	POS	POS	POS	POS	POS	POS	NEG	NEG	NEG	NEG	NEG	NEG	NEG	NEG	POS	POS	NEG	POS
Trinidad&Tobago_2020-025_7089M	Human	Wound Swab: Cellulitis/Left middle finger infection	male, born in 1993	CC152	CC152-MSSA (PVL+)	POS	POS	POS	POS	POS	POS	AMB	POS	POS	POS	POS	POS	POS	POS	NEG	NEG	NEG	NEG	NEG	NEG	NEG	NEG	AMB	POS	NEG	POS
Trinidad&Tobago_2020-026_7187M	Human	Wound Swab: Surgical site Infections/Day 9 Post C-section	female, born in 2002	CC152	CC152-MSSA (PVL+)	POS	POS	POS	POS	POS	POS	AMB	POS	POS	POS	POS	POS	POS	POS	NEG	NEG	NEG	NEG	NEG	NEG	NEG	NEG	POS	POS	NEG	POS
Trinidad&Tobago_2020-032_7275M	Human	Pus: Breast abscess	female, born in 2004	CC152	CC152-MSSA (PVL+)	POS	POS	POS	POS	POS	POS	AMB	POS	POS	POS	POS	POS	POS	POS	NEG	NEG	NEG	NEG	NEG	NEG	NEG	NEG	AMB	POS	NEG	POS
Trinidad&Tobago_2020-037_7307M	Human	Ear swab: Ear Infection/Ruptured Tympanic membrane	male, born in 2011	CC152	CC152-MSSA (PVL+)	POS	POS	POS	POS	POS	POS	AMB	POS	POS	POS	POS	POS	POS	POS	NEG	NEG	NEG	NEG	NEG	NEG	NEG	NEG	POS	POS	NEG	POS
Trinidad&Tobago_2020-041_7335M	Human	Wound Swab: Skin abscess/Swollen right thigh	female, born in 1984	CC152	CC152-MSSA (PVL+)	POS	POS	POS	POS	POS	POS	AMB	POS	POS	POS	POS	POS	POS	POS	NEG	NEG	NEG	NEG	NEG	NEG	NEG	NEG	POS	POS	NEG	POS
Trinidad&Tobago_2020-089_54136	Human	Wound Swab: Left knee effusion	male, born in 1964	CC152	CC152-MSSA (PVL+)	POS	POS	POS	POS	POS	AMB	NEG	POS	POS	POS	POS	NEG	POS	POS	NEG	NEG	NEG	NEG	NEG	NEG	NEG	NEG	NEG	NEG	NEG	POS
Trinidad&Tobago_2020-091_59153	Human	L Ear swab: Ear Infection	female, born in 2019	CC152	CC152-MSSA (PVL+)	POS	POS	POS	POS	POS	POS	AMB	POS	POS	POS	POS	POS	POS	POS	NEG	NEG	NEG	NEG	NEG	NEG	NEG	NEG	AMB	POS	NEG	POS
Trinidad&Tobago_2020-092_54817	Human	Rt leg abscess: Cellulitis	female, born in 2009	CC152	CC152-MSSA (PVL+)	POS	POS	POS	POS	POS	POS	AMB	POS	POS	POS	POS	POS	POS	POS	NEG	NEG	NEG	NEG	NEG	NEG	NEG	NEG	AMB	POS	NEG	POS
Trinidad&Tobago_2020-093_55998	Human	Urine: UTI	female, born in 1974	CC152	CC152-MSSA (PVL+)	POS	POS	POS	POS	POS	POS	AMB	POS	POS	POS	POS	POS	POS	POS	NEG	NEG	NEG	NEG	NEG	NEG	NEG	NEG	POS	POS	NEG	POS
Trinidad&Tobago_2020-097_55558	Human	Wound Swab: Abscess	female, born in 1957	CC152	CC152-MSSA (PVL+)	POS	POS	POS	POS	POS	POS	AMB	POS	POS	POS	POS	POS	POS	POS	NEG	NEG	NEG	NEG	NEG	NEG	NEG	NEG	POS	POS	NEG	POS
>CC188																															
Jamaica_2020-069_JM08	Human			CC188	CC188-MSSA	POS	POS	POS	POS	POS	POS	POS	POS	POS	POS	POS	POS	POS	POS	NEG	NEG	NEG	NEG	NEG	NEG	NEG	NEG	NEG	POS	NEG	POS
>CC239																															
Trinidad&Tobago_2020-040_7327M	Human	Wound Swab: Surgical site/Wound Infection	female, born in 1989	CC239	CC239-MRSA-[III+SCCmer+ccrC]	POS	POS	POS	POS	POS	POS	POS	POS	POS	POS	POS	POS	POS	POS	NEG	NEG	NEG	NEG	NEG	NEG	NEG	NEG	NEG	POS	NEG	POS
Trinidad&Tobago_2020-060_7328M	Human	Wound Swab: Appendicitis/Post surgical Infection	female, born in 2015	CC239	CC239-MRSA-[III+SCCmer+ccrC]	POS	POS	POS	POS	POS	POS	POS	POS	POS	POS	POS	POS	POS	POS	NEG	NEG	NEG	NEG	NEG	NEG	NEG	NEG	NEG	POS	NEG	POS
Trinidad&Tobago_2020-009_371M	Human	Wound Swab: Skin sepsis/Left ankle sepsis	male, born in 1966	CC239	CC239-MRSA-[mec III variant] (atypical "SE-Asian Clade")	POS	POS	POS	POS	POS	POS	POS	POS	POS	POS	POS	POS	POS	POS	NEG	NEG	NEG	NEG	NEG	NEG	NEG	NEG	NEG	NEG	NEG	POS
Trinidad&Tobago_2020-010_372M	Human	Wound Swab: Skin sepsis/Thigh (donor site) wound infection	male, born in 1966	CC239	CC239-MRSA-[mec III variant] (atypical "SE-Asian Clade")	POS	POS	POS	POS	POS	POS	POS	POS	POS	POS	POS	POS	POS	POS	NEG	NEG	NEG	NEG	NEG	NEG	NEG	NEG	NEG	AMB	NEG	POS
Trinidad&Tobago_2020-021_7037M	Human	Wound Swab: Surgical site/post surgical Infection	female, born in 1955	CC239	CC239-MRSA-[mec III variant] (atypical "SE-Asian Clade")	POS	POS	POS	POS	POS	POS	POS	POS	POS	POS	POS	POS	POS	POS	NEG	NEG	NEG	NEG	NEG	NEG	NEG	NEG	NEG	POS	NEG	POS
Trinidad&Tobago_2020-048_8421A	Human	BLOOD: Sepsi	female, born in 1968	CC239	CC239-MRSA-[mec III variant] (atypical "SE-Asian Clade")	POS	POS	POS	POS	POS	POS	POS	POS	POS	POS	POS	POS	POS	POS	NEG	NEG	NEG	NEG	NEG	NEG	NEG	NEG	NEG	POS	NEG	POS
Trinidad&Tobago_2020-076_8482B	Human	BLOOD: Biphenotypic leukaemia/Blood port Sepsis	female, born in 2008	CC239	CC239-MRSA-[mec III variant] (atypical "SE-Asian Clade")	POS	POS	POS	POS	POS	POS	POS	POS	POS	POS	POS	POS	POS	POS	NEG	NEG	NEG	NEG	NEG	NEG	NEG	NEG	NEG	POS	NEG	POS
>CC398																															
Trinidad&Tobago_2020-023_7056M	Human	Wound Swab: Surgical site/sternal wound infection	male, born in 1982	CC398	CC398-MSSA	POS	POS	POS	POS	POS	POS	POS	POS	POS	POS	POS	POS	POS	POS	NEG	NEG	NEG	NEG	NEG	NEG	NEG	NEG	NEG	NEG	NEG	POS
Trinidad&Tobago_2020-084_55982	Human	Wound Swab: Left subclavian catheter discharge	female, born in 1992	CC398	CC398-MSSA	POS	POS	POS	POS	POS	POS	POS	POS	POS	POS	POS	POS	POS	POS	NEG	NEG	NEG	NEG	NEG	NEG	NEG	NEG	NEG	NEG	NEG	POS
Trinidad&Tobago_2020-085_55722	Human	Wound swab: Scrotal abscess	male, born in 2015	CC398	CC398-MSSA	POS	POS	POS	POS	POS	POS	POS	POS	POS	POS	POS	POS	POS	POS	NEG	NEG	NEG	NEG	NEG	NEG	NEG	NEG	NEG	NEG	NEG	POS
Trinidad&Tobago_2020-094_55111	Human	Wound Swab: Right knee cellulitis	male, born in 1965	CC398	CC398-MSSA	POS	POS	POS	POS	POS	POS	POS	POS	POS	POS	POS	POS	POS	POS	NEG	NEG	NEG	NEG	NEG	NEG	NEG	NEG	NEG	NEG	NEG	POS
Trinidad&Tobago_2020-104_6929M	Human	Pus: Sporotrichosis	male, born in 2009	CC398	CC398-MSSA	POS	POS	POS	POS	POS	POS	POS	POS	POS	POS	POS	POS	POS	POS	NEG	NEG	NEG	NEG	NEG	NEG	NEG	NEG	NEG	NEG	NEG	POS
Argenteus CC2596																															
Trinidad-Tobago_SSTI-2020_098_59348	Human	Blood: Sepsis	female, born in 1976		SCC negatives	POS	POS	POS	NEG	NEG	NEG	NEG	POS	POS	POS	NEG	NEG	NEG	NEG	NEG	NEG	NEG	NEG	NEG	POS	NEG	NEG	NEG	NEG	NEG	POS
Trinidad-Tobago_SSTI-2020_102_54485	Human	Wound Swab: L Thumb abscess	male, born in 2015		SCC negatives	POS	POS	POS	NEG	NEG	NEG	NEG	POS	POS	POS	NEG	NEG	NEG	NEG	NEG	NEG	NEG	NEG	NEG	POS	NEG	NEG	NEG	NEG	NEG	POS

METHICILLIN RESISTANCE AND SCCmec TYPING																															HEAVY METAL RESISTANCES		RESISTANCE : PENICILLINASE			RESISTANCE : MLS-ANTIBIOTICS											
									kdp-SCC locus						ACME-SCC locus					ccrA/B-01		ccrA/B-02		ccrA/B-03		ccrC		ccrA/B-04																	vga(A)		
ugpQ	mecA	delta_mecR	mecR	mecI	xylR/ mecR2	mecC	blaZ (SCC mec XI)	plsSCC (COL)	kdpA-SCC	kdpB-SCC	kdpC-SCC	kdpD-SCC	kdpE-SCC	fusC (Q6GD50)	ACME total	arcA-SCC	arcB-SCC	arcC-SCC	arcD-SCC	ccrA-1	ccrB-1	ccrA-2	ccrB-2	ccrA-3	ccrB-3	ccrAA	ccrC	ccrA-4	ccrB-4	Q9X B68 - dcs	merA	merB	blaZ	blaI	blaR	ermA	ermB	ermC	linA/linuA	msrA	mefA	mph(C)	vat(A)	vat(B)	vga(A)	vga(A) (BM 3327)	vgb
Glycerophosphoryl diester phosphodiesterase. Accompanies mecA in nearly all SCCmec	Modified penicillin binding protein (PBP2a)	Truncated methicillin resistance operon repressor 1. Truncated mecR1 is	Methicillin resistance operon repressor 1. Un-truncated sequence in SCCmec II	Methicillin-resistance regulatory protein. Present in SCCmec II (although	Methicillin resistance operon repressor 2, Homolog of xylose repressor	Alternate gene encoding a modified penicillin binding protein . Present in, and	Beta-lactamase from SCCmec XI	Plasmin-sensitive surface protein, prevents bacterial adhesion in	Potassium-translocating ATPase A, chain 2	Potassium-transporting ATPase B, chain 1	Potassium-translocating ATPase C, chain 2	Sensor kinase protein	KDP operon transcriptional regulatory protein	SCC-associated fusidic acid resistance gene. Present alone in "SCCfus" elements or	ACME total	Arginine deiminase	Ornithine carbamoyltransferase	Carbamate kinase	Arginine/ornithine antiporter	Cassette chromosome recombinase A, type 1	Cassette chromosome recombinase B, type 1	Cassette chromosome recombinase A, type 2	Cassette chromosome recombinase B, type 2	Cassette chromosome recombinase A, type 3	Cassette chromosome recombinase B, type 3	Cassette chromosome recombinase AA/C		Cassette chromosome recombinase A, type 4	Cassette chromosome recombinase B, type 4	Located at the terminus of SCCmec directly next to orfX. This locus comprises the downstream	mercury resistance operon		beta-lactamase	beta lactamase repressor (inhibitor)	beta-lactamase regulatory protein	rRNA adenine N-6-methyl-transferase, erythromycin/ clindamycin resistance	erythro-mycin/clindamycin resistance	erythro-mycin/clindamycin resistance	Lincosamid-Nucleotidyltransferase	energy-dependent efflux of erythro-mycin	macrolide efflux protein A	probable lysylphos-phatidyl-glycerol synthetase	virginia-mycin A acetyltransferase	acetyl-transferase inactivating streptogramin A	ATP binding protein, streptogramin-A-resistance		virginia-mycin B hydrolase
NEG	NEG	NEG	NEG	NEG	NEG	NEG	NEG	NEG	NEG	NEG	NEG	NEG	NEG	NEG	NEG	NEG	NEG	NEG	NEG	NEG	NEG	NEG	NEG	NEG	NEG	NEG	NEG	NEG	NEG	NEG	NEG	NEG	POS	POS	POS	NEG	NEG	NEG	NEG	NEG	NEG	NEG	NEG	NEG	NEG	NEG	NEG
NEG	NEG	NEG	NEG	NEG	NEG	NEG	NEG	NEG	NEG	NEG	NEG	NEG	NEG	NEG	NEG	NEG	NEG	NEG	NEG	NEG	NEG	NEG	NEG	NEG	NEG	NEG	NEG	NEG	NEG	NEG	NEG	NEG	POS	POS	POS	NEG	NEG	NEG	NEG	NEG	NEG	NEG	NEG	NEG	NEG	NEG	NEG
NEG	NEG	NEG	NEG	NEG	NEG	NEG	NEG	NEG	NEG	NEG	NEG	NEG	NEG	NEG	NEG	NEG	NEG	NEG	NEG	NEG	NEG	NEG	NEG	NEG	NEG	NEG	NEG	NEG	NEG	NEG	NEG	NEG	POS	POS	POS	NEG	NEG	NEG	NEG	NEG	NEG	NEG	NEG	NEG	NEG	NEG	NEG
NEG	NEG	NEG	NEG	NEG	NEG	NEG	NEG	NEG	NEG	NEG	NEG	NEG	NEG	NEG	NEG	NEG	NEG	NEG	NEG	NEG	NEG	NEG	NEG	NEG	NEG	NEG	NEG	NEG	NEG	NEG	NEG	NEG	POS	POS	POS	NEG	NEG	NEG	NEG	NEG	NEG	NEG	NEG	NEG	NEG	NEG	NEG
NEG	NEG	NEG	NEG	NEG	NEG	NEG	NEG	NEG	NEG	NEG	NEG	NEG	NEG	NEG	NEG	NEG	NEG	NEG	NEG	NEG	NEG	NEG	NEG	NEG	NEG	NEG	NEG	NEG	NEG	NEG	NEG	NEG	NEG	NEG	NEG	NEG	NEG	NEG	NEG	NEG	NEG	NEG	NEG	NEG	NEG	NEG	NEG
NEG	NEG	NEG	NEG	NEG	NEG	NEG	NEG	NEG	NEG	NEG	NEG	NEG	NEG	NEG	NEG	NEG	NEG	NEG	NEG	NEG	NEG	NEG	NEG	NEG	NEG	NEG	NEG	NEG	NEG	NEG	NEG	NEG	POS	POS	AMB	NEG	NEG	NEG	NEG	NEG	NEG	NEG	NEG	NEG	NEG	NEG	NEG
NEG	NEG	NEG	NEG	NEG	NEG	NEG	NEG	NEG	NEG	NEG	NEG	NEG	NEG	NEG	NEG	NEG	NEG	NEG	NEG	NEG	NEG	NEG	NEG	NEG	NEG	NEG	NEG	NEG	NEG	NEG	NEG	NEG	POS	POS	POS	NEG	NEG	NEG	NEG	NEG	NEG	NEG	NEG	NEG	NEG	NEG	NEG
NEG	NEG	NEG	NEG	NEG	NEG	NEG	NEG	NEG	NEG	NEG	NEG	NEG	NEG	NEG	NEG	NEG	NEG	NEG	NEG	NEG	NEG	NEG	NEG	NEG	NEG	NEG	NEG	NEG	NEG	NEG	NEG	NEG	POS	POS	POS	NEG	NEG	NEG	NEG	NEG	NEG	NEG	NEG	NEG	NEG	NEG	NEG
NEG	NEG	NEG	NEG	NEG	NEG	NEG	NEG	NEG	NEG	NEG	NEG	NEG	NEG	NEG	NEG	NEG	NEG	NEG	NEG	NEG	NEG	NEG	NEG	NEG	NEG	NEG	NEG	NEG	NEG	NEG	NEG	NEG	NEG	NEG	NEG	NEG	NEG	NEG	NEG	NEG	NEG	NEG	NEG	NEG	NEG	NEG	NEG
NEG	NEG	NEG	NEG	NEG	NEG	NEG	NEG	NEG	NEG	NEG	NEG	NEG	NEG	NEG	NEG	NEG	NEG	NEG	NEG	NEG	NEG	NEG	NEG	NEG	NEG	NEG	NEG	NEG	NEG	NEG	NEG	NEG	POS	POS	POS	NEG	NEG	NEG	NEG	NEG	NEG	NEG	NEG	NEG	NEG	NEG	NEG
NEG	NEG	NEG	NEG	NEG	NEG	NEG	NEG	NEG	NEG	NEG	NEG	NEG	NEG	NEG	NEG	NEG	NEG	NEG	NEG	NEG	NEG	NEG	NEG	NEG	NEG	NEG	NEG	NEG	NEG	NEG	NEG	NEG	NEG	NEG	NEG	NEG	NEG	NEG	NEG	POS	NEG	NEG	NEG	NEG	NEG	NEG	NEG
NEG	NEG	NEG	NEG	NEG	NEG	NEG	NEG	NEG	NEG	NEG	NEG	NEG	NEG	NEG	NEG	NEG	NEG	NEG	NEG	NEG	NEG	NEG	NEG	NEG	NEG	NEG	NEG	NEG	NEG	NEG	NEG	NEG	POS	POS	POS	NEG	NEG	NEG	NEG	NEG	NEG	NEG	NEG	NEG	NEG	NEG	NEG
NEG	NEG	NEG	NEG	NEG	NEG	NEG	NEG	NEG	NEG	NEG	NEG	NEG	NEG	NEG	NEG	NEG	NEG	NEG	NEG	NEG	NEG	NEG	NEG	NEG	NEG	NEG	NEG	NEG	NEG	NEG	NEG	NEG	POS	POS	POS	NEG	NEG	NEG	NEG	NEG	NEG	NEG	NEG	NEG	NEG	NEG	NEG
NEG	NEG	NEG	NEG	NEG	NEG	NEG	NEG	NEG	NEG	NEG	NEG	NEG	NEG	NEG	NEG	NEG	NEG	NEG	NEG	NEG	NEG	NEG	NEG	NEG	NEG	NEG	NEG	NEG	NEG	NEG	NEG	NEG	POS	POS	POS	NEG	NEG	NEG	NEG	NEG	NEG	NEG	NEG	NEG	NEG	NEG	NEG
NEG	NEG	NEG	NEG	NEG	NEG	NEG	NEG	NEG	NEG	NEG	NEG	NEG	NEG	NEG	NEG	NEG	NEG	NEG	NEG	NEG	NEG	NEG	NEG	NEG	NEG	NEG	NEG	NEG	NEG	NEG	NEG	NEG	POS	POS	POS	NEG	NEG	NEG	NEG	NEG	NEG	NEG	NEG	NEG	NEG	NEG	NEG
NEG	NEG	NEG	NEG	NEG	NEG	NEG	NEG	NEG	NEG	NEG	NEG	NEG	NEG	NEG	NEG	NEG	NEG	NEG	NEG	NEG	NEG	NEG	NEG	NEG	NEG	NEG	NEG	NEG	NEG	NEG	NEG	NEG	POS	POS	POS	NEG	NEG	NEG	NEG	NEG	NEG	NEG	NEG	NEG	NEG	NEG	NEG
NEG	NEG	NEG	NEG	NEG	NEG	NEG	NEG	NEG	NEG	NEG	NEG	NEG	NEG	NEG	NEG	NEG	NEG	NEG	NEG	NEG	NEG	NEG	NEG	NEG	NEG	NEG	NEG	NEG	NEG	NEG	NEG	NEG	POS	POS	POS	NEG	NEG	NEG	NEG	NEG	NEG	NEG	NEG	NEG	NEG	NEG	NEG
NEG	NEG	NEG	NEG	NEG	NEG	NEG	NEG	NEG	NEG	NEG	NEG	NEG	NEG	NEG	NEG	NEG	NEG	NEG	NEG	NEG	NEG	NEG	NEG	NEG	NEG	NEG	NEG	NEG	NEG	NEG	NEG	NEG	NEG	NEG	NEG	NEG	NEG	NEG	NEG	NEG	NEG	NEG	NEG	NEG	NEG	NEG	NEG
NEG	NEG	NEG	NEG	NEG	NEG	NEG	NEG	NEG	NEG	NEG	NEG	NEG	NEG	NEG	NEG	NEG	NEG	NEG	NEG	NEG	NEG	NEG	NEG	NEG	NEG	NEG	NEG	NEG	NEG	NEG	NEG	NEG	POS	POS	POS	NEG	NEG	NEG	NEG	NEG	NEG	NEG	NEG	NEG	NEG	NEG	NEG
NEG	NEG	NEG	NEG	NEG	NEG	NEG	NEG	NEG	NEG	NEG	NEG	NEG	NEG	NEG	NEG	NEG	NEG	NEG	NEG	NEG	NEG	NEG	NEG	NEG	NEG	NEG	NEG	NEG	NEG	NEG	NEG	NEG	POS	POS	POS	NEG	NEG	NEG	NEG	NEG	NEG	NEG	NEG	NEG	NEG	NEG	NEG
NEG	NEG	NEG	NEG	NEG	NEG	NEG	NEG	NEG	NEG	NEG	NEG	NEG	NEG	NEG	NEG	NEG	NEG	NEG	NEG	NEG	NEG	NEG	NEG	NEG	NEG	NEG	NEG	NEG	NEG	NEG	NEG	NEG	POS	POS	POS	NEG	NEG	NEG	NEG	NEG	NEG	NEG	NEG	NEG	NEG	NEG	NEG
NEG	NEG	NEG	NEG	NEG	NEG	NEG	NEG	NEG	NEG	NEG	NEG	NEG	NEG	NEG	NEG	NEG	NEG	NEG	NEG	NEG	NEG	NEG	NEG	NEG	NEG	NEG	NEG	NEG	NEG	NEG	NEG	NEG	POS	POS	POS	NEG	NEG	NEG	AMB	NEG	NEG	NEG	NEG	NEG	NEG	NEG	NEG
NEG	NEG	NEG	NEG	NEG	NEG	NEG	NEG	NEG	NEG	NEG	NEG	NEG	NEG	NEG	NEG	NEG	NEG	NEG	NEG	NEG	NEG	NEG	NEG	NEG	NEG	NEG	NEG	NEG	NEG	NEG	NEG	NEG	POS	POS	POS	NEG	NEG	NEG	NEG	NEG	NEG	NEG	NEG	NEG	NEG	NEG	NEG
NEG	NEG	NEG	NEG	NEG	NEG	NEG	NEG	NEG	NEG	NEG	NEG	NEG	NEG	NEG	NEG	NEG	NEG	NEG	NEG	NEG	NEG	NEG	NEG	NEG	NEG	NEG	NEG	NEG	NEG	NEG	NEG	NEG	POS	POS	POS	NEG	NEG	NEG	NEG	NEG	NEG	NEG	NEG	NEG	NEG	NEG	NEG
NEG	NEG	NEG	NEG	NEG	NEG	NEG	NEG	NEG	NEG	NEG	NEG	NEG	NEG	NEG	NEG	NEG	NEG	NEG	NEG	NEG	NEG	NEG	NEG	NEG	NEG	NEG	NEG	NEG	NEG	NEG	NEG	NEG	NEG	NEG	NEG	NEG	NEG	NEG	NEG	NEG	NEG	NEG	NEG	NEG	NEG	NEG	NEG
NEG	NEG	NEG	NEG	NEG	NEG	NEG	NEG	NEG	NEG	NEG	NEG	NEG	NEG	NEG	NEG	NEG	NEG	NEG	NEG	NEG	NEG	NEG	NEG	NEG	NEG	NEG	NEG	NEG	NEG	NEG	NEG	NEG	NEG	NEG	NEG	NEG	NEG	NEG	NEG	NEG	NEG	NEG	NEG	NEG	NEG	NEG	NEG
NEG	NEG	NEG	NEG	NEG	NEG	NEG	NEG	NEG	NEG	NEG	NEG	NEG	NEG	NEG	NEG	NEG	NEG	NEG	NEG	NEG	NEG	NEG	NEG	NEG	NEG	NEG	NEG	NEG	NEG	NEG	NEG	NEG	POS	POS	POS	NEG	NEG	NEG	NEG	NEG	NEG	NEG	NEG	NEG	NEG	NEG	NEG
NEG	NEG	NEG	NEG	NEG	NEG	NEG	NEG	NEG	NEG	NEG	NEG	NEG	NEG	NEG	NEG	NEG	NEG	NEG	NEG	NEG	NEG	NEG	NEG	NEG	NEG	NEG	NEG	NEG	NEG	NEG	NEG	NEG	POS	POS	POS	NEG	NEG	NEG	NEG	NEG	NEG	NEG	NEG	NEG	NEG	NEG	NEG
NEG	NEG	NEG	NEG	NEG	NEG	NEG	NEG	NEG	NEG	NEG	NEG	NEG	NEG	NEG	NEG	NEG	NEG	NEG	NEG	NEG	NEG	NEG	NEG	NEG	NEG	NEG	NEG	NEG	NEG	NEG	NEG	NEG	POS	POS	POS	NEG	NEG	NEG	NEG	NEG	NEG	NEG	NEG	NEG	NEG	NEG	NEG
NEG	NEG	NEG	NEG	NEG	NEG	NEG	NEG	NEG	NEG	NEG	NEG	NEG	NEG	NEG	NEG	NEG	NEG	NEG	NEG	NEG	NEG	NEG	NEG	NEG	NEG	NEG	NEG	NEG	NEG	NEG	NEG	NEG	POS	POS	POS	NEG	NEG	NEG	NEG	NEG	NEG	NEG	NEG	NEG	NEG	NEG	NEG
NEG	NEG	NEG	NEG	NEG	NEG	NEG	NEG	NEG	NEG	NEG	NEG	NEG	NEG	NEG	NEG	NEG	NEG	NEG	NEG	NEG	NEG	NEG	NEG	NEG	NEG	NEG	NEG	NEG	NEG	NEG	NEG	NEG	POS	POS	POS	NEG	NEG	NEG	NEG	NEG	NEG	NEG	NEG	NEG	NEG	NEG	NEG
NEG	NEG	NEG	NEG	NEG	NEG	NEG	NEG	NEG	NEG	NEG	NEG	NEG	NEG	NEG	NEG	NEG	NEG	NEG	NEG	NEG	NEG	NEG	NEG	NEG	NEG	NEG	NEG	NEG	NEG	NEG	NEG	NEG	POS	POS	POS	NEG	NEG	NEG	NEG	NEG	NEG	NEG	NEG	NEG	NEG	NEG	NEG
NEG	NEG	NEG	NEG	NEG	NEG	NEG	NEG	NEG	NEG	NEG	NEG	NEG	NEG	NEG	NEG	NEG	NEG	NEG	NEG	NEG	NEG	NEG	NEG	NEG	NEG	NEG	NEG	NEG	NEG	NEG	NEG	NEG	POS	POS	POS	NEG	NEG	NEG	NEG	NEG	NEG	NEG	NEG	NEG	NEG	NEG	NEG
NEG	NEG	NEG	NEG	NEG	NEG	NEG	NEG	NEG	NEG	NEG	NEG	NEG	NEG	NEG	NEG	NEG	NEG	NEG	NEG	NEG	NEG	NEG	NEG	NEG	NEG	NEG	NEG	NEG	NEG	NEG	NEG	NEG	POS	POS	POS	NEG	NEG	NEG	NEG	NEG	NEG	NEG	NEG	NEG	NEG	NEG	NEG
NEG	NEG	NEG	NEG	NEG	NEG	NEG	NEG	NEG	NEG	NEG	NEG	NEG	NEG	NEG	NEG	NEG	NEG	NEG	NEG	NEG	NEG	NEG	NEG	NEG	NEG	NEG	NEG	NEG	NEG	NEG	NEG	NEG	POS	POS	POS	NEG	NEG	NEG	NEG	NEG	NEG	NEG	NEG	NEG	NEG	NEG	NEG
NEG	NEG	NEG	NEG	NEG	NEG	NEG	NEG	NEG	NEG	NEG	NEG	NEG	NEG	NEG	NEG	NEG	NEG	NEG	NEG	NEG	NEG	NEG	NEG	NEG	NEG	NEG	NEG	NEG	NEG	NEG	NEG	NEG	POS	POS	POS	NEG	NEG	NEG	NEG	NEG	NEG	NEG	NEG	NEG	NEG	NEG	NEG
NEG	NEG	NEG	NEG	NEG	NEG	NEG	NEG	NEG	NEG	NEG	NEG	NEG	NEG	NEG	NEG	NEG	NEG	NEG	NEG	NEG	NEG	NEG	NEG	NEG	NEG	NEG	NEG	NEG	NEG	NEG	NEG	NEG	POS	POS	POS	NEG	NEG	NEG	POS	NEG	NEG	NEG	NEG	NEG	NEG	NEG	NEG
NEG	NEG	NEG	NEG	NEG	NEG	NEG	NEG	NEG	NEG	NEG	NEG	NEG	NEG	NEG	NEG	NEG	NEG	NEG	NEG	NEG	NEG	NEG	NEG	NEG	NEG	NEG	NEG	NEG	NEG	NEG	NEG	NEG	POS	POS	POS	NEG	NEG	NEG	NEG	NEG	NEG	NEG	NEG	NEG	NEG	NEG	NEG
NEG	NEG	NEG	NEG	NEG	NEG	NEG	NEG	NEG	NEG	NEG	NEG	NEG	NEG	NEG	POS	POS	POS	POS	POS	NEG	NEG	NEG	NEG	NEG	NEG	NEG	NEG	NEG	NEG	NEG	NEG	NEG	NEG	NEG	NEG	NEG	NEG	NEG	NEG	POS	NEG	POS	NEG	NEG	NEG	NEG	NEG
NEG	NEG	NEG	NEG	NEG	NEG	NEG	NEG	NEG	NEG	NEG	NEG	NEG	NEG	NEG	NEG	NEG	NEG	NEG	NEG	NEG	NEG	NEG	NEG	NEG	NEG	NEG	NEG	NEG	NEG	NEG	NEG	NEG	POS	POS	POS	NEG	NEG	NEG	NEG	NEG	NEG	NEG	NEG	NEG	NEG	NEG	NEG
NEG	NEG	NEG	NEG	NEG	NEG	NEG	NEG	NEG	NEG	NEG	NEG	NEG	NEG	NEG	NEG	NEG	NEG	NEG	NEG	NEG	NEG	NEG	NEG	NEG	NEG	NEG	NEG	NEG	NEG	NEG	NEG	NEG	NEG	NEG	NEG	NEG	NEG	NEG	NEG	NEG	NEG	NEG	NEG	NEG	NEG	NEG	NEG
NEG	NEG	NEG	NEG	NEG	NEG	NEG	NEG	NEG	NEG	NEG	NEG	NEG	NEG	NEG	NEG	NEG	NEG	NEG	NEG	NEG	NEG	NEG	NEG	NEG	NEG	NEG	NEG	NEG	NEG	NEG	NEG	NEG	POS	POS	POS	NEG	NEG	NEG	NEG	NEG	NEG	NEG	NEG	NEG	NEG	NEG	NEG
NEG	NEG	NEG	NEG	NEG	NEG	NEG	NEG	NEG	NEG	NEG	NEG	NEG	NEG	NEG	NEG	NEG	NEG	NEG	NEG	NEG	NEG	NEG	NEG	NEG	NEG	NEG	NEG	NEG	NEG	NEG	NEG	NEG	NEG	NEG	NEG	NEG	NEG	NEG	NEG	NEG	NEG	NEG	NEG	NEG	NEG	NEG	NEG
NEG	NEG	NEG	NEG	NEG	NEG	NEG	NEG	NEG	NEG	NEG	NEG	NEG	NEG	NEG	NEG	NEG	NEG	NEG	NEG	NEG	NEG	NEG	NEG	NEG	NEG	NEG	NEG	NEG	NEG	NEG	NEG	NEG	NEG	NEG	NEG	NEG	NEG	NEG	NEG	NEG	NEG	NEG	NEG	NEG	NEG	NEG	NEG
POS	POS	POS	NEG	NEG	NEG	NEG	NEG	NEG	NEG	NEG	NEG	NEG	NEG	NEG	NEG	NEG	NEG	NEG	NEG	NEG	NEG	POS	POS	NEG	NEG	NEG	NEG	NEG	NEG	POS	NEG	NEG	POS	POS	POS	NEG	NEG	POS	NEG	NEG	NEG	NEG	NEG	NEG	NEG	NEG	NEG
NEG	NEG	NEG	NEG	NEG	NEG	NEG	NEG	NEG	NEG	NEG	NEG	NEG	NEG	NEG	NEG	NEG	NEG	NEG	NEG	NEG	NEG	NEG	NEG	NEG	NEG	NEG	NEG	NEG	NEG	NEG	NEG	NEG	NEG	NEG	NEG	NEG	NEG	NEG	NEG	NEG	NEG	NEG	NEG	NEG	NEG	NEG	NEG
NEG	NEG	NEG	NEG	NEG	NEG	NEG	NEG	NEG	NEG	NEG	NEG	NEG	NEG	NEG	NEG	NEG	NEG	NEG	NEG	NEG	NEG	NEG	NEG	NEG	NEG	NEG	NEG	NEG	NEG	NEG	NEG	NEG	POS	POS	POS	NEG	NEG	NEG	NEG	NEG	NEG	NEG	NEG	NEG	NEG	NEG	NEG
NEG	NEG	NEG	NEG	NEG	NEG	NEG	NEG	NEG	NEG	NEG	NEG	NEG	NEG	NEG	NEG	NEG	NEG	NEG	NEG	NEG	NEG	NEG	NEG	NEG	NEG	NEG	NEG	NEG	NEG	NEG	NEG	NEG	POS	POS	POS	NEG	NEG	NEG	NEG	NEG	NEG	NEG	NEG	NEG	NEG	NEG	NEG
NEG	NEG	NEG	NEG	NEG	NEG	NEG	NEG	NEG	NEG	NEG	NEG	NEG	NEG	NEG	NEG	NEG	NEG	NEG	NEG	NEG	NEG	NEG	NEG	NEG	NEG	NEG	NEG	NEG	NEG	NEG	NEG	NEG	POS	POS	POS	NEG	NEG	NEG	NEG	NEG	NEG	NEG	NEG	NEG	NEG	NEG	NEG
NEG	NEG	NEG	NEG	NEG	NEG	NEG	NEG	NEG	NEG	NEG	NEG	NEG	NEG	NEG	NEG	NEG	NEG	NEG	NEG	NEG	NEG	NEG	NEG	NEG	NEG	NEG	NEG	NEG	NEG	NEG	NEG	NEG	POS	POS	POS	NEG	NEG	NEG	NEG	NEG	NEG	NEG	NEG	NEG	NEG	NEG	NEG
NEG	NEG	NEG	NEG	NEG	NEG	NEG	NEG	NEG	NEG	NEG	NEG	NEG	NEG	NEG	NEG	NEG	NEG	NEG	NEG	NEG	NEG	NEG	NEG	NEG	NEG	NEG	NEG	NEG	NEG	NEG	NEG	NEG	NEG	NEG	NEG	NEG	NEG	NEG	NEG	NEG	NEG	NEG	NEG	NEG	NEG	NEG	NEG
NEG	NEG	NEG	NEG	NEG	NEG	NEG	NEG	NEG	NEG	NEG	NEG	NEG	NEG	NEG	NEG	NEG	NEG	NEG	NEG	NEG	NEG	NEG	NEG	NEG	NEG	NEG	NEG	NEG	NEG	NEG	NEG	NEG	POS	POS	POS	NEG	NEG	NEG	NEG	NEG	NEG	NEG	NEG	NEG	NEG	NEG	NEG
NEG	NEG	NEG	NEG	NEG	NEG	NEG	NEG	NEG	NEG	NEG	NEG	NEG	NEG	NEG	NEG	NEG	NEG	NEG	NEG	NEG	NEG	NEG	NEG	NEG	NEG	NEG	NEG	NEG	NEG	NEG	NEG	NEG	POS	POS	POS	NEG	NEG	NEG	NEG	NEG	NEG	NEG	NEG	NEG	NEG	NEG	NEG
NEG	NEG	NEG	NEG	NEG	NEG	NEG	NEG	NEG	NEG	NEG	NEG	NEG	NEG	NEG	NEG	NEG	NEG	NEG	NEG	NEG	NEG	NEG	NEG	NEG	NEG	NEG	NEG	NEG	NEG	NEG	NEG	NEG	POS	POS	AMB	NEG	NEG	NEG	NEG	NEG	NEG	NEG	NEG	NEG	NEG	NEG	NEG
NEG	NEG	NEG	NEG	NEG	NEG	NEG	NEG	NEG	NEG	NEG	NEG	NEG	NEG	NEG	NEG	NEG	NEG	NEG	NEG	NEG	NEG	NEG	NEG	NEG	NEG	NEG	NEG	NEG	NEG	NEG	NEG	NEG	POS	POS	POS	NEG	NEG	NEG	NEG	NEG	NEG	NEG	NEG	NEG	NEG	NEG	NEG
POS	POS	NEG	NEG	NEG	NEG	NEG	NEG	NEG	NEG	NEG	NEG	NEG	NEG	POS	NEG	NEG	NEG	NEG	NEG	NEG	NEG	NEG	NEG	NEG	NEG	POS	POS	NEG	NEG	NEG	NEG	NEG	POS	POS	POS	NEG	NEG	NEG	NEG	NEG	NEG	NEG	NEG	NEG	NEG	NEG	NEG
POS	POS	POS	NEG	NEG	NEG	NEG	NEG	NEG	NEG	NEG	NEG	NEG	NEG	NEG	NEG	NEG	NEG	NEG	NEG	NEG	NEG	POS	POS	NEG	NEG	NEG	NEG	NEG	NEG	POS	NEG	NEG	POS	POS	POS	NEG	NEG	NEG	NEG	NEG	NEG	NEG	NEG	NEG	NEG	NEG	NEG
NEG	NEG	NEG	NEG	NEG	NEG	NEG	NEG	NEG	NEG	NEG	NEG	NEG	NEG	NEG	NEG	NEG	NEG	NEG	NEG	NEG	NEG	NEG	NEG	NEG	NEG	NEG	NEG	NEG	NEG	NEG	NEG	NEG	POS	POS	POS	NEG	NEG	NEG	NEG	NEG	NEG	NEG	NEG	NEG	NEG	NEG	NEG
NEG	NEG	NEG	NEG	NEG	NEG	NEG	NEG	NEG	NEG	NEG	NEG	NEG	NEG	NEG	NEG	NEG	NEG	NEG	NEG	NEG	NEG	NEG	NEG	NEG	NEG	NEG	NEG	NEG	NEG	NEG	NEG	NEG	POS	POS	POS	NEG	NEG	NEG	NEG	NEG	NEG	NEG	NEG	NEG	NEG	NEG	NEG
NEG	NEG	NEG	NEG	NEG	NEG	NEG	NEG	NEG	NEG	NEG	NEG	NEG	NEG	NEG	NEG	NEG	NEG	NEG	NEG	NEG	NEG	NEG	NEG	NEG	NEG	NEG	NEG	NEG	NEG	NEG	NEG	NEG	POS	POS	POS	NEG	NEG	NEG	NEG	NEG	NEG	NEG	NEG	NEG	NEG	NEG	NEG
NEG	NEG	NEG	NEG	NEG	NEG	NEG	NEG	NEG	NEG	NEG	NEG	NEG	NEG	NEG	NEG	NEG	NEG	NEG	NEG	NEG	NEG	NEG	NEG	NEG	NEG	NEG	NEG	NEG	NEG	NEG	NEG	NEG	POS	POS	POS	NEG	NEG	NEG	NEG	NEG	NEG	NEG	NEG	NEG	NEG	NEG	NEG
NEG	NEG	NEG	NEG	NEG	NEG	NEG	NEG	NEG	NEG	NEG	NEG	NEG	NEG	NEG	NEG	NEG	NEG	NEG	NEG	NEG	NEG	NEG	NEG	NEG	NEG	NEG	NEG	NEG	NEG	NEG	NEG	NEG	NEG	NEG	NEG	NEG	NEG	NEG	NEG	NEG	NEG	NEG	NEG	NEG	NEG	NEG	NEG
NEG	NEG	NEG	NEG	NEG	NEG	NEG	NEG	NEG	NEG	NEG	NEG	NEG	NEG	NEG	NEG	NEG	NEG	NEG	NEG	NEG	NEG	NEG	NEG	NEG	NEG	NEG	NEG	NEG	NEG	NEG	NEG	NEG	POS	POS	POS	NEG	NEG	NEG	NEG	NEG	NEG	NEG	NEG	NEG	NEG	NEG	NEG
NEG	NEG	NEG	NEG	NEG	NEG	NEG	NEG	NEG	NEG	NEG	NEG	NEG	NEG	NEG	NEG	NEG	NEG	NEG	NEG	NEG	NEG	NEG	NEG	NEG	NEG	NEG	NEG	NEG	NEG	NEG	NEG	NEG	POS	POS	POS	NEG	NEG	NEG	NEG	NEG	NEG	NEG	NEG	NEG	NEG	NEG	NEG
NEG	NEG	NEG	NEG	NEG	NEG	NEG	NEG	NEG	NEG	NEG	NEG	NEG	NEG	NEG	NEG	NEG	NEG	NEG	NEG	NEG	NEG	NEG	NEG	NEG	NEG	NEG	NEG	NEG	NEG	NEG	NEG	NEG	POS	POS	POS	NEG	NEG	NEG	NEG	NEG	NEG	NEG	NEG	NEG	NEG	NEG	NEG
NEG	NEG	NEG	NEG	NEG	NEG	NEG	NEG	NEG	NEG	NEG	NEG	NEG	NEG	NEG	NEG	NEG	NEG	NEG	NEG	NEG	NEG	NEG	NEG	NEG	NEG	NEG	NEG	NEG	NEG	NEG	NEG	NEG	POS	POS	POS	NEG	NEG	NEG	NEG	NEG	NEG	NEG	NEG	NEG	NEG	NEG	NEG
NEG	NEG	NEG	NEG	NEG	NEG	NEG	NEG	NEG	NEG	NEG	NEG	NEG	NEG	NEG	NEG	NEG	NEG	NEG	NEG	NEG	NEG	NEG	NEG	NEG	NEG	NEG	NEG	NEG	NEG	NEG	NEG	NEG	NEG	NEG	NEG	NEG	NEG	NEG	NEG	NEG	NEG	NEG	NEG	NEG	NEG	NEG	NEG
NEG	NEG	NEG	NEG	NEG	NEG	NEG	NEG	NEG	NEG	NEG	NEG	NEG	NEG	NEG	NEG	NEG	NEG	NEG	NEG	NEG	NEG	NEG	NEG	NEG	NEG	NEG	NEG	NEG	NEG	NEG	NEG	NEG	NEG	NEG	NEG	NEG	NEG	NEG	NEG	NEG	NEG	NEG	NEG	NEG	NEG	NEG	NEG
NEG	NEG	NEG	NEG	NEG	NEG	NEG	NEG	NEG	NEG	NEG	NEG	NEG	NEG	NEG	NEG	NEG	NEG	NEG	NEG	NEG	NEG	NEG	NEG	NEG	NEG	NEG	NEG	NEG	NEG	NEG	NEG	NEG	NEG	NEG	NEG	NEG	NEG	NEG	NEG	NEG	NEG	NEG	NEG	NEG	NEG	NEG	NEG
NEG	NEG	NEG	NEG	NEG	NEG	NEG	NEG	NEG	NEG	NEG	NEG	NEG	NEG	NEG	NEG	NEG	NEG	NEG	NEG	NEG	NEG	NEG	NEG	NEG	NEG	NEG	NEG	NEG	NEG	NEG	NEG	NEG	POS	POS	POS	NEG	NEG	NEG	NEG	NEG	NEG	NEG	NEG	NEG	NEG	NEG	NEG
NEG	NEG	NEG	NEG	NEG	NEG	NEG	NEG	NEG	NEG	NEG	NEG	NEG	NEG	NEG	NEG	NEG	NEG	NEG	NEG	NEG	NEG	NEG	NEG	NEG	NEG	NEG	NEG	NEG	NEG	NEG	NEG	NEG	POS	POS	POS	NEG	NEG	NEG	NEG	NEG	NEG	NEG	NEG	NEG	NEG	NEG	NEG
NEG	NEG	NEG	NEG	NEG	NEG	NEG	NEG	NEG	NEG	NEG	NEG	NEG	NEG	NEG	NEG	NEG	NEG	NEG	NEG	NEG	NEG	NEG	NEG	NEG	NEG	NEG	NEG	NEG	NEG	NEG	NEG	NEG	POS	POS	POS	NEG	NEG	NEG	NEG	NEG	NEG	NEG	NEG	NEG	NEG	NEG	NEG
NEG	NEG	NEG	NEG	NEG	NEG	NEG	NEG	NEG	NEG	NEG	NEG	NEG	NEG	NEG	NEG	NEG	NEG	NEG	NEG	NEG	NEG	NEG	NEG	NEG	NEG	NEG	NEG	NEG	NEG	NEG	NEG	NEG	NEG	NEG	NEG	NEG	NEG	NEG	NEG	NEG	NEG	NEG	NEG	NEG	NEG	NEG	NEG
NEG	NEG	NEG	NEG	NEG	NEG	NEG	NEG	NEG	NEG	NEG	NEG	NEG	NEG	NEG	NEG	NEG	NEG	NEG	NEG	NEG	NEG	NEG	NEG	NEG	NEG	NEG	NEG	NEG	NEG	NEG	NEG	NEG	POS	POS	POS	NEG	NEG	NEG	AMB	POS	NEG	POS	NEG	NEG	NEG	NEG	NEG
NEG	NEG	NEG	NEG	NEG	NEG	NEG	NEG	NEG	NEG	NEG	NEG	NEG	NEG	NEG	NEG	NEG	NEG	NEG	NEG	NEG	NEG	NEG	NEG	NEG	NEG	NEG	NEG	NEG	NEG	NEG	NEG	NEG	NEG	NEG	NEG	NEG	NEG	NEG	NEG	NEG	NEG	NEG	NEG	NEG	NEG	NEG	NEG
NEG	NEG	NEG	NEG	NEG	NEG	NEG	NEG	NEG	NEG	NEG	NEG	NEG	NEG	NEG	NEG	NEG	NEG	NEG	NEG	NEG	NEG	NEG	NEG	NEG	NEG	NEG	NEG	NEG	NEG	NEG	NEG	NEG	POS	POS	POS	NEG	NEG	NEG	NEG	NEG	NEG	NEG	NEG	NEG	NEG	NEG	NEG
NEG	NEG	NEG	NEG	NEG	NEG	NEG	NEG	NEG	NEG	NEG	NEG	NEG	NEG	NEG	NEG	NEG	NEG	NEG	NEG	NEG	NEG	NEG	NEG	NEG	NEG	NEG	NEG	NEG	NEG	NEG	NEG	NEG	POS	POS	POS	NEG	NEG	NEG	NEG	NEG	NEG	NEG	NEG	NEG	NEG	NEG	NEG
NEG	NEG	NEG	NEG	NEG	NEG	NEG	NEG	NEG	NEG	NEG	NEG	NEG	NEG	NEG	NEG	NEG	NEG	NEG	NEG	NEG	NEG	NEG	NEG	NEG	NEG	NEG	NEG	NEG	NEG	NEG	NEG	NEG	POS	POS	POS	NEG	NEG	NEG	NEG	NEG	NEG	NEG	NEG	NEG	NEG	NEG	NEG
NEG	NEG	NEG	NEG	NEG	NEG	NEG	NEG	NEG	NEG	NEG	NEG	NEG	NEG	NEG	NEG	NEG	NEG	NEG	NEG	NEG	NEG	NEG	NEG	NEG	NEG	NEG	NEG	NEG	NEG	NEG	NEG	NEG	POS	POS	POS	NEG	NEG	NEG	NEG	NEG	NEG	NEG	NEG	NEG	NEG	NEG	NEG
NEG	NEG	NEG	NEG	NEG	NEG	NEG	NEG	NEG	NEG	NEG	NEG	NEG	NEG	NEG	NEG	NEG	NEG	NEG	NEG	NEG	NEG	NEG	NEG	NEG	NEG	NEG	NEG	NEG	NEG	NEG	NEG	NEG	POS	POS	POS	NEG	NEG	NEG	NEG	NEG	NEG	NEG	NEG	NEG	NEG	NEG	NEG
NEG	NEG	NEG	NEG	NEG	NEG	NEG	NEG	NEG	NEG	NEG	NEG	NEG	NEG	NEG	NEG	NEG	NEG	NEG	NEG	NEG	NEG	NEG	NEG	NEG	NEG	NEG	NEG	NEG	NEG	NEG	NEG	NEG	POS	POS	POS	NEG	NEG	NEG	NEG	NEG	NEG	NEG	NEG	NEG	NEG	NEG	NEG
NEG	NEG	NEG	NEG	NEG	NEG	NEG	NEG	NEG	NEG	NEG	NEG	NEG	NEG	NEG	NEG	NEG	NEG	NEG	NEG	NEG	NEG	NEG	NEG	NEG	NEG	NEG	NEG	NEG	NEG	NEG	NEG	NEG	POS	POS	POS	NEG	NEG	NEG	NEG	NEG	NEG	NEG	NEG	NEG	NEG	NEG	NEG
NEG	NEG	NEG	NEG	NEG	NEG	NEG	NEG	NEG	NEG	NEG	NEG	NEG	NEG	NEG	NEG	NEG	NEG	NEG	NEG	NEG	NEG	NEG	NEG	NEG	NEG	NEG	NEG	NEG	NEG	NEG	NEG	NEG	POS	POS	POS	NEG	NEG	NEG	NEG	NEG	NEG	NEG	NEG	NEG	NEG	NEG	NEG
NEG	NEG	NEG	NEG	NEG	NEG	NEG	NEG	NEG	NEG	NEG	NEG	NEG	NEG	NEG	NEG	NEG	NEG	NEG	NEG	NEG	NEG	NEG	NEG	NEG	NEG	NEG	NEG	NEG	NEG	NEG	NEG	NEG	POS	POS	POS	NEG	NEG	NEG	NEG	NEG	NEG	NEG	NEG	NEG	NEG	NEG	NEG
NEG	NEG	NEG	NEG	NEG	NEG	NEG	NEG	NEG	NEG	NEG	NEG	NEG	NEG	NEG	NEG	NEG	NEG	NEG	NEG	NEG	NEG	NEG	NEG	NEG	NEG	NEG	NEG	NEG	NEG	NEG	NEG	NEG	POS	POS	POS	NEG	NEG	NEG	NEG	NEG	NEG	NEG	NEG	NEG	NEG	NEG	NEG
NEG	NEG	NEG	NEG	NEG	NEG	NEG	NEG	NEG	NEG	NEG	NEG	NEG	NEG	NEG	NEG	NEG	NEG	NEG	NEG	NEG	NEG	NEG	NEG	NEG	NEG	NEG	NEG	NEG	NEG	NEG	NEG	NEG	NEG	NEG	NEG	NEG	NEG	NEG	NEG	NEG	NEG	NEG	NEG	NEG	NEG	NEG	NEG
NEG	NEG	NEG	NEG	NEG	NEG	NEG	NEG	NEG	NEG	NEG	NEG	NEG	NEG	NEG	NEG	NEG	NEG	NEG	NEG	NEG	NEG	NEG	NEG	NEG	NEG	NEG	NEG	NEG	NEG	NEG	NEG	NEG	POS	POS	POS	NEG	NEG	NEG	NEG	NEG	NEG	NEG	NEG	NEG	NEG	NEG	NEG
POS	POS	POS	POS	POS	POS	NEG	NEG	NEG	NEG	NEG	NEG	NEG	NEG	NEG	NEG	NEG	NEG	NEG	NEG	NEG	AMB	NEG	NEG	POS	POS	NEG	POS	NEG	NEG	NEG	POS	POS	POS	POS	POS	POS	NEG	NEG	NEG	NEG	NEG	NEG	NEG	NEG	NEG	NEG	NEG
POS	POS	POS	POS	POS	POS	NEG	NEG	NEG	NEG	NEG	NEG	NEG	NEG	NEG	NEG	NEG	NEG	NEG	NEG	NEG	AMB	NEG	NEG	POS	POS	NEG	POS	NEG	NEG	NEG	POS	POS	POS	POS	POS	POS	NEG	NEG	NEG	NEG	NEG	NEG	NEG	NEG	NEG	NEG	NEG
POS	POS	POS	POS	POS	POS	NEG	NEG	NEG	NEG	NEG	NEG	NEG	NEG	NEG	NEG	NEG	NEG	NEG	NEG	NEG	NEG	NEG	NEG	POS	POS	NEG	POS	NEG	POS	NEG	POS	POS	POS	POS	POS	POS	NEG	NEG	NEG	NEG	NEG	NEG	NEG	NEG	NEG	NEG	NEG
POS	POS	POS	POS	POS	POS	NEG	NEG	NEG	NEG	NEG	NEG	NEG	NEG	NEG	NEG	NEG	NEG	NEG	NEG	NEG	NEG	NEG	NEG	POS	POS	NEG	POS	NEG	POS	NEG	POS	POS	POS	POS	POS	POS	NEG	NEG	NEG	NEG	NEG	NEG	NEG	NEG	NEG	NEG	NEG
POS	POS	POS	POS	POS	POS	NEG	NEG	NEG	NEG	NEG	NEG	NEG	NEG	NEG	NEG	NEG	NEG	NEG	NEG	NEG	AMB	NEG	NEG	POS	POS	NEG	POS	NEG	POS	NEG	POS	POS	POS	POS	POS	POS	NEG	POS	NEG	NEG	NEG	NEG	NEG	NEG	NEG	NEG	NEG
POS	POS	POS	POS	POS	POS	NEG	NEG	NEG	NEG	NEG	NEG	NEG	NEG	NEG	NEG	NEG	NEG	NEG	NEG	NEG	AMB	NEG	NEG	POS	POS	NEG	POS	NEG	POS	NEG	NEG	NEG	POS	POS	POS	POS	NEG	NEG	NEG	NEG	NEG	NEG	NEG	NEG	NEG	NEG	NEG
POS	POS	POS	POS	POS	POS	NEG	NEG	NEG	NEG	NEG	NEG	NEG	NEG	NEG	NEG	NEG	NEG	NEG	NEG	NEG	AMB	NEG	NEG	POS	POS	NEG	POS	NEG	POS	NEG	POS	POS	POS	POS	POS	POS	NEG	NEG	NEG	NEG	NEG	NEG	NEG	NEG	NEG	NEG	NEG
NEG	NEG	NEG	NEG	NEG	NEG	NEG	NEG	NEG	NEG	NEG	NEG	NEG	NEG	NEG	NEG	NEG	NEG	NEG	NEG	NEG	NEG	NEG	NEG	NEG	NEG	NEG	NEG	NEG	NEG	NEG	NEG	NEG	POS	POS	POS	NEG	NEG	NEG	NEG	NEG	NEG	NEG	NEG	NEG	NEG	NEG	NEG
NEG	NEG	NEG	NEG	NEG	NEG	NEG	NEG	NEG	NEG	NEG	NEG	NEG	NEG	NEG	NEG	NEG	NEG	NEG	NEG	NEG	NEG	NEG	NEG	NEG	NEG	NEG	NEG	NEG	NEG	NEG	NEG	NEG	POS	POS	POS	NEG	NEG	NEG	NEG	NEG	NEG	NEG	NEG	NEG	NEG	NEG	NEG
NEG	NEG	NEG	NEG	NEG	NEG	NEG	NEG	NEG	NEG	NEG	NEG	NEG	NEG	NEG	NEG	NEG	NEG	NEG	NEG	NEG	NEG	NEG	NEG	NEG	NEG	NEG	NEG	NEG	NEG	NEG	NEG	NEG	POS	POS	POS	NEG	NEG	NEG	NEG	NEG	NEG	NEG	NEG	NEG	NEG	NEG	NEG
NEG	NEG	NEG	NEG	NEG	NEG	NEG	POS	NEG	NEG	NEG	NEG	NEG	NEG	NEG	NEG	NEG	NEG	NEG	NEG	NEG	NEG	NEG	NEG	NEG	NEG	NEG	NEG	NEG	NEG	NEG	NEG	NEG	POS	POS	POS	NEG	NEG	NEG	NEG	NEG	NEG	NEG	NEG	NEG	NEG	NEG	NEG
NEG	NEG	NEG	NEG	NEG	NEG	NEG	NEG	NEG	NEG	NEG	NEG	NEG	NEG	NEG	NEG	NEG	NEG	NEG	NEG	NEG	NEG	NEG	NEG	NEG	NEG	NEG	NEG	NEG	NEG	NEG	NEG	NEG	POS	POS	POS	NEG	NEG	NEG	NEG	NEG	NEG	NEG	NEG	NEG	NEG	NEG	NEG
NEG	NEG	NEG	NEG	NEG	NEG	NEG	NEG	NEG	NEG	NEG	NEG	NEG	NEG	NEG	NEG	NEG	NEG	NEG	NEG	NEG	NEG	NEG	NEG	NEG	NEG	NEG	NEG	NEG	NEG	NEG	NEG	NEG	NEG	NEG	NEG	NEG	NEG	NEG	NEG	NEG	NEG	NEG	NEG	NEG	NEG	NEG	NEG
NEG	NEG	NEG	NEG	NEG	NEG	NEG	NEG	NEG	NEG	NEG	NEG	NEG	NEG	NEG	NEG	NEG	NEG	NEG	NEG	NEG	NEG	NEG	NEG	NEG	NEG	NEG	NEG	NEG	NEG	NEG	NEG	NEG	POS	POS	POS	NEG	NEG	NEG	NEG	NEG	NEG	NEG	NEG	NEG	NEG	NEG	NEG

RESISTANCE : AMINOGLYSOSIDES			RESISTANCE : MISCELLANEOUS GENES																									VIRULENCE : SUPERANTIGENS																					
									cat							fosB			qacC									tst1	sea													egc							
aacA-aphD	aadD	aphA3	sat	dfrA	far1	mupA	tetK	tetM	cat	cat (pC221)	cat (pc223)	cat (pMC524)	cat (pSBK203R)	cfr	fexA	fosB	fosB (plasmid)	qacA	qacC	qacC (cons)	qacC (equine)	qacC (SAS)	qacC (Ssap)	qacC (ST94)	vanA	vanB	vanZ	tst1 (consensus)	entA	entA (320E)	entA (N315) / entP	seB	seC	seD	seE	seH	seJ	seK	seL	seQ	seR	egc (total)	seg	sei	selm	seln (con-sensus)	selo	selu	ORF CM14
bifunctional enzyme Aac/Aph, gentamicin resistance	amino-glycoside adenyl-transferase,to bramycin resistance	3'5''-aminog-lycoside phospho-transferase, neo-/ kanamycin resistance	strepto-thricine-acetyl-transferase	dihydro-folate reductase type 1	fusidic acid resistence	mupirocin resistence protein	tetracycline resistance	tetracycline resistance	chloramphenicol acetyltransferase					23S rRNA methyltransferase	chloramphenicol/florfenicol exporter	metallothiol transferase		quaternary ammonium compound resistance protein A	quaternary ammonium compound resistance protein C						vancomycin resistance gene	vancomycin resistance gene from enterococci and Clostridium	teicoplanin resistance gene from enterococci	toxic shock syndrome toxin 1	Enterotoxin A		Enterotoxin A, allele from strain N315 = Entero-toxin P	Enterotoxin B	Enterotoxin C	Enterotoxin D	Enterotoxin E	Enterotoxin H	Enterotoxin J	Enterotoxin K	Enterotoxin L	Enterotoxin Q	Enterotoxin R	egc cluster	Enterotoxin G	Enterotoxin I	Enterotoxin M	Enterotoxin N	Enterotoxin O	Enterotoxin U and/or Y	Enterotoxin-like protein ORF CM14
NEG	NEG	NEG	NEG	NEG	NEG	NEG	NEG	NEG	NEG	NEG	NEG	NEG	NEG	NEG	NEG	NEG	NEG	NEG	NEG	NEG	NEG	NEG	NEG	NEG	NEG	NEG	NEG	POS	POS	NEG	NEG	NEG	POS	NEG	NEG	POS	NEG	POS	POS	POS	NEG	NEG	NEG	NEG	NEG	NEG	NEG	NEG	NEG
NEG	NEG	NEG	NEG	NEG	NEG	NEG	NEG	NEG	NEG	NEG	NEG	NEG	NEG	NEG	NEG	NEG	NEG	NEG	NEG	NEG	NEG	NEG	NEG	NEG	NEG	NEG	NEG	POS	POS	NEG	NEG	NEG	POS	NEG	NEG	POS	NEG	POS	POS	POS	NEG	NEG	NEG	NEG	NEG	NEG	NEG	NEG	NEG
NEG	NEG	NEG	NEG	NEG	NEG	NEG	NEG	NEG	NEG	NEG	NEG	NEG	NEG	NEG	NEG	NEG	NEG	NEG	NEG	NEG	NEG	NEG	NEG	NEG	NEG	NEG	NEG	POS	POS	NEG	NEG	NEG	POS	NEG	NEG	POS	NEG	POS	POS	POS	NEG	NEG	NEG	NEG	NEG	NEG	NEG	NEG	NEG
NEG	NEG	NEG	NEG	NEG	NEG	NEG	NEG	NEG	NEG	NEG	NEG	NEG	NEG	NEG	NEG	NEG	NEG	NEG	NEG	NEG	NEG	NEG	NEG	NEG	NEG	NEG	NEG	POS	POS	NEG	NEG	NEG	POS	NEG	NEG	POS	NEG	POS	POS	POS	NEG	NEG	NEG	NEG	NEG	NEG	NEG	NEG	NEG
NEG	NEG	NEG	NEG	NEG	NEG	NEG	NEG	NEG	NEG	NEG	NEG	NEG	NEG	NEG	NEG	NEG	NEG	NEG	NEG	NEG	NEG	NEG	NEG	NEG	NEG	NEG	NEG	NEG	POS	NEG	NEG	NEG	NEG	NEG	NEG	POS	NEG	POS	NEG	POS	NEG	NEG	NEG	NEG	NEG	NEG	NEG	NEG	NEG
NEG	NEG	NEG	NEG	NEG	NEG	NEG	NEG	NEG	NEG	NEG	NEG	NEG	NEG	NEG	NEG	POS	NEG	NEG	NEG	NEG	NEG	NEG	NEG	NEG	NEG	NEG	NEG	NEG	NEG	NEG	NEG	NEG	NEG	NEG	NEG	NEG	NEG	NEG	NEG	NEG	NEG	POS	POS	POS	POS	POS	NEG	POS	NEG
NEG	NEG	NEG	NEG	NEG	NEG	NEG	NEG	NEG	NEG	NEG	NEG	NEG	NEG	NEG	NEG	POS	NEG	NEG	NEG	NEG	NEG	NEG	NEG	NEG	NEG	NEG	NEG	NEG	NEG	NEG	NEG	NEG	NEG	NEG	NEG	NEG	NEG	NEG	NEG	NEG	NEG	POS	POS	POS	POS	POS	POS	POS	NEG
NEG	NEG	NEG	NEG	NEG	NEG	NEG	NEG	NEG	NEG	NEG	NEG	NEG	NEG	NEG	NEG	POS	NEG	NEG	NEG	NEG	NEG	NEG	NEG	NEG	NEG	NEG	NEG	NEG	NEG	NEG	NEG	NEG	NEG	NEG	NEG	NEG	NEG	NEG	NEG	NEG	NEG	POS	POS	POS	POS	POS	POS	POS	NEG
NEG	NEG	NEG	NEG	NEG	NEG	NEG	NEG	NEG	NEG	NEG	NEG	NEG	NEG	NEG	NEG	POS	NEG	NEG	NEG	NEG	NEG	NEG	NEG	NEG	NEG	NEG	NEG	NEG	NEG	NEG	NEG	NEG	NEG	NEG	NEG	NEG	NEG	NEG	NEG	NEG	NEG	NEG	NEG	NEG	NEG	NEG	NEG	NEG	NEG
NEG	NEG	NEG	NEG	NEG	NEG	NEG	NEG	NEG	NEG	NEG	NEG	NEG	NEG	NEG	NEG	POS	NEG	NEG	NEG	NEG	NEG	NEG	NEG	NEG	NEG	NEG	NEG	NEG	NEG	NEG	NEG	NEG	NEG	NEG	NEG	NEG	NEG	NEG	NEG	NEG	NEG	NEG	NEG	NEG	NEG	NEG	NEG	NEG	NEG
NEG	NEG	NEG	NEG	NEG	NEG	NEG	NEG	NEG	NEG	NEG	NEG	NEG	NEG	NEG	NEG	POS	NEG	NEG	NEG	NEG	NEG	NEG	NEG	NEG	NEG	NEG	NEG	NEG	POS	NEG	NEG	NEG	NEG	NEG	NEG	NEG	NEG	NEG	NEG	NEG	NEG	NEG	NEG	NEG	NEG	NEG	NEG	NEG	NEG
NEG	NEG	NEG	NEG	NEG	NEG	NEG	NEG	NEG	NEG	NEG	NEG	NEG	NEG	NEG	NEG	POS	NEG	NEG	NEG	NEG	NEG	NEG	NEG	NEG	NEG	NEG	NEG	NEG	NEG	NEG	NEG	NEG	NEG	NEG	NEG	NEG	NEG	NEG	NEG	NEG	NEG	NEG	NEG	NEG	NEG	NEG	NEG	NEG	NEG
NEG	NEG	NEG	NEG	NEG	NEG	NEG	NEG	NEG	NEG	NEG	NEG	NEG	NEG	NEG	NEG	POS	NEG	NEG	NEG	NEG	NEG	NEG	NEG	NEG	NEG	NEG	NEG	NEG	NEG	NEG	NEG	NEG	NEG	NEG	NEG	NEG	NEG	NEG	NEG	NEG	NEG	NEG	NEG	NEG	NEG	NEG	NEG	NEG	NEG
NEG	NEG	NEG	NEG	NEG	NEG	NEG	NEG	NEG	NEG	NEG	NEG	NEG	NEG	NEG	NEG	POS	NEG	NEG	NEG	NEG	NEG	NEG	NEG	NEG	NEG	NEG	NEG	NEG	NEG	NEG	NEG	NEG	NEG	NEG	NEG	NEG	NEG	NEG	NEG	NEG	NEG	NEG	NEG	NEG	NEG	NEG	NEG	NEG	NEG
NEG	NEG	NEG	NEG	NEG	NEG	NEG	NEG	NEG	NEG	NEG	NEG	NEG	NEG	NEG	NEG	POS	NEG	NEG	NEG	NEG	NEG	NEG	NEG	NEG	NEG	NEG	NEG	NEG	NEG	NEG	NEG	NEG	NEG	NEG	NEG	NEG	NEG	NEG	NEG	NEG	NEG	NEG	NEG	NEG	NEG	NEG	NEG	NEG	NEG
NEG	NEG	NEG	NEG	NEG	NEG	NEG	NEG	NEG	NEG	NEG	NEG	NEG	NEG	NEG	NEG	POS	NEG	NEG	NEG	NEG	NEG	NEG	NEG	NEG	NEG	NEG	NEG	NEG	POS	NEG	NEG	NEG	NEG	NEG	NEG	NEG	NEG	NEG	NEG	NEG	NEG	NEG	NEG	NEG	NEG	NEG	NEG	NEG	NEG
NEG	NEG	NEG	NEG	NEG	NEG	NEG	NEG	NEG	NEG	NEG	NEG	NEG	NEG	NEG	NEG	POS	NEG	NEG	NEG	NEG	NEG	NEG	NEG	NEG	NEG	NEG	NEG	NEG	POS	NEG	NEG	NEG	NEG	NEG	NEG	NEG	NEG	NEG	NEG	NEG	NEG	NEG	NEG	NEG	NEG	NEG	NEG	NEG	NEG
NEG	NEG	NEG	NEG	NEG	NEG	NEG	NEG	NEG	NEG	NEG	NEG	NEG	NEG	NEG	NEG	POS	NEG	NEG	NEG	NEG	NEG	NEG	NEG	NEG	NEG	NEG	NEG	NEG	POS	NEG	NEG	NEG	NEG	NEG	NEG	NEG	NEG	NEG	NEG	NEG	NEG	NEG	NEG	NEG	NEG	NEG	NEG	NEG	NEG
NEG	NEG	NEG	NEG	NEG	NEG	NEG	NEG	NEG	NEG	NEG	NEG	NEG	NEG	NEG	NEG	POS	NEG	NEG	NEG	NEG	NEG	NEG	NEG	NEG	NEG	NEG	NEG	NEG	POS	NEG	NEG	NEG	NEG	NEG	NEG	NEG	NEG	NEG	NEG	NEG	NEG	NEG	NEG	NEG	NEG	NEG	NEG	NEG	NEG
NEG	NEG	NEG	NEG	NEG	NEG	NEG	NEG	NEG	NEG	NEG	NEG	NEG	NEG	NEG	NEG	NEG	NEG	NEG	NEG	NEG	NEG	NEG	NEG	NEG	NEG	NEG	NEG	NEG	NEG	NEG	POS	NEG	NEG	NEG	NEG	NEG	NEG	NEG	NEG	NEG	NEG	NEG	NEG	NEG	NEG	NEG	NEG	NEG	NEG
NEG	NEG	NEG	NEG	NEG	NEG	NEG	NEG	NEG	NEG	NEG	NEG	NEG	NEG	NEG	NEG	NEG	NEG	NEG	NEG	NEG	NEG	NEG	NEG	NEG	NEG	NEG	NEG	NEG	NEG	NEG	POS	NEG	NEG	NEG	NEG	NEG	NEG	NEG	NEG	NEG	NEG	NEG	NEG	NEG	NEG	NEG	NEG	NEG	NEG
NEG	POS	NEG	NEG	NEG	NEG	POS	NEG	NEG	NEG	NEG	NEG	NEG	NEG	NEG	NEG	NEG	NEG	NEG	NEG	NEG	NEG	NEG	NEG	NEG	NEG	NEG	NEG	NEG	NEG	NEG	POS	NEG	NEG	NEG	NEG	NEG	NEG	NEG	NEG	NEG	NEG	NEG	NEG	NEG	NEG	NEG	NEG	NEG	NEG
NEG	NEG	NEG	NEG	NEG	NEG	NEG	NEG	NEG	NEG	NEG	NEG	NEG	NEG	NEG	NEG	NEG	NEG	NEG	POS	POS	NEG	NEG	NEG	NEG	NEG	NEG	NEG	NEG	NEG	NEG	POS	NEG	NEG	NEG	NEG	NEG	NEG	NEG	NEG	NEG	NEG	NEG	NEG	NEG	NEG	NEG	NEG	NEG	NEG
NEG	NEG	NEG	NEG	NEG	NEG	NEG	NEG	NEG	NEG	NEG	NEG	NEG	NEG	NEG	NEG	NEG	NEG	NEG	NEG	NEG	NEG	NEG	NEG	NEG	NEG	NEG	NEG	NEG	NEG	NEG	POS	NEG	NEG	NEG	NEG	NEG	NEG	NEG	NEG	NEG	NEG	NEG	NEG	NEG	NEG	NEG	NEG	NEG	NEG
NEG	NEG	NEG	NEG	NEG	NEG	NEG	NEG	NEG	NEG	NEG	NEG	NEG	NEG	NEG	NEG	POS	NEG	NEG	POS	NEG	NEG	POS	NEG	NEG	NEG	NEG	NEG	NEG	NEG	NEG	NEG	NEG	NEG	NEG	NEG	NEG	NEG	NEG	NEG	NEG	NEG	NEG	NEG	NEG	NEG	NEG	NEG	NEG	NEG
NEG	NEG	NEG	NEG	NEG	NEG	NEG	NEG	NEG	NEG	NEG	NEG	NEG	NEG	NEG	NEG	POS	NEG	NEG	POS	NEG	NEG	POS	NEG	NEG	NEG	NEG	NEG	NEG	NEG	NEG	NEG	NEG	NEG	NEG	NEG	NEG	NEG	NEG	NEG	NEG	NEG	NEG	NEG	NEG	NEG	NEG	NEG	NEG	NEG
NEG	NEG	NEG	NEG	NEG	NEG	NEG	NEG	NEG	NEG	NEG	NEG	NEG	NEG	NEG	NEG	POS	NEG	NEG	NEG	NEG	NEG	NEG	NEG	NEG	NEG	NEG	NEG	NEG	NEG	NEG	NEG	NEG	NEG	POS	NEG	NEG	POS	POS	NEG	POS	POS	NEG	NEG	NEG	NEG	NEG	NEG	NEG	NEG
NEG	NEG	NEG	NEG	NEG	NEG	NEG	NEG	NEG	NEG	NEG	NEG	NEG	NEG	NEG	NEG	POS	NEG	NEG	NEG	NEG	NEG	NEG	NEG	NEG	NEG	NEG	NEG	NEG	NEG	NEG	NEG	NEG	NEG	POS	NEG	NEG	POS	POS	NEG	POS	POS	NEG	NEG	NEG	NEG	NEG	NEG	NEG	NEG
NEG	NEG	NEG	NEG	NEG	NEG	NEG	NEG	NEG	NEG	NEG	NEG	NEG	NEG	NEG	NEG	POS	NEG	NEG	NEG	NEG	NEG	NEG	NEG	NEG	NEG	NEG	NEG	NEG	NEG	NEG	NEG	NEG	NEG	POS	NEG	NEG	POS	POS	NEG	POS	POS	NEG	NEG	NEG	NEG	NEG	NEG	NEG	NEG
NEG	NEG	NEG	NEG	NEG	NEG	NEG	NEG	NEG	NEG	NEG	NEG	NEG	NEG	NEG	NEG	POS	NEG	NEG	NEG	NEG	NEG	NEG	NEG	NEG	NEG	NEG	NEG	NEG	NEG	NEG	NEG	NEG	NEG	NEG	NEG	NEG	NEG	POS	NEG	POS	NEG	NEG	NEG	NEG	NEG	NEG	NEG	NEG	NEG
NEG	NEG	NEG	NEG	NEG	NEG	NEG	NEG	NEG	NEG	NEG	NEG	NEG	NEG	NEG	NEG	POS	NEG	NEG	NEG	NEG	NEG	NEG	NEG	NEG	NEG	NEG	NEG	NEG	NEG	NEG	NEG	NEG	NEG	POS	NEG	NEG	POS	POS	NEG	POS	POS	NEG	NEG	NEG	NEG	NEG	NEG	NEG	NEG
NEG	NEG	NEG	NEG	NEG	NEG	NEG	NEG	NEG	NEG	NEG	NEG	NEG	NEG	NEG	NEG	POS	NEG	NEG	NEG	NEG	NEG	NEG	NEG	NEG	NEG	NEG	NEG	NEG	NEG	NEG	NEG	NEG	NEG	POS	NEG	NEG	POS	POS	NEG	POS	POS	NEG	NEG	NEG	NEG	NEG	NEG	NEG	NEG
NEG	NEG	NEG	NEG	NEG	NEG	NEG	NEG	NEG	NEG	NEG	NEG	NEG	NEG	NEG	NEG	POS	NEG	NEG	NEG	NEG	NEG	NEG	NEG	NEG	NEG	NEG	NEG	NEG	NEG	NEG	NEG	NEG	NEG	POS	NEG	NEG	POS	POS	NEG	POS	POS	NEG	NEG	NEG	NEG	NEG	NEG	NEG	NEG
NEG	NEG	NEG	NEG	NEG	NEG	NEG	NEG	NEG	NEG	NEG	NEG	NEG	NEG	NEG	NEG	POS	NEG	NEG	NEG	NEG	NEG	NEG	NEG	NEG	NEG	NEG	NEG	NEG	NEG	NEG	NEG	NEG	NEG	POS	NEG	NEG	POS	POS	NEG	POS	POS	NEG	NEG	NEG	NEG	NEG	NEG	NEG	NEG
NEG	NEG	NEG	NEG	NEG	NEG	NEG	NEG	NEG	NEG	NEG	NEG	NEG	NEG	NEG	NEG	POS	NEG	NEG	NEG	NEG	NEG	NEG	NEG	NEG	NEG	NEG	NEG	NEG	NEG	NEG	NEG	NEG	NEG	POS	NEG	NEG	POS	POS	NEG	POS	POS	NEG	NEG	NEG	NEG	NEG	NEG	NEG	NEG
NEG	NEG	NEG	NEG	NEG	NEG	NEG	NEG	NEG	NEG	NEG	NEG	NEG	NEG	NEG	NEG	POS	NEG	NEG	NEG	NEG	NEG	NEG	NEG	NEG	NEG	NEG	NEG	NEG	NEG	NEG	NEG	NEG	NEG	POS	NEG	NEG	POS	POS	NEG	POS	POS	NEG	NEG	NEG	NEG	NEG	NEG	NEG	NEG
NEG	NEG	NEG	NEG	NEG	NEG	NEG	NEG	NEG	NEG	NEG	NEG	NEG	NEG	NEG	NEG	POS	NEG	NEG	NEG	NEG	NEG	NEG	NEG	NEG	NEG	NEG	NEG	NEG	NEG	NEG	NEG	NEG	NEG	POS	NEG	NEG	POS	POS	NEG	POS	POS	NEG	NEG	NEG	NEG	NEG	NEG	NEG	NEG
NEG	NEG	NEG	NEG	NEG	NEG	POS	NEG	NEG	NEG	NEG	NEG	NEG	NEG	NEG	NEG	POS	NEG	NEG	NEG	NEG	NEG	NEG	NEG	NEG	NEG	NEG	NEG	NEG	NEG	NEG	NEG	NEG	NEG	POS	NEG	NEG	POS	POS	NEG	POS	POS	NEG	NEG	NEG	NEG	NEG	NEG	NEG	NEG
NEG	NEG	NEG	NEG	NEG	NEG	NEG	NEG	NEG	NEG	NEG	NEG	NEG	NEG	NEG	NEG	POS	NEG	NEG	NEG	NEG	NEG	NEG	NEG	NEG	NEG	NEG	NEG	NEG	NEG	NEG	NEG	NEG	NEG	POS	NEG	NEG	POS	POS	NEG	POS	NEG	NEG	NEG	NEG	NEG	NEG	NEG	NEG	NEG
NEG	NEG	POS	POS	NEG	NEG	NEG	NEG	NEG	NEG	NEG	NEG	NEG	NEG	NEG	NEG	POS	NEG	NEG	NEG	NEG	NEG	NEG	NEG	NEG	NEG	NEG	NEG	NEG	NEG	NEG	NEG	NEG	NEG	NEG	NEG	NEG	NEG	POS	NEG	POS	NEG	NEG	NEG	NEG	NEG	NEG	NEG	NEG	NEG
NEG	NEG	NEG	NEG	NEG	NEG	NEG	NEG	NEG	NEG	NEG	NEG	NEG	NEG	NEG	NEG	POS	NEG	NEG	NEG	NEG	NEG	NEG	NEG	NEG	NEG	NEG	NEG	NEG	NEG	NEG	NEG	POS	NEG	NEG	NEG	NEG	NEG	NEG	NEG	NEG	NEG	POS	POS	POS	POS	POS	POS	POS	NEG
NEG	NEG	NEG	NEG	NEG	NEG	NEG	NEG	NEG	NEG	NEG	NEG	NEG	NEG	NEG	NEG	POS	NEG	NEG	NEG	NEG	NEG	NEG	NEG	NEG	NEG	NEG	NEG	NEG	NEG	NEG	POS	NEG	NEG	NEG	NEG	NEG	NEG	NEG	NEG	NEG	NEG	NEG	NEG	NEG	NEG	NEG	NEG	NEG	POS
NEG	NEG	NEG	NEG	NEG	NEG	NEG	NEG	NEG	NEG	NEG	NEG	NEG	NEG	NEG	NEG	POS	NEG	NEG	NEG	NEG	NEG	NEG	NEG	NEG	NEG	NEG	NEG	NEG	NEG	NEG	NEG	NEG	NEG	NEG	NEG	NEG	NEG	NEG	NEG	NEG	NEG	NEG	NEG	NEG	NEG	NEG	NEG	NEG	NEG
NEG	NEG	NEG	NEG	NEG	NEG	NEG	NEG	NEG	NEG	NEG	NEG	NEG	NEG	NEG	NEG	POS	NEG	NEG	NEG	NEG	NEG	NEG	NEG	NEG	NEG	NEG	NEG	NEG	NEG	NEG	NEG	NEG	POS	NEG	NEG	NEG	NEG	NEG	POS	NEG	NEG	NEG	NEG	NEG	NEG	NEG	NEG	NEG	NEG
NEG	NEG	NEG	NEG	NEG	NEG	NEG	NEG	NEG	NEG	NEG	NEG	NEG	NEG	NEG	NEG	POS	NEG	NEG	NEG	NEG	NEG	NEG	NEG	NEG	NEG	NEG	NEG	NEG	NEG	NEG	NEG	NEG	POS	NEG	NEG	NEG	NEG	NEG	POS	NEG	NEG	NEG	NEG	NEG	NEG	NEG	NEG	NEG	NEG
POS	NEG	NEG	NEG	POS	NEG	NEG	NEG	NEG	NEG	NEG	NEG	NEG	NEG	NEG	NEG	NEG	NEG	NEG	NEG	NEG	NEG	NEG	NEG	NEG	NEG	NEG	NEG	POS	NEG	NEG	NEG	NEG	POS	NEG	NEG	NEG	NEG	NEG	POS	NEG	NEG	POS	POS	POS	POS	POS	POS	POS	NEG
NEG	NEG	NEG	NEG	NEG	NEG	NEG	NEG	NEG	NEG	NEG	NEG	NEG	NEG	NEG	NEG	POS	NEG	NEG	NEG	NEG	NEG	NEG	NEG	NEG	NEG	NEG	NEG	NEG	NEG	NEG	NEG	NEG	NEG	NEG	NEG	NEG	NEG	NEG	NEG	NEG	NEG	POS	POS	POS	POS	POS	POS	POS	NEG
NEG	NEG	NEG	NEG	NEG	NEG	NEG	NEG	NEG	NEG	NEG	NEG	NEG	NEG	NEG	NEG	NEG	NEG	NEG	NEG	NEG	NEG	NEG	NEG	NEG	NEG	NEG	NEG	NEG	NEG	NEG	NEG	NEG	NEG	NEG	NEG	NEG	NEG	NEG	NEG	NEG	NEG	POS	POS	POS	POS	POS	POS	POS	NEG
NEG	NEG	NEG	NEG	NEG	NEG	NEG	NEG	NEG	NEG	NEG	NEG	NEG	NEG	NEG	NEG	NEG	NEG	NEG	NEG	NEG	NEG	NEG	NEG	NEG	NEG	NEG	NEG	NEG	NEG	NEG	NEG	NEG	NEG	NEG	NEG	NEG	NEG	NEG	NEG	NEG	NEG	POS	POS	POS	POS	POS	POS	POS	NEG
NEG	NEG	NEG	NEG	NEG	NEG	NEG	NEG	NEG	NEG	NEG	NEG	NEG	NEG	NEG	NEG	NEG	NEG	NEG	NEG	NEG	NEG	NEG	NEG	NEG	NEG	NEG	NEG	NEG	NEG	NEG	NEG	NEG	NEG	NEG	NEG	NEG	NEG	NEG	NEG	NEG	NEG	POS	POS	POS	POS	POS	POS	POS	NEG
NEG	NEG	NEG	NEG	NEG	NEG	NEG	NEG	NEG	NEG	NEG	NEG	NEG	NEG	NEG	NEG	NEG	NEG	NEG	NEG	NEG	NEG	NEG	NEG	NEG	NEG	NEG	NEG	NEG	NEG	NEG	NEG	NEG	NEG	NEG	NEG	NEG	NEG	NEG	NEG	NEG	NEG	POS	POS	POS	POS	POS	POS	POS	NEG
NEG	NEG	NEG	NEG	NEG	NEG	NEG	NEG	NEG	NEG	NEG	NEG	NEG	NEG	NEG	NEG	NEG	NEG	NEG	NEG	NEG	NEG	NEG	NEG	NEG	NEG	NEG	NEG	NEG	NEG	NEG	NEG	POS	NEG	NEG	NEG	NEG	NEG	POS	NEG	POS	NEG	NEG	NEG	NEG	NEG	NEG	NEG	NEG	NEG
NEG	NEG	NEG	NEG	NEG	NEG	NEG	NEG	NEG	NEG	NEG	NEG	NEG	NEG	NEG	NEG	POS	NEG	NEG	NEG	NEG	NEG	NEG	NEG	NEG	NEG	NEG	NEG	NEG	NEG	NEG	NEG	NEG	POS	NEG	NEG	NEG	NEG	NEG	POS	NEG	NEG	POS	POS	POS	POS	POS	POS	POS	NEG
NEG	NEG	NEG	NEG	NEG	NEG	NEG	NEG	NEG	NEG	NEG	NEG	NEG	NEG	NEG	NEG	POS	NEG	NEG	NEG	NEG	NEG	NEG	NEG	NEG	NEG	NEG	NEG	NEG	NEG	NEG	NEG	NEG	POS	NEG	NEG	NEG	NEG	NEG	POS	NEG	NEG	POS	POS	POS	POS	POS	POS	POS	NEG
NEG	NEG	NEG	NEG	NEG	NEG	NEG	NEG	NEG	NEG	NEG	NEG	NEG	NEG	NEG	NEG	POS	NEG	NEG	NEG	NEG	NEG	NEG	NEG	NEG	NEG	NEG	NEG	NEG	NEG	NEG	NEG	NEG	POS	NEG	NEG	NEG	NEG	NEG	NEG	NEG	NEG	POS	POS	POS	POS	POS	POS	POS	NEG
NEG	NEG	NEG	NEG	NEG	NEG	NEG	NEG	NEG	NEG	NEG	NEG	NEG	NEG	NEG	NEG	POS	NEG	NEG	NEG	NEG	NEG	NEG	NEG	NEG	NEG	NEG	NEG	NEG	NEG	NEG	NEG	NEG	POS	NEG	NEG	NEG	NEG	NEG	POS	NEG	NEG	POS	POS	POS	POS	POS	POS	POS	NEG
NEG	NEG	NEG	NEG	NEG	NEG	NEG	POS	NEG	NEG	NEG	NEG	NEG	NEG	NEG	NEG	POS	NEG	NEG	NEG	NEG	NEG	NEG	NEG	NEG	NEG	NEG	NEG	NEG	NEG	NEG	NEG	NEG	POS	NEG	NEG	NEG	NEG	NEG	POS	NEG	NEG	POS	POS	POS	POS	POS	POS	POS	NEG
NEG	NEG	NEG	NEG	NEG	NEG	NEG	NEG	NEG	NEG	NEG	NEG	NEG	NEG	NEG	NEG	NEG	NEG	NEG	NEG	NEG	NEG	NEG	NEG	NEG	NEG	NEG	NEG	NEG	NEG	NEG	NEG	NEG	NEG	NEG	NEG	NEG	NEG	NEG	NEG	NEG	NEG	NEG	NEG	NEG	NEG	NEG	NEG	NEG	NEG
NEG	NEG	NEG	NEG	NEG	NEG	NEG	NEG	NEG	NEG	NEG	NEG	NEG	NEG	NEG	NEG	NEG	NEG	NEG	NEG	NEG	NEG	NEG	NEG	NEG	NEG	NEG	NEG	NEG	NEG	NEG	NEG	NEG	NEG	NEG	NEG	NEG	NEG	NEG	NEG	NEG	NEG	NEG	NEG	NEG	NEG	NEG	NEG	NEG	NEG
NEG	NEG	NEG	NEG	NEG	NEG	NEG	NEG	NEG	NEG	NEG	NEG	NEG	NEG	NEG	NEG	NEG	NEG	NEG	NEG	NEG	NEG	NEG	NEG	NEG	NEG	NEG	NEG	NEG	NEG	NEG	NEG	NEG	NEG	NEG	NEG	NEG	NEG	NEG	NEG	NEG	NEG	NEG	NEG	NEG	NEG	NEG	NEG	NEG	NEG
NEG	NEG	NEG	NEG	NEG	NEG	NEG	NEG	NEG	NEG	NEG	NEG	NEG	NEG	NEG	NEG	NEG	NEG	NEG	NEG	NEG	NEG	NEG	NEG	NEG	NEG	NEG	NEG	NEG	NEG	NEG	NEG	NEG	NEG	NEG	NEG	NEG	NEG	NEG	NEG	NEG	NEG	NEG	NEG	NEG	NEG	NEG	NEG	NEG	NEG
NEG	NEG	NEG	NEG	NEG	NEG	NEG	NEG	NEG	NEG	NEG	NEG	NEG	NEG	NEG	NEG	NEG	NEG	NEG	NEG	NEG	NEG	NEG	NEG	NEG	NEG	NEG	NEG	NEG	NEG	NEG	NEG	NEG	NEG	NEG	NEG	NEG	NEG	NEG	NEG	NEG	NEG	NEG	NEG	NEG	NEG	NEG	NEG	NEG	NEG
NEG	NEG	NEG	NEG	NEG	NEG	NEG	NEG	NEG	NEG	NEG	NEG	NEG	NEG	NEG	NEG	NEG	NEG	NEG	NEG	NEG	NEG	NEG	NEG	NEG	NEG	NEG	NEG	NEG	NEG	NEG	NEG	NEG	NEG	NEG	NEG	NEG	NEG	NEG	NEG	NEG	NEG	NEG	NEG	NEG	NEG	NEG	NEG	NEG	NEG
NEG	NEG	NEG	NEG	NEG	NEG	NEG	NEG	NEG	NEG	NEG	NEG	NEG	NEG	NEG	NEG	NEG	NEG	NEG	NEG	NEG	NEG	NEG	NEG	NEG	NEG	NEG	NEG	POS	NEG	NEG	NEG	NEG	POS	NEG	NEG	NEG	NEG	NEG	POS	NEG	NEG	NEG	NEG	NEG	NEG	NEG	NEG	NEG	NEG
NEG	NEG	NEG	NEG	NEG	NEG	NEG	NEG	NEG	NEG	NEG	NEG	NEG	NEG	NEG	NEG	NEG	NEG	NEG	NEG	NEG	NEG	NEG	NEG	NEG	NEG	NEG	NEG	POS	NEG	NEG	NEG	NEG	POS	NEG	NEG	NEG	NEG	NEG	POS	NEG	NEG	NEG	NEG	NEG	NEG	NEG	NEG	NEG	NEG
NEG	NEG	NEG	NEG	NEG	NEG	NEG	NEG	NEG	NEG	NEG	NEG	NEG	NEG	NEG	NEG	NEG	NEG	NEG	NEG	NEG	NEG	NEG	NEG	NEG	NEG	NEG	NEG	POS	NEG	NEG	NEG	NEG	POS	NEG	NEG	NEG	NEG	NEG	POS	NEG	NEG	NEG	NEG	NEG	NEG	NEG	NEG	NEG	NEG
NEG	NEG	NEG	NEG	NEG	NEG	NEG	NEG	NEG	NEG	NEG	NEG	NEG	NEG	NEG	NEG	NEG	NEG	NEG	NEG	NEG	NEG	NEG	NEG	NEG	NEG	NEG	NEG	POS	NEG	NEG	NEG	NEG	POS	NEG	NEG	NEG	NEG	NEG	POS	NEG	NEG	NEG	NEG	NEG	NEG	NEG	NEG	NEG	NEG
NEG	NEG	NEG	NEG	NEG	NEG	NEG	NEG	NEG	NEG	NEG	NEG	NEG	NEG	NEG	NEG	NEG	NEG	NEG	NEG	NEG	NEG	NEG	NEG	NEG	NEG	NEG	NEG	NEG	NEG	NEG	NEG	NEG	NEG	NEG	NEG	NEG	NEG	NEG	NEG	NEG	NEG	NEG	NEG	NEG	NEG	NEG	NEG	NEG	NEG
NEG	NEG	NEG	NEG	NEG	NEG	NEG	NEG	NEG	NEG	NEG	NEG	NEG	NEG	NEG	NEG	POS	NEG	NEG	NEG	NEG	NEG	NEG	NEG	NEG	NEG	NEG	NEG	NEG	NEG	NEG	NEG	NEG	POS	NEG	NEG	NEG	NEG	NEG	POS	NEG	NEG	NEG	NEG	NEG	NEG	NEG	NEG	NEG	NEG
NEG	NEG	NEG	NEG	NEG	NEG	NEG	NEG	NEG	NEG	NEG	NEG	NEG	NEG	NEG	NEG	POS	NEG	NEG	NEG	NEG	NEG	NEG	NEG	NEG	NEG	NEG	NEG	NEG	NEG	NEG	NEG	NEG	NEG	NEG	NEG	NEG	NEG	NEG	NEG	NEG	NEG	NEG	NEG	NEG	NEG	NEG	NEG	NEG	NEG
NEG	NEG	NEG	NEG	NEG	NEG	NEG	NEG	NEG	NEG	NEG	NEG	NEG	NEG	NEG	NEG	POS	NEG	NEG	NEG	NEG	NEG	NEG	NEG	NEG	NEG	NEG	NEG	NEG	NEG	NEG	NEG	POS	NEG	NEG	NEG	NEG	NEG	NEG	NEG	NEG	NEG	POS	POS	AMB	POS	POS	POS	POS	POS
NEG	NEG	NEG	NEG	NEG	NEG	NEG	NEG	NEG	NEG	NEG	NEG	NEG	NEG	NEG	NEG	NEG	NEG	NEG	NEG	NEG	NEG	NEG	NEG	NEG	NEG	NEG	NEG	NEG	NEG	NEG	NEG	NEG	NEG	NEG	NEG	NEG	NEG	NEG	NEG	NEG	NEG	NEG	NEG	NEG	NEG	NEG	NEG	NEG	NEG
NEG	NEG	NEG	NEG	NEG	NEG	NEG	NEG	NEG	NEG	NEG	NEG	NEG	NEG	NEG	NEG	NEG	NEG	NEG	NEG	NEG	NEG	NEG	NEG	NEG	NEG	NEG	NEG	NEG	NEG	NEG	NEG	NEG	NEG	NEG	NEG	NEG	NEG	NEG	NEG	NEG	NEG	NEG	NEG	NEG	NEG	NEG	NEG	NEG	NEG
NEG	NEG	NEG	NEG	NEG	NEG	NEG	NEG	NEG	NEG	NEG	NEG	NEG	NEG	NEG	NEG	NEG	NEG	NEG	NEG	NEG	NEG	NEG	NEG	NEG	NEG	NEG	NEG	NEG	NEG	NEG	NEG	NEG	NEG	NEG	NEG	NEG	NEG	NEG	NEG	NEG	NEG	NEG	NEG	NEG	NEG	NEG	NEG	NEG	NEG
NEG	POS	NEG	NEG	NEG	NEG	NEG	POS	NEG	NEG	NEG	NEG	NEG	NEG	NEG	NEG	NEG	NEG	NEG	NEG	NEG	NEG	NEG	NEG	NEG	NEG	NEG	NEG	NEG	NEG	NEG	NEG	NEG	NEG	NEG	NEG	NEG	NEG	NEG	NEG	NEG	NEG	NEG	NEG	NEG	NEG	NEG	NEG	NEG	NEG
NEG	NEG	NEG	NEG	NEG	NEG	NEG	NEG	NEG	NEG	NEG	NEG	NEG	NEG	NEG	NEG	NEG	NEG	NEG	NEG	NEG	NEG	NEG	NEG	NEG	NEG	NEG	NEG	NEG	NEG	NEG	NEG	NEG	NEG	NEG	NEG	NEG	NEG	NEG	NEG	NEG	NEG	NEG	NEG	NEG	NEG	NEG	NEG	NEG	NEG
NEG	NEG	NEG	NEG	NEG	NEG	NEG	NEG	NEG	NEG	NEG	NEG	NEG	NEG	NEG	NEG	NEG	NEG	NEG	NEG	NEG	NEG	NEG	NEG	NEG	NEG	NEG	NEG	NEG	NEG	NEG	NEG	NEG	NEG	NEG	NEG	NEG	NEG	NEG	NEG	NEG	NEG	NEG	NEG	NEG	NEG	NEG	NEG	NEG	NEG
NEG	NEG	NEG	NEG	NEG	NEG	NEG	NEG	NEG	NEG	NEG	NEG	NEG	NEG	NEG	NEG	NEG	NEG	NEG	NEG	NEG	NEG	NEG	NEG	NEG	NEG	NEG	NEG	NEG	NEG	NEG	NEG	NEG	NEG	NEG	NEG	NEG	NEG	NEG	NEG	NEG	NEG	NEG	NEG	NEG	NEG	NEG	NEG	NEG	NEG
NEG	NEG	NEG	NEG	NEG	NEG	NEG	NEG	NEG	NEG	NEG	NEG	NEG	NEG	NEG	NEG	NEG	NEG	NEG	NEG	NEG	NEG	NEG	NEG	NEG	NEG	NEG	NEG	NEG	NEG	NEG	NEG	NEG	NEG	NEG	NEG	NEG	NEG	NEG	NEG	NEG	NEG	NEG	NEG	NEG	NEG	NEG	NEG	NEG	NEG
NEG	NEG	NEG	NEG	NEG	NEG	NEG	NEG	NEG	NEG	NEG	NEG	NEG	NEG	NEG	NEG	NEG	NEG	NEG	NEG	NEG	NEG	NEG	NEG	NEG	NEG	NEG	NEG	NEG	NEG	NEG	NEG	NEG	NEG	NEG	NEG	NEG	NEG	NEG	NEG	NEG	NEG	NEG	NEG	NEG	NEG	NEG	NEG	NEG	NEG
NEG	NEG	NEG	NEG	NEG	NEG	NEG	NEG	NEG	NEG	NEG	NEG	NEG	NEG	NEG	NEG	NEG	NEG	NEG	NEG	NEG	NEG	NEG	NEG	NEG	NEG	NEG	NEG	NEG	NEG	NEG	NEG	NEG	NEG	NEG	NEG	NEG	NEG	NEG	NEG	NEG	NEG	NEG	NEG	NEG	NEG	NEG	NEG	NEG	NEG
NEG	NEG	NEG	NEG	NEG	NEG	NEG	NEG	NEG	NEG	NEG	NEG	NEG	NEG	NEG	NEG	NEG	NEG	NEG	NEG	NEG	NEG	NEG	NEG	NEG	NEG	NEG	NEG	NEG	POS	NEG	NEG	NEG	NEG	NEG	NEG	NEG	NEG	NEG	NEG	NEG	NEG	NEG	NEG	NEG	NEG	NEG	NEG	NEG	NEG
NEG	NEG	NEG	NEG	NEG	NEG	NEG	NEG	NEG	NEG	NEG	NEG	NEG	NEG	NEG	NEG	NEG	NEG	NEG	NEG	NEG	NEG	NEG	NEG	NEG	NEG	NEG	NEG	NEG	NEG	NEG	NEG	NEG	NEG	NEG	NEG	NEG	NEG	NEG	NEG	NEG	NEG	NEG	NEG	NEG	NEG	NEG	NEG	NEG	NEG
NEG	NEG	NEG	NEG	NEG	NEG	NEG	NEG	NEG	NEG	NEG	NEG	NEG	NEG	NEG	NEG	NEG	NEG	NEG	NEG	NEG	NEG	NEG	NEG	NEG	NEG	NEG	NEG	NEG	NEG	NEG	NEG	NEG	NEG	NEG	NEG	NEG	NEG	NEG	NEG	NEG	NEG	NEG	NEG	NEG	NEG	NEG	NEG	NEG	NEG
NEG	NEG	NEG	NEG	NEG	NEG	NEG	NEG	NEG	NEG	NEG	NEG	NEG	NEG	NEG	NEG	NEG	NEG	NEG	NEG	NEG	NEG	NEG	NEG	NEG	NEG	NEG	NEG	NEG	NEG	NEG	NEG	NEG	NEG	NEG	NEG	NEG	NEG	NEG	NEG	NEG	NEG	NEG	NEG	NEG	NEG	NEG	NEG	NEG	NEG
NEG	NEG	NEG	NEG	NEG	NEG	NEG	NEG	NEG	NEG	NEG	NEG	NEG	NEG	NEG	NEG	NEG	NEG	NEG	NEG	NEG	NEG	NEG	NEG	NEG	NEG	NEG	NEG	NEG	NEG	NEG	NEG	NEG	NEG	NEG	NEG	NEG	NEG	NEG	NEG	NEG	NEG	NEG	NEG	NEG	NEG	NEG	NEG	NEG	NEG
NEG	NEG	NEG	NEG	NEG	NEG	NEG	NEG	NEG	NEG	NEG	NEG	NEG	NEG	NEG	NEG	NEG	NEG	NEG	NEG	NEG	NEG	NEG	NEG	NEG	NEG	NEG	NEG	NEG	NEG	NEG	NEG	NEG	NEG	NEG	NEG	NEG	NEG	NEG	NEG	NEG	NEG	NEG	NEG	NEG	NEG	NEG	NEG	NEG	NEG
NEG	NEG	NEG	NEG	NEG	NEG	NEG	NEG	NEG	NEG	NEG	NEG	NEG	NEG	NEG	NEG	NEG	NEG	NEG	NEG	NEG	NEG	NEG	NEG	NEG	NEG	NEG	NEG	NEG	NEG	NEG	NEG	NEG	NEG	NEG	NEG	NEG	NEG	NEG	NEG	NEG	NEG	NEG	NEG	NEG	NEG	NEG	NEG	NEG	NEG
NEG	NEG	NEG	NEG	NEG	NEG	NEG	NEG	NEG	NEG	NEG	NEG	NEG	NEG	NEG	NEG	NEG	NEG	NEG	NEG	NEG	NEG	NEG	NEG	NEG	NEG	NEG	NEG	NEG	NEG	NEG	NEG	NEG	NEG	NEG	NEG	NEG	NEG	NEG	NEG	NEG	NEG	NEG	NEG	NEG	NEG	NEG	NEG	NEG	NEG
POS	NEG	POS	POS	NEG	NEG	NEG	NEG	POS	NEG	NEG	NEG	NEG	NEG	NEG	NEG	POS	NEG	POS	NEG	NEG	NEG	NEG	NEG	NEG	NEG	NEG	NEG	NEG	NEG	NEG	NEG	NEG	NEG	NEG	NEG	NEG	NEG	POS	NEG	POS	NEG	NEG	NEG	NEG	NEG	NEG	NEG	NEG	NEG
POS	NEG	POS	POS	NEG	NEG	NEG	NEG	POS	NEG	NEG	NEG	NEG	NEG	NEG	NEG	POS	NEG	POS	NEG	NEG	NEG	NEG	NEG	NEG	NEG	NEG	NEG	NEG	NEG	NEG	NEG	NEG	NEG	NEG	NEG	NEG	NEG	POS	NEG	POS	NEG	NEG	NEG	NEG	NEG	NEG	NEG	NEG	NEG
POS	NEG	POS	POS	NEG	NEG	NEG	NEG	POS	NEG	NEG	NEG	NEG	NEG	NEG	NEG	POS	NEG	POS	NEG	NEG	NEG	NEG	NEG	NEG	NEG	NEG	NEG	NEG	POS	NEG	NEG	NEG	NEG	NEG	NEG	NEG	NEG	POS	NEG	POS	NEG	NEG	NEG	NEG	NEG	NEG	NEG	NEG	NEG
POS	NEG	POS	POS	NEG	NEG	NEG	NEG	POS	NEG	NEG	NEG	NEG	NEG	NEG	NEG	POS	NEG	POS	NEG	NEG	NEG	NEG	NEG	NEG	NEG	NEG	NEG	NEG	POS	NEG	NEG	NEG	NEG	NEG	NEG	NEG	NEG	POS	NEG	POS	NEG	NEG	NEG	NEG	NEG	NEG	NEG	NEG	NEG
POS	NEG	POS	POS	NEG	NEG	NEG	NEG	POS	NEG	NEG	NEG	NEG	NEG	NEG	NEG	POS	NEG	POS	NEG	NEG	NEG	NEG	NEG	NEG	NEG	NEG	NEG	NEG	NEG	NEG	NEG	NEG	NEG	NEG	NEG	NEG	NEG	POS	NEG	POS	NEG	NEG	NEG	NEG	NEG	NEG	NEG	NEG	NEG
POS	NEG	POS	POS	NEG	NEG	NEG	NEG	POS	NEG	NEG	NEG	NEG	NEG	NEG	NEG	POS	NEG	NEG	NEG	NEG	NEG	NEG	NEG	NEG	NEG	NEG	NEG	NEG	NEG	NEG	NEG	NEG	NEG	NEG	NEG	NEG	NEG	POS	NEG	POS	NEG	NEG	NEG	NEG	NEG	NEG	NEG	NEG	NEG
POS	NEG	POS	POS	NEG	NEG	NEG	NEG	POS	NEG	NEG	NEG	NEG	NEG	NEG	NEG	POS	NEG	POS	NEG	NEG	NEG	NEG	NEG	NEG	NEG	NEG	NEG	NEG	NEG	NEG	NEG	NEG	NEG	NEG	NEG	NEG	NEG	POS	NEG	POS	NEG	NEG	NEG	NEG	NEG	NEG	NEG	NEG	NEG
NEG	NEG	NEG	NEG	NEG	NEG	NEG	NEG	NEG	NEG	NEG	NEG	NEG	NEG	NEG	NEG	NEG	NEG	NEG	NEG	NEG	NEG	NEG	NEG	NEG	NEG	NEG	NEG	NEG	NEG	NEG	NEG	NEG	NEG	NEG	NEG	NEG	NEG	NEG	NEG	NEG	NEG	NEG	NEG	NEG	NEG	NEG	NEG	NEG	NEG
NEG	NEG	NEG	NEG	NEG	NEG	NEG	NEG	NEG	NEG	NEG	NEG	NEG	NEG	NEG	NEG	NEG	NEG	NEG	NEG	NEG	NEG	NEG	NEG	NEG	NEG	NEG	NEG	NEG	NEG	NEG	NEG	NEG	NEG	NEG	NEG	NEG	NEG	NEG	NEG	NEG	NEG	NEG	NEG	NEG	NEG	NEG	NEG	NEG	NEG
NEG	NEG	NEG	NEG	NEG	NEG	NEG	NEG	NEG	NEG	NEG	NEG	NEG	NEG	NEG	NEG	NEG	NEG	NEG	NEG	NEG	NEG	NEG	NEG	NEG	NEG	NEG	NEG	NEG	NEG	NEG	NEG	NEG	NEG	NEG	NEG	NEG	NEG	NEG	NEG	NEG	NEG	NEG	NEG	NEG	NEG	NEG	NEG	NEG	NEG
NEG	NEG	NEG	NEG	NEG	NEG	NEG	NEG	NEG	NEG	NEG	NEG	NEG	NEG	NEG	NEG	NEG	NEG	NEG	NEG	NEG	NEG	NEG	NEG	NEG	NEG	NEG	NEG	NEG	NEG	NEG	NEG	NEG	NEG	NEG	NEG	NEG	NEG	NEG	NEG	NEG	NEG	NEG	NEG	NEG	NEG	NEG	NEG	NEG	NEG
NEG	NEG	NEG	NEG	NEG	NEG	NEG	NEG	NEG	NEG	NEG	NEG	NEG	NEG	NEG	NEG	NEG	NEG	NEG	NEG	NEG	NEG	NEG	NEG	NEG	NEG	NEG	NEG	NEG	NEG	NEG	NEG	NEG	NEG	NEG	NEG	NEG	NEG	NEG	NEG	NEG	NEG	NEG	NEG	NEG	NEG	NEG	NEG	NEG	NEG
NEG	NEG	NEG	NEG	NEG	NEG	NEG	NEG	NEG	NEG	NEG	NEG	NEG	NEG	NEG	NEG	NEG	NEG	NEG	NEG	NEG	NEG	NEG	NEG	NEG	NEG	NEG	NEG	NEG	NEG	NEG	NEG	NEG	NEG	NEG	NEG	NEG	NEG	NEG	NEG	NEG	NEG	NEG	NEG	NEG	NEG	NEG	NEG	NEG	NEG
NEG	NEG	NEG	NEG	NEG	NEG	NEG	NEG	NEG	NEG	NEG	NEG	NEG	NEG	NEG	NEG	NEG	NEG	NEG	NEG	NEG	NEG	NEG	NEG	NEG	NEG	NEG	NEG	NEG	NEG	NEG	NEG	NEG	NEG	NEG	NEG	NEG	NEG	NEG	NEG	NEG	NEG	NEG	NEG	NEG	NEG	NEG	NEG	NEG	NEG

VIRULENCE : HLG AND LEUKOCIDINS											VIRULENCE : HAEMOLYSINS											VIRULENCE : HLB-CONV PHAGES			VIRULENCE : OTHER FACTORS						VIRULENCE : PROTEASES									
lukF	lukS		hlgA	PVL LATERAL FLOW	lukF-PV	lukS-PV	lukF-PV (P83)	lukM	lukD	lukE	lukX	lukY		corB	hla	hlIII		hlb				sak	chp	scn	etA	etB	etD	edinA	edinB	edinC	aur			splA	splB	splE	sspA	sspB	sspP	
lukF	lukS	lukS (ST22+ST45)										lukY	lukY (ST30+ST45)			hlIII (cons)	hlIII (other than RF122)	hlb-probe 1	hlb-probe 2	hlb-probe 3	un-truncated hlb										aur (cons)	aur (Other than MRSA252)	aur (MRSA252)						sspP (cons)	sspP (other than ST93)
Haemolysin gamma / leukocidin, component B	Haemolysin gamma / leukocidin, component C		Haemolysin gamma, component A		Panton Valentine leukocidin F component	Panton Valentine leukocidin S component	F component from ruminant leukocidin	S component from ruminant leukocidin	leukocidin D component	leukocidin E component	leukocidin/ haemolysin toxin family protein	leukocidin/haemolysin toxin family protein		Putative membrane protein	Haemolysin alpha	Putative membrane protein		haemolysin beta				staphylo-kinase	chemotaxis-inhibiting protein (CHIPS)	Staphyl. Comple-ment inhibitor	exfoliative toxin serotype A	exfoliative toxin serotype B	exfoliative toxin D	epidermal cell differen-tiation inhibitor	epidermal cell differen-tiation inhibitor B	epidermal cell differen-tiation inhibitor C	aureolysin			serin- protease A	serin- protease B	serin- protease E	glutamylendop eptidase	Staphopain B, protease	Staphopain A (Staphylopain A), protease	
POS	POS	POS	POS	negative	NEG	NEG	NEG	NEG	POS	POS	POS	POS	NEG	POS	POS	POS	POS	POS	POS	POS	NEG	POS	NEG	POS	NEG	NEG	NEG	NEG	NEG	NEG	POS	POS	NEG	POS	POS	POS	POS	POS	POS	POS
POS	POS	POS	POS	negative	NEG	NEG	NEG	NEG	POS	POS	POS	POS	NEG	POS	POS	POS	POS	POS	POS	POS	NEG	POS	NEG	POS	NEG	NEG	NEG	NEG	NEG	NEG	POS	POS	NEG	POS	POS	POS	POS	POS	POS	POS
POS	POS	NEG	POS	negative	NEG	NEG	NEG	NEG	POS	POS	POS	POS	NEG	POS	NEG	POS	POS	NEG	AMB	NEG	NEG	POS	NEG	POS	NEG	NEG	NEG	NEG	NEG	NEG	POS	POS	NEG	POS	POS	POS	POS	POS	POS	POS
POS	POS	NEG	POS	negative	NEG	NEG	NEG	NEG	POS	POS	POS	POS	NEG	POS	NEG	POS	POS	NEG	AMB	NEG	NEG	POS	NEG	POS	NEG	NEG	NEG	NEG	NEG	NEG	POS	POS	NEG	POS	POS	POS	POS	POS	POS	POS
POS	POS	NEG	POS	negative	NEG	NEG	NEG	NEG	POS	POS	NEG	NEG	NEG	POS	NEG	POS	NEG	POS	NEG	NEG	NEG	NEG	NEG	NEG	NEG	NEG	NEG	NEG	NEG	NEG	NEG	POS	NEG	POS	POS	POS	POS	POS	POS	POS
POS	POS	NEG	POS	negative	NEG	NEG	NEG	NEG	POS	POS	POS	NEG	NEG	POS	NEG	POS	AMB	NEG	POS	NEG	NEG	POS	NEG	AMB	NEG	NEG	NEG	NEG	NEG	NEG	NEG	POS	NEG	POS	POS	NEG	POS	POS	POS	POS
POS	POS	POS	POS	POSITIVE	POS	POS	NEG	NEG	POS	POS	POS	POS	NEG	POS	POS	POS	POS	NEG	POS	NEG	NEG	POS	POS	POS	NEG	NEG	NEG	POS	NEG	NEG	POS	POS	NEG	POS	POS	NEG	POS	POS	POS	POS
POS	POS	NEG	POS	POSITIVE	POS	POS	NEG	NEG	POS	POS	POS	NEG	NEG	POS	NEG	POS	POS	NEG	POS	NEG	NEG	POS	POS	POS	NEG	NEG	NEG	POS	NEG	NEG	AMB	POS	NEG	POS	POS	NEG	POS	POS	POS	POS
POS	POS	POS	POS	negative	NEG	NEG	NEG	NEG	POS	POS	POS	POS	NEG	POS	POS	POS	POS	AMB	POS	NEG	POS	NEG	NEG	NEG	POS	NEG	NEG	NEG	NEG	NEG	POS	POS	NEG	POS	POS	POS	POS	POS	POS	POS
POS	POS	POS	POS	negative	NEG	NEG	NEG	NEG	POS	POS	POS	POS	NEG	POS	POS	POS	POS	POS	POS	POS	NEG	POS	NEG	POS	NEG	NEG	NEG	NEG	NEG	NEG	POS	POS	NEG	POS	POS	POS	POS	POS	POS	POS
POS	POS	POS	POS	negative	NEG	NEG	NEG	NEG	POS	POS	POS	POS	NEG	POS	POS	POS	POS	POS	POS	POS	NEG	POS	NEG	POS	POS	NEG	NEG	NEG	NEG	NEG	POS	POS	NEG	POS	POS	POS	POS	POS	POS	POS
POS	POS	POS	POS	negative	NEG	NEG	NEG	NEG	POS	POS	POS	POS	NEG	POS	POS	POS	POS	POS	POS	POS	NEG	POS	NEG	POS	NEG	NEG	NEG	NEG	NEG	NEG	POS	POS	NEG	POS	POS	POS	POS	POS	POS	POS
POS	POS	NEG	POS	negative	NEG	NEG	NEG	NEG	POS	POS	POS	NEG	NEG	POS	NEG	POS	POS	NEG	POS	NEG	NEG	POS	NEG	POS	NEG	NEG	NEG	NEG	NEG	NEG	POS	POS	NEG	POS	POS	POS	POS	POS	POS	POS
POS	POS	POS	POS	POSITIVE	POS	POS	NEG	NEG	POS	POS	POS	POS	NEG	POS	POS	POS	POS	POS	POS	POS	NEG	POS	NEG	POS	NEG	NEG	NEG	NEG	NEG	NEG	POS	POS	NEG	POS	POS	POS	POS	POS	POS	POS
POS	POS	POS	POS	POSITIVE	POS	POS	NEG	NEG	POS	POS	POS	POS	NEG	POS	POS	POS	POS	POS	POS	POS	NEG	POS	NEG	POS	NEG	NEG	NEG	NEG	NEG	NEG	POS	POS	NEG	POS	POS	POS	POS	POS	POS	POS
POS	POS	AMB	POS	POSITIVE	POS	POS	NEG	NEG	POS	POS	POS	POS	NEG	POS	POS	POS	POS	POS	POS	AMB	NEG	POS	NEG	POS	NEG	NEG	NEG	NEG	NEG	NEG	POS	POS	NEG	POS	POS	POS	POS	POS	POS	POS
POS	POS	POS	POS	POSITIVE	POS	POS	NEG	NEG	POS	POS	POS	POS	NEG	POS	POS	POS	POS	POS	POS	POS	NEG	POS	NEG	POS	NEG	NEG	NEG	NEG	NEG	NEG	POS	POS	NEG	POS	POS	POS	POS	POS	POS	POS
POS	POS	POS	POS	POSITIVE	POS	POS	NEG	NEG	POS	POS	POS	POS	NEG	POS	POS	POS	POS	POS	POS	POS	NEG	POS	NEG	POS	NEG	NEG	NEG	NEG	NEG	NEG	POS	POS	NEG	POS	POS	POS	POS	POS	POS	POS
POS	POS	NEG	POS	POSITIVE	POS	POS	NEG	NEG	POS	POS	POS	NEG	NEG	POS	NEG	POS	AMB	NEG	POS	NEG	NEG	POS	NEG	POS	NEG	NEG	NEG	NEG	NEG	NEG	NEG	POS	NEG	POS	POS	POS	POS	POS	POS	POS
POS	POS	POS	POS	negative	NEG	NEG	NEG	NEG	POS	POS	POS	POS	NEG	POS	POS	POS	POS	POS	POS	POS	NEG	POS	NEG	POS	NEG	NEG	NEG	NEG	NEG	NEG	POS	POS	NEG	POS	POS	POS	POS	POS	POS	POS
POS	POS	POS	POS	negative	NEG	NEG	NEG	NEG	POS	POS	POS	POS	NEG	POS	POS	POS	POS	POS	POS	POS	NEG	POS	NEG	POS	NEG	NEG	NEG	NEG	NEG	NEG	POS	POS	NEG	POS	POS	POS	POS	POS	POS	POS
POS	AMB	POS	POS	negative	NEG	NEG	NEG	NEG	POS	POS	POS	POS	NEG	POS	POS	POS	POS	POS	POS	POS	NEG	POS	NEG	POS	NEG	NEG	NEG	NEG	NEG	NEG	POS	POS	NEG	POS	POS	POS	POS	POS	POS	POS
POS	AMB	POS	POS	negative	NEG	NEG	NEG	NEG	POS	POS	POS	POS	NEG	POS	POS	POS	POS	POS	POS	POS	NEG	POS	NEG	POS	NEG	NEG	NEG	NEG	NEG	NEG	POS	POS	NEG	POS	POS	POS	POS	POS	POS	POS
POS	AMB	POS	POS	negative	NEG	NEG	NEG	NEG	POS	POS	POS	POS	NEG	POS	POS	POS	POS	POS	POS	POS	NEG	POS	NEG	POS	NEG	NEG	NEG	NEG	NEG	NEG	POS	POS	NEG	POS	POS	POS	POS	POS	POS	POS
POS	POS	POS	POS	negative	NEG	NEG	NEG	NEG	POS	POS	POS	POS	NEG	POS	POS	POS	POS	POS	POS	POS	NEG	POS	POS	POS	NEG	NEG	NEG	NEG	NEG	NEG	POS	POS	NEG	POS	POS	POS	POS	POS	POS	POS
POS	POS	NEG	POS	negative	NEG	NEG	NEG	NEG	POS	POS	NEG	NEG	NEG	POS	NEG	POS	NEG	POS	POS	NEG	NEG	POS	POS	NEG	NEG	NEG	NEG	NEG	NEG	NEG	NEG	POS	NEG	POS	POS	POS	POS	POS	POS	POS
POS	POS	NEG	POS	POSITIVE	POS	POS	NEG	NEG	POS	POS	POS	POS	NEG	POS	POS	POS	POS	POS	POS	POS	NEG	POS	POS	POS	NEG	NEG	NEG	NEG	NEG	NEG	POS	POS	NEG	NEG	NEG	NEG	POS	POS	POS	POS
POS	POS	NEG	POS	POSITIVE	POS	POS	NEG	NEG	POS	POS	POS	POS	NEG	POS	POS	POS	POS	POS	POS	POS	NEG	POS	POS	POS	NEG	NEG	NEG	NEG	NEG	NEG	POS	POS	NEG	NEG	NEG	NEG	POS	POS	POS	POS
POS	POS	NEG	POS	POSITIVE	POS	POS	NEG	NEG	POS	POS	POS	POS	NEG	POS	POS	POS	POS	AMB	POS	POS	NEG	POS	POS	POS	NEG	NEG	NEG	NEG	NEG	NEG	POS	POS	NEG	NEG	NEG	NEG	POS	POS	POS	POS
POS	POS	AMB	POS	POSITIVE	POS	POS	NEG	NEG	POS	POS	POS	POS	NEG	POS	POS	POS	POS	POS	POS	POS	NEG	POS	POS	POS	NEG	NEG	NEG	NEG	NEG	NEG	POS	POS	NEG	POS	POS	POS	POS	POS	POS	POS
POS	POS	POS	POS	POSITIVE	POS	POS	NEG	NEG	POS	POS	POS	POS	NEG	POS	POS	POS	POS	POS	POS	POS	NEG	POS	POS	POS	NEG	NEG	NEG	NEG	NEG	NEG	POS	POS	NEG	NEG	NEG	NEG	POS	POS	POS	POS
POS	POS	POS	POS	POSITIVE	POS	POS	NEG	NEG	POS	POS	POS	POS	NEG	POS	POS	POS	POS	POS	POS	POS	NEG	POS	POS	POS	NEG	NEG	NEG	NEG	NEG	NEG	POS	POS	NEG	NEG	NEG	NEG	POS	POS	POS	POS
POS	POS	POS	POS	POSITIVE	POS	POS	NEG	NEG	POS	POS	POS	POS	NEG	POS	POS	POS	POS	POS	POS	POS	NEG	POS	POS	POS	NEG	NEG	NEG	NEG	NEG	NEG	POS	POS	NEG	NEG	NEG	NEG	POS	POS	POS	POS
POS	POS	POS	POS	negative !	POS	POS	NEG	NEG	POS	POS	POS	POS	NEG	POS	POS	POS	POS	POS	POS	POS	NEG	POS	POS	POS	NEG	NEG	NEG	NEG	NEG	NEG	POS	POS	NEG	NEG	NEG	NEG	POS	POS	POS	POS
POS	POS	POS	POS	POSITIVE	POS	POS	NEG	NEG	POS	POS	POS	POS	NEG	POS	POS	POS	POS	POS	POS	POS	NEG	POS	POS	POS	NEG	NEG	NEG	NEG	NEG	NEG	POS	POS	NEG	NEG	NEG	NEG	POS	POS	POS	POS
POS	POS	POS	POS	POSITIVE	POS	POS	NEG	NEG	POS	POS	POS	POS	NEG	POS	POS	POS	POS	POS	POS	POS	NEG	POS	POS	POS	NEG	NEG	NEG	NEG	NEG	NEG	POS	POS	NEG	NEG	NEG	NEG	POS	POS	POS	POS
POS	POS	POS	POS	POSITIVE	POS	POS	NEG	NEG	POS	POS	POS	POS	NEG	POS	POS	POS	POS	POS	POS	POS	NEG	POS	POS	POS	NEG	NEG	NEG	NEG	NEG	NEG	POS	POS	NEG	NEG	NEG	NEG	POS	POS	POS	POS
POS	POS	NEG	POS	POSITIVE	AMB	POS	NEG	NEG	POS	POS	NEG	NEG	NEG	POS	NEG	POS	AMB	POS	POS	NEG	NEG	POS	POS	POS	NEG	NEG	NEG	NEG	NEG	NEG	POS	POS	NEG	NEG	NEG	NEG	POS	POS	POS	POS
POS	POS	POS	POS	POSITIVE	POS	POS	NEG	NEG	POS	POS	POS	POS	NEG	POS	POS	POS	POS	POS	POS	POS	NEG	POS	POS	POS	NEG	NEG	NEG	NEG	NEG	NEG	POS	POS	NEG	POS	POS	POS	POS	POS	POS	POS
POS	POS	POS	POS	negative	NEG	NEG	NEG	NEG	NEG	NEG	POS	POS	NEG	POS	POS	POS	POS	POS	POS	POS	NEG	POS	POS	POS	NEG	NEG	NEG	NEG	NEG	NEG	POS	POS	NEG	NEG	NEG	NEG	POS	POS	POS	POS
POS	POS	POS	POS	negative	NEG	NEG	NEG	NEG	POS	POS	POS	POS	NEG	POS	POS	POS	POS	POS	POS	POS	NEG	POS	POS	POS	NEG	NEG	NEG	NEG	NEG	NEG	POS	POS	NEG	POS	POS	POS	POS	POS	POS	POS
POS	POS	POS	POS	negative	NEG	NEG	NEG	NEG	POS	POS	POS	POS	NEG	POS	POS	POS	POS	NEG	NEG	NEG	NEG	NEG	POS	POS	NEG	NEG	NEG	NEG	NEG	NEG	POS	POS	NEG	POS	POS	POS	POS	POS	POS	POS
POS	POS	POS	POS	negative	NEG	NEG	NEG	NEG	POS	POS	POS	POS	NEG	POS	POS	POS	POS	NEG	NEG	NEG	NEG	NEG	POS	POS	NEG	NEG	NEG	NEG	NEG	NEG	POS	POS	NEG	POS	POS	POS	POS	POS	POS	POS
POS	POS	POS	POS	negative	NEG	NEG	NEG	NEG	POS	POS	POS	POS	NEG	POS	POS	POS	POS	NEG	NEG	NEG	NEG	NEG	POS	POS	NEG	NEG	NEG	NEG	NEG	NEG	POS	POS	NEG	POS	POS	POS	POS	POS	POS	POS
POS	POS	POS	POS	POSITIVE	POS	POS	NEG	NEG	NEG	NEG	POS	POS	NEG	POS	POS	NEG	NEG	POS	POS	POS	NEG	POS	POS	POS	NEG	NEG	NEG	NEG	NEG	NEG	POS	NEG	POS	NEG	NEG	NEG	POS	POS	POS	POS
POS	POS	NEG	POS	negative	NEG	NEG	NEG	NEG	NEG	NEG	POS	NEG	POS	POS	NEG	POS	POS	POS	POS	AMB	NEG	POS	POS	POS	NEG	NEG	NEG	NEG	NEG	NEG	POS	NEG	POS	NEG	NEG	POS	POS	POS	POS	POS
POS	POS	POS	POS	negative	NEG	NEG	NEG	NEG	NEG	NEG	POS	NEG	POS	POS	POS	POS	NEG	NEG	NEG	NEG	NEG	POS	POS	POS	NEG	NEG	NEG	NEG	NEG	NEG	NEG	NEG	POS	NEG	NEG	NEG	POS	POS	POS	POS
POS	POS	POS	POS	negative	NEG	NEG	NEG	NEG	NEG	NEG	POS	NEG	POS	POS	POS	POS	NEG	NEG	NEG	NEG	NEG	POS	POS	POS	NEG	NEG	NEG	NEG	NEG	NEG	NEG	NEG	POS	NEG	NEG	NEG	POS	POS	POS	POS
POS	NEG	POS	POS	negative	NEG	NEG	NEG	NEG	NEG	NEG	POS	NEG	POS	POS	POS	POS	NEG	NEG	NEG	NEG	NEG	POS	POS	POS	NEG	NEG	NEG	NEG	NEG	NEG	NEG	NEG	POS	NEG	NEG	NEG	POS	POS	POS	POS
POS	POS	POS	POS	negative	NEG	NEG	NEG	NEG	NEG	NEG	POS	NEG	POS	POS	POS	POS	NEG	NEG	NEG	NEG	NEG	POS	POS	POS	NEG	NEG	NEG	NEG	NEG	NEG	POS	NEG	POS	NEG	NEG	NEG	POS	POS	POS	POS
AMB	POS	NEG	POS	negative	NEG	NEG	NEG	NEG	NEG	NEG	AMB	NEG	NEG	POS	NEG	POS	NEG	NEG	NEG	NEG	POS	POS	POS	POS	NEG	NEG	NEG	NEG	NEG	NEG	AMB	NEG	NEG	NEG	NEG	NEG	POS	POS	POS	POS
POS	POS	NEG	POS	negative	NEG	NEG	NEG	NEG	POS	POS	AMB	POS	NEG	POS	POS	POS	POS	POS	POS	POS	NEG	POS	POS	POS	NEG	NEG	NEG	NEG	NEG	NEG	POS	POS	NEG	POS	POS	POS	POS	POS	POS	POS
POS	POS	POS	POS	negative	NEG	NEG	NEG	NEG	POS	POS	POS	POS	NEG	POS	POS	POS	POS	POS	POS	POS	NEG	POS	POS	POS	NEG	NEG	NEG	NEG	NEG	NEG	POS	POS	NEG	POS	POS	POS	POS	POS	POS	POS
POS	POS	NEG	POS	negative	NEG	NEG	NEG	NEG	POS	POS	POS	POS	NEG	POS	NEG	POS	POS	POS	POS	NEG	NEG	POS	POS	POS	NEG	NEG	NEG	NEG	NEG	NEG	POS	POS	NEG	POS	POS	POS	POS	POS	POS	POS
POS	POS	POS	POS	negative	NEG	NEG	NEG	NEG	POS	POS	POS	POS	NEG	POS	POS	POS	POS	POS	POS	POS	NEG	POS	POS	POS	NEG	NEG	NEG	NEG	NEG	NEG	POS	POS	NEG	POS	POS	POS	POS	POS	POS	POS
POS	POS	POS	POS	negative	NEG	NEG	NEG	NEG	POS	POS	POS	POS	NEG	POS	POS	POS	POS	POS	POS	POS	NEG	POS	POS	POS	NEG	NEG	NEG	NEG	NEG	NEG	POS	POS	NEG	POS	POS	POS	POS	POS	POS	POS
POS	POS	POS	POS	negative	NEG	NEG	NEG	NEG	POS	POS	POS	POS	NEG	POS	POS	POS	POS	POS	POS	POS	NEG	POS	POS	POS	NEG	NEG	NEG	NEG	NEG	NEG	POS	POS	NEG	POS	POS	NEG	POS	POS	POS	POS
POS	POS	POS	POS	negative	NEG	NEG	NEG	NEG	POS	POS	POS	POS	NEG	POS	POS	POS	POS	POS	POS	POS	NEG	POS	NEG	POS	NEG	NEG	NEG	NEG	NEG	NEG	POS	POS	NEG	POS	POS	POS	POS	POS	POS	POS
POS	POS	POS	POS	negative	NEG	NEG	NEG	NEG	POS	POS	POS	POS	NEG	POS	POS	POS	POS	POS	POS	POS	NEG	POS	NEG	POS	NEG	NEG	NEG	NEG	NEG	NEG	POS	POS	NEG	POS	POS	POS	POS	POS	POS	POS
POS	POS	POS	POS	negative	NEG	NEG	NEG	NEG	POS	POS	POS	POS	NEG	POS	POS	POS	POS	POS	POS	POS	NEG	POS	NEG	POS	NEG	NEG	NEG	NEG	NEG	NEG	POS	POS	NEG	POS	POS	POS	POS	POS	POS	POS
POS	POS	POS	POS	negative	NEG	NEG	NEG	NEG	POS	POS	POS	POS	NEG	POS	POS	POS	POS	POS	POS	POS	NEG	POS	NEG	POS	NEG	NEG	NEG	NEG	NEG	NEG	POS	POS	NEG	POS	POS	POS	POS	POS	POS	POS
POS	POS	POS	POS	negative	NEG	NEG	NEG	NEG	POS	POS	POS	POS	NEG	POS	POS	POS	POS	POS	POS	POS	POS	NEG	NEG	NEG	NEG	NEG	NEG	NEG	NEG	NEG	POS	POS	NEG	POS	POS	POS	POS	POS	POS	POS
POS	POS	AMB	POS	negative	NEG	NEG	NEG	NEG	POS	POS	POS	POS	NEG	POS	POS	POS	POS	POS	POS	POS	NEG	POS	NEG	POS	NEG	NEG	NEG	NEG	NEG	NEG	POS	POS	NEG	POS	POS	POS	POS	POS	POS	POS
POS	POS	POS	POS	negative	NEG	NEG	NEG	NEG	POS	POS	POS	POS	NEG	POS	POS	POS	POS	POS	POS	POS	NEG	POS	NEG	POS	NEG	NEG	NEG	NEG	NEG	NEG	POS	POS	NEG	POS	POS	POS	POS	POS	POS	POS
POS	POS	NEG	POS	negative	NEG	NEG	NEG	NEG	NEG	POS	AMB	NEG	NEG	NEG	NEG	POS	NEG	POS	POS	AMB	NEG	POS	NEG	POS	NEG	NEG	NEG	NEG	NEG	NEG	POS	POS	NEG	POS	POS	POS	POS	POS	POS	POS
POS	POS	NEG	POS	negative	NEG	NEG	NEG	NEG	NEG	POS	POS	NEG	NEG	AMB	NEG	POS	AMB	POS	POS	AMB	NEG	POS	NEG	AMB	NEG	NEG	NEG	NEG	NEG	NEG	AMB	POS	NEG	POS	POS	POS	POS	POS	POS	POS
POS	POS	NEG	POS	negative	NEG	NEG	NEG	NEG	NEG	POS	POS	NEG	NEG	POS	NEG	POS	POS	POS	POS	NEG	POS	NEG	NEG	NEG	NEG	NEG	NEG	NEG	NEG	NEG	POS	POS	NEG	POS	POS	POS	POS	POS	POS	POS
POS	POS	POS	POS	negative	NEG	NEG	NEG	NEG	POS	POS	POS	POS	NEG	POS	POS	POS	POS	POS	POS	POS	NEG	POS	NEG	POS	NEG	NEG	NEG	NEG	NEG	NEG	POS	POS	NEG	POS	POS	POS	POS	POS	POS	POS
POS	POS	AMB	POS	negative	NEG	NEG	NEG	NEG	POS	POS	POS	POS	NEG	POS	POS	POS	POS	POS	POS	POS	NEG	POS	NEG	POS	NEG	NEG	NEG	NEG	NEG	NEG	POS	POS	NEG	POS	POS	POS	POS	POS	POS	POS
POS	POS	NEG	POS	negative	NEG	NEG	NEG	NEG	POS	POS	POS	POS	NEG	POS	POS	POS	NEG	POS	POS	POS	NEG	POS	NEG	POS	POS	NEG	NEG	NEG	NEG	NEG	POS	POS	NEG	POS	POS	NEG	POS	POS	POS	POS
POS	NEG	NEG	POS	negative	NEG	NEG	NEG	NEG	NEG	NEG	NEG	POS	NEG	NEG	POS	POS	NEG	NEG	NEG	POS	POS	POS	POS	POS	NEG	NEG	NEG	NEG	POS	NEG	POS	POS	NEG	NEG	NEG	NEG	POS	POS	POS	POS
POS	NEG	NEG	POS	POSITIVE	POS	POS	NEG	NEG	NEG	NEG	NEG	POS	NEG	NEG	POS	POS	NEG	NEG	NEG	POS	POS	POS	NEG	POS	NEG	NEG	NEG	NEG	POS	NEG	POS	POS	NEG	NEG	NEG	NEG	POS	POS	POS	POS
POS	NEG	NEG	POS	POSITIVE	POS	POS	NEG	NEG	NEG	NEG	NEG	POS	NEG	NEG	POS	POS	NEG	NEG	NEG	POS	POS	POS	NEG	POS	NEG	NEG	NEG	NEG	POS	NEG	POS	POS	NEG	NEG	NEG	NEG	POS	POS	POS	POS
POS	NEG	NEG	POS	POSITIVE	POS	POS	NEG	NEG	NEG	NEG	NEG	POS	NEG	NEG	POS	POS	NEG	NEG	NEG	POS	POS	POS	NEG	POS	NEG	NEG	NEG	NEG	POS	NEG	POS	POS	NEG	NEG	NEG	NEG	POS	POS	POS	POS
POS	NEG	NEG	POS	POSITIVE	POS	POS	NEG	NEG	NEG	NEG	NEG	POS	NEG	NEG	POS	POS	NEG	NEG	NEG	POS	POS	POS	NEG	POS	NEG	NEG	NEG	NEG	POS	NEG	POS	POS	NEG	NEG	NEG	NEG	POS	POS	POS	POS
POS	NEG	NEG	POS	POSITIVE	POS	POS	NEG	NEG	NEG	NEG	NEG	POS	NEG	NEG	POS	POS	NEG	NEG	NEG	POS	POS	POS	NEG	POS	NEG	NEG	NEG	NEG	POS	NEG	POS	POS	NEG	NEG	NEG	NEG	POS	POS	POS	POS
POS	NEG	NEG	POS	POSITIVE	POS	POS	NEG	NEG	NEG	NEG	NEG	POS	NEG	NEG	POS	POS	NEG	NEG	NEG	POS	POS	POS	NEG	POS	NEG	NEG	NEG	NEG	POS	NEG	POS	POS	NEG	NEG	NEG	NEG	POS	POS	POS	POS
POS	NEG	NEG	POS	POSITIVE	POS	POS	NEG	NEG	NEG	NEG	NEG	POS	NEG	NEG	POS	POS	NEG	NEG	NEG	POS	POS	POS	NEG	POS	NEG	NEG	NEG	NEG	POS	NEG	POS	POS	NEG	NEG	NEG	NEG	POS	POS	POS	POS
POS	NEG	NEG	POS	POSITIVE	POS	POS	NEG	NEG	NEG	NEG	NEG	POS	NEG	NEG	POS	POS	NEG	NEG	NEG	POS	POS	POS	NEG	POS	NEG	NEG	NEG	NEG	POS	NEG	POS	POS	NEG	NEG	NEG	NEG	POS	POS	POS	POS
POS	NEG	NEG	POS	POSITIVE	POS	POS	NEG	NEG	NEG	NEG	NEG	POS	NEG	NEG	POS	POS	NEG	NEG	NEG	POS	POS	POS	NEG	POS	NEG	NEG	NEG	NEG	POS	NEG	POS	POS	NEG	NEG	NEG	NEG	POS	POS	POS	POS
POS	NEG	NEG	POS	POSITIVE	POS	POS	NEG	NEG	NEG	NEG	NEG	POS	NEG	NEG	POS	POS	NEG	NEG	NEG	POS	POS	POS	NEG	POS	NEG	NEG	NEG	NEG	POS	NEG	POS	POS	NEG	NEG	NEG	NEG	POS	POS	POS	POS
NEG	NEG	NEG	NEG	POSITIVE	POS	POS	NEG	NEG	NEG	NEG	NEG	NEG	NEG	NEG	NEG	POS	NEG	NEG	NEG	NEG	POS	POS	NEG	POS	NEG	NEG	NEG	NEG	POS	NEG	NEG	POS	NEG	NEG	NEG	NEG	POS	POS	POS	POS
POS	NEG	NEG	POS	POSITIVE	POS	POS	NEG	NEG	NEG	NEG	NEG	POS	NEG	NEG	POS	POS	NEG	NEG	NEG	POS	POS	POS	NEG	POS	NEG	NEG	NEG	NEG	POS	NEG	POS	POS	NEG	NEG	NEG	NEG	POS	POS	POS	POS
POS	NEG	NEG	POS	POSITIVE	POS	POS	NEG	NEG	NEG	NEG	NEG	POS	NEG	NEG	POS	POS	NEG	NEG	NEG	POS	POS	POS	NEG	POS	NEG	NEG	NEG	NEG	POS	NEG	POS	POS	NEG	NEG	NEG	NEG	POS	POS	POS	POS
POS	NEG	NEG	POS	POSITIVE	POS	POS	NEG	NEG	NEG	NEG	NEG	POS	NEG	NEG	POS	POS	NEG	NEG	NEG	POS	POS	POS	NEG	POS	NEG	NEG	NEG	NEG	POS	NEG	POS	POS	NEG	NEG	NEG	NEG	POS	POS	POS	POS
POS	NEG	NEG	POS	POSITIVE	POS	POS	NEG	NEG	NEG	NEG	NEG	POS	NEG	NEG	POS	POS	NEG	NEG	NEG	POS	POS	POS	NEG	POS	NEG	NEG	NEG	NEG	POS	NEG	POS	POS	NEG	NEG	NEG	NEG	POS	POS	POS	POS
POS	POS	POS	POS	negative	NEG	NEG	NEG	NEG	POS	POS	POS	POS	NEG	POS	POS	POS	POS	POS	POS	POS	NEG	POS	NEG	POS	NEG	NEG	NEG	NEG	NEG	NEG	POS	POS	NEG	POS	POS	POS	POS	POS	POS	POS
POS	POS	NEG	POS	negative	NEG	NEG	NEG	NEG	POS	POS	POS	POS	NEG	POS	POS	POS	POS	POS	POS	POS	POS	NEG	NEG	NEG	NEG	NEG	NEG	NEG	NEG	NEG	POS	NEG	POS	POS	POS	POS	POS	POS	POS	POS
POS	POS	NEG	POS	negative	NEG	NEG	NEG	NEG	POS	POS	POS	POS	NEG	POS	POS	POS	POS	POS	POS	POS	POS	NEG	NEG	NEG	NEG	NEG	NEG	NEG	NEG	NEG	POS	NEG	POS	POS	POS	POS	POS	POS	POS	POS
POS	POS	NEG	POS	negative	NEG	NEG	NEG	NEG	POS	POS	POS	POS	NEG	POS	POS	POS	POS	POS	POS	POS	NEG	POS	NEG	POS	NEG	NEG	NEG	NEG	NEG	NEG	POS	NEG	POS	POS	POS	POS	POS	POS	POS	POS
POS	POS	NEG	POS	negative	NEG	NEG	NEG	NEG	POS	POS	POS	POS	NEG	POS	POS	POS	POS	POS	POS	POS	NEG	POS	NEG	POS	NEG	NEG	NEG	NEG	NEG	NEG	POS	NEG	POS	POS	POS	POS	POS	POS	POS	POS
POS	POS	NEG	POS	negative	NEG	NEG	NEG	NEG	POS	POS	POS	POS	NEG	POS	POS	POS	POS	POS	POS	POS	POS	NEG	NEG	NEG	NEG	NEG	NEG	NEG	NEG	NEG	POS	NEG	POS	POS	POS	POS	POS	POS	POS	POS
POS	POS	NEG	POS	negative	NEG	NEG	NEG	NEG	POS	POS	POS	POS	NEG	POS	POS	POS	POS	POS	POS	POS	POS	NEG	NEG	NEG	NEG	NEG	NEG	NEG	NEG	NEG	POS	NEG	POS	POS	POS	POS	POS	POS	POS	POS
POS	POS	NEG	POS	negative	NEG	NEG	NEG	NEG	POS	POS	POS	POS	NEG	POS	POS	POS	POS	POS	POS	POS	POS	NEG	NEG	NEG	NEG	NEG	NEG	NEG	NEG	NEG	POS	NEG	POS	POS	POS	POS	POS	POS	POS	POS
POS	POS	POS	POS	negative	NEG	NEG	NEG	NEG	NEG	NEG	POS	POS	NEG	POS	POS	POS	POS	NEG	POS	NEG	NEG	NEG	POS	POS	NEG	NEG	NEG	NEG	NEG	NEG	POS	NEG	POS	NEG	NEG	NEG	POS	POS	POS	POS
POS	POS	POS	POS	negative	NEG	NEG	NEG	NEG	NEG	NEG	POS	POS	NEG	POS	POS	POS	POS	POS	POS	NEG	NEG	NEG	POS	POS	NEG	NEG	NEG	NEG	NEG	NEG	POS	NEG	POS	NEG	NEG	NEG	POS	POS	POS	POS
POS	POS	POS	POS	negative	NEG	NEG	NEG	NEG	NEG	NEG	POS	POS	NEG	POS	POS	POS	POS	POS	POS	NEG	NEG	NEG	POS	POS	NEG	NEG	NEG	NEG	NEG	NEG	POS	NEG	POS	NEG	NEG	NEG	POS	POS	POS	POS
POS	POS	POS	POS	negative	NEG	NEG	NEG	NEG	NEG	NEG	POS	POS	NEG	POS	POS	POS	POS	POS	POS	NEG	NEG	NEG	POS	POS	NEG	NEG	NEG	NEG	NEG	NEG	POS	NEG	POS	NEG	NEG	NEG	POS	POS	POS	POS
POS	POS	POS	POS	negative	NEG	NEG	NEG	NEG	NEG	NEG	POS	POS	NEG	POS	POS	POS	POS	POS	POS	NEG	NEG	NEG	POS	POS	NEG	NEG	NEG	NEG	NEG	NEG	POS	NEG	POS	NEG	NEG	NEG	POS	POS	POS	POS
NEG	NEG	NEG	NEG	negative	NEG	NEG	NEG	NEG	NEG	NEG	NEG	NEG	NEG	NEG	NEG	NEG	NEG	NEG	NEG	NEG	NEG	NEG	NEG	POS	NEG	NEG	NEG	NEG	NEG	NEG	NEG	NEG	NEG	NEG	NEG	NEG	NEG	NEG	NEG	NEG
NEG	NEG	NEG	NEG	negative	NEG	NEG	NEG	NEG	NEG	NEG	NEG	NEG	NEG	NEG	NEG	NEG	NEG	NEG	NEG	NEG	NEG	NEG	NEG	POS	NEG	NEG	NEG	NEG	NEG	NEG	NEG	NEG	NEG	NEG	NEG	NEG	NEG	NEG	NEG	NEG

VIRULENCE : STAPHYLOCOCCAL SUPERANTIGEN/ENTEROTOXIN-LIKE GENES (SET/SSL)

setC / selX	ssl01												ssl02		ssl03			ssl04		ssl05				ssl06		ssl07			ssl08		ssl09			ssl10			ssl11				setB3		setB2		setB1
	set6-var1_11	set6-var2_11	set6-var1_12	set6-var2_12	set6-var4_11	ssl01-RF122	ssl01/set6 (COL)	ssl01/set6 (Mu50+N315)	ssl01/set6 (MW2+MSSA476)	ssl01/set6 (MRSA252)	ssl01/set6 (RF122)	ssl01/set6 (other alleles)	ssl02/set7	ssl02/set7 (MRSA252)	ssl03/set8_probe 1	ssl03/set8_probe 2	ssl03/set8 (MRSA252, SAR0424)	ssl04/set9	ssl04/set9 (MRSA252, SAR0425)	ssl05/set3_probe 1	ssl05/set3 (RF122, probe-611)	ssl05/set3_probe 2 (612)	ssl05/set3 (MRSA252)	ssl06/set21	ssl06 (NCTC8325+MW2)	ssl07/set1	ssl07/set1 (MRSA252)	ssl07/set1 (AF188836)	ssl08/set12_probe 1	ssl08/set12_probe 2	ssl09/set5_probe 1	ssl09/set5_probe 2	ssl09/set5 (MRSA252)	ssl10/set4	ssl10 (RF122)	ssl10/set4 (MRSA252)	ssl11/set2 (COL)	ssl11+set2(Mu50+N315)	ssl11+set2(MW2+MSSA476)	ssl11/set2 (MRSA252)	setB3	setB3 (MRSA252)	setB2	setB2 (MRSA252)	
Staphyl. exotoxin-like protein	Staphylococcal superantigen-like protein 1												Staphylococcal superantigen-like protein 2		Staphylococcal superantigen-like protein 3			Staphylococcal superantigen-like protein 4		Staphylococcal superantigen-like protein 5				Staphylococcal superantigen-like protein 6		Staphylococcal superantigen-like protein 7			Staphylococcal superantigen-like protein 8		Staphylococcal superantigen-like protein 9			Staphylococcal superantigen-like protein 10			Staphylococcal superantigene-like protein 11				Staphylococcal exotoxin-like protein, second locus				
POS	NEG	POS	NEG	POS	NEG	NEG	NEG	NEG	POS	NEG	NEG	NEG	POS	NEG	POS	POS	NEG	POS	NEG	POS	AMB	POS	NEG	POS	POS	POS	AMB	NEG	POS	POS	POS	POS	NEG	POS	NEG	AMB	NEG	NEG	POS	NEG	POS	NEG	POS	NEG	POS
POS	NEG	POS	NEG	POS	NEG	NEG	NEG	NEG	POS	NEG	NEG	NEG	POS	NEG	POS	POS	NEG	POS	NEG	POS	AMB	POS	NEG	POS	POS	POS	AMB	NEG	POS	POS	POS	POS	NEG	POS	NEG	AMB	NEG	NEG	POS	NEG	POS	NEG	POS	NEG	POS
POS	NEG	POS	NEG	POS	NEG	NEG	NEG	NEG	POS	NEG	NEG	NEG	POS	NEG	POS	POS	NEG	POS	NEG	POS	NEG	POS	NEG	POS	POS	POS	NEG	NEG	POS	POS	POS	POS	NEG	POS	NEG	NEG	NEG	NEG	POS	NEG	POS	NEG	POS	NEG	POS
POS	NEG	POS	NEG	POS	NEG	NEG	NEG	NEG	POS	NEG	NEG	NEG	POS	NEG	POS	POS	NEG	POS	NEG	POS	NEG	POS	NEG	POS	POS	POS	NEG	NEG	POS	POS	POS	POS	NEG	POS	NEG	NEG	NEG	NEG	POS	NEG	POS	NEG	POS	NEG	POS
POS	NEG	POS	NEG	POS	NEG	NEG	NEG	NEG	POS	NEG	NEG	NEG	POS	NEG	POS	POS	NEG	POS	NEG	NEG	NEG	POS	NEG	NEG	POS	NEG	NEG	NEG	POS	POS	POS	POS	NEG	POS	NEG	NEG	NEG	NEG	POS	NEG	POS	NEG	POS	NEG	POS
POS	POS	NEG	POS	NEG	NEG	NEG	NEG	POS	NEG	NEG	NEG	NEG	POS	NEG	POS	POS	NEG	POS	NEG	POS	NEG	POS	NEG	NEG	NEG	POS	NEG	NEG	POS	POS	POS	POS	NEG	POS	NEG	NEG	NEG	POS	NEG	NEG	POS	NEG	POS	NEG	POS
POS	POS	NEG	POS	NEG	POS	NEG	NEG	POS	NEG	NEG	NEG	NEG	POS	AMB	POS	POS	NEG	POS	NEG	POS	AMB	POS	NEG	NEG	NEG	POS	AMB	NEG	POS	POS	POS	POS	NEG	POS	NEG	AMB	NEG	POS	NEG	NEG	POS	NEG	POS	NEG	POS
POS	POS	NEG	POS	NEG	NEG	NEG	NEG	POS	NEG	NEG	NEG	NEG	POS	NEG	POS	POS	NEG	POS	NEG	POS	NEG	POS	NEG	NEG	NEG	POS	NEG	NEG	POS	POS	POS	POS	NEG	POS	NEG	NEG	NEG	POS	NEG	NEG	POS	NEG	POS	NEG	POS
POS	NEG	NEG	POS	NEG	POS	POS	NEG	NEG	NEG	NEG	NEG	POS	POS	AMB	POS	POS	NEG	POS	NEG	POS	AMB	POS	NEG	NEG	NEG	POS	NEG	NEG	POS	POS	POS	POS	NEG	POS	AMB	AMB	NEG	POS	NEG	NEG	POS	NEG	POS	NEG	POS
POS	NEG	NEG	NEG	NEG	POS	POS	NEG	NEG	NEG	NEG	NEG	POS	POS	NEG	POS	POS	NEG	POS	NEG	POS	POS	AMB	NEG	NEG	NEG	POS	NEG	NEG	POS	POS	POS	POS	NEG	AMB	POS	NEG	NEG	POS	NEG	NEG	POS	NEG	POS	NEG	POS
POS	NEG	NEG	NEG	NEG	POS	POS	NEG	NEG	NEG	NEG	NEG	POS	POS	NEG	POS	POS	NEG	POS	NEG	POS	POS	AMB	NEG	NEG	NEG	POS	NEG	NEG	POS	POS	POS	POS	NEG	POS	AMB	NEG	NEG	POS	NEG	NEG	POS	NEG	POS	NEG	POS
POS	NEG	NEG	AMB	NEG	POS	POS	NEG	NEG	NEG	NEG	NEG	POS	POS	AMB	POS	POS	NEG	POS	NEG	POS	POS	AMB	NEG	NEG	NEG	POS	NEG	NEG	POS	POS	POS	POS	NEG	AMB	POS	AMB	NEG	POS	NEG	NEG	POS	NEG	POS	NEG	POS
POS	NEG	NEG	NEG	NEG	POS	POS	NEG	NEG	NEG	NEG	NEG	POS	POS	NEG	NEG	POS	NEG	POS	NEG	POS	POS	NEG	NEG	NEG	NEG	NEG	NEG	NEG	POS	POS	POS	POS	NEG	AMB	POS	NEG	NEG	POS	NEG	NEG	POS	NEG	POS	NEG	POS
POS	NEG	NEG	NEG	NEG	POS	POS	NEG	NEG	NEG	NEG	NEG	POS	POS	NEG	POS	POS	NEG	POS	NEG	POS	POS	AMB	NEG	NEG	NEG	POS	NEG	NEG	POS	POS	POS	POS	NEG	POS	POS	NEG	NEG	POS	NEG	NEG	POS	NEG	POS	NEG	POS
POS	NEG	NEG	POS	NEG	POS	POS	NEG	NEG	NEG	NEG	NEG	POS	POS	AMB	POS	POS	NEG	POS	NEG	POS	AMB	POS	NEG	NEG	NEG	POS	NEG	NEG	POS	POS	POS	POS	NEG	POS	AMB	AMB	NEG	POS	NEG	NEG	POS	NEG	POS	NEG	POS
POS	NEG	NEG	NEG	NEG	POS	POS	NEG	NEG	NEG	NEG	NEG	POS	POS	NEG	POS	POS	NEG	POS	NEG	POS	POS	AMB	NEG	NEG	NEG	POS	NEG	NEG	POS	POS	POS	POS	NEG	POS	POS	NEG	NEG	POS	NEG	NEG	POS	NEG	POS	NEG	POS
POS	NEG	NEG	NEG	NEG	POS	POS	NEG	NEG	NEG	NEG	NEG	POS	POS	NEG	POS	POS	NEG	POS	NEG	POS	POS	AMB	NEG	NEG	NEG	POS	NEG	NEG	POS	POS	POS	POS	NEG	AMB	POS	NEG	NEG	POS	NEG	NEG	POS	NEG	POS	NEG	POS
POS	NEG	NEG	NEG	NEG	POS	POS	NEG	NEG	NEG	NEG	NEG	POS	POS	NEG	POS	POS	NEG	POS	NEG	POS	POS	AMB	NEG	NEG	NEG	POS	NEG	NEG	POS	POS	POS	POS	NEG	POS	AMB	NEG	NEG	POS	NEG	NEG	POS	NEG	POS	NEG	POS
POS	NEG	NEG	NEG	NEG	POS	POS	NEG	NEG	NEG	NEG	NEG	POS	POS	NEG	POS	POS	NEG	POS	NEG	POS	POS	NEG	NEG	NEG	NEG	NEG	NEG	NEG	POS	POS	POS	POS	NEG	AMB	POS	NEG	NEG	POS	NEG	NEG	POS	NEG	POS	NEG	POS
POS	POS	NEG	NEG	POS	NEG	NEG	NEG	NEG	NEG	NEG	NEG	POS	POS	AMB	POS	POS	NEG	POS	NEG	POS	NEG	POS	NEG	POS	POS	POS	AMB	NEG	POS	POS	POS	POS	NEG	POS	NEG	AMB	NEG	NEG	NEG	NEG	POS	NEG	POS	NEG	POS
POS	POS	NEG	NEG	POS	NEG	NEG	NEG	NEG	NEG	NEG	NEG	POS	POS	AMB	POS	POS	NEG	POS	NEG	POS	NEG	POS	NEG	POS	POS	POS	AMB	AMB	POS	POS	POS	POS	NEG	POS	NEG	AMB	NEG	NEG	NEG	NEG	POS	NEG	POS	NEG	POS
POS	POS	NEG	NEG	POS	NEG	NEG	NEG	NEG	NEG	NEG	NEG	POS	POS	NEG	POS	POS	NEG	POS	NEG	POS	NEG	POS	NEG	POS	POS	POS	AMB	NEG	POS	POS	POS	POS	NEG	POS	NEG	AMB	NEG	NEG	NEG	NEG	POS	NEG	POS	NEG	POS
POS	POS	NEG	NEG	POS	NEG	NEG	NEG	NEG	NEG	NEG	NEG	POS	POS	AMB	POS	POS	NEG	POS	NEG	POS	NEG	POS	NEG	POS	POS	POS	AMB	NEG	POS	POS	POS	POS	NEG	POS	NEG	AMB	NEG	NEG	NEG	NEG	POS	NEG	POS	NEG	POS
POS	POS	NEG	NEG	POS	NEG	NEG	NEG	NEG	NEG	NEG	NEG	POS	POS	AMB	NEG	NEG	NEG	POS	NEG	POS	NEG	POS	NEG	POS	POS	POS	AMB	NEG	POS	POS	POS	POS	NEG	POS	NEG	AMB	NEG	NEG	NEG	NEG	POS	NEG	POS	NEG	POS
POS	POS	NEG	AMB	NEG	POS	NEG	POS	AMB	NEG	NEG	NEG	NEG	POS	NEG	POS	POS	NEG	POS	NEG	POS	AMB	POS	NEG	POS	POS	POS	AMB	NEG	POS	POS	POS	POS	NEG	POS	NEG	AMB	POS	NEG	NEG	NEG	POS	NEG	POS	NEG	POS
POS	POS	NEG	NEG	NEG	POS	NEG	POS	AMB	NEG	NEG	NEG	NEG	NEG	NEG	POS	POS	NEG	POS	NEG	POS	NEG	POS	NEG	NEG	POS	POS	NEG	NEG	POS	POS	POS	POS	NEG	POS	NEG	NEG	POS	NEG	NEG	NEG	POS	NEG	POS	NEG	POS
POS	POS	NEG	NEG	NEG	POS	NEG	POS	AMB	NEG	NEG	NEG	NEG	POS	NEG	POS	POS	NEG	POS	NEG	POS	NEG	POS	NEG	POS	POS	POS	AMB	NEG	POS	POS	POS	POS	NEG	POS	NEG	AMB	POS	NEG	NEG	NEG	POS	NEG	POS	NEG	POS
POS	POS	NEG	NEG	NEG	POS	NEG	POS	AMB	NEG	NEG	NEG	NEG	POS	NEG	POS	POS	NEG	POS	NEG	POS	NEG	POS	NEG	POS	POS	POS	AMB	NEG	POS	POS	POS	POS	NEG	POS	NEG	NEG	POS	NEG	NEG	NEG	POS	NEG	POS	NEG	POS
POS	POS	NEG	NEG	NEG	POS	NEG	POS	AMB	NEG	NEG	NEG	NEG	POS	NEG	POS	POS	NEG	POS	NEG	POS	NEG	POS	NEG	POS	POS	POS	AMB	NEG	POS	POS	POS	POS	NEG	POS	NEG	AMB	POS	NEG	NEG	NEG	POS	NEG	POS	NEG	POS
POS	POS	NEG	AMB	NEG	POS	NEG	POS	AMB	NEG	NEG	NEG	NEG	POS	NEG	POS	POS	NEG	POS	NEG	POS	AMB	POS	NEG	POS	POS	POS	AMB	NEG	POS	POS	POS	POS	NEG	POS	NEG	AMB	POS	NEG	NEG	NEG	POS	NEG	POS	NEG	POS
POS	POS	NEG	AMB	NEG	POS	NEG	POS	AMB	NEG	NEG	NEG	NEG	POS	NEG	POS	POS	NEG	POS	NEG	POS	AMB	POS	NEG	POS	POS	POS	AMB	NEG	POS	POS	POS	POS	NEG	POS	NEG	AMB	POS	NEG	NEG	NEG	POS	NEG	POS	NEG	POS
POS	POS	NEG	AMB	NEG	POS	NEG	POS	AMB	NEG	NEG	NEG	NEG	POS	NEG	POS	POS	NEG	POS	NEG	POS	AMB	POS	NEG	POS	POS	POS	AMB	NEG	POS	POS	POS	POS	NEG	POS	NEG	AMB	POS	NEG	NEG	NEG	POS	NEG	POS	NEG	POS
POS	POS	NEG	AMB	NEG	POS	NEG	POS	AMB	NEG	NEG	NEG	NEG	POS	AMB	POS	POS	NEG	POS	NEG	POS	AMB	POS	NEG	POS	POS	POS	AMB	NEG	POS	POS	POS	POS	NEG	POS	AMB	AMB	POS	NEG	NEG	NEG	POS	NEG	POS	NEG	POS
POS	POS	NEG	POS	NEG	POS	NEG	POS	AMB	NEG	NEG	NEG	NEG	POS	NEG	POS	POS	NEG	POS	NEG	POS	NEG	POS	NEG	POS	POS	POS	AMB	NEG	POS	POS	POS	POS	NEG	POS	NEG	NEG	POS	NEG	NEG	NEG	POS	NEG	POS	NEG	POS
POS	POS	NEG	AMB	NEG	POS	NEG	POS	AMB	NEG	NEG	NEG	NEG	POS	NEG	POS	POS	NEG	POS	NEG	POS	AMB	POS	NEG	POS	POS	POS	AMB	NEG	POS	POS	POS	POS	NEG	POS	NEG	AMB	POS	NEG	NEG	NEG	POS	NEG	POS	NEG	POS
POS	POS	NEG	POS	NEG	POS	NEG	POS	AMB	NEG	NEG	NEG	NEG	POS	AMB	POS	POS	NEG	POS	NEG	POS	AMB	POS	NEG	POS	POS	POS	AMB	NEG	POS	POS	POS	POS	NEG	POS	NEG	AMB	POS	NEG	NEG	NEG	POS	NEG	POS	NEG	POS
POS	POS	NEG	AMB	NEG	POS	NEG	POS	AMB	NEG	NEG	NEG	NEG	POS	NEG	POS	POS	NEG	POS	NEG	POS	AMB	POS	NEG	POS	POS	POS	AMB	NEG	POS	POS	POS	POS	NEG	POS	NEG	AMB	POS	NEG	NEG	NEG	POS	NEG	POS	NEG	POS
POS	POS	NEG	NEG	NEG	POS	NEG	POS	AMB	NEG	NEG	NEG	NEG	POS	NEG	POS	NEG	NEG	POS	NEG	POS	NEG	POS	NEG	NEG	POS	POS	NEG	NEG	POS	POS	POS	POS	NEG	POS	NEG	NEG	POS	NEG	NEG	NEG	POS	NEG	POS	NEG	POS
POS	POS	NEG	AMB	NEG	POS	NEG	POS	AMB	NEG	NEG	NEG	NEG	POS	NEG	POS	POS	NEG	POS	NEG	POS	AMB	POS	NEG	POS	POS	POS	AMB	NEG	POS	POS	POS	POS	NEG	POS	NEG	AMB	POS	NEG	NEG	NEG	POS	NEG	POS	NEG	POS
POS	NEG	POS	NEG	POS	NEG	POS	NEG	NEG	POS	NEG	NEG	NEG	POS	NEG	POS	POS	NEG	POS	NEG	NEG	AMB	POS	NEG	POS	POS	POS	AMB	AMB	POS	POS	POS	POS	NEG	POS	NEG	AMB	NEG	NEG	NEG	NEG	POS	NEG	POS	NEG	POS
POS	NEG	NEG	NEG	NEG	POS	POS	NEG	NEG	NEG	NEG	NEG	POS	POS	NEG	POS	POS	NEG	POS	NEG	POS	AMB	POS	NEG	POS	POS	POS	NEG	NEG	POS	POS	NEG	NEG	NEG	POS	NEG	AMB	POS	NEG	NEG	NEG	POS	NEG	POS	NEG	POS
POS	NEG	AMB	NEG	NEG	POS	NEG	NEG	NEG	NEG	NEG	NEG	POS	POS	NEG	POS	POS	NEG	POS	NEG	POS	POS	AMB	NEG	POS	POS	POS	AMB	NEG	POS	POS	POS	POS	NEG	AMB	POS	NEG	NEG	NEG	NEG	POS	POS	NEG	POS	NEG	POS
POS	NEG	POS	NEG	NEG	POS	NEG	NEG	NEG	NEG	NEG	NEG	POS	POS	NEG	POS	POS	NEG	POS	NEG	POS	POS	AMB	NEG	POS	POS	POS	AMB	NEG	POS	POS	POS	POS	NEG	AMB	POS	NEG	NEG	NEG	NEG	POS	POS	NEG	POS	NEG	POS
POS	NEG	POS	NEG	NEG	POS	NEG	NEG	NEG	NEG	NEG	NEG	POS	POS	AMB	POS	POS	NEG	POS	NEG	POS	POS	AMB	NEG	POS	POS	POS	AMB	NEG	POS	POS	POS	POS	NEG	POS	AMB	NEG	NEG	NEG	NEG	POS	POS	NEG	POS	NEG	POS
POS	NEG	POS	POS	NEG	NEG	NEG	NEG	NEG	NEG	POS	NEG	NEG	POS	AMB	NEG	NEG	POS	NEG	POS	POS	NEG	NEG	POS	NEG	NEG	AMB	AMB	POS	NEG	NEG	POS	POS	NEG	POS	NEG	NEG	NEG	NEG	NEG	NEG	NEG	NEG	NEG	NEG	NEG
NEG	NEG	POS	POS	NEG	NEG	NEG	NEG	NEG	NEG	POS	NEG	NEG	NEG	POS	NEG	NEG	POS	NEG	POS	NEG	NEG	NEG	POS	NEG	NEG	NEG	POS	NEG	NEG	NEG	NEG	NEG	POS	NEG	NEG	POS	NEG	NEG	NEG	NEG	NEG	POS	NEG	POS	AMB
POS	NEG	POS	AMB	NEG	POS	NEG	NEG	NEG	NEG	NEG	NEG	POS	AMB	POS	NEG	NEG	NEG	NEG	POS	NEG	NEG	NEG	POS	NEG	NEG	NEG	AMB	POS	NEG	NEG	NEG	NEG	POS	AMB	NEG	POS	NEG	NEG	NEG	NEG	NEG	POS	NEG	AMB	NEG
POS	NEG	POS	POS	NEG	POS	NEG	NEG	NEG	NEG	POS	NEG	NEG	AMB	POS	NEG	NEG	NEG	NEG	POS	NEG	NEG	NEG	POS	NEG	AMB	NEG	AMB	POS	NEG	NEG	NEG	NEG	POS	AMB	NEG	POS	NEG	NEG	NEG	NEG	NEG	POS	NEG	NEG	NEG
AMB	NEG	POS	NEG	NEG	NEG	NEG	NEG	NEG	NEG	NEG	NEG	POS	NEG	POS	NEG	NEG	NEG	NEG	POS	NEG	NEG	NEG	POS	NEG	NEG	NEG	AMB	POS	NEG	NEG	NEG	NEG	POS	AMB	NEG	POS	NEG	NEG	NEG	NEG	NEG	POS	NEG	POS	NEG
POS	NEG	POS	POS	NEG	POS	NEG	NEG	NEG	NEG	POS	NEG	NEG	AMB	POS	NEG	NEG	NEG	NEG	POS	NEG	NEG	NEG	POS	NEG	AMB	NEG	AMB	POS	NEG	NEG	NEG	NEG	POS	AMB	NEG	POS	NEG	NEG	NEG	NEG	NEG	POS	NEG	NEG	NEG
POS	POS	NEG	POS	NEG	NEG	NEG	NEG	POS	NEG	NEG	NEG	NEG	POS	NEG	POS	POS	NEG	POS	NEG	POS	NEG	POS	NEG	NEG	NEG	POS	NEG	NEG	POS	POS	POS	POS	NEG	AMB	POS	NEG	NEG	NEG	NEG	NEG	POS	NEG	POS	NEG	POS
POS	POS	NEG	NEG	NEG	NEG	NEG	NEG	NEG	NEG	NEG	NEG	POS	POS	NEG	POS	POS	NEG	POS	NEG	POS	POS	AMB	NEG	NEG	NEG	POS	AMB	NEG	POS	POS	POS	POS	NEG	POS	NEG	NEG	NEG	NEG	NEG	NEG	POS	NEG	POS	NEG	POS
POS	POS	NEG	NEG	NEG	NEG	NEG	NEG	NEG	NEG	NEG	NEG	POS	POS	AMB	POS	POS	NEG	POS	NEG	POS	POS	AMB	NEG	NEG	NEG	POS	AMB	NEG	POS	POS	POS	POS	NEG	POS	NEG	AMB	NEG	NEG	NEG	NEG	POS	NEG	POS	NEG	POS
POS	POS	NEG	NEG	NEG	NEG	NEG	NEG	NEG	NEG	NEG	NEG	POS	POS	NEG	POS	POS	NEG	POS	NEG	POS	POS	NEG	NEG	NEG	NEG	POS	NEG	NEG	POS	POS	POS	POS	NEG	POS	NEG	NEG	NEG	NEG	NEG	NEG	POS	NEG	POS	NEG	POS
POS	POS	NEG	NEG	NEG	NEG	NEG	NEG	NEG	NEG	NEG	NEG	POS	POS	NEG	POS	POS	NEG	POS	NEG	POS	POS	AMB	NEG	NEG	NEG	POS	AMB	AMB	POS	POS	POS	POS	NEG	POS	NEG	AMB	NEG	NEG	NEG	NEG	POS	NEG	POS	NEG	POS
POS	POS	NEG	NEG	NEG	NEG	NEG	NEG	NEG	NEG	NEG	NEG	POS	POS	NEG	POS	POS	NEG	POS	NEG	POS	POS	AMB	NEG	NEG	NEG	POS	AMB	NEG	POS	POS	POS	POS	NEG	POS	NEG	AMB	NEG	NEG	NEG	NEG	POS	NEG	POS	NEG	POS
POS	NEG	POS	NEG	NEG	POS	NEG	NEG	NEG	NEG	NEG	NEG	POS	POS	AMB	POS	POS	NEG	NEG	NEG	NEG	AMB	POS	NEG	POS	POS	POS	AMB	NEG	POS	POS	POS	POS	NEG	POS	NEG	AMB	NEG	NEG	NEG	NEG	POS	NEG	POS	NEG	POS
POS	NEG	NEG	NEG	NEG	NEG	POS	NEG	NEG	NEG	NEG	POS	NEG	POS	NEG	POS	POS	NEG	POS	NEG	POS	POS	AMB	NEG	NEG	NEG	POS	AMB	NEG	POS	POS	POS	POS	NEG	POS	NEG	AMB	NEG	NEG	NEG	NEG	POS	NEG	POS	NEG	POS
POS	NEG	NEG	NEG	NEG	NEG	POS	NEG	NEG	NEG	NEG	POS	NEG	POS	AMB	POS	POS	NEG	POS	NEG	POS	POS	AMB	NEG	NEG	NEG	POS	AMB	NEG	POS	POS	POS	POS	NEG	POS	NEG	AMB	NEG	NEG	NEG	NEG	POS	NEG	POS	NEG	POS
POS	NEG	NEG	NEG	NEG	NEG	POS	NEG	NEG	NEG	NEG	POS	NEG	POS	AMB	POS	POS	NEG	POS	NEG	POS	POS	AMB	NEG	NEG	NEG	POS	AMB	AMB	POS	POS	POS	POS	NEG	POS	NEG	AMB	NEG	NEG	NEG	NEG	POS	NEG	POS	NEG	POS
POS	NEG	NEG	NEG	NEG	NEG	POS	NEG	NEG	NEG	NEG	POS	NEG	POS	NEG	POS	POS	NEG	POS	NEG	POS	POS	AMB	NEG	NEG	NEG	POS	AMB	NEG	POS	POS	POS	POS	NEG	POS	NEG	AMB	NEG	NEG	NEG	NEG	POS	NEG	POS	NEG	POS
POS	NEG	NEG	NEG	NEG	NEG	POS	NEG	NEG	NEG	NEG	POS	NEG	POS	NEG	POS	POS	NEG	POS	NEG	POS	POS	AMB	NEG	NEG	NEG	POS	AMB	AMB	POS	POS	POS	POS	NEG	POS	NEG	AMB	NEG	NEG	NEG	NEG	POS	NEG	POS	NEG	POS
POS	NEG	NEG	NEG	NEG	NEG	POS	NEG	NEG	NEG	NEG	POS	NEG	POS	NEG	POS	POS	NEG	POS	NEG	POS	POS	AMB	NEG	NEG	NEG	POS	AMB	NEG	POS	POS	POS	POS	NEG	POS	NEG	AMB	NEG	NEG	NEG	NEG	POS	NEG	POS	NEG	POS
POS	NEG	NEG	NEG	NEG	NEG	POS	NEG	NEG	NEG	NEG	POS	NEG	POS	NEG	POS	POS	NEG	POS	NEG	POS	POS	AMB	NEG	NEG	NEG	POS	AMB	NEG	POS	POS	POS	POS	NEG	POS	NEG	AMB	NEG	NEG	NEG	NEG	POS	NEG	POS	NEG	POS
POS	NEG	NEG	NEG	NEG	NEG	POS	NEG	NEG	NEG	NEG	POS	NEG	POS	NEG	NEG	NEG	NEG	POS	NEG	POS	POS	NEG	NEG	NEG	NEG	POS	NEG	NEG	POS	POS	POS	POS	NEG	POS	NEG	NEG	NEG	NEG	NEG	NEG	POS	NEG	POS	NEG	POS
POS	NEG	NEG	NEG	NEG	NEG	POS	NEG	NEG	NEG	NEG	POS	NEG	POS	NEG	POS	POS	NEG	POS	NEG	POS	POS	NEG	NEG	NEG	NEG	POS	NEG	NEG	POS	POS	POS	POS	NEG	POS	NEG	NEG	NEG	NEG	NEG	NEG	POS	NEG	POS	NEG	POS
POS	NEG	NEG	NEG	NEG	NEG	POS	NEG	NEG	NEG	NEG	POS	NEG	POS	NEG	POS	POS	NEG	POS	NEG	POS	POS	NEG	NEG	NEG	NEG	POS	NEG	NEG	POS	POS	POS	POS	NEG	POS	NEG	NEG	NEG	NEG	NEG	NEG	POS	NEG	POS	NEG	POS
POS	NEG	POS	NEG	POS	NEG	NEG	NEG	NEG	POS	NEG	NEG	NEG	POS	NEG	POS	POS	NEG	POS	NEG	NEG	NEG	POS	NEG	NEG	NEG	POS	AMB	NEG	POS	POS	POS	POS	NEG	POS	NEG	AMB	NEG	NEG	NEG	NEG	POS	NEG	POS	NEG	POS
POS	NEG	POS	NEG	POS	NEG	NEG	NEG	NEG	POS	NEG	NEG	NEG	POS	NEG	POS	POS	NEG	POS	NEG	NEG	NEG	POS	NEG	NEG	NEG	POS	AMB	NEG	POS	POS	POS	POS	NEG	POS	NEG	AMB	NEG	NEG	NEG	NEG	POS	NEG	POS	NEG	POS
POS	POS	NEG	POS	NEG	POS	NEG	NEG	POS	NEG	NEG	NEG	NEG	NEG	NEG	POS	POS	NEG	POS	NEG	NEG	POS	AMB	NEG	POS	POS	POS	NEG	NEG	POS	POS	POS	POS	NEG	AMB	POS	NEG	NEG	NEG	NEG	NEG	POS	NEG	POS	NEG	POS
NEG	NEG	NEG	NEG	POS	NEG	POS	NEG	NEG	NEG	NEG	POS	NEG	AMB	POS	NEG	NEG	NEG	NEG	NEG	NEG	NEG	NEG	NEG	NEG	NEG	NEG	NEG	NEG	NEG	NEG	NEG	NEG	NEG	POS	NEG	NEG	NEG	NEG	NEG	NEG	POS	NEG	POS	NEG	POS
NEG	NEG	NEG	NEG	POS	NEG	POS	NEG	NEG	NEG	NEG	POS	NEG	AMB	POS	NEG	NEG	NEG	NEG	NEG	NEG	NEG	NEG	NEG	NEG	NEG	NEG	NEG	NEG	NEG	NEG	NEG	NEG	NEG	NEG	NEG	NEG	NEG	NEG	NEG	NEG	POS	NEG	POS	NEG	POS
NEG	NEG	NEG	NEG	POS	NEG	POS	NEG	NEG	NEG	NEG	POS	NEG	AMB	POS	NEG	NEG	NEG	NEG	NEG	NEG	NEG	NEG	NEG	NEG	NEG	NEG	NEG	NEG	NEG	NEG	NEG	NEG	NEG	NEG	NEG	NEG	NEG	NEG	NEG	NEG	POS	NEG	POS	NEG	POS
NEG	NEG	NEG	NEG	POS	NEG	POS	NEG	NEG	NEG	NEG	POS	NEG	AMB	POS	NEG	NEG	NEG	NEG	NEG	NEG	NEG	NEG	NEG	NEG	NEG	NEG	NEG	NEG	NEG	NEG	NEG	NEG	NEG	POS	NEG	NEG	NEG	NEG	NEG	NEG	POS	NEG	POS	NEG	POS
NEG	NEG	NEG	NEG	POS	NEG	POS	NEG	NEG	NEG	NEG	POS	NEG	AMB	POS	NEG	NEG	NEG	NEG	NEG	NEG	NEG	NEG	NEG	NEG	NEG	NEG	NEG	NEG	NEG	NEG	NEG	NEG	NEG	POS	NEG	NEG	NEG	NEG	NEG	NEG	POS	NEG	POS	NEG	POS
NEG	NEG	NEG	NEG	POS	NEG	POS	NEG	NEG	NEG	NEG	POS	NEG	AMB	POS	NEG	NEG	NEG	NEG	NEG	NEG	NEG	NEG	NEG	NEG	NEG	NEG	NEG	NEG	NEG	NEG	NEG	NEG	NEG	POS	NEG	NEG	NEG	NEG	NEG	NEG	POS	NEG	POS	NEG	POS
NEG	NEG	NEG	NEG	POS	NEG	POS	NEG	NEG	NEG	NEG	POS	NEG	POS	AMB	NEG	NEG	NEG	NEG	NEG	NEG	NEG	NEG	NEG	NEG	NEG	NEG	NEG	NEG	NEG	NEG	NEG	NEG	NEG	POS	NEG	NEG	NEG	NEG	NEG	NEG	POS	NEG	POS	NEG	POS
NEG	NEG	NEG	NEG	POS	NEG	POS	NEG	NEG	NEG	NEG	POS	NEG	AMB	POS	NEG	NEG	NEG	NEG	NEG	NEG	NEG	NEG	NEG	NEG	NEG	NEG	NEG	NEG	NEG	NEG	NEG	NEG	NEG	POS	NEG	NEG	NEG	NEG	NEG	NEG	POS	NEG	POS	NEG	POS
NEG	NEG	NEG	NEG	POS	NEG	POS	NEG	NEG	NEG	NEG	POS	NEG	POS	AMB	NEG	NEG	NEG	NEG	NEG	NEG	NEG	NEG	NEG	NEG	NEG	NEG	NEG	NEG	NEG	NEG	NEG	NEG	NEG	NEG	NEG	NEG	NEG	NEG	NEG	NEG	POS	NEG	POS	NEG	POS
NEG	NEG	NEG	NEG	POS	NEG	POS	NEG	NEG	NEG	NEG	POS	NEG	AMB	POS	NEG	NEG	NEG	NEG	NEG	NEG	NEG	NEG	NEG	NEG	NEG	NEG	NEG	NEG	NEG	NEG	NEG	NEG	NEG	POS	NEG	NEG	NEG	NEG	NEG	NEG	POS	NEG	POS	NEG	POS
NEG	NEG	NEG	NEG	POS	NEG	POS	NEG	NEG	NEG	NEG	POS	NEG	AMB	POS	NEG	NEG	NEG	NEG	NEG	NEG	NEG	NEG	NEG	NEG	NEG	NEG	NEG	NEG	NEG	NEG	NEG	NEG	NEG	POS	NEG	NEG	NEG	NEG	NEG	NEG	POS	NEG	POS	NEG	POS
NEG	NEG	NEG	NEG	POS	NEG	POS	NEG	NEG	NEG	NEG	POS	NEG	NEG	NEG	NEG	NEG	NEG	NEG	NEG	NEG	NEG	NEG	NEG	NEG	NEG	NEG	NEG	NEG	NEG	NEG	NEG	NEG	NEG	NEG	NEG	NEG	NEG	NEG	NEG	NEG	POS	NEG	POS	NEG	POS
NEG	NEG	NEG	NEG	POS	NEG	POS	NEG	NEG	NEG	NEG	POS	NEG	POS	AMB	NEG	NEG	NEG	NEG	NEG	NEG	NEG	NEG	NEG	NEG	NEG	NEG	NEG	NEG	NEG	NEG	NEG	NEG	NEG	POS	NEG	NEG	NEG	NEG	NEG	NEG	POS	NEG	POS	NEG	POS
NEG	NEG	NEG	NEG	POS	NEG	POS	NEG	NEG	NEG	NEG	POS	NEG	AMB	POS	NEG	NEG	NEG	NEG	NEG	NEG	NEG	NEG	NEG	NEG	NEG	NEG	NEG	NEG	NEG	NEG	NEG	NEG	NEG	POS	NEG	NEG	NEG	NEG	NEG	NEG	POS	NEG	POS	NEG	POS
NEG	NEG	NEG	NEG	POS	NEG	POS	NEG	NEG	NEG	NEG	POS	NEG	AMB	POS	NEG	NEG	NEG	NEG	NEG	NEG	NEG	NEG	NEG	NEG	NEG	NEG	NEG	NEG	NEG	NEG	NEG	NEG	NEG	POS	NEG	NEG	NEG	NEG	NEG	NEG	POS	NEG	POS	NEG	POS
NEG	NEG	NEG	NEG	POS	NEG	POS	NEG	NEG	NEG	NEG	POS	NEG	AMB	POS	NEG	NEG	NEG	NEG	NEG	NEG	NEG	NEG	NEG	NEG	NEG	NEG	NEG	NEG	NEG	NEG	NEG	NEG	NEG	POS	NEG	NEG	NEG	NEG	NEG	NEG	POS	NEG	POS	NEG	POS
POS	NEG	POS	NEG	POS	NEG	NEG	NEG	NEG	POS	NEG	NEG	NEG	POS	NEG	POS	POS	NEG	POS	NEG	POS	AMB	POS	NEG	NEG	NEG	POS	AMB	NEG	POS	POS	POS	POS	NEG	POS	NEG	AMB	NEG	NEG	POS	NEG	POS	NEG	POS	NEG	POS
POS	POS	NEG	POS	NEG	POS	NEG	POS	AMB	NEG	NEG	NEG	NEG	POS	AMB	POS	POS	NEG	POS	NEG	POS	AMB	POS	NEG	POS	POS	POS	AMB	AMB	POS	POS	POS	POS	NEG	POS	NEG	AMB	POS	NEG	NEG	NEG	POS	NEG	POS	NEG	POS
POS	POS	NEG	POS	NEG	POS	NEG	POS	AMB	NEG	NEG	NEG	NEG	POS	AMB	POS	POS	NEG	POS	NEG	POS	AMB	POS	NEG	POS	POS	POS	AMB	AMB	POS	POS	POS	POS	NEG	POS	NEG	AMB	POS	NEG	NEG	NEG	POS	NEG	POS	NEG	POS
POS	POS	NEG	NEG	NEG	POS	NEG	POS	AMB	NEG	NEG	NEG	NEG	POS	NEG	POS	POS	NEG	POS	NEG	POS	NEG	POS	NEG	POS	POS	POS	AMB	AMB	POS	POS	POS	POS	NEG	POS	NEG	NEG	POS	NEG	NEG	NEG	POS	NEG	POS	NEG	POS
POS	POS	NEG	NEG	NEG	POS	NEG	POS	AMB	NEG	NEG	NEG	NEG	POS	NEG	POS	POS	NEG	POS	NEG	POS	AMB	POS	NEG	POS	POS	POS	AMB	NEG	POS	POS	POS	POS	NEG	POS	NEG	AMB	POS	NEG	NEG	NEG	POS	NEG	POS	NEG	POS
POS	POS	NEG	AMB	NEG	POS	NEG	POS	AMB	NEG	NEG	NEG	NEG	POS	AMB	POS	POS	NEG	POS	NEG	POS	AMB	POS	NEG	POS	POS	POS	AMB	AMB	POS	POS	POS	POS	NEG	POS	NEG	AMB	POS	NEG	NEG	NEG	POS	NEG	POS	NEG	POS
POS	POS	NEG	AMB	NEG	POS	NEG	POS	AMB	NEG	NEG	NEG	NEG	POS	NEG	POS	POS	NEG	POS	NEG	POS	AMB	POS	NEG	POS	POS	POS	AMB	NEG	POS	POS	POS	POS	NEG	POS	NEG	AMB	POS	NEG	NEG	NEG	POS	NEG	POS	NEG	POS
POS	POS	NEG	POS	NEG	POS	NEG	POS	AMB	NEG	NEG	NEG	NEG	POS	AMB	POS	POS	NEG	POS	NEG	POS	AMB	POS	NEG	POS	POS	POS	AMB	AMB	POS	POS	POS	POS	NEG	POS	NEG	AMB	POS	NEG	NEG	NEG	POS	NEG	POS	NEG	POS
POS	POS	NEG	POS	NEG	POS	NEG	NEG	POS	NEG	NEG	NEG	NEG	NEG	POS	NEG	NEG	NEG	NEG	POS	NEG	NEG	NEG	POS	NEG	NEG	AMB	AMB	POS	NEG	NEG	NEG	NEG	POS	AMB	NEG	POS	NEG	NEG	NEG	POS	NEG	POS	NEG	POS	POS
POS	POS	NEG	POS	NEG	POS	NEG	NEG	POS	NEG	NEG	NEG	NEG	AMB	POS	NEG	NEG	NEG	NEG	POS	NEG	NEG	NEG	POS	NEG	NEG	AMB	AMB	POS	NEG	NEG	NEG	NEG	POS	AMB	NEG	POS	NEG	NEG	NEG	POS	NEG	POS	NEG	POS	POS
POS	POS	NEG	POS	NEG	POS	NEG	NEG	POS	NEG	NEG	NEG	NEG	AMB	POS	NEG	NEG	NEG	NEG	POS	NEG	NEG	NEG	POS	NEG	NEG	AMB	AMB	POS	NEG	NEG	NEG	NEG	POS	AMB	NEG	POS	NEG	NEG	NEG	POS	NEG	POS	NEG	POS	POS
POS	POS	NEG	POS	NEG	POS	NEG	NEG	POS	NEG	NEG	NEG	NEG	AMB	POS	NEG	NEG	NEG	NEG	POS	NEG	NEG	NEG	POS	NEG	NEG	AMB	AMB	POS	NEG	NEG	NEG	NEG	POS	AMB	NEG	POS	NEG	NEG	NEG	AMB	NEG	POS	NEG	POS	POS
POS	POS	NEG	POS	NEG	POS	NEG	NEG	POS	NEG	NEG	NEG	NEG	AMB	POS	NEG	NEG	NEG	NEG	POS	NEG	NEG	NEG	POS	NEG	AMB	AMB	AMB	POS	NEG	NEG	NEG	NEG	POS	AMB	NEG	POS	NEG	NEG	NEG	POS	NEG	POS	NEG	POS	POS
POS	NEG	POS	NEG	NEG	POS	POS	NEG	NEG	NEG	NEG	NEG	POS	NEG	NEG	NEG	NEG	NEG	AMB	POS	NEG	NEG	NEG	NEG	NEG	POS	NEG	NEG	NEG	NEG	NEG	NEG	NEG	NEG	POS	NEG	NEG	NEG	NEG	NEG	NEG	NEG	NEG	NEG	NEG	NEG
POS	NEG	POS	NEG	NEG	AMB	POS	NEG	NEG	NEG	NEG	NEG	POS	NEG	NEG	NEG	NEG	NEG	AMB	AMB	NEG	NEG	NEG	NEG	NEG	POS	NEG	NEG	NEG	NEG	NEG	NEG	NEG	NEG	NEG	NEG	NEG	NEG	NEG	NEG	NEG	NEG	NEG	NEG	NEG	NEG

CAPSULE- AND BIOFILM-ASSOCIATED GENES																	ADHAESION FACTORS / GENES ENCODING MICROBIAL SURFACE COMPONENTS RECOGNIZING ADHESIVE MATRIX MOLECULES (MSCRAMM GENES)																																
Capsule type 1				Capsule type 5				Capsule type 8					icaA	icaC	icaD	bap	bbp							clfA					clfB					cna	ebh	ebpS					eno	efb		fnbA					
cap 1	capH1	capJ1	capK1	cap 5	capH5	capJ5	capK5	cap 8	capH8	capI8	capJ8	capK8	icaA	icaC	icaD	bap	bbp	bbp (cons)	bbp (COL+MW2)	bbp (MRSA252)	bbp (Mu50)	bbp (RF122)	bbp (ST45)	clfA	clfA (cons)	clfA (COL+ RF122)	clfA (MRSA252)	clfA (Mu50+ MW2)	clfB	clfB (cons)	clfB (COL+Mu50)	clfB (MW2)	clfB (RF122)			ebpS	ebpS_probe 612	ebpS_probe 614	ebpS (01-1111)	ebpS (COL)		efb / fib	efb / fib (MRSA252)	fnbA	fnbA (cons)	fnbA (COL)	fnbA (MRSA252)	fnbA (Mu50+ MW2)	fnbA (RF122)
Capsule type 1	capsular poly-saccharide synthesis enzyme	O-antigen polymerase	capsular poly-saccharide biosyn- thesis protein	Capsule type 5	capsular poly-saccharide synthesis enzyme	O-antigen polymerase	capsular poly-saccharide biosyn- thesis protein	Capsule type 8	capsular poly-saccharide synthesis enzyme	capsular poly-saccharide biosyn- thesis protein	O-antigen poly-merase	capsular poly-saccharide biosyn- thesis protein	intercellular adhesion protein A	intercellular adhesion protein C	biofilm PIA synthesis protein D	Surface protein involved in biofilm formation	Bone sialoprotein-binding protein							Clumping factor A					Clumping factor B					Collagen-binding adhesin	Cell wall associated fibronectin-binding protein	cell surface elastin binding protein					enolase	fibrinogen binding protein (19 kDa)		fibronectin-binding protein A					
NEG	NEG	NEG	NEG	NEG	NEG	NEG	NEG	POS	POS	POS	POS	POS	POS	POS	POS	NEG	POS	POS	POS	NEG	NEG	NEG	NEG	POS	POS	NEG	NEG	POS	POS	POS	NEG	POS	AMB	POS	POS	POS	POS	POS	NEG	NEG	POS	POS	NEG	POS	POS	NEG	NEG	POS	NEG
NEG	NEG	NEG	NEG	NEG	NEG	NEG	NEG	POS	POS	POS	POS	POS	POS	POS	POS	NEG	POS	POS	POS	NEG	NEG	NEG	NEG	POS	POS	NEG	NEG	POS	POS	POS	NEG	POS	AMB	POS	POS	POS	POS	POS	NEG	NEG	POS	POS	NEG	POS	POS	NEG	NEG	POS	NEG
NEG	NEG	NEG	NEG	NEG	NEG	NEG	NEG	POS	POS	POS	POS	POS	POS	POS	POS	NEG	POS	POS	POS	NEG	NEG	NEG	NEG	POS	POS	NEG	NEG	POS	POS	POS	NEG	POS	NEG	POS	POS	POS	POS	POS	NEG	NEG	POS	POS	NEG	POS	POS	NEG	NEG	POS	NEG
NEG	NEG	NEG	NEG	NEG	NEG	NEG	NEG	POS	POS	POS	POS	POS	POS	POS	POS	NEG	POS	POS	POS	NEG	NEG	NEG	NEG	POS	POS	NEG	NEG	POS	POS	POS	NEG	POS	NEG	POS	POS	POS	POS	POS	NEG	NEG	POS	POS	NEG	POS	POS	NEG	NEG	POS	NEG
NEG	NEG	NEG	NEG	NEG	NEG	NEG	NEG	POS	NEG	POS	POS	POS	POS	POS	POS	NEG	NEG	NEG	NEG	NEG	NEG	NEG	NEG	POS	POS	NEG	NEG	POS	POS	POS	NEG	POS	NEG	POS	POS	POS	NEG	POS	NEG	NEG	POS	POS	NEG	POS	POS	NEG	NEG	POS	NEG
NEG	NEG	NEG	NEG	POS	POS	POS	POS	NEG	NEG	NEG	NEG	NEG	POS	POS	POS	NEG	POS	POS	NEG	NEG	POS	NEG	NEG	POS	POS	NEG	NEG	POS	POS	POS	POS	NEG	NEG	NEG	POS	POS	POS	POS	NEG	NEG	POS	POS	NEG	POS	POS	NEG	NEG	POS	NEG
NEG	NEG	NEG	NEG	POS	POS	POS	POS	NEG	NEG	NEG	NEG	NEG	POS	POS	POS	NEG	POS	POS	NEG	NEG	POS	NEG	NEG	POS	POS	NEG	NEG	POS	POS	POS	POS	NEG	NEG	NEG	POS	POS	POS	POS	NEG	NEG	POS	POS	NEG	POS	POS	NEG	NEG	POS	NEG
NEG	NEG	NEG	NEG	POS	POS	POS	POS	NEG	NEG	NEG	NEG	NEG	POS	POS	POS	NEG	POS	POS	NEG	NEG	POS	NEG	NEG	POS	POS	NEG	NEG	POS	POS	POS	POS	NEG	NEG	NEG	POS	POS	POS	POS	NEG	NEG	POS	POS	NEG	POS	POS	NEG	NEG	POS	NEG
NEG	NEG	NEG	NEG	NEG	NEG	NEG	NEG	POS	POS	POS	POS	POS	POS	POS	POS	NEG	POS	POS	POS	NEG	AMB	NEG	NEG	POS	POS	NEG	NEG	POS	POS	POS	NEG	NEG	POS	POS	POS	POS	POS	POS	NEG	NEG	POS	POS	NEG	POS	POS	NEG	POS	NEG	NEG
NEG	NEG	NEG	NEG	NEG	NEG	NEG	NEG	POS	POS	POS	POS	POS	POS	POS	POS	NEG	POS	POS	POS	NEG	NEG	NEG	NEG	POS	POS	NEG	NEG	POS	POS	POS	NEG	NEG	NEG	POS	POS	POS	POS	POS	NEG	NEG	POS	POS	NEG	POS	POS	NEG	POS	NEG	NEG
NEG	NEG	NEG	NEG	NEG	NEG	NEG	NEG	POS	POS	POS	POS	POS	POS	POS	POS	NEG	POS	POS	POS	NEG	NEG	NEG	NEG	POS	POS	NEG	NEG	POS	POS	POS	NEG	NEG	POS	POS	POS	POS	POS	POS	NEG	NEG	POS	POS	NEG	POS	POS	NEG	POS	NEG	NEG
NEG	NEG	NEG	NEG	NEG	NEG	NEG	NEG	POS	POS	POS	POS	POS	POS	POS	POS	NEG	POS	POS	POS	NEG	AMB	NEG	NEG	POS	POS	NEG	NEG	POS	POS	POS	NEG	NEG	POS	POS	POS	POS	POS	POS	NEG	NEG	POS	POS	NEG	POS	POS	NEG	POS	NEG	NEG
NEG	NEG	NEG	NEG	NEG	NEG	NEG	NEG	POS	POS	POS	POS	POS	POS	POS	POS	NEG	POS	POS	POS	NEG	NEG	NEG	NEG	POS	POS	NEG	NEG	POS	POS	POS	NEG	NEG	NEG	POS	POS	POS	NEG	POS	NEG	NEG	POS	POS	NEG	POS	POS	NEG	POS	NEG	NEG
NEG	NEG	NEG	NEG	NEG	NEG	NEG	NEG	POS	POS	POS	POS	POS	POS	POS	POS	NEG	POS	POS	POS	NEG	NEG	NEG	NEG	POS	POS	NEG	NEG	POS	POS	POS	NEG	NEG	POS	POS	POS	POS	POS	POS	NEG	NEG	POS	POS	NEG	POS	POS	NEG	POS	NEG	NEG
NEG	NEG	NEG	NEG	NEG	NEG	NEG	NEG	POS	POS	POS	POS	POS	POS	POS	POS	NEG	POS	POS	POS	NEG	AMB	NEG	NEG	POS	POS	NEG	NEG	POS	POS	POS	NEG	NEG	POS	POS	POS	POS	POS	POS	NEG	NEG	POS	POS	NEG	POS	POS	NEG	POS	NEG	NEG
NEG	NEG	NEG	NEG	NEG	NEG	NEG	NEG	POS	POS	POS	POS	POS	POS	POS	POS	NEG	POS	POS	POS	NEG	NEG	NEG	NEG	POS	POS	NEG	NEG	POS	POS	POS	NEG	NEG	POS	POS	POS	POS	POS	POS	NEG	NEG	POS	POS	NEG	POS	POS	NEG	POS	NEG	NEG
NEG	NEG	NEG	NEG	NEG	NEG	NEG	NEG	POS	POS	POS	POS	POS	POS	POS	POS	NEG	POS	POS	POS	NEG	AMB	NEG	NEG	POS	POS	NEG	NEG	POS	POS	POS	NEG	NEG	POS	POS	POS	POS	POS	POS	NEG	NEG	POS	POS	NEG	POS	POS	NEG	POS	NEG	NEG
NEG	NEG	NEG	NEG	NEG	NEG	NEG	NEG	POS	POS	POS	POS	POS	POS	POS	POS	NEG	POS	POS	POS	NEG	NEG	NEG	NEG	POS	POS	NEG	NEG	POS	POS	POS	NEG	NEG	POS	POS	POS	POS	POS	POS	NEG	NEG	POS	POS	NEG	POS	POS	NEG	POS	NEG	NEG
NEG	NEG	NEG	NEG	NEG	NEG	NEG	NEG	POS	POS	POS	POS	POS	POS	POS	POS	NEG	POS	POS	POS	NEG	NEG	NEG	NEG	POS	NEG	NEG	NEG	POS	POS	POS	NEG	NEG	NEG	POS	POS	POS	NEG	POS	NEG	NEG	POS	POS	NEG	POS	POS	NEG	POS	NEG	NEG
NEG	NEG	NEG	NEG	NEG	NEG	NEG	NEG	POS	POS	POS	POS	POS	POS	POS	POS	NEG	POS	POS	POS	AMB	NEG	NEG	NEG	POS	POS	POS	AMB	AMB	POS	POS	NEG	NEG	POS	NEG	POS	POS	POS	POS	NEG	POS	POS	POS	NEG	POS	POS	NEG	NEG	NEG	NEG
NEG	NEG	NEG	NEG	NEG	NEG	NEG	NEG	POS	POS	POS	POS	POS	POS	POS	POS	NEG	POS	POS	POS	AMB	NEG	NEG	NEG	POS	POS	POS	AMB	AMB	POS	POS	NEG	NEG	POS	NEG	POS	POS	POS	POS	NEG	POS	POS	POS	NEG	POS	POS	NEG	NEG	NEG	NEG
NEG	NEG	NEG	NEG	NEG	NEG	NEG	NEG	POS	POS	POS	POS	POS	POS	POS	POS	NEG	POS	POS	POS	AMB	NEG	NEG	NEG	POS	POS	POS	AMB	AMB	POS	POS	NEG	NEG	POS	NEG	POS	POS	POS	POS	NEG	POS	POS	POS	NEG	POS	POS	NEG	NEG	NEG	NEG
NEG	NEG	NEG	NEG	NEG	NEG	NEG	NEG	POS	POS	POS	POS	POS	POS	POS	POS	NEG	POS	POS	POS	AMB	NEG	NEG	NEG	POS	POS	POS	AMB	AMB	POS	POS	NEG	NEG	POS	NEG	POS	POS	POS	POS	NEG	POS	POS	POS	NEG	POS	POS	NEG	NEG	NEG	NEG
NEG	NEG	NEG	NEG	NEG	NEG	NEG	NEG	POS	POS	POS	POS	POS	POS	POS	POS	NEG	POS	POS	POS	AMB	NEG	NEG	NEG	POS	POS	POS	AMB	AMB	POS	POS	NEG	NEG	POS	NEG	POS	POS	POS	POS	NEG	POS	POS	POS	NEG	POS	POS	NEG	NEG	NEG	NEG
NEG	NEG	NEG	NEG	POS	POS	POS	POS	NEG	NEG	NEG	NEG	NEG	POS	POS	POS	NEG	POS	POS	POS	NEG	AMB	NEG	NEG	POS	POS	POS	AMB	AMB	POS	POS	POS	NEG	NEG	NEG	POS	POS	POS	POS	NEG	POS	POS	POS	NEG	POS	POS	POS	NEG	NEG	NEG
NEG	NEG	NEG	NEG	POS	POS	POS	POS	NEG	NEG	NEG	NEG	NEG	POS	POS	POS	NEG	POS	POS	POS	NEG	NEG	NEG	NEG	POS	POS	POS	NEG	NEG	POS	POS	POS	NEG	NEG	NEG	POS	POS	POS	POS	NEG	POS	POS	POS	NEG	POS	POS	POS	NEG	NEG	NEG
NEG	NEG	NEG	NEG	POS	POS	POS	POS	NEG	NEG	NEG	NEG	NEG	POS	POS	POS	NEG	POS	POS	POS	NEG	NEG	NEG	NEG	POS	POS	POS	AMB	AMB	POS	POS	POS	NEG	NEG	NEG	POS	POS	POS	POS	NEG	POS	POS	POS	NEG	POS	POS	POS	NEG	NEG	NEG
NEG	NEG	NEG	NEG	POS	POS	POS	POS	NEG	NEG	NEG	NEG	NEG	POS	POS	POS	NEG	POS	POS	POS	NEG	NEG	NEG	NEG	POS	POS	POS	AMB	AMB	POS	POS	POS	NEG	NEG	NEG	POS	POS	POS	POS	NEG	POS	POS	POS	NEG	POS	POS	POS	NEG	NEG	NEG
NEG	NEG	NEG	NEG	POS	POS	POS	POS	NEG	NEG	NEG	NEG	NEG	POS	POS	POS	NEG	POS	POS	POS	NEG	NEG	NEG	NEG	POS	POS	POS	AMB	AMB	POS	POS	POS	NEG	NEG	NEG	POS	POS	POS	POS	NEG	POS	POS	POS	NEG	POS	POS	POS	NEG	NEG	NEG
NEG	NEG	NEG	NEG	POS	POS	POS	POS	NEG	NEG	NEG	NEG	NEG	POS	POS	POS	NEG	POS	POS	POS	NEG	NEG	NEG	NEG	POS	POS	POS	AMB	AMB	POS	POS	POS	NEG	NEG	NEG	POS	POS	POS	POS	NEG	POS	POS	POS	NEG	POS	POS	POS	NEG	NEG	NEG
NEG	NEG	NEG	NEG	POS	POS	POS	POS	NEG	NEG	NEG	NEG	NEG	POS	POS	POS	NEG	POS	POS	POS	NEG	NEG	NEG	NEG	POS	POS	POS	AMB	AMB	POS	POS	POS	NEG	NEG	NEG	POS	POS	POS	POS	NEG	POS	POS	POS	NEG	POS	POS	POS	NEG	NEG	NEG
NEG	NEG	NEG	NEG	POS	POS	POS	POS	NEG	NEG	NEG	POS	NEG	POS	POS	POS	NEG	POS	POS	POS	NEG	NEG	NEG	NEG	POS	POS	POS	AMB	AMB	POS	POS	POS	NEG	NEG	NEG	POS	POS	POS	POS	NEG	POS	POS	POS	NEG	POS	POS	POS	NEG	NEG	NEG
NEG	NEG	NEG	NEG	POS	POS	POS	POS	NEG	NEG	NEG	NEG	NEG	POS	POS	POS	NEG	POS	POS	POS	NEG	NEG	NEG	NEG	POS	POS	POS	AMB	AMB	POS	POS	POS	NEG	NEG	NEG	POS	POS	POS	POS	NEG	POS	POS	POS	NEG	POS	POS	POS	NEG	NEG	NEG
NEG	NEG	NEG	NEG	POS	POS	POS	POS	NEG	NEG	NEG	NEG	NEG	POS	POS	POS	NEG	POS	POS	POS	NEG	NEG	NEG	NEG	POS	POS	POS	AMB	AMB	POS	POS	POS	NEG	NEG	NEG	POS	POS	POS	POS	NEG	POS	POS	POS	NEG	POS	POS	POS	NEG	NEG	NEG
NEG	NEG	NEG	NEG	POS	POS	POS	POS	NEG	NEG	NEG	NEG	NEG	POS	POS	POS	NEG	POS	POS	POS	NEG	NEG	NEG	NEG	POS	POS	POS	AMB	AMB	POS	POS	POS	NEG	NEG	NEG	POS	POS	POS	POS	NEG	POS	POS	POS	NEG	POS	POS	POS	NEG	NEG	NEG
NEG	NEG	NEG	NEG	POS	POS	POS	POS	NEG	NEG	NEG	NEG	NEG	POS	POS	POS	NEG	POS	POS	POS	NEG	NEG	NEG	NEG	POS	POS	POS	AMB	AMB	POS	POS	POS	NEG	NEG	NEG	POS	POS	POS	POS	NEG	POS	POS	POS	NEG	POS	POS	POS	NEG	NEG	NEG
NEG	NEG	NEG	NEG	POS	POS	POS	POS	NEG	NEG	NEG	NEG	NEG	POS	POS	POS	NEG	POS	POS	POS	NEG	NEG	NEG	NEG	POS	POS	POS	AMB	AMB	POS	POS	POS	NEG	NEG	NEG	POS	POS	POS	POS	NEG	POS	POS	POS	NEG	POS	POS	POS	NEG	NEG	NEG
NEG	NEG	NEG	NEG	POS	POS	POS	POS	NEG	NEG	NEG	NEG	NEG	POS	POS	POS	NEG	POS	POS	POS	NEG	NEG	NEG	NEG	POS	POS	POS	AMB	AMB	POS	POS	POS	NEG	NEG	NEG	POS	POS	POS	POS	NEG	POS	POS	POS	NEG	POS	POS	POS	NEG	NEG	NEG
NEG	NEG	NEG	NEG	POS	POS	POS	POS	NEG	NEG	NEG	NEG	NEG	POS	POS	POS	NEG	POS	POS	POS	NEG	NEG	NEG	NEG	POS	POS	POS	NEG	AMB	POS	POS	POS	NEG	NEG	NEG	POS	POS	POS	POS	NEG	POS	POS	POS	NEG	POS	POS	POS	NEG	NEG	NEG
NEG	NEG	NEG	NEG	POS	POS	POS	POS	NEG	NEG	NEG	NEG	NEG	POS	POS	POS	NEG	POS	POS	POS	NEG	NEG	NEG	NEG	POS	POS	POS	AMB	AMB	POS	POS	POS	NEG	NEG	NEG	POS	POS	POS	POS	NEG	POS	POS	POS	NEG	POS	POS	POS	NEG	NEG	NEG
NEG	NEG	NEG	NEG	POS	POS	POS	POS	NEG	NEG	NEG	NEG	NEG	POS	POS	POS	NEG	POS	POS	NEG	NEG	POS	NEG	NEG	POS	POS	NEG	NEG	POS	POS	POS	NEG	NEG	POS	NEG	POS	POS	POS	POS	NEG	NEG	POS	POS	NEG	POS	POS	NEG	POS	NEG	NEG
NEG	NEG	NEG	NEG	NEG	NEG	NEG	NEG	POS	POS	POS	POS	POS	POS	POS	POS	NEG	NEG	NEG	NEG	NEG	NEG	NEG	NEG	POS	POS	POS	AMB	AMB	POS	POS	NEG	NEG	POS	POS	POS	POS	POS	POS	NEG	NEG	POS	POS	NEG	POS	POS	NEG	NEG	NEG	NEG
NEG	NEG	NEG	NEG	NEG	NEG	NEG	NEG	POS	POS	POS	POS	POS	POS	POS	POS	NEG	POS	POS	NEG	NEG	POS	NEG	NEG	POS	POS	NEG	NEG	POS	POS	POS	NEG	POS	AMB	NEG	POS	POS	POS	POS	NEG	POS	POS	POS	NEG	POS	POS	POS	NEG	NEG	NEG
NEG	NEG	NEG	NEG	NEG	NEG	NEG	NEG	POS	POS	POS	POS	POS	POS	POS	POS	NEG	POS	POS	NEG	NEG	POS	NEG	NEG	POS	POS	NEG	NEG	POS	POS	POS	NEG	POS	AMB	NEG	POS	POS	POS	POS	NEG	POS	POS	POS	NEG	POS	POS	POS	NEG	NEG	NEG
NEG	NEG	NEG	NEG	NEG	NEG	NEG	NEG	POS	POS	POS	POS	POS	POS	POS	POS	NEG	POS	POS	NEG	NEG	POS	NEG	NEG	POS	POS	NEG	NEG	POS	POS	POS	NEG	POS	AMB	NEG	POS	POS	POS	POS	NEG	POS	POS	POS	NEG	POS	POS	POS	NEG	NEG	NEG
NEG	NEG	NEG	NEG	POS	POS	POS	POS	NEG	NEG	NEG	NEG	NEG	POS	POS	POS	NEG	NEG	NEG	NEG	NEG	NEG	NEG	NEG	POS	POS	NEG	NEG	POS	POS	POS	NEG	NEG	POS	POS	NEG	POS	POS	POS	NEG	POS	POS	NEG	POS	POS	POS	NEG	NEG	POS	NEG
NEG	NEG	NEG	NEG	NEG	NEG	NEG	NEG	POS	POS	POS	POS	POS	POS	POS	POS	NEG	POS	POS	NEG	POS	NEG	NEG	NEG	POS	POS	NEG	POS	NEG	POS	POS	NEG	NEG	NEG	POS	POS	POS	POS	POS	NEG	NEG	POS	NEG	POS	POS	POS	NEG	POS	NEG	NEG
NEG	NEG	NEG	NEG	NEG	NEG	NEG	NEG	POS	POS	POS	POS	POS	POS	POS	POS	NEG	POS	POS	NEG	NEG	NEG	NEG	POS	POS	POS	AMB	NEG	POS	POS	POS	NEG	POS	AMB	POS	POS	POS	NEG	POS	POS	NEG	POS	NEG	POS	POS	POS	NEG	NEG	POS	NEG
NEG	NEG	NEG	NEG	NEG	NEG	NEG	NEG	POS	POS	POS	POS	POS	POS	POS	POS	NEG	POS	POS	NEG	NEG	NEG	NEG	POS	POS	POS	AMB	NEG	POS	POS	POS	NEG	POS	AMB	POS	POS	POS	NEG	POS	POS	NEG	POS	NEG	POS	POS	POS	NEG	NEG	POS	NEG
NEG	NEG	NEG	NEG	NEG	NEG	NEG	NEG	POS	POS	POS	POS	POS	POS	POS	POS	NEG	POS	POS	NEG	NEG	NEG	NEG	POS	POS	POS	NEG	NEG	POS	POS	POS	NEG	POS	AMB	POS	POS	POS	NEG	POS	POS	NEG	POS	NEG	POS	POS	POS	NEG	NEG	POS	NEG
NEG	NEG	NEG	NEG	NEG	NEG	NEG	NEG	POS	POS	POS	POS	POS	POS	POS	POS	NEG	NEG	NEG	NEG	NEG	NEG	NEG	NEG	POS	POS	AMB	NEG	POS	POS	POS	NEG	POS	AMB	POS	POS	POS	NEG	POS	POS	NEG	POS	NEG	POS	POS	POS	NEG	NEG	POS	NEG
NEG	NEG	NEG	NEG	NEG	NEG	NEG	NEG	POS	POS	POS	POS	POS	POS	POS	POS	NEG	POS	POS	NEG	NEG	NEG	NEG	NEG	POS	POS	POS	NEG	AMB	POS	POS	NEG	NEG	POS	NEG	POS	POS	POS	POS	NEG	POS	POS	POS	NEG	POS	POS	NEG	POS	NEG	NEG
NEG	NEG	NEG	NEG	POS	POS	POS	POS	NEG	NEG	NEG	NEG	NEG	POS	POS	POS	NEG	POS	POS	NEG	NEG	POS	NEG	NEG	POS	POS	POS	AMB	AMB	POS	POS	NEG	NEG	NEG	NEG	POS	POS	NEG	POS	NEG	NEG	POS	POS	NEG	POS	POS	POS	NEG	NEG	NEG
NEG	NEG	NEG	NEG	POS	POS	POS	POS	NEG	NEG	NEG	NEG	NEG	POS	POS	POS	NEG	POS	POS	NEG	NEG	POS	NEG	NEG	POS	POS	POS	AMB	AMB	POS	POS	NEG	NEG	POS	NEG	POS	POS	POS	POS	NEG	NEG	POS	POS	NEG	POS	POS	POS	NEG	NEG	NEG
NEG	NEG	NEG	NEG	POS	POS	POS	POS	NEG	NEG	NEG	NEG	NEG	POS	POS	POS	NEG	POS	POS	NEG	NEG	POS	NEG	NEG	POS	POS	POS	NEG	AMB	POS	POS	NEG	NEG	NEG	NEG	POS	POS	POS	POS	NEG	NEG	POS	POS	NEG	POS	POS	POS	NEG	NEG	NEG
NEG	NEG	NEG	NEG	POS	POS	POS	POS	NEG	NEG	NEG	NEG	NEG	POS	POS	POS	NEG	POS	POS	NEG	NEG	POS	NEG	NEG	POS	POS	POS	AMB	AMB	POS	POS	NEG	NEG	POS	NEG	POS	POS	POS	POS	NEG	NEG	POS	POS	NEG	POS	POS	POS	NEG	NEG	NEG
NEG	NEG	NEG	NEG	POS	POS	POS	POS	NEG	NEG	NEG	NEG	NEG	POS	POS	POS	NEG	POS	POS	NEG	NEG	POS	NEG	NEG	POS	POS	POS	AMB	AMB	POS	POS	NEG	NEG	POS	NEG	POS	POS	POS	POS	NEG	NEG	POS	POS	NEG	POS	POS	POS	NEG	NEG	NEG
NEG	NEG	NEG	NEG	NEG	NEG	NEG	NEG	POS	POS	POS	POS	POS	POS	POS	POS	NEG	POS	POS	NEG	NEG	POS	NEG	NEG	POS	POS	NEG	NEG	POS	POS	POS	NEG	NEG	POS	NEG	POS	POS	POS	POS	NEG	POS	POS	POS	NEG	POS	POS	NEG	NEG	NEG	NEG
NEG	NEG	NEG	NEG	POS	POS	POS	POS	NEG	NEG	NEG	NEG	NEG	POS	POS	POS	NEG	POS	POS	NEG	NEG	POS	NEG	NEG	POS	POS	POS	AMB	AMB	POS	POS	NEG	NEG	POS	NEG	POS	POS	POS	POS	NEG	NEG	POS	POS	NEG	POS	POS	NEG	NEG	NEG	NEG
NEG	NEG	NEG	NEG	POS	POS	POS	POS	NEG	NEG	NEG	NEG	NEG	POS	POS	POS	NEG	POS	POS	NEG	NEG	POS	NEG	NEG	POS	POS	POS	AMB	AMB	POS	POS	NEG	NEG	POS	NEG	POS	POS	POS	POS	NEG	NEG	POS	POS	NEG	POS	POS	NEG	NEG	NEG	NEG
NEG	NEG	NEG	NEG	POS	POS	POS	POS	NEG	NEG	NEG	NEG	NEG	POS	POS	POS	NEG	POS	POS	NEG	NEG	POS	NEG	NEG	POS	POS	POS	AMB	AMB	POS	POS	NEG	NEG	POS	NEG	POS	POS	POS	POS	NEG	NEG	POS	POS	NEG	POS	POS	NEG	NEG	NEG	NEG
NEG	NEG	NEG	NEG	POS	POS	POS	POS	NEG	NEG	NEG	NEG	NEG	POS	POS	POS	NEG	NEG	NEG	NEG	NEG	NEG	NEG	NEG	POS	POS	POS	AMB	AMB	POS	POS	NEG	NEG	POS	NEG	POS	POS	POS	POS	NEG	NEG	POS	POS	NEG	POS	POS	NEG	NEG	NEG	NEG
NEG	NEG	NEG	NEG	POS	POS	POS	POS	NEG	NEG	NEG	NEG	NEG	POS	POS	POS	NEG	POS	POS	NEG	NEG	NEG	NEG	NEG	POS	POS	POS	AMB	AMB	POS	POS	NEG	NEG	POS	NEG	POS	POS	POS	POS	NEG	NEG	POS	POS	NEG	POS	POS	NEG	NEG	NEG	NEG
NEG	NEG	NEG	NEG	POS	POS	POS	POS	NEG	NEG	NEG	NEG	NEG	POS	POS	POS	NEG	POS	POS	NEG	NEG	POS	NEG	NEG	POS	POS	POS	AMB	AMB	POS	POS	NEG	NEG	POS	NEG	POS	POS	POS	POS	NEG	NEG	POS	POS	NEG	POS	POS	NEG	NEG	NEG	NEG
NEG	NEG	NEG	NEG	POS	POS	POS	POS	NEG	NEG	NEG	NEG	NEG	POS	POS	POS	NEG	POS	POS	NEG	NEG	POS	NEG	NEG	POS	POS	POS	AMB	AMB	POS	POS	NEG	NEG	POS	NEG	POS	POS	POS	POS	NEG	NEG	POS	POS	NEG	POS	POS	NEG	NEG	NEG	NEG
NEG	NEG	NEG	NEG	POS	POS	POS	POS	NEG	NEG	NEG	NEG	NEG	POS	POS	POS	NEG	POS	POS	NEG	NEG	POS	NEG	NEG	POS	POS	POS	NEG	AMB	POS	POS	NEG	NEG	NEG	NEG	POS	POS	POS	POS	NEG	NEG	POS	POS	NEG	POS	POS	NEG	NEG	NEG	NEG
NEG	NEG	NEG	NEG	POS	POS	POS	POS	NEG	NEG	NEG	NEG	NEG	POS	POS	POS	NEG	POS	POS	NEG	NEG	POS	NEG	NEG	POS	POS	POS	NEG	AMB	POS	POS	NEG	NEG	NEG	NEG	POS	POS	POS	POS	NEG	NEG	POS	POS	NEG	POS	POS	NEG	NEG	NEG	NEG
NEG	NEG	NEG	NEG	POS	POS	POS	POS	NEG	NEG	NEG	NEG	NEG	POS	POS	POS	NEG	POS	POS	NEG	NEG	POS	NEG	NEG	POS	POS	POS	AMB	AMB	POS	POS	NEG	NEG	NEG	NEG	POS	POS	POS	POS	NEG	NEG	POS	POS	NEG	POS	POS	NEG	NEG	NEG	NEG
NEG	NEG	NEG	NEG	NEG	NEG	NEG	NEG	POS	POS	POS	POS	POS	POS	POS	POS	NEG	POS	POS	POS	AMB	NEG	NEG	NEG	POS	POS	POS	AMB	AMB	POS	POS	NEG	NEG	POS	NEG	POS	POS	POS	POS	NEG	NEG	POS	POS	NEG	POS	POS	NEG	POS	NEG	NEG
NEG	NEG	NEG	NEG	NEG	NEG	NEG	NEG	POS	POS	POS	POS	POS	POS	POS	POS	NEG	POS	POS	POS	AMB	NEG	NEG	NEG	POS	POS	POS	AMB	AMB	POS	POS	NEG	NEG	POS	NEG	POS	POS	POS	POS	NEG	NEG	POS	POS	NEG	POS	POS	NEG	POS	NEG	NEG
NEG	NEG	NEG	NEG	NEG	NEG	NEG	NEG	POS	POS	POS	POS	POS	POS	POS	POS	NEG	NEG	NEG	NEG	NEG	NEG	NEG	NEG	POS	POS	NEG	NEG	POS	POS	POS	NEG	NEG	NEG	POS	POS	POS	NEG	POS	NEG	NEG	POS	POS	NEG	POS	POS	NEG	NEG	NEG	NEG
NEG	NEG	NEG	NEG	POS	POS	POS	POS	NEG	NEG	NEG	NEG	NEG	POS	NEG	POS	NEG	POS	POS	POS	NEG	NEG	NEG	NEG	POS	POS	POS	AMB	AMB	POS	POS	NEG	POS	AMB	POS	POS	POS	POS	POS	NEG	NEG	POS	NEG	NEG	POS	POS	NEG	NEG	NEG	NEG
NEG	NEG	NEG	NEG	POS	POS	POS	POS	NEG	NEG	NEG	NEG	NEG	POS	NEG	POS	NEG	POS	POS	POS	NEG	NEG	NEG	NEG	POS	POS	POS	AMB	AMB	POS	POS	NEG	POS	AMB	POS	POS	POS	POS	POS	NEG	NEG	POS	NEG	NEG	POS	POS	NEG	NEG	NEG	NEG
NEG	NEG	NEG	NEG	POS	POS	POS	POS	NEG	NEG	NEG	NEG	NEG	POS	NEG	POS	NEG	POS	POS	POS	NEG	NEG	NEG	NEG	POS	POS	POS	AMB	AMB	POS	POS	NEG	POS	AMB	POS	POS	POS	POS	POS	NEG	NEG	POS	NEG	NEG	POS	POS	NEG	NEG	NEG	NEG
NEG	NEG	NEG	NEG	POS	POS	POS	POS	NEG	NEG	NEG	NEG	NEG	POS	NEG	POS	NEG	POS	POS	POS	NEG	NEG	NEG	NEG	POS	POS	POS	AMB	AMB	POS	POS	NEG	POS	AMB	POS	POS	POS	POS	POS	NEG	NEG	POS	NEG	NEG	POS	POS	NEG	NEG	NEG	NEG
NEG	NEG	NEG	NEG	POS	POS	POS	POS	NEG	NEG	NEG	NEG	NEG	POS	NEG	POS	NEG	POS	POS	POS	NEG	NEG	NEG	NEG	POS	POS	POS	AMB	AMB	POS	POS	NEG	AMB	POS	POS	POS	POS	POS	POS	NEG	NEG	POS	NEG	NEG	POS	POS	NEG	NEG	NEG	NEG
NEG	NEG	NEG	NEG	POS	POS	POS	POS	NEG	NEG	NEG	NEG	NEG	POS	NEG	POS	NEG	POS	POS	POS	NEG	NEG	NEG	NEG	POS	POS	POS	AMB	AMB	POS	POS	NEG	POS	AMB	POS	POS	POS	POS	POS	NEG	NEG	POS	NEG	NEG	POS	POS	NEG	NEG	NEG	NEG
NEG	NEG	NEG	NEG	POS	POS	POS	POS	NEG	NEG	NEG	NEG	NEG	POS	NEG	POS	NEG	POS	POS	POS	NEG	NEG	NEG	NEG	POS	POS	POS	AMB	AMB	POS	POS	NEG	AMB	POS	POS	POS	POS	POS	POS	NEG	NEG	POS	NEG	NEG	POS	POS	NEG	NEG	NEG	NEG
NEG	NEG	NEG	NEG	POS	POS	POS	POS	NEG	NEG	NEG	NEG	NEG	POS	NEG	POS	NEG	POS	POS	POS	NEG	NEG	NEG	NEG	POS	POS	POS	AMB	AMB	POS	POS	NEG	AMB	POS	POS	POS	POS	POS	POS	NEG	NEG	POS	NEG	NEG	POS	POS	NEG	NEG	NEG	NEG
NEG	NEG	NEG	NEG	POS	POS	POS	POS	NEG	NEG	NEG	NEG	NEG	POS	NEG	POS	NEG	POS	POS	POS	NEG	NEG	NEG	NEG	POS	POS	POS	AMB	AMB	POS	POS	NEG	POS	AMB	POS	POS	POS	POS	POS	NEG	NEG	POS	NEG	NEG	POS	POS	NEG	NEG	NEG	NEG
NEG	NEG	NEG	NEG	POS	POS	POS	POS	NEG	NEG	NEG	NEG	NEG	POS	NEG	POS	NEG	POS	POS	POS	NEG	NEG	NEG	NEG	POS	POS	POS	AMB	AMB	POS	POS	NEG	POS	AMB	POS	POS	POS	POS	POS	NEG	NEG	POS	NEG	NEG	POS	POS	NEG	NEG	NEG	NEG
NEG	NEG	NEG	NEG	POS	POS	POS	POS	NEG	NEG	NEG	NEG	NEG	POS	NEG	POS	NEG	POS	POS	POS	NEG	NEG	NEG	NEG	POS	POS	POS	AMB	AMB	POS	POS	NEG	POS	AMB	POS	POS	POS	POS	POS	NEG	NEG	POS	NEG	NEG	POS	POS	NEG	NEG	NEG	NEG
NEG	NEG	NEG	NEG	NEG	POS	AMB	NEG	NEG	NEG	NEG	NEG	NEG	POS	NEG	POS	NEG	POS	POS	NEG	NEG	NEG	NEG	NEG	POS	POS	POS	NEG	AMB	POS	POS	NEG	POS	AMB	NEG	POS	POS	POS	POS	NEG	NEG	POS	NEG	NEG	POS	POS	NEG	NEG	NEG	NEG
NEG	NEG	NEG	NEG	POS	POS	POS	POS	NEG	NEG	NEG	NEG	NEG	POS	NEG	POS	NEG	POS	POS	POS	NEG	NEG	NEG	NEG	POS	POS	POS	AMB	AMB	POS	POS	NEG	POS	AMB	POS	POS	POS	POS	POS	NEG	NEG	POS	NEG	NEG	POS	POS	NEG	NEG	NEG	NEG
NEG	NEG	NEG	NEG	POS	POS	POS	POS	NEG	NEG	NEG	NEG	NEG	POS	NEG	POS	NEG	POS	POS	POS	NEG	NEG	NEG	NEG	POS	POS	POS	AMB	AMB	POS	POS	NEG	AMB	POS	POS	POS	POS	POS	POS	NEG	NEG	POS	NEG	NEG	POS	POS	NEG	NEG	NEG	NEG
NEG	NEG	NEG	NEG	POS	POS	POS	POS	NEG	NEG	NEG	NEG	NEG	POS	NEG	POS	NEG	POS	POS	POS	NEG	NEG	NEG	NEG	POS	POS	POS	AMB	AMB	POS	POS	NEG	AMB	POS	POS	POS	POS	POS	POS	NEG	NEG	POS	NEG	NEG	POS	POS	NEG	NEG	NEG	NEG
NEG	NEG	NEG	NEG	POS	POS	POS	POS	NEG	NEG	NEG	NEG	NEG	POS	NEG	POS	NEG	POS	POS	POS	NEG	NEG	NEG	NEG	POS	POS	POS	AMB	AMB	POS	POS	NEG	AMB	POS	POS	POS	POS	POS	POS	NEG	NEG	POS	NEG	NEG	POS	POS	NEG	NEG	NEG	NEG
NEG	NEG	NEG	NEG	NEG	NEG	NEG	NEG	POS	POS	POS	POS	POS	POS	POS	POS	NEG	POS	POS	POS	NEG	AMB	NEG	NEG	POS	POS	NEG	NEG	POS	POS	POS	NEG	NEG	POS	POS	POS	POS	POS	POS	NEG	POS	POS	POS	NEG	POS	POS	NEG	NEG	NEG	NEG
NEG	NEG	NEG	NEG	NEG	NEG	NEG	NEG	POS	POS	POS	POS	POS	POS	POS	POS	NEG	POS	POS	POS	NEG	NEG	NEG	NEG	POS	POS	POS	AMB	AMB	POS	POS	NEG	NEG	POS	POS	POS	POS	POS	POS	NEG	POS	POS	POS	NEG	POS	POS	POS	NEG	NEG	NEG
NEG	NEG	NEG	NEG	NEG	NEG	NEG	NEG	POS	POS	POS	POS	POS	POS	POS	POS	NEG	POS	POS	POS	NEG	NEG	NEG	NEG	POS	POS	POS	AMB	AMB	POS	POS	NEG	NEG	POS	POS	POS	POS	POS	POS	NEG	POS	POS	POS	NEG	POS	POS	POS	NEG	NEG	NEG
NEG	NEG	NEG	NEG	NEG	NEG	NEG	NEG	POS	POS	POS	POS	POS	POS	POS	POS	NEG	POS	POS	POS	NEG	NEG	NEG	NEG	POS	POS	POS	AMB	AMB	POS	POS	NEG	NEG	NEG	POS	POS	POS	POS	POS	NEG	POS	POS	POS	NEG	POS	POS	POS	NEG	NEG	NEG
NEG	NEG	NEG	NEG	NEG	NEG	NEG	NEG	POS	POS	POS	POS	POS	POS	POS	POS	NEG	POS	POS	POS	NEG	NEG	NEG	NEG	POS	POS	POS	AMB	AMB	POS	POS	NEG	NEG	POS	POS	POS	POS	POS	POS	NEG	POS	POS	POS	NEG	POS	POS	POS	NEG	NEG	NEG
NEG	NEG	NEG	NEG	NEG	NEG	NEG	NEG	POS	POS	POS	POS	POS	POS	POS	POS	NEG	POS	POS	POS	NEG	NEG	NEG	NEG	POS	POS	POS	AMB	AMB	POS	POS	NEG	NEG	POS	POS	POS	POS	POS	POS	NEG	POS	POS	POS	NEG	POS	POS	POS	NEG	NEG	NEG
NEG	NEG	NEG	NEG	NEG	NEG	NEG	NEG	POS	POS	POS	POS	POS	POS	POS	POS	NEG	POS	POS	POS	NEG	NEG	NEG	NEG	POS	POS	POS	AMB	AMB	POS	POS	NEG	NEG	POS	POS	POS	POS	POS	POS	NEG	POS	POS	POS	NEG	POS	POS	POS	NEG	NEG	NEG
NEG	NEG	NEG	NEG	NEG	NEG	NEG	NEG	POS	POS	POS	POS	POS	POS	POS	POS	NEG	POS	POS	POS	NEG	NEG	NEG	NEG	POS	POS	POS	AMB	AMB	POS	POS	NEG	NEG	POS	POS	POS	POS	POS	POS	NEG	POS	POS	POS	NEG	POS	POS	POS	NEG	NEG	NEG
NEG	NEG	NEG	NEG	POS	POS	POS	POS	NEG	NEG	NEG	NEG	NEG	POS	POS	POS	NEG	POS	POS	NEG	NEG	NEG	NEG	POS	POS	POS	POS	AMB	AMB	POS	POS	NEG	POS	AMB	POS	POS	POS	NEG	POS	POS	POS	POS	NEG	POS	POS	POS	NEG	NEG	NEG	NEG
NEG	NEG	NEG	NEG	POS	POS	POS	POS	NEG	NEG	NEG	NEG	NEG	POS	POS	POS	NEG	POS	POS	NEG	NEG	AMB	NEG	POS	POS	POS	POS	AMB	AMB	POS	POS	NEG	POS	AMB	POS	POS	POS	NEG	POS	POS	POS	POS	NEG	POS	POS	POS	NEG	NEG	NEG	NEG
NEG	NEG	NEG	NEG	POS	POS	POS	POS	NEG	NEG	NEG	NEG	NEG	POS	POS	POS	NEG	POS	POS	NEG	NEG	AMB	NEG	POS	POS	POS	POS	AMB	AMB	POS	POS	NEG	POS	AMB	POS	POS	POS	NEG	POS	POS	POS	POS	NEG	POS	POS	POS	NEG	NEG	NEG	NEG
NEG	NEG	NEG	NEG	POS	POS	POS	POS	NEG	NEG	NEG	NEG	NEG	POS	POS	POS	NEG	POS	POS	NEG	NEG	NEG	NEG	POS	POS	POS	POS	AMB	AMB	POS	POS	NEG	AMB	POS	POS	POS	POS	NEG	POS	POS	POS	POS	NEG	POS	POS	POS	NEG	NEG	NEG	NEG
NEG	NEG	NEG	NEG	POS	POS	POS	POS	NEG	NEG	NEG	NEG	NEG	POS	POS	POS	NEG	POS	POS	AMB	NEG	AMB	NEG	POS	POS	POS	POS	AMB	AMB	POS	POS	NEG	AMB	POS	POS	POS	POS	NEG	POS	POS	POS	POS	NEG	POS	POS	POS	NEG	NEG	NEG	NEG
NEG	NEG	NEG	NEG	POS	POS	POS	POS	NEG	NEG	NEG	NEG	NEG	POS	NEG	NEG	NEG	POS	POS	NEG	NEG	NEG	NEG	POS	POS	NEG	NEG	POS	NEG	POS	AMB	POS	NEG	NEG	POS	NEG	NEG	NEG	NEG	NEG	NEG	POS	NEG	NEG	POS	POS	NEG	POS	NEG	NEG
NEG	NEG	NEG	NEG	POS	NEG	POS	POS	NEG	NEG	NEG	NEG	NEG	AMB	NEG	NEG	NEG	POS	POS	NEG	NEG	NEG	NEG	NEG	POS	NEG	NEG	POS	NEG	POS	NEG	POS	NEG	NEG	POS	NEG	NEG	NEG	NEG	NEG	NEG	POS	NEG	NEG	POS	POS	NEG	POS	NEG	NEG

ADHAESION FACTORS / GENES ENCODING MICROBIAL SURFACE COMPONENTS RECOGNIZING ADHESIVE MATRIX MOLECULES (MSCRAMM GENES)																																		IMMUNOD.AG.B		DEFENSIN RESIST.		TRANSFERRIN BINDING PROT			PUTATIVE TRANSPORTER			
fnbB							map				sasG				sasX= sesI	sdrC							sdrD					vwb						isaB		mprF		isdA			lmrP			
fnbB	fnbB (COL)	fnbB (COL+Mu50+MW2)	fnbB (Mu50)	fnbB (MW2)	fnbB (ST15)	fnbB (ST45-2)	map	map (COL)	map (MRSA252)	map (Mu50+MW2)	sasG	sasG (COL+Mu50)	sasG (MW2)	sasG (OtherThan 252+122)		sdrC	sdrC (cons)	sdrC (B1)	sdrC (COL)	sdrC (Mu50)	sdrC (MW2+ MRSA252+RF122)	sdrC (OtherThan 252+RF122)	sdrD	sdrD (cons)	sdrD (COL+MW2)	sdrD (Mu50)	sdrD (other)	vwb	vwb (cons)	vwb (COL+MW2)	vwb (MRSA252)	vwb (Mu50)	vwb (RF122)	isaB	isaB (MRSA252)	mprF (COL+MW2)	mprF (Mu50+252)	isdA (cons)	isdA (MRSA252)	isdA (Other Than MRSA252)	lmrP (OtherThanRF122)	lmrP (OtherThanRF122)	lmrP (RF122)	lmrP (RF122)
Fibronectin-binding protein B							Major histocompatibility complex class II analog protein (=Extracellular adherence protein, eap)				Staphylococcus aureus surface protein G					Ser-Asp rich fibrinogen-/bone sialoprotein-binding protein C							Ser-Asp rich fibrinogen-/bone sialoprotein-binding protein D					van Willebrand factor binding protein						immunodominant antigen B		defensin resistance protein		transferrin-binding protein			hypothetical protein, similar to integral membrane protein LmrP			
POS	NEG	AMB	NEG	POS	NEG	NEG	POS	NEG	NEG	POS	POS	NEG	POS	POS	n/a	POS	POS	NEG	NEG	NEG	POS	POS	POS	POS	POS	NEG	NEG	POS	POS	POS	NEG	NEG	NEG	POS	AMB	POS	AMB	POS	NEG	POS	POS	POS	NEG	NEG
POS	NEG	POS	NEG	AMB	NEG	NEG	POS	NEG	NEG	POS	POS	NEG	POS	POS	n/a	POS	POS	NEG	NEG	NEG	POS	POS	POS	POS	POS	NEG	NEG	POS	POS	POS	NEG	NEG	NEG	POS	AMB	POS	AMB	POS	NEG	POS	POS	POS	NEG	NEG
POS	NEG	AMB	NEG	POS	NEG	NEG	POS	NEG	NEG	POS	POS	NEG	POS	POS	n/a	POS	POS	NEG	NEG	NEG	POS	POS	POS	POS	POS	NEG	NEG	POS	POS	POS	NEG	NEG	NEG	POS	AMB	POS	NEG	POS	NEG	POS	POS	POS	NEG	NEG
POS	NEG	POS	NEG	AMB	NEG	NEG	POS	NEG	NEG	POS	POS	NEG	POS	POS	n/a	POS	POS	NEG	NEG	NEG	POS	POS	POS	POS	POS	NEG	NEG	POS	POS	POS	NEG	NEG	NEG	POS	AMB	POS	NEG	POS	NEG	POS	POS	POS	NEG	NEG
POS	NEG	POS	NEG	AMB	NEG	NEG	NEG	NEG	NEG	NEG	POS	NEG	POS	POS	n/a	POS	POS	NEG	NEG	NEG	POS	POS	NEG	NEG	NEG	NEG	NEG	POS	POS	POS	NEG	NEG	NEG	POS	NEG	POS	NEG	POS	NEG	POS	POS	POS	NEG	NEG
POS	NEG	POS	AMB	NEG	NEG	NEG	POS	NEG	NEG	POS	POS	POS	NEG	POS	n/a	POS	POS	NEG	NEG	POS	NEG	POS	POS	POS	NEG	POS	NEG	POS	POS	NEG	NEG	POS	NEG	POS	NEG	NEG	POS	POS	NEG	POS	POS	POS	NEG	NEG
POS	NEG	POS	AMB	NEG	NEG	NEG	POS	AMB	NEG	POS	POS	POS	NEG	POS	n/a	POS	POS	NEG	NEG	POS	NEG	POS	POS	POS	NEG	POS	NEG	POS	POS	NEG	NEG	POS	NEG	POS	AMB	AMB	POS	POS	NEG	POS	POS	POS	NEG	NEG
POS	NEG	POS	AMB	NEG	NEG	NEG	POS	NEG	NEG	POS	POS	POS	NEG	POS	n/a	POS	POS	NEG	NEG	POS	NEG	POS	POS	POS	NEG	POS	NEG	POS	POS	NEG	NEG	POS	NEG	POS	AMB	NEG	POS	POS	NEG	POS	POS	POS	NEG	NEG
POS	NEG	POS	AMB	NEG	NEG	NEG	POS	NEG	NEG	POS	POS	NEG	POS	POS	n/a	POS	POS	NEG	POS	NEG	NEG	POS	POS	POS	NEG	POS	NEG	POS	POS	NEG	POS	NEG	NEG	POS	AMB	POS	AMB	POS	NEG	POS	POS	POS	NEG	NEG
POS	NEG	POS	AMB	NEG	NEG	NEG	POS	NEG	NEG	POS	POS	NEG	POS	POS	n/a	POS	POS	NEG	POS	NEG	NEG	POS	POS	POS	NEG	POS	NEG	POS	POS	NEG	POS	NEG	NEG	POS	AMB	POS	AMB	POS	NEG	POS	POS	POS	NEG	NEG
POS	NEG	POS	AMB	NEG	NEG	NEG	POS	NEG	NEG	POS	POS	NEG	POS	POS	n/a	POS	POS	NEG	POS	NEG	NEG	POS	POS	POS	NEG	POS	NEG	POS	POS	NEG	POS	NEG	NEG	POS	AMB	POS	AMB	POS	NEG	POS	POS	POS	NEG	NEG
POS	NEG	POS	AMB	NEG	NEG	NEG	POS	NEG	NEG	POS	POS	NEG	POS	POS	n/a	POS	POS	NEG	POS	NEG	NEG	POS	POS	POS	NEG	POS	NEG	POS	POS	NEG	POS	NEG	NEG	POS	AMB	POS	AMB	POS	NEG	POS	POS	POS	NEG	NEG
POS	NEG	AMB	POS	NEG	NEG	NEG	POS	NEG	NEG	POS	POS	NEG	POS	POS	n/a	POS	POS	NEG	POS	NEG	NEG	POS	POS	POS	NEG	POS	NEG	POS	POS	NEG	POS	NEG	NEG	POS	AMB	POS	NEG	POS	NEG	POS	POS	POS	NEG	NEG
POS	NEG	POS	AMB	NEG	NEG	NEG	POS	NEG	NEG	POS	POS	NEG	POS	POS	n/a	POS	POS	NEG	POS	NEG	NEG	POS	POS	POS	NEG	POS	NEG	POS	POS	NEG	POS	NEG	NEG	POS	AMB	POS	AMB	POS	NEG	POS	POS	POS	NEG	NEG
POS	NEG	POS	AMB	NEG	NEG	NEG	POS	NEG	NEG	POS	POS	NEG	POS	POS	n/a	POS	POS	NEG	POS	NEG	NEG	POS	POS	POS	NEG	POS	NEG	POS	POS	NEG	POS	NEG	NEG	POS	AMB	POS	AMB	POS	NEG	POS	POS	POS	NEG	NEG
POS	NEG	POS	AMB	NEG	NEG	NEG	POS	NEG	NEG	POS	POS	NEG	POS	POS	n/a	POS	POS	NEG	POS	NEG	NEG	POS	POS	POS	NEG	POS	NEG	POS	POS	NEG	POS	NEG	NEG	POS	AMB	POS	AMB	POS	NEG	POS	POS	POS	NEG	NEG
POS	NEG	POS	AMB	NEG	NEG	NEG	POS	NEG	NEG	POS	POS	NEG	POS	POS	n/a	POS	POS	NEG	POS	NEG	NEG	POS	POS	POS	NEG	POS	NEG	POS	POS	NEG	POS	NEG	NEG	POS	AMB	POS	AMB	POS	NEG	POS	POS	POS	NEG	NEG
POS	NEG	POS	AMB	NEG	NEG	NEG	POS	NEG	NEG	POS	POS	NEG	POS	POS	n/a	POS	POS	NEG	POS	NEG	NEG	POS	POS	POS	NEG	POS	NEG	POS	POS	NEG	POS	NEG	NEG	POS	AMB	POS	AMB	POS	NEG	POS	POS	POS	NEG	NEG
POS	NEG	POS	AMB	NEG	NEG	NEG	POS	NEG	NEG	POS	POS	NEG	POS	POS	n/a	POS	POS	NEG	POS	NEG	NEG	POS	POS	POS	NEG	POS	NEG	POS	POS	NEG	AMB	NEG	NEG	POS	NEG	POS	NEG	POS	NEG	POS	POS	POS	NEG	NEG
POS	NEG	AMB	NEG	NEG	NEG	POS	POS	NEG	NEG	POS	NEG	NEG	NEG	NEG	n/a	POS	POS	NEG	POS	NEG	NEG	POS	POS	POS	NEG	POS	NEG	POS	POS	NEG	NEG	NEG	NEG	POS	AMB	POS	AMB	POS	NEG	POS	POS	POS	NEG	NEG
POS	NEG	AMB	NEG	NEG	NEG	POS	POS	NEG	NEG	POS	NEG	NEG	NEG	NEG	n/a	POS	POS	NEG	POS	NEG	NEG	POS	POS	POS	NEG	POS	NEG	POS	POS	NEG	NEG	AMB	NEG	POS	AMB	POS	AMB	POS	NEG	POS	POS	POS	NEG	NEG
POS	NEG	AMB	NEG	NEG	NEG	POS	POS	NEG	NEG	POS	NEG	NEG	NEG	NEG	n/a	POS	POS	NEG	POS	NEG	NEG	POS	POS	POS	NEG	POS	NEG	POS	POS	NEG	NEG	NEG	NEG	POS	AMB	POS	AMB	POS	NEG	POS	POS	POS	NEG	NEG
POS	NEG	AMB	NEG	NEG	NEG	POS	POS	NEG	NEG	POS	NEG	NEG	NEG	NEG	n/a	POS	POS	NEG	POS	NEG	NEG	POS	POS	POS	NEG	POS	NEG	POS	POS	NEG	NEG	NEG	NEG	POS	AMB	POS	AMB	POS	NEG	POS	POS	POS	NEG	NEG
POS	NEG	AMB	NEG	NEG	NEG	POS	POS	NEG	NEG	POS	NEG	NEG	NEG	NEG	n/a	POS	POS	NEG	POS	NEG	NEG	POS	POS	POS	NEG	POS	NEG	POS	POS	NEG	NEG	NEG	NEG	POS	AMB	POS	AMB	POS	NEG	POS	POS	POS	NEG	NEG
POS	POS	AMB	AMB	NEG	NEG	NEG	POS	POS	NEG	NEG	POS	POS	NEG	POS	n/a	POS	POS	NEG	POS	NEG	NEG	POS	NEG	NEG	NEG	NEG	NEG	POS	POS	POS	NEG	NEG	NEG	POS	AMB	POS	AMB	POS	NEG	POS	POS	POS	NEG	NEG
POS	POS	AMB	NEG	NEG	NEG	NEG	POS	POS	NEG	NEG	POS	POS	NEG	POS	n/a	POS	POS	NEG	POS	NEG	NEG	POS	NEG	NEG	NEG	NEG	NEG	POS	POS	POS	NEG	NEG	NEG	POS	NEG	POS	NEG	POS	NEG	POS	POS	POS	NEG	NEG
POS	POS	AMB	NEG	NEG	NEG	NEG	POS	POS	NEG	NEG	POS	POS	NEG	POS	n/a	POS	POS	NEG	POS	NEG	NEG	POS	POS	POS	POS	NEG	NEG	POS	POS	POS	NEG	NEG	NEG	POS	AMB	POS	AMB	POS	NEG	POS	POS	POS	NEG	NEG
POS	POS	AMB	NEG	NEG	NEG	NEG	POS	POS	NEG	NEG	POS	POS	NEG	POS	n/a	POS	POS	NEG	POS	NEG	NEG	POS	POS	POS	POS	NEG	NEG	POS	POS	POS	NEG	NEG	NEG	POS	AMB	POS	NEG	POS	NEG	POS	POS	POS	NEG	NEG
POS	POS	AMB	NEG	NEG	NEG	NEG	POS	POS	NEG	NEG	POS	POS	NEG	POS	n/a	POS	POS	NEG	POS	NEG	NEG	POS	POS	POS	POS	NEG	NEG	POS	POS	POS	NEG	NEG	NEG	POS	AMB	POS	AMB	POS	NEG	POS	POS	POS	NEG	NEG
POS	POS	AMB	NEG	NEG	NEG	NEG	POS	POS	NEG	NEG	POS	POS	NEG	POS	n/a	POS	POS	NEG	POS	NEG	NEG	POS	POS	POS	POS	NEG	NEG	POS	POS	POS	NEG	NEG	NEG	POS	AMB	POS	AMB	POS	NEG	POS	POS	POS	NEG	NEG
POS	POS	AMB	NEG	NEG	NEG	NEG	POS	POS	NEG	NEG	POS	POS	NEG	POS	n/a	POS	POS	NEG	POS	NEG	NEG	POS	POS	POS	POS	NEG	NEG	POS	POS	POS	NEG	NEG	NEG	POS	AMB	POS	AMB	POS	NEG	POS	POS	POS	NEG	NEG
POS	POS	AMB	AMB	NEG	NEG	NEG	POS	POS	NEG	NEG	POS	POS	NEG	POS	n/a	POS	POS	NEG	POS	NEG	NEG	POS	POS	POS	POS	NEG	NEG	POS	POS	POS	NEG	NEG	NEG	POS	AMB	POS	AMB	POS	NEG	POS	POS	POS	NEG	NEG
POS	POS	AMB	AMB	NEG	NEG	NEG	POS	POS	NEG	NEG	POS	POS	NEG	POS	n/a	POS	POS	NEG	POS	NEG	NEG	POS	POS	POS	POS	NEG	NEG	POS	POS	POS	NEG	NEG	NEG	POS	AMB	POS	AMB	POS	NEG	POS	POS	POS	NEG	NEG
POS	POS	AMB	NEG	NEG	NEG	NEG	POS	POS	NEG	NEG	POS	POS	NEG	POS	NEG	POS	POS	NEG	POS	NEG	NEG	POS	POS	POS	POS	NEG	NEG	POS	POS	POS	NEG	NEG	NEG	POS	AMB	POS	NEG	POS	NEG	POS	POS	POS	NEG	NEG
POS	POS	AMB	NEG	NEG	NEG	NEG	POS	POS	NEG	NEG	POS	POS	NEG	POS	NEG	POS	POS	NEG	POS	NEG	NEG	POS	POS	POS	POS	NEG	NEG	POS	POS	POS	NEG	NEG	NEG	POS	AMB	POS	AMB	POS	NEG	POS	POS	POS	NEG	NEG
POS	POS	AMB	AMB	NEG	NEG	NEG	POS	POS	NEG	NEG	POS	POS	NEG	POS	n/a	POS	POS	NEG	POS	NEG	NEG	POS	POS	POS	POS	NEG	NEG	POS	POS	POS	NEG	NEG	NEG	POS	AMB	POS	AMB	POS	NEG	POS	POS	POS	NEG	NEG
POS	POS	AMB	NEG	NEG	NEG	NEG	POS	POS	NEG	NEG	POS	POS	NEG	POS	n/a	POS	POS	NEG	POS	NEG	NEG	POS	POS	POS	POS	NEG	NEG	POS	POS	POS	NEG	NEG	NEG	POS	AMB	POS	AMB	POS	NEG	POS	POS	POS	NEG	NEG
POS	POS	AMB	NEG	NEG	NEG	NEG	POS	POS	NEG	NEG	POS	POS	NEG	POS	n/a	POS	POS	NEG	POS	NEG	NEG	POS	POS	POS	POS	NEG	NEG	POS	POS	POS	NEG	NEG	NEG	POS	AMB	POS	NEG	POS	NEG	POS	POS	POS	NEG	NEG
POS	POS	AMB	NEG	NEG	NEG	NEG	POS	POS	NEG	NEG	POS	POS	NEG	POS	NEG	POS	POS	NEG	POS	NEG	NEG	POS	POS	POS	POS	NEG	NEG	POS	POS	POS	NEG	NEG	NEG	POS	AMB	POS	AMB	POS	NEG	POS	POS	POS	NEG	NEG
POS	NEG	POS	AMB	NEG	NEG	NEG	POS	POS	AMB	POS	NEG	NEG	NEG	NEG	n/a	POS	POS	NEG	NEG	POS	POS	POS	NEG	NEG	NEG	NEG	NEG	POS	POS	NEG	POS	NEG	NEG	POS	AMB	AMB	POS	POS	NEG	POS	POS	POS	NEG	NEG
POS	NEG	AMB	NEG	NEG	NEG	POS	POS	POS	NEG	POS	NEG	NEG	NEG	NEG	n/a	POS	POS	NEG	POS	NEG	NEG	POS	NEG	NEG	NEG	NEG	NEG	POS	POS	NEG	NEG	NEG	NEG	POS	AMB	POS	AMB	POS	NEG	POS	POS	POS	NEG	NEG
POS	NEG	POS	AMB	NEG	AMB	NEG	NEG	NEG	NEG	NEG	POS	NEG	POS	POS	n/a	POS	POS	NEG	POS	NEG	NEG	POS	POS	POS	NEG	POS	NEG	POS	POS	NEG	NEG	NEG	NEG	POS	AMB	POS	AMB	POS	POS	NEG	POS	POS	NEG	NEG
NEG	NEG	NEG	NEG	NEG	NEG	NEG	POS	POS	NEG	POS	POS	NEG	POS	POS	n/a	POS	POS	NEG	POS	NEG	NEG	POS	POS	POS	NEG	POS	NEG	POS	POS	NEG	NEG	NEG	NEG	POS	AMB	POS	AMB	POS	POS	NEG	POS	POS	NEG	NEG
NEG	NEG	NEG	NEG	NEG	NEG	NEG	POS	POS	NEG	POS	POS	NEG	POS	POS	n/a	POS	POS	NEG	POS	NEG	NEG	POS	POS	POS	NEG	POS	NEG	POS	POS	NEG	NEG	NEG	NEG	POS	AMB	POS	AMB	POS	POS	NEG	POS	POS	NEG	NEG
POS	NEG	AMB	NEG	POS	NEG	NEG	POS	NEG	NEG	POS	POS	NEG	POS	POS	NEG	POS	POS	NEG	NEG	POS	NEG	POS	POS	POS	NEG	NEG	POS	POS	POS	NEG	NEG	NEG	POS	NEG	POS	AMB	POS	POS	NEG	POS	POS	POS	NEG	NEG
POS	NEG	AMB	POS	NEG	NEG	NEG	POS	NEG	POS	NEG	NEG	NEG	NEG	NEG	n/a	POS	POS	NEG	NEG	NEG	NEG	NEG	POS	POS	NEG	NEG	POS	POS	POS	NEG	POS	NEG	NEG	NEG	POS	NEG	POS	POS	POS	NEG	POS	POS	NEG	NEG
POS	NEG	POS	AMB	NEG	NEG	NEG	POS	NEG	POS	NEG	NEG	NEG	NEG	NEG	n/a	POS	POS	POS	NEG	NEG	NEG	NEG	POS	POS	POS	NEG	NEG	POS	NEG	NEG	POS	NEG	NEG	NEG	POS	NEG	NEG	POS	POS	NEG	POS	POS	NEG	NEG
POS	NEG	POS	AMB	NEG	NEG	NEG	POS	NEG	POS	NEG	NEG	NEG	NEG	NEG	n/a	POS	POS	POS	NEG	NEG	NEG	NEG	POS	POS	POS	NEG	NEG	POS	NEG	NEG	POS	NEG	NEG	NEG	POS	NEG	NEG	POS	POS	AMB	POS	POS	NEG	NEG
POS	NEG	POS	AMB	NEG	NEG	NEG	POS	NEG	POS	NEG	NEG	NEG	NEG	NEG	n/a	POS	POS	POS	NEG	NEG	NEG	NEG	NEG	NEG	NEG	NEG	NEG	POS	NEG	NEG	POS	NEG	NEG	NEG	POS	NEG	POS	POS	POS	NEG	POS	POS	NEG	NEG
POS	NEG	POS	AMB	NEG	AMB	NEG	POS	NEG	POS	NEG	NEG	NEG	NEG	NEG	n/a	POS	POS	POS	NEG	NEG	NEG	NEG	NEG	NEG	NEG	NEG	NEG	POS	AMB	NEG	POS	NEG	NEG	NEG	POS	NEG	POS	POS	POS	AMB	POS	POS	NEG	NEG
POS	NEG	POS	NEG	NEG	NEG	NEG	POS	POS	NEG	NEG	POS	NEG	POS	POS	n/a	POS	POS	NEG	NEG	NEG	POS	NEG	POS	POS	NEG	NEG	NEG	POS	POS	NEG	NEG	NEG	NEG	POS	NEG	POS	NEG	POS	NEG	POS	NEG	NEG	POS	POS
POS	NEG	POS	NEG	NEG	NEG	NEG	POS	NEG	NEG	POS	POS	NEG	POS	POS	n/a	POS	POS	NEG	NEG	POS	POS	POS	POS	POS	NEG	POS	NEG	POS	POS	NEG	NEG	NEG	NEG	POS	AMB	POS	NEG	POS	NEG	POS	POS	POS	NEG	NEG
POS	NEG	POS	AMB	NEG	NEG	NEG	POS	NEG	NEG	POS	POS	NEG	POS	POS	n/a	POS	POS	NEG	NEG	POS	POS	POS	POS	POS	NEG	POS	NEG	POS	POS	NEG	NEG	NEG	NEG	POS	AMB	POS	AMB	POS	NEG	POS	POS	POS	NEG	NEG
POS	NEG	POS	NEG	NEG	NEG	NEG	POS	NEG	NEG	POS	POS	NEG	POS	POS	n/a	POS	POS	NEG	NEG	POS	NEG	POS	POS	POS	NEG	POS	NEG	POS	POS	NEG	NEG	NEG	NEG	POS	NEG	POS	NEG	POS	NEG	POS	POS	POS	NEG	NEG
POS	NEG	POS	AMB	NEG	NEG	NEG	POS	NEG	NEG	POS	POS	NEG	POS	POS	n/a	POS	POS	NEG	NEG	POS	POS	POS	POS	POS	NEG	POS	NEG	POS	POS	NEG	NEG	NEG	NEG	POS	AMB	POS	AMB	POS	NEG	POS	POS	POS	NEG	NEG
POS	NEG	POS	NEG	NEG	NEG	NEG	POS	NEG	NEG	POS	POS	NEG	POS	POS	NEG	POS	POS	NEG	NEG	POS	POS	POS	POS	POS	NEG	POS	NEG	POS	POS	NEG	NEG	NEG	NEG	POS	AMB	POS	AMB	POS	NEG	POS	POS	POS	NEG	NEG
POS	NEG	POS	AMB	NEG	NEG	NEG	POS	NEG	NEG	POS	POS	NEG	POS	POS	NEG	POS	POS	NEG	NEG	POS	POS	POS	POS	POS	NEG	NEG	POS	POS	POS	NEG	NEG	POS	NEG	POS	AMB	POS	AMB	POS	NEG	POS	POS	POS	NEG	NEG
POS	NEG	POS	AMB	NEG	NEG	NEG	POS	POS	NEG	POS	POS	POS	NEG	POS	n/a	POS	POS	NEG	NEG	POS	POS	POS	POS	POS	NEG	POS	NEG	POS	POS	NEG	NEG	NEG	POS	POS	AMB	POS	AMB	POS	NEG	POS	POS	POS	NEG	NEG
POS	NEG	POS	AMB	NEG	NEG	NEG	POS	POS	NEG	POS	POS	POS	NEG	POS	n/a	POS	POS	NEG	NEG	POS	POS	POS	POS	POS	NEG	POS	NEG	POS	POS	NEG	NEG	NEG	POS	POS	AMB	POS	AMB	POS	NEG	POS	POS	POS	NEG	NEG
POS	NEG	POS	AMB	NEG	NEG	NEG	POS	POS	NEG	POS	POS	POS	NEG	POS	n/a	POS	POS	NEG	NEG	POS	POS	POS	POS	POS	NEG	POS	NEG	POS	POS	NEG	NEG	NEG	POS	POS	AMB	POS	AMB	POS	NEG	POS	POS	POS	NEG	NEG
POS	NEG	POS	AMB	NEG	NEG	NEG	POS	POS	NEG	POS	POS	POS	NEG	POS	n/a	POS	POS	NEG	NEG	POS	POS	POS	POS	POS	NEG	POS	NEG	POS	POS	NEG	NEG	NEG	POS	POS	AMB	POS	AMB	POS	NEG	POS	POS	POS	NEG	NEG
POS	NEG	POS	AMB	NEG	NEG	NEG	POS	POS	NEG	POS	POS	POS	NEG	POS	n/a	POS	POS	NEG	NEG	POS	POS	POS	POS	POS	NEG	POS	NEG	POS	POS	NEG	NEG	NEG	POS	POS	AMB	POS	AMB	POS	NEG	POS	POS	POS	NEG	NEG
POS	NEG	POS	AMB	NEG	NEG	NEG	POS	POS	NEG	POS	POS	POS	NEG	POS	n/a	POS	POS	NEG	NEG	POS	POS	POS	POS	POS	NEG	POS	NEG	POS	POS	NEG	NEG	NEG	POS	POS	AMB	POS	AMB	POS	NEG	POS	POS	POS	NEG	NEG
POS	NEG	POS	AMB	NEG	NEG	NEG	POS	POS	NEG	POS	POS	POS	NEG	POS	n/a	POS	POS	NEG	NEG	POS	POS	POS	POS	POS	NEG	POS	NEG	POS	POS	NEG	NEG	NEG	POS	POS	AMB	POS	AMB	POS	NEG	POS	POS	POS	NEG	NEG
POS	NEG	POS	AMB	NEG	NEG	NEG	POS	NEG	NEG	POS	POS	POS	NEG	POS	n/a	POS	POS	NEG	NEG	POS	NEG	POS	POS	POS	NEG	POS	NEG	POS	POS	NEG	NEG	NEG	POS	POS	NEG	POS	NEG	POS	NEG	POS	POS	POS	NEG	NEG
POS	NEG	POS	AMB	NEG	NEG	NEG	POS	NEG	NEG	POS	POS	POS	NEG	POS	n/a	POS	POS	NEG	NEG	POS	NEG	POS	POS	POS	NEG	POS	NEG	POS	POS	NEG	NEG	NEG	POS	POS	NEG	POS	NEG	POS	NEG	POS	POS	POS	NEG	NEG
POS	NEG	POS	AMB	NEG	NEG	NEG	POS	NEG	NEG	POS	POS	POS	NEG	POS	n/a	POS	POS	NEG	NEG	POS	NEG	POS	POS	POS	NEG	POS	NEG	POS	POS	NEG	NEG	NEG	POS	POS	AMB	POS	NEG	POS	NEG	POS	POS	POS	NEG	NEG
POS	NEG	POS	NEG	NEG	NEG	NEG	POS	NEG	NEG	POS	NEG	NEG	NEG	NEG	n/a	POS	POS	NEG	NEG	POS	NEG	POS	POS	POS	POS	NEG	NEG	POS	POS	NEG	NEG	NEG	NEG	POS	AMB	AMB	POS	POS	POS	NEG	POS	POS	NEG	NEG
POS	NEG	POS	NEG	NEG	NEG	NEG	POS	NEG	NEG	POS	NEG	NEG	NEG	NEG	n/a	POS	POS	NEG	NEG	POS	NEG	POS	POS	POS	POS	NEG	NEG	POS	POS	NEG	NEG	NEG	NEG	POS	AMB	AMB	POS	POS	POS	NEG	POS	POS	NEG	NEG
NEG	NEG	NEG	NEG	NEG	NEG	NEG	POS	POS	NEG	NEG	NEG	NEG	NEG	NEG	n/a	POS	POS	NEG	POS	NEG	NEG	NEG	POS	POS	NEG	NEG	NEG	POS	POS	POS	NEG	NEG	NEG	POS	AMB	POS	NEG	POS	NEG	POS	NEG	NEG	POS	POS
POS	NEG	POS	AMB	NEG	NEG	NEG	NEG	NEG	NEG	NEG	NEG	NEG	NEG	NEG	n/a	NEG	NEG	NEG	NEG	NEG	NEG	NEG	POS	POS	POS	NEG	NEG	POS	POS	NEG	NEG	NEG	NEG	POS	AMB	POS	NEG	POS	NEG	NEG	NEG	NEG	POS	POS
POS	NEG	POS	AMB	NEG	NEG	NEG	NEG	NEG	NEG	NEG	NEG	NEG	NEG	NEG	n/a	NEG	NEG	NEG	NEG	NEG	NEG	NEG	POS	POS	POS	NEG	NEG	POS	POS	NEG	NEG	NEG	NEG	POS	AMB	POS	NEG	POS	NEG	NEG	NEG	NEG	POS	POS
POS	NEG	POS	AMB	NEG	NEG	NEG	NEG	NEG	NEG	NEG	NEG	NEG	NEG	NEG	n/a	NEG	NEG	NEG	NEG	NEG	NEG	NEG	POS	POS	POS	NEG	NEG	POS	POS	NEG	NEG	NEG	NEG	POS	AMB	POS	NEG	POS	NEG	NEG	NEG	NEG	POS	POS
POS	NEG	POS	AMB	NEG	NEG	NEG	NEG	NEG	NEG	NEG	NEG	NEG	NEG	NEG	n/a	NEG	NEG	NEG	NEG	NEG	NEG	NEG	POS	POS	POS	NEG	NEG	POS	POS	NEG	NEG	NEG	NEG	POS	AMB	POS	NEG	POS	NEG	NEG	NEG	NEG	POS	POS
POS	NEG	POS	AMB	NEG	NEG	NEG	NEG	NEG	NEG	NEG	NEG	NEG	NEG	NEG	n/a	NEG	NEG	NEG	NEG	NEG	NEG	NEG	POS	POS	POS	NEG	NEG	POS	POS	NEG	NEG	NEG	NEG	POS	AMB	POS	NEG	POS	NEG	NEG	NEG	NEG	POS	POS
POS	NEG	POS	AMB	NEG	NEG	NEG	NEG	NEG	NEG	NEG	NEG	NEG	NEG	NEG	n/a	NEG	NEG	NEG	NEG	NEG	NEG	NEG	POS	POS	POS	NEG	NEG	POS	POS	NEG	NEG	NEG	NEG	POS	AMB	POS	NEG	POS	NEG	NEG	NEG	NEG	POS	POS
POS	NEG	POS	AMB	NEG	NEG	NEG	NEG	NEG	NEG	NEG	NEG	NEG	NEG	NEG	n/a	NEG	NEG	NEG	NEG	NEG	NEG	NEG	POS	POS	POS	NEG	NEG	POS	POS	NEG	NEG	NEG	NEG	POS	AMB	POS	NEG	POS	NEG	NEG	NEG	NEG	POS	POS
POS	NEG	POS	AMB	NEG	NEG	NEG	NEG	NEG	NEG	NEG	NEG	NEG	NEG	NEG	n/a	NEG	NEG	NEG	NEG	NEG	NEG	NEG	POS	POS	POS	NEG	NEG	POS	POS	NEG	NEG	NEG	NEG	POS	AMB	POS	NEG	POS	NEG	NEG	NEG	NEG	POS	POS
POS	NEG	POS	AMB	NEG	NEG	NEG	NEG	NEG	NEG	NEG	NEG	NEG	NEG	NEG	n/a	NEG	NEG	NEG	NEG	NEG	NEG	NEG	POS	POS	POS	NEG	NEG	POS	POS	NEG	NEG	NEG	NEG	AMB	POS	POS	NEG	POS	NEG	NEG	NEG	NEG	POS	POS
POS	NEG	POS	AMB	NEG	NEG	NEG	NEG	NEG	NEG	NEG	NEG	NEG	NEG	NEG	n/a	NEG	NEG	NEG	NEG	NEG	NEG	NEG	POS	POS	POS	NEG	NEG	POS	POS	NEG	NEG	NEG	NEG	POS	AMB	POS	NEG	POS	NEG	NEG	NEG	NEG	POS	POS
POS	NEG	POS	AMB	NEG	NEG	NEG	NEG	NEG	NEG	NEG	NEG	NEG	NEG	NEG	n/a	NEG	NEG	NEG	NEG	NEG	NEG	NEG	POS	POS	POS	NEG	NEG	POS	POS	NEG	NEG	NEG	NEG	POS	AMB	POS	NEG	POS	NEG	NEG	NEG	NEG	POS	POS
POS	NEG	POS	AMB	NEG	NEG	NEG	NEG	NEG	NEG	NEG	NEG	NEG	NEG	NEG	n/a	NEG	NEG	NEG	NEG	NEG	NEG	NEG	POS	NEG	POS	NEG	NEG	POS	POS	NEG	NEG	NEG	NEG	POS	AMB	NEG	NEG	POS	NEG	NEG	NEG	NEG	NEG	POS
POS	NEG	POS	AMB	NEG	NEG	NEG	NEG	NEG	NEG	NEG	NEG	NEG	NEG	NEG	n/a	NEG	NEG	NEG	NEG	NEG	NEG	NEG	POS	POS	POS	NEG	NEG	POS	POS	NEG	NEG	NEG	NEG	POS	AMB	POS	NEG	POS	NEG	NEG	NEG	NEG	POS	POS
POS	NEG	POS	AMB	NEG	NEG	NEG	NEG	NEG	NEG	NEG	NEG	NEG	NEG	NEG	n/a	NEG	NEG	NEG	NEG	NEG	NEG	NEG	POS	POS	POS	NEG	NEG	POS	POS	NEG	NEG	NEG	NEG	POS	AMB	POS	NEG	POS	NEG	NEG	NEG	NEG	POS	POS
POS	NEG	POS	AMB	NEG	NEG	NEG	NEG	NEG	NEG	NEG	NEG	NEG	NEG	NEG	n/a	NEG	NEG	NEG	NEG	NEG	NEG	NEG	POS	POS	POS	NEG	NEG	POS	POS	NEG	NEG	NEG	NEG	POS	AMB	POS	NEG	POS	NEG	NEG	NEG	NEG	POS	POS
POS	NEG	POS	AMB	NEG	NEG	NEG	NEG	NEG	NEG	NEG	NEG	NEG	NEG	NEG	n/a	NEG	NEG	NEG	NEG	NEG	NEG	NEG	POS	POS	POS	NEG	NEG	POS	POS	NEG	NEG	NEG	NEG	POS	AMB	POS	NEG	POS	NEG	NEG	NEG	NEG	POS	POS
NEG	NEG	NEG	NEG	NEG	NEG	NEG	POS	POS	NEG	POS	NEG	NEG	NEG	NEG	n/a	POS	POS	NEG	POS	NEG	NEG	NEG	POS	POS	NEG	POS	NEG	POS	POS	NEG	POS	NEG	NEG	POS	AMB	POS	AMB	POS	NEG	POS	POS	POS	NEG	NEG
POS	POS	AMB	NEG	NEG	NEG	NEG	POS	POS	NEG	NEG	POS	POS	NEG	POS	POS	POS	POS	NEG	POS	NEG	NEG	POS	POS	POS	POS	NEG	NEG	POS	POS	POS	NEG	NEG	NEG	NEG	POS	POS	AMB	POS	NEG	POS	POS	POS	NEG	NEG
POS	POS	AMB	AMB	NEG	NEG	NEG	POS	POS	NEG	NEG	POS	POS	NEG	POS	n/a	POS	POS	NEG	POS	NEG	NEG	POS	POS	POS	POS	NEG	NEG	POS	POS	POS	NEG	NEG	NEG	NEG	POS	POS	AMB	POS	NEG	POS	POS	POS	NEG	NEG
POS	POS	AMB	NEG	NEG	NEG	NEG	POS	POS	NEG	NEG	POS	POS	NEG	POS	POS	POS	POS	NEG	POS	NEG	NEG	POS	POS	POS	POS	NEG	NEG	POS	POS	POS	NEG	NEG	NEG	NEG	POS	POS	AMB	POS	NEG	POS	POS	POS	NEG	NEG
POS	POS	AMB	NEG	NEG	NEG	NEG	POS	POS	NEG	NEG	POS	POS	NEG	POS	n/a	POS	POS	NEG	POS	NEG	NEG	POS	POS	POS	POS	NEG	NEG	POS	POS	POS	NEG	NEG	NEG	NEG	POS	POS	AMB	POS	NEG	POS	POS	POS	NEG	NEG
POS	POS	AMB	NEG	NEG	NEG	NEG	POS	POS	NEG	NEG	POS	POS	NEG	POS	POS	POS	POS	NEG	POS	NEG	NEG	POS	POS	POS	POS	NEG	NEG	POS	POS	POS	NEG	NEG	NEG	NEG	POS	POS	AMB	POS	NEG	POS	POS	POS	NEG	NEG
POS	POS	AMB	AMB	NEG	NEG	NEG	POS	POS	NEG	NEG	POS	POS	NEG	POS	POS	POS	POS	NEG	POS	NEG	NEG	POS	POS	POS	POS	NEG	NEG	POS	POS	POS	NEG	NEG	NEG	NEG	POS	POS	AMB	POS	NEG	POS	POS	POS	NEG	NEG
POS	POS	AMB	NEG	NEG	NEG	NEG	POS	POS	NEG	NEG	POS	POS	NEG	POS	n/a	POS	POS	NEG	POS	NEG	NEG	POS	POS	POS	POS	NEG	NEG	POS	POS	POS	NEG	NEG	NEG	NEG	POS	POS	AMB	POS	NEG	POS	POS	POS	NEG	NEG
POS	NEG	POS	NEG	NEG	NEG	NEG	POS	POS	POS	NEG	NEG	NEG	NEG	NEG	n/a	POS	POS	NEG	NEG	AMB	AMB	NEG	POS	POS	NEG	NEG	NEG	POS	POS	NEG	NEG	NEG	NEG	NEG	POS	NEG	NEG	POS	POS	NEG	POS	POS	NEG	NEG
POS	NEG	POS	AMB	NEG	NEG	NEG	POS	POS	POS	NEG	NEG	NEG	NEG	NEG	n/a	POS	POS	NEG	NEG	AMB	POS	NEG	POS	POS	NEG	NEG	NEG	POS	POS	NEG	NEG	NEG	NEG	NEG	POS	NEG	NEG	POS	POS	NEG	POS	POS	NEG	NEG
POS	NEG	POS	AMB	NEG	NEG	NEG	POS	POS	POS	NEG	NEG	NEG	NEG	NEG	n/a	POS	POS	NEG	NEG	AMB	POS	NEG	POS	POS	NEG	NEG	NEG	POS	POS	NEG	NEG	NEG	NEG	NEG	POS	NEG	NEG	POS	POS	NEG	POS	POS	NEG	NEG
POS	NEG	POS	AMB	NEG	NEG	NEG	POS	POS	POS	NEG	NEG	NEG	NEG	NEG	n/a	POS	POS	NEG	NEG	POS	POS	NEG	POS	POS	NEG	NEG	NEG	POS	POS	NEG	NEG	NEG	NEG	NEG	POS	NEG	NEG	POS	POS	NEG	POS	POS	NEG	NEG
POS	NEG	POS	AMB	NEG	NEG	NEG	POS	POS	POS	AMB	NEG	NEG	NEG	NEG	n/a	POS	POS	NEG	NEG	POS	POS	NEG	POS	POS	NEG	POS	NEG	POS	POS	NEG	NEG	NEG	NEG	NEG	POS	NEG	POS	POS	POS	AMB	POS	POS	NEG	NEG
POS	NEG	POS	AMB	NEG	NEG	NEG	NEG	NEG	NEG	NEG	NEG	NEG	NEG	NEG	n/a	POS	POS	NEG	NEG	NEG	NEG	NEG	POS	POS	NEG	NEG	POS	POS	POS	NEG	NEG	NEG	NEG	NEG	POS	POS	AMB	NEG	NEG	NEG	NEG	POS	NEG	NEG
POS	NEG	POS	NEG	NEG	NEG	NEG	NEG	NEG	NEG	NEG	NEG	NEG	NEG	NEG	n/a	POS	POS	NEG	NEG	NEG	NEG	NEG	POS	POS	NEG	NEG	POS	NEG	NEG	NEG	NEG	NEG	NEG	NEG	POS	POS	AMB	NEG	NEG	NEG	NEG	POS	NEG	NEG

TYPE I RESTRICTION-MODIFICATION SYSTEM, SINGLE SEQUENCE SPECIFICITY PROTEIN													MISCELLANEOUS GENES				HYALURONATE LYASE							
hsdS1	hsdS2				hsdS3					hsdSx			ear2	Q2YUB3	Q7A4X2	sdrM	hysA1			hysA2				
hsdS1-RF122	hsdS2 (ST5+ST8)	hsdS2 (MW2+476)	hsdS2 (RF122)	hsdS2 (MRSA252)	hsdS3 (AllOtherThanRF122+252)	hsdS3 (ST8+ST1+RF122)	hsdS3 (Mu50+N315)	hsdS3 (CC51+252)	hsdS3 (MRSA252)	hsdSx (CC25)	hsdSx (CC15)	hsdSx (etd)					hysA1 (MRSA252)	hysA1 (MRSA252+RF122) and/or hysA2 (cons)	hysA1 (MRSA252+RF122) and/or hysA2 (COL+USA300)	hysA2 (All Other Than MRSA252)	hysA2 (COL+USA300+NCTC8325)	hysA2 (All Other Than COL+USA300+NCTC8325)	hysA2 (All Other Than COL+USA300+NCTC8325)	hysA2 (MRSA252)
type I site-specific deoxyribo-nuclease subunit, 1st locus	type I site-specific deoxyribonuclease subunit, 2nd locus				type I site-specific deoxyribonuclease subunit, 3rd locus					type I site-specific deoxyribonuclease subunit, unknown locus			Putative protein	Multidrug resistance transporter	Putative protein	Multidrug resistance transporter	Hyaluronate lyase, variable first / second locus			Hyaluronate lyase, second locus				
NEG	NEG	POS	NEG	NEG	POS	POS	NEG	NEG	NEG	POS	NEG	NEG	POS	NEG	NEG	POS	NEG	POS	NEG	POS	NEG	POS	POS	NEG
NEG	NEG	POS	NEG	NEG	POS	POS	NEG	NEG	NEG	POS	NEG	NEG	POS	NEG	NEG	POS	NEG	POS	NEG	POS	NEG	POS	POS	NEG
NEG	NEG	POS	NEG	NEG	POS	POS	NEG	NEG	NEG	POS	NEG	NEG	POS	NEG	NEG	POS	NEG	POS	NEG	POS	NEG	POS	NEG	NEG
NEG	NEG	POS	NEG	NEG	POS	POS	NEG	NEG	NEG	POS	NEG	NEG	POS	NEG	NEG	POS	NEG	POS	NEG	POS	NEG	POS	AMB	NEG
NEG	NEG	POS	NEG	NEG	NEG	POS	NEG	NEG	NEG	POS	NEG	NEG	POS	NEG	NEG	POS	NEG	POS	NEG	NEG	NEG	POS	NEG	NEG
NEG	POS	NEG	NEG	NEG	NEG	NEG	POS	NEG	NEG	POS	NEG	NEG	NEG	NEG	POS	POS	NEG	POS	NEG	POS	NEG	NEG	POS	NEG
NEG	POS	NEG	NEG	NEG	POS	NEG	POS	NEG	NEG	POS	NEG	NEG	NEG	NEG	POS	POS	NEG	POS	NEG	POS	NEG	POS	POS	NEG
NEG	POS	NEG	NEG	NEG	NEG	NEG	POS	NEG	NEG	POS	NEG	NEG	NEG	NEG	POS	POS	NEG	POS	NEG	AMB	NEG	NEG	POS	NEG
NEG	NEG	NEG	NEG	NEG	POS	POS	NEG	POS	NEG	POS	NEG	NEG	POS	NEG	NEG	POS	NEG	POS	NEG	NEG	NEG	POS	POS	NEG
NEG	NEG	NEG	NEG	NEG	POS	POS	NEG	POS	NEG	POS	NEG	NEG	POS	NEG	NEG	POS	NEG	POS	NEG	NEG	NEG	POS	POS	NEG
NEG	NEG	NEG	NEG	NEG	POS	POS	NEG	POS	NEG	POS	NEG	NEG	POS	NEG	NEG	POS	NEG	POS	NEG	NEG	NEG	POS	POS	NEG
NEG	NEG	NEG	NEG	NEG	POS	POS	NEG	POS	NEG	POS	AMB	NEG	POS	NEG	NEG	POS	NEG	POS	NEG	NEG	NEG	POS	POS	NEG
NEG	NEG	NEG	NEG	NEG	AMB	POS	NEG	POS	NEG	POS	NEG	NEG	POS	NEG	NEG	POS	NEG	POS	NEG	NEG	NEG	NEG	POS	NEG
NEG	NEG	NEG	NEG	NEG	POS	POS	NEG	POS	NEG	POS	NEG	NEG	POS	NEG	NEG	POS	NEG	POS	NEG	NEG	NEG	POS	POS	NEG
NEG	NEG	NEG	NEG	NEG	POS	POS	NEG	POS	NEG	POS	NEG	NEG	POS	NEG	NEG	POS	NEG	POS	NEG	NEG	NEG	POS	POS	NEG
NEG	NEG	NEG	NEG	NEG	POS	POS	NEG	POS	NEG	POS	AMB	NEG	POS	NEG	NEG	POS	NEG	POS	NEG	NEG	NEG	POS	POS	NEG
NEG	NEG	NEG	NEG	NEG	POS	POS	NEG	POS	NEG	POS	NEG	NEG	POS	NEG	NEG	POS	NEG	POS	NEG	NEG	NEG	POS	POS	NEG
NEG	NEG	NEG	NEG	NEG	POS	POS	NEG	POS	NEG	POS	NEG	NEG	POS	NEG	NEG	POS	NEG	POS	NEG	NEG	NEG	POS	POS	NEG
NEG	NEG	NEG	NEG	NEG	NEG	POS	NEG	POS	NEG	POS	NEG	NEG	POS	NEG	NEG	POS	NEG	POS	NEG	NEG	NEG	NEG	POS	NEG
NEG	NEG	NEG	NEG	NEG	NEG	NEG	NEG	NEG	NEG	NEG	AMB	POS	NEG	NEG	POS	POS	NEG	POS	NEG	NEG	NEG	POS	POS	NEG
NEG	NEG	NEG	NEG	NEG	NEG	NEG	NEG	NEG	NEG	NEG	AMB	POS	NEG	NEG	POS	POS	NEG	POS	NEG	NEG	NEG	POS	POS	NEG
NEG	NEG	NEG	NEG	NEG	NEG	NEG	NEG	NEG	NEG	NEG	NEG	POS	NEG	NEG	POS	POS	NEG	POS	NEG	NEG	NEG	POS	POS	NEG
NEG	NEG	NEG	NEG	NEG	NEG	NEG	NEG	NEG	NEG	NEG	NEG	POS	NEG	NEG	POS	POS	NEG	POS	NEG	NEG	NEG	POS	POS	NEG
NEG	NEG	NEG	NEG	NEG	NEG	NEG	NEG	NEG	NEG	NEG	NEG	POS	NEG	NEG	POS	POS	NEG	POS	NEG	NEG	NEG	POS	POS	NEG
NEG	POS	NEG	NEG	NEG	POS	POS	NEG	NEG	NEG	POS	NEG	NEG	POS	NEG	NEG	POS	NEG	POS	POS	POS	POS	NEG	NEG	NEG
NEG	POS	NEG	NEG	NEG	NEG	POS	NEG	NEG	NEG	POS	NEG	NEG	POS	NEG	NEG	POS	NEG	POS	NEG	NEG	POS	NEG	NEG	NEG
NEG	POS	NEG	NEG	NEG	NEG	NEG	NEG	NEG	NEG	POS	NEG	NEG	NEG	NEG	NEG	POS	NEG	POS	POS	POS	POS	NEG	NEG	NEG
NEG	POS	NEG	NEG	NEG	NEG	NEG	NEG	NEG	NEG	NEG	NEG	NEG	NEG	NEG	NEG	POS	NEG	POS	POS	POS	POS	NEG	NEG	NEG
NEG	POS	NEG	NEG	NEG	NEG	NEG	NEG	NEG	NEG	NEG	NEG	NEG	NEG	NEG	NEG	POS	NEG	POS	POS	POS	POS	NEG	NEG	NEG
NEG	POS	NEG	NEG	NEG	POS	POS	NEG	NEG	NEG	POS	NEG	NEG	POS	NEG	NEG	POS	NEG	POS	POS	POS	POS	NEG	NEG	NEG
NEG	POS	NEG	NEG	NEG	NEG	NEG	NEG	NEG	NEG	POS	NEG	NEG	NEG	NEG	NEG	POS	NEG	POS	POS	POS	POS	NEG	NEG	NEG
NEG	POS	NEG	NEG	NEG	NEG	NEG	NEG	NEG	NEG	POS	NEG	NEG	NEG	NEG	NEG	POS	NEG	POS	POS	POS	POS	NEG	NEG	NEG
NEG	POS	NEG	NEG	NEG	NEG	NEG	NEG	NEG	NEG	POS	NEG	NEG	NEG	NEG	NEG	POS	NEG	POS	POS	POS	POS	NEG	NEG	NEG
NEG	POS	NEG	NEG	NEG	NEG	NEG	NEG	NEG	NEG	POS	NEG	NEG	NEG	NEG	NEG	POS	NEG	POS	POS	POS	POS	NEG	NEG	NEG
NEG	POS	NEG	NEG	NEG	NEG	NEG	NEG	NEG	NEG	POS	NEG	NEG	NEG	NEG	NEG	POS	NEG	POS	POS	POS	POS	NEG	NEG	NEG
NEG	POS	NEG	NEG	NEG	NEG	NEG	NEG	NEG	NEG	POS	NEG	NEG	NEG	NEG	NEG	POS	NEG	POS	POS	POS	POS	NEG	NEG	NEG
NEG	POS	NEG	NEG	NEG	NEG	NEG	NEG	NEG	NEG	POS	NEG	NEG	NEG	NEG	NEG	POS	NEG	POS	POS	POS	POS	NEG	NEG	NEG
NEG	POS	NEG	NEG	NEG	NEG	NEG	NEG	NEG	NEG	NEG	NEG	NEG	NEG	NEG	NEG	POS	NEG	POS	NEG	NEG	POS	NEG	NEG	NEG
NEG	POS	NEG	NEG	NEG	POS	POS	NEG	NEG	NEG	POS	NEG	NEG	POS	NEG	NEG	POS	NEG	POS	POS	POS	POS	NEG	NEG	NEG
NEG	NEG	POS	NEG	NEG	NEG	NEG	NEG	NEG	NEG	NEG	NEG	NEG	NEG	NEG	POS	POS	NEG	POS	NEG	NEG	NEG	POS	POS	NEG
NEG	POS	NEG	NEG	NEG	POS	NEG	NEG	POS	NEG	POS	NEG	NEG	NEG	NEG	NEG	POS	NEG	POS	NEG	NEG	NEG	POS	POS	NEG
NEG	NEG	NEG	NEG	NEG	NEG	NEG	NEG	NEG	NEG	NEG	POS	NEG	NEG	NEG	NEG	POS	NEG	POS	POS	AMB	POS	POS	POS	NEG
NEG	NEG	NEG	NEG	NEG	NEG	NEG	NEG	NEG	NEG	NEG	POS	NEG	NEG	NEG	NEG	POS	NEG	POS	POS	POS	POS	POS	POS	NEG
NEG	NEG	NEG	NEG	NEG	NEG	NEG	NEG	NEG	NEG	NEG	POS	NEG	NEG	NEG	NEG	POS	NEG	POS	POS	POS	POS	POS	POS	NEG
NEG	POS	NEG	NEG	NEG	POS	NEG	NEG	NEG	NEG	POS	NEG	NEG	NEG	NEG	POS	NEG	NEG	POS	NEG	NEG	NEG	POS	POS	NEG
NEG	NEG	NEG	NEG	POS	NEG	NEG	NEG	POS	POS	NEG	NEG	NEG	NEG	NEG	POS	POS	POS	POS	POS	NEG	NEG	POS	NEG	POS
NEG	NEG	NEG	NEG	POS	NEG	NEG	NEG	NEG	NEG	AMB	NEG	NEG	NEG	NEG	POS	POS	NEG	POS	NEG	NEG	NEG	POS	NEG	NEG
NEG	NEG	NEG	NEG	POS	NEG	NEG	NEG	NEG	NEG	AMB	NEG	NEG	NEG	NEG	POS	POS	NEG	POS	NEG	NEG	NEG	POS	NEG	NEG
NEG	NEG	NEG	NEG	POS	NEG	NEG	NEG	NEG	NEG	NEG	NEG	NEG	NEG	NEG	POS	POS	NEG	POS	NEG	NEG	NEG	NEG	NEG	NEG
NEG	NEG	NEG	NEG	POS	NEG	NEG	NEG	NEG	NEG	POS	AMB	NEG	NEG	NEG	POS	POS	NEG	POS	NEG	NEG	NEG	POS	NEG	NEG
NEG	NEG	NEG	NEG	NEG	NEG	NEG	NEG	POS	NEG	NEG	NEG	POS	NEG	NEG	NEG	POS	NEG	POS	NEG	NEG	NEG	POS	NEG	NEG
NEG	NEG	NEG	NEG	NEG	NEG	NEG	NEG	POS	NEG	POS	NEG	NEG	NEG	NEG	POS	POS	NEG	POS	NEG	NEG	NEG	POS	POS	NEG
NEG	NEG	NEG	NEG	NEG	POS	NEG	NEG	POS	NEG	POS	NEG	NEG	NEG	NEG	POS	POS	NEG	POS	NEG	POS	NEG	POS	POS	NEG
NEG	NEG	NEG	NEG	NEG	NEG	NEG	NEG	POS	NEG	NEG	NEG	NEG	NEG	NEG	POS	POS	NEG	POS	NEG	POS	NEG	POS	NEG	NEG
NEG	NEG	NEG	NEG	NEG	POS	NEG	NEG	POS	NEG	POS	NEG	NEG	NEG	NEG	POS	POS	NEG	POS	NEG	POS	NEG	POS	POS	NEG
NEG	NEG	NEG	NEG	NEG	AMB	NEG	NEG	POS	NEG	POS	NEG	NEG	NEG	NEG	POS	POS	NEG	POS	NEG	POS	NEG	POS	POS	NEG
POS	NEG	NEG	NEG	NEG	POS	NEG	NEG	NEG	POS	POS	NEG	NEG	NEG	NEG	NEG	POS	NEG	POS	NEG	POS	NEG	POS	POS	NEG
NEG	NEG	NEG	NEG	NEG	AMB	NEG	NEG	NEG	NEG	POS	NEG	NEG	NEG	NEG	POS	POS	NEG	POS	NEG	NEG	NEG	POS	POS	NEG
NEG	NEG	NEG	NEG	NEG	POS	NEG	NEG	NEG	NEG	POS	NEG	NEG	NEG	NEG	POS	POS	NEG	POS	NEG	NEG	NEG	POS	POS	NEG
NEG	NEG	NEG	NEG	NEG	POS	NEG	NEG	NEG	NEG	POS	NEG	NEG	NEG	NEG	POS	POS	NEG	POS	NEG	NEG	NEG	POS	POS	NEG
NEG	NEG	NEG	NEG	NEG	AMB	NEG	NEG	NEG	NEG	POS	NEG	NEG	NEG	NEG	POS	POS	NEG	POS	NEG	NEG	NEG	POS	POS	NEG
NEG	NEG	NEG	NEG	NEG	AMB	NEG	NEG	NEG	NEG	POS	NEG	NEG	NEG	NEG	POS	POS	NEG	POS	NEG	NEG	NEG	POS	POS	NEG
NEG	NEG	NEG	NEG	NEG	NEG	NEG	NEG	NEG	NEG	POS	NEG	NEG	NEG	NEG	POS	POS	NEG	POS	NEG	NEG	NEG	POS	POS	NEG
NEG	NEG	NEG	NEG	NEG	NEG	NEG	NEG	NEG	NEG	POS	NEG	NEG	NEG	NEG	POS	POS	NEG	POS	NEG	NEG	NEG	POS	POS	NEG
NEG	NEG	NEG	NEG	NEG	NEG	NEG	NEG	NEG	NEG	NEG	NEG	NEG	NEG	NEG	POS	POS	NEG	POS	NEG	NEG	NEG	POS	POS	NEG
NEG	NEG	NEG	NEG	NEG	NEG	NEG	NEG	NEG	NEG	NEG	NEG	NEG	NEG	NEG	POS	POS	NEG	POS	NEG	NEG	NEG	POS	POS	NEG
NEG	NEG	NEG	NEG	NEG	NEG	NEG	NEG	NEG	NEG	AMB	NEG	NEG	NEG	NEG	POS	POS	NEG	POS	NEG	NEG	NEG	POS	POS	NEG
NEG	POS	NEG	NEG	NEG	POS	NEG	NEG	NEG	NEG	POS	NEG	POS	POS	NEG	NEG	POS	POS	POS	POS	NEG	POS	POS	POS	NEG
NEG	POS	NEG	NEG	NEG	AMB	NEG	NEG	NEG	NEG	POS	NEG	POS	POS	NEG	NEG	POS	POS	POS	POS	NEG	POS	POS	POS	NEG
NEG	POS	NEG	NEG	NEG	NEG	NEG	NEG	POS	NEG	POS	NEG	NEG	NEG	NEG	POS	POS	NEG	POS	POS	NEG	POS	NEG	NEG	NEG
NEG	POS	NEG	NEG	NEG	NEG	NEG	NEG	POS	POS	POS	NEG	POS	NEG	NEG	NEG	POS	NEG	POS	POS	NEG	POS	NEG	NEG	POS
NEG	POS	NEG	NEG	NEG	NEG	NEG	NEG	POS	POS	POS	NEG	POS	NEG	NEG	NEG	POS	NEG	POS	POS	NEG	POS	NEG	NEG	NEG
NEG	POS	NEG	NEG	NEG	NEG	NEG	NEG	POS	POS	POS	NEG	POS	NEG	NEG	NEG	POS	NEG	POS	AMB	NEG	POS	NEG	NEG	NEG
NEG	POS	NEG	NEG	NEG	NEG	NEG	NEG	POS	POS	POS	NEG	POS	NEG	NEG	NEG	POS	NEG	POS	POS	NEG	POS	NEG	NEG	POS
NEG	POS	NEG	NEG	NEG	NEG	NEG	NEG	POS	POS	POS	NEG	POS	NEG	NEG	NEG	POS	NEG	POS	POS	NEG	POS	NEG	NEG	POS
NEG	POS	NEG	NEG	NEG	NEG	NEG	NEG	POS	POS	POS	NEG	POS	NEG	NEG	NEG	POS	NEG	POS	POS	NEG	POS	NEG	NEG	POS
NEG	POS	NEG	NEG	NEG	NEG	NEG	NEG	POS	POS	POS	NEG	POS	NEG	NEG	NEG	POS	NEG	POS	AMB	NEG	POS	NEG	NEG	AMB
NEG	POS	NEG	NEG	NEG	NEG	NEG	NEG	POS	POS	POS	NEG	POS	NEG	NEG	NEG	POS	NEG	POS	POS	NEG	POS	NEG	NEG	AMB
NEG	POS	NEG	NEG	NEG	NEG	NEG	NEG	POS	POS	POS	NEG	POS	NEG	NEG	NEG	POS	NEG	POS	POS	NEG	POS	NEG	NEG	POS
NEG	POS	NEG	NEG	NEG	NEG	NEG	NEG	POS	POS	POS	NEG	POS	NEG	NEG	NEG	POS	NEG	POS	POS	NEG	POS	NEG	NEG	POS
NEG	POS	NEG	NEG	NEG	NEG	NEG	NEG	POS	POS	POS	NEG	POS	NEG	NEG	NEG	POS	NEG	POS	POS	NEG	POS	NEG	NEG	AMB
NEG	POS	NEG	NEG	NEG	NEG	NEG	NEG	POS	POS	NEG	NEG	POS	NEG	NEG	NEG	POS	NEG	POS	NEG	NEG	POS	NEG	NEG	NEG
NEG	POS	NEG	NEG	NEG	NEG	NEG	NEG	POS	POS	POS	NEG	POS	NEG	NEG	NEG	POS	NEG	POS	POS	NEG	POS	NEG	NEG	NEG
NEG	POS	NEG	NEG	NEG	NEG	NEG	NEG	POS	POS	POS	NEG	POS	NEG	NEG	NEG	POS	NEG	POS	POS	NEG	POS	NEG	NEG	NEG
NEG	POS	NEG	NEG	NEG	NEG	NEG	NEG	POS	POS	POS	NEG	POS	NEG	NEG	NEG	POS	NEG	POS	POS	NEG	POS	NEG	NEG	NEG
NEG	POS	NEG	NEG	NEG	NEG	NEG	NEG	POS	POS	POS	NEG	POS	NEG	NEG	NEG	POS	NEG	POS	POS	NEG	POS	NEG	NEG	NEG
NEG	NEG	POS	NEG	NEG	POS	NEG	NEG	POS	NEG	POS	NEG	NEG	NEG	NEG	NEG	POS	NEG	POS	NEG	POS	NEG	POS	POS	NEG
NEG	POS	NEG	NEG	NEG	POS	POS	NEG	NEG	NEG	POS	NEG	NEG	POS	NEG	NEG	POS	NEG	POS	POS	POS	POS	NEG	NEG	NEG
NEG	POS	NEG	NEG	NEG	POS	POS	NEG	NEG	NEG	POS	NEG	NEG	POS	NEG	NEG	POS	NEG	POS	POS	POS	POS	NEG	NEG	NEG
NEG	POS	NEG	NEG	NEG	POS	POS	NEG	NEG	NEG	POS	NEG	NEG	POS	NEG	NEG	POS	NEG	POS	POS	POS	POS	NEG	NEG	NEG
NEG	POS	NEG	NEG	NEG	POS	POS	NEG	NEG	NEG	POS	NEG	NEG	POS	NEG	NEG	POS	NEG	POS	POS	POS	POS	NEG	NEG	NEG
NEG	POS	NEG	NEG	NEG	POS	POS	NEG	NEG	NEG	POS	NEG	NEG	POS	NEG	NEG	POS	NEG	POS	POS	POS	POS	NEG	NEG	NEG
NEG	POS	NEG	NEG	NEG	POS	POS	NEG	NEG	NEG	POS	NEG	NEG	POS	NEG	NEG	POS	NEG	POS	POS	POS	POS	NEG	NEG	NEG
NEG	POS	NEG	NEG	NEG	POS	POS	NEG	NEG	NEG	POS	NEG	NEG	POS	NEG	NEG	POS	NEG	POS	POS	POS	POS	NEG	NEG	NEG
NEG	NEG	NEG	NEG	NEG	NEG	NEG	NEG	NEG	NEG	NEG	POS	NEG	NEG	NEG	NEG	POS	NEG	POS	POS	NEG	POS	NEG	NEG	NEG
NEG	NEG	NEG	NEG	NEG	NEG	NEG	NEG	NEG	NEG	NEG	POS	NEG	NEG	NEG	NEG	POS	NEG	POS	POS	NEG	POS	NEG	NEG	NEG
NEG	NEG	NEG	NEG	NEG	NEG	NEG	NEG	NEG	NEG	NEG	POS	NEG	NEG	NEG	NEG	POS	NEG	POS	POS	NEG	POS	NEG	NEG	NEG
NEG	NEG	NEG	NEG	NEG	NEG	NEG	NEG	NEG	NEG	NEG	POS	NEG	NEG	NEG	NEG	POS	NEG	POS	POS	NEG	POS	NEG	NEG	NEG
NEG	NEG	NEG	NEG	NEG	NEG	NEG	NEG	NEG	NEG	POS	POS	NEG	NEG	NEG	NEG	POS	NEG	POS	POS	NEG	POS	NEG	NEG	AMB
POS	NEG	NEG	NEG	NEG	NEG	NEG	NEG	NEG	NEG	NEG	AMB	NEG	NEG	NEG	NEG	NEG	AMB	POS	POS	NEG	POS	POS	POS	NEG
POS	NEG	NEG	NEG	NEG	NEG	NEG	NEG	NEG	NEG	NEG	NEG	NEG	NEG	NEG	NEG	NEG	AMB	AMB	POS	NEG	POS	POS	POS	NEG
