# Supplementary material for: Clonal Complexes Distribution of Staphylococcus aureus Isolates from Clinical Samples from the Caribbean Islands
Source: Antibiotics (Basel). 2023 Jun 14;12(6):1050. doi: 10.3390/antibiotics12061050 (PMC10295549; doi:10.3390/antibiotics12061050)

Supplemental file S2: Alignment of *hld* and the *agr* genes of the CC8 reference sequence NCTC8325, CP000253.1, the PVL-non-producer Trinidad&Tobago_2020-042_7641M (TT-042) and the PVL-producing isolate
Trinidad&Tobago_2020-043_7352M (TT-043). The red arrow indicates a deletion in *agrC*-1 of TT-042.

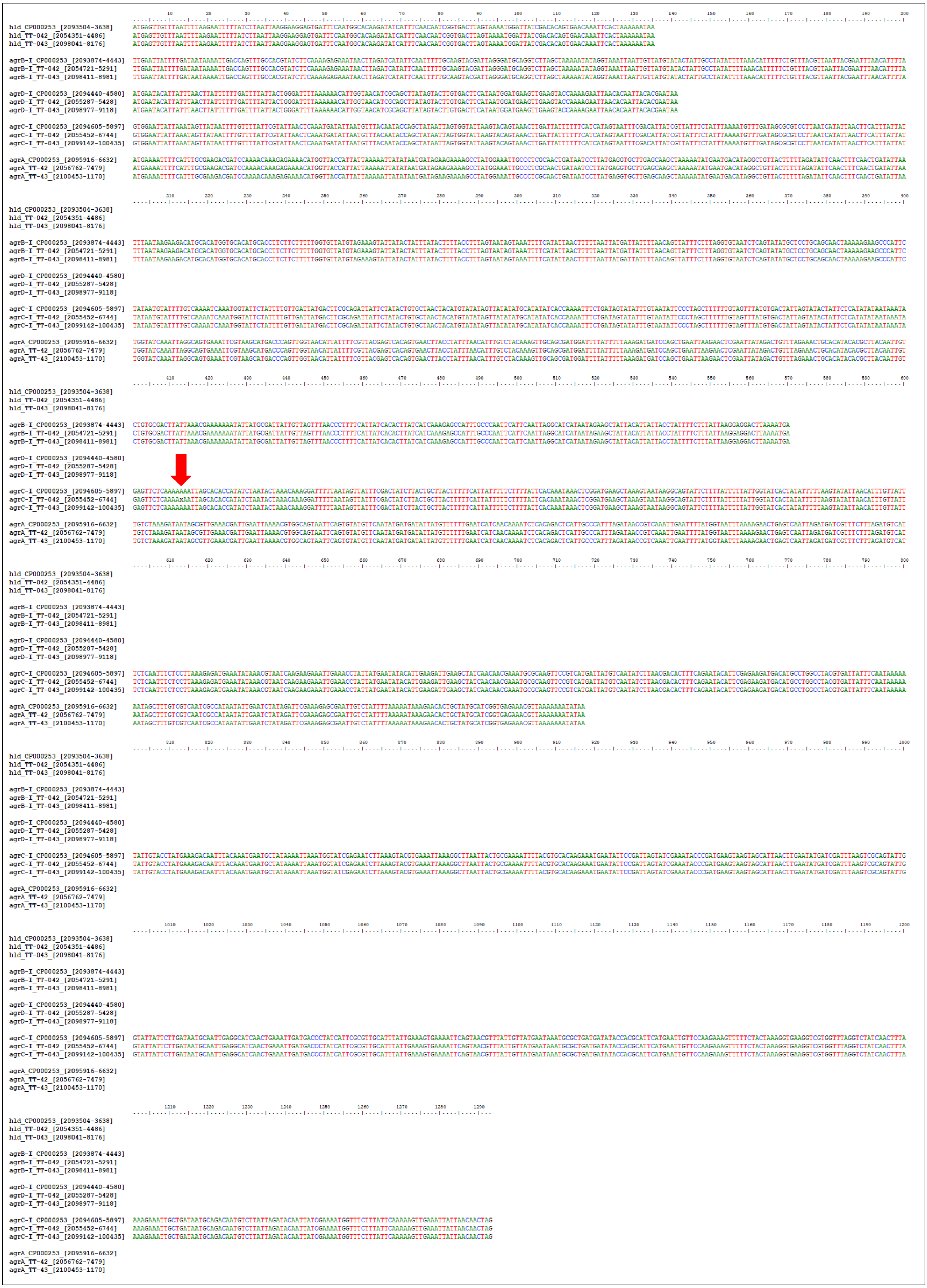

Supplement: Supplementary file 1 [file antibiotics-12-01050-s001.zip › Supplemental File S3_hld and agr alignment.pdf]
